# Bis-pyrene probes of foldamer conformation in solution and in phospholipid bilayers†
†Electronic supplementary information (ESI) available: Synthetic procedures and NMR spectra for new compounds, analytical procedures and data for fluorescence studies and X-ray crystallography data. CCDC 1843678–1843682. For ESI and crystallographic data in CIF or other electronic format see DOI: 10.1039/c8sc02532k


**DOI:** 10.1039/c8sc02532k

**Published:** 2018-07-17

**Authors:** Francis G. A. Lister, Natasha Eccles, Sarah J. Pike, Robert A. Brown, George F. S. Whitehead, James Raftery, Simon J. Webb, Jonathan Clayden

**Affiliations:** a School of Chemistry , University of Manchester , Oxford Road , Manchester M13 9PL , UK . Email: S.Webb@manchester.ac.uk; b Manchester Institute of Biotechnology , University of Manchester , 131 Princess St , Manchester M1 7DN , UK; c Faculty of Life Sciences , University of Bradford , Bradford , West Yorkshire BD7 1DP , UK; d School of Chemistry , University of Bristol , Cantock's Close , Bristol BS8 1TS , UK . Email: j.clayden@bristol.ac.uk

## Abstract

Exploring the detailed structural features of synthetic molecules in the membrane phase requires sensitive probes of conformation. Here we describe the design, synthesis and characterization of bis(pyrene) probes that report conformational changes in membrane-active dynamic foldamers. The probes were designed to distinguish between left-handed (*M*) and right-handed (*P*) screw-sense conformers of 3_10_-helical α-aminoisobutyric acid (Aib) peptide foldamers, both in solution and in bilayer membranes. Several different bis(pyrene) probes were synthesized and ligated to the C-terminus of Aib tetramers that had different chiral residues at the N-terminus, residues that favored either an *M* or a *P* screw-sense in the 3_10_-helix. The readily synthesized and conveniently incorporated *N*-acetyl-1,2-bis(pyren-1′-yl)ethylenediamine probe proved to have the best properties. In solution, changes in foldamer screw-sense induced substantial changes in the ratio of excimer/monomer fluorescence emission (*E*/*M*) for this reporter of conformation, with X-ray crystallography revealing that opposite screw-senses produce very different interpyrene distances in the reporter. In bilayers, this convenient and sensitive fluorescent reporter allowed, for the first time, an investigation of how the chirality of natural phospholipids affects foldamer conformation.

## Abstract 2

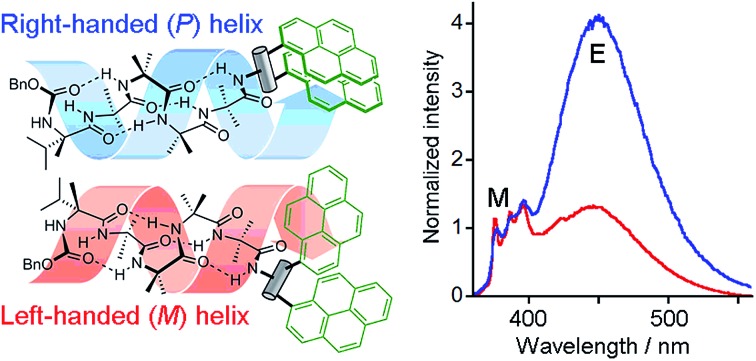
An optimized fluorescent probe, developed using spectroscopic and crystallographic analysis, reported on peptide foldamer conformation in different environments and revealed that phospholipid chirality can influence conformation.

## Introduction

Conformational control of configurationally achiral helical folded oligomers (foldamers) has recently been shown to provide a route to biomimetic nanoscale molecular devices capable of transmitting information over multi-nanometer distances.^1^ Foldamers composed of α-aminoisobutyric acid (Aib) have proven to be particularly promising in this respect.^2^ Aib foldamers with at least four Aib residues can fold into 3_10_-helices, in which left-handed (*M*) and right-handed (*P*) screw-senses are equally populated owing to the lack of chirality in an Aib residue.^3^ Short Aib foldamers interconvert rapidly between screw-senses on the typical ^1^H NMR spectroscopy timescale,3*a* allowing any induced imbalance in the ratio of *M* to *P* helices to give rise to diagnostic changes in the NMR spectra of appropriately designed foldamers.^4^

The conformational uniformity of Aib oligomers1*e* means that a conformational preference induced by a chiral group, a “controller”, at one terminus of the helix, propagates throughout the foldamer and leads to an imbalance in the ratio of *M* and *P* helical conformers.^5^ Covalent or non-covalent interaction of this controller with the foldamer, typically at its N-terminus, can be viewed as a form of information input. This information, encoded in the conformational preference of the oligomer, can be read at the distal terminus of the foldamer by a group, a “reporter”, which is designed to give a diagnostic spectroscopic output.

NMR spectroscopy is a powerful tool for studying conformational interchange and conformational preferences in Aib foldamers in isotropic solution. ^1^H, ^19^F and ^13^C NMR spectroscopic reporters have been incorporated into Aib foldamers, and used to report on conformational control from both the N- and C-termini.^5^,^6^ For example, a ^13^C NMR spectroscopic probe was used to report on dynamic exchange of chiral messengers at the N-terminus of a class of boron-containing dynamic foldamer, allowing the development of a synthetic system that functions in organic solvent to mimic the behavior of purinergic receptors.^7^

Nonetheless, true biological receptors in the G protein coupled receptor class function as membrane-embedded structures. Transferring foldamer-based biomimetic receptors from organic solvent into a phospholipid bilayer, the environment experienced by most natural receptors, presents several analytical challenges. ^1^H or ^13^C signals of NMR-detectable reporters will be masked by signals from lipids, especially as the reporter should be at a low loading in the membrane. The anisotropic membrane environment will also broaden NMR signals. We have shown that ^19^F NMR spectroscopy6*c* with magic-angle spinning can overcome these problems to some extent, but the spectral acquisition times are long, high concentrations of multilamellar vesicles are required, and samples must be spun rapidly before spectral acquisition, preventing real-time detection of conformational changes.^8^ A more biomimetic system would use unilamellar vesicles with distinct interior and exterior environments. To explore the function of dynamic foldamers in intact unilamellar vesicles, alternative spectroscopic techniques were needed.

Fluorescent probes are compatible with membrane environments and can report on changes in orientation and distance between chromophores,^9^ for example through Förster resonance energy transfer, reversible electronic energy transfer^10^ and excimer formation.^11^ We recently reported a successful application of fluorescent reporters of dynamic conformational change in a membrane environment,^12^ and we now describe in detail the design, development and analysis of membrane-compatible fluorescent probes of conformation.

**Fig. 1:**
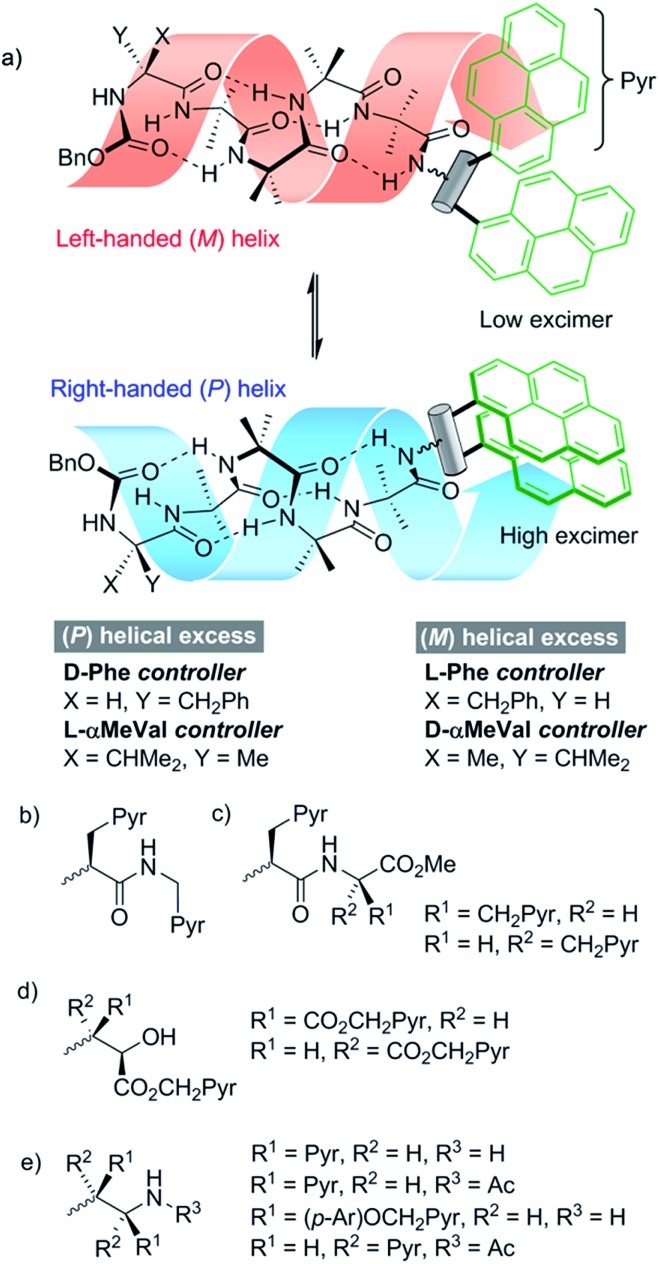
Development of chiral, conformationally responsive fluorescent probes. Pyr = pyren-1-yl. *p*-Ar = 1,4-disubstituted benzene. (a) Schematic bis(pyren-1-yl) probe attached to *M* and *P* screw-sense conformers of a controlled Aib_4_ helix; (b), (c) reporters incorporating one or two pyrenylalanine (Pya) residues; (d) reporters derived from aminoalcohol probes; (e) reporters derived from 1,2-bis(aryl)ethylenediamine probes.

We opted to explore the use of excimer emission from pyrene chromophores.^13^ The emission spectrum of pyrene varies according to its proximity to other pyrene moieties. When two pyrene molecules are less than ∼10 Å apart, an excited dimer (excimer) can form that has broad emission at longer wavelengths (425–550 nm).^14^ Incorporating two pyrene rings (Pyr, Fig. 1a) into a fluorescent reporter group may allow conformational changes in the reporter to vary the distance between the pyrene rings, modulating the intensity of the excimer emission.

Since screw-sense conformers are enantiomeric, any conformational changes induced in an achiral reporter would not give rise to a change in conventional fluorescence emission spectra. A chiral reporter would nonetheless give rise to diastereoisomeric conformers with different emission properties, allowing fluorescence spectroscopy to report on changes in screw-sense preference. However, the use of a chiral reporter brings with it a potential problem, as it must be conformationally responsive without exerting a strong chiral influence over the foldamer helix. Provided also that intermolecular excimer formation is avoided, such probes would be powerful tools that can report on conformational change in both solution and membrane phases. By correlating these changes in fluorescence with established data from NMR spectroscopy, a quantitative interpretation of the fluorescence changes should be possible.

## Results and discussion

Our strategy for the development of bis(pyrene) probes is illustrated in Fig. 1a. A successful probe would exhibit detectable differences in the ratio of excimer to monomer emission (*E*/*M* ratio) when ligated to the C-terminus of a foldamer helix carrying a range of N-terminal chiral controllers with different abilities to favor either *M* or *P* screw-senses. An Aib_4_ helical foldamer scaffold was selected, as Aib_4_ is rapidly synthetically accessible yet long enough to fold into a single turn 3_10_-helix. It is also long enough to prevent direct, rather than conformationally relayed, interaction between chiral controller and reporter.^5^ Furthermore, a wide range of chiral controllers, such as Cbz(α-methylvaline) [Cbz(α-MeVal)],1*e* have been covalently attached to the N-terminus of Aib_4_ foldamers and the extent to which they each favor either *M* or *P* helical conformations has been quantified by NMR spectroscopy;^5^ this provides data for the calibration of the fluorescent response from bis(pyrene) probes.

Aib_4_ foldamers with a defined screw-sense preference arising from chiral N-terminal residues, along with achiral Aib_4_ foldamers as control compounds, were ligated to three generations of bis(pyrene) probes (Fig. 1b–e). The first generation of probes were formed from pyrenylalanine, with the second generation derived from l-tartrate and the third generation based upon 1,2-bis(aryl)ethylenediamine building blocks. All of these probe designs have the potential to donate hydrogen bond(s) back into the 3_10_-helix, increasing the likely sensitivity of their conformational preference to the sense of the helix. The most promising probes were then optimized and subsequently used in bilayer studies.

### Development of pyrenylalanine-based probes

**Scheme 1:**
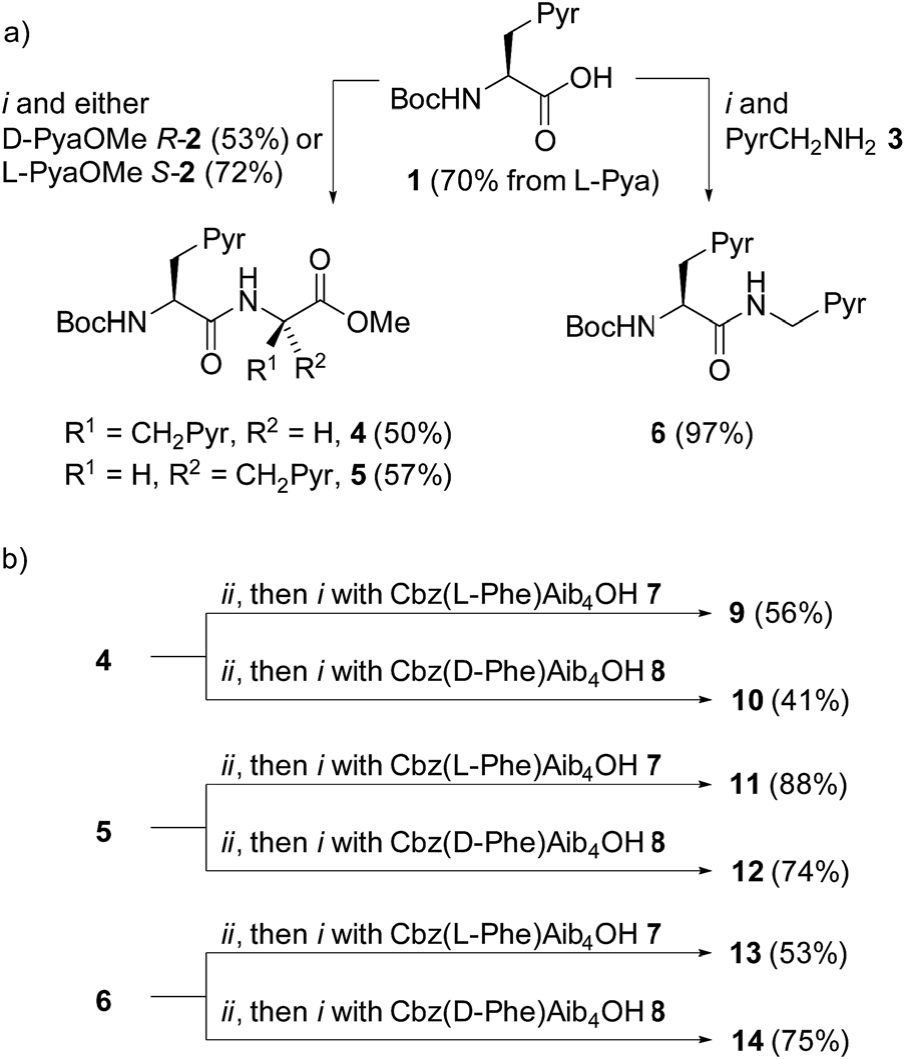
(a) Synthesis of ‘first generation’ probes **4–6**. Pyr = pyren-1-yl. (i), EDC, HOBt, i-Pr_2_NEt, CH_2_Cl_2_, RT. (b) Synthesis of foldamers **9–14** bearing first generation probes. (ii), TFA/CH_2_Cl_2_.

A simple way to functionalize an Aib foldamer helix with pyren-1-yl groups was to incorporate adjacent 3-(pyren-1′-yl)alanine (Pya) residues (Fig. 1b and c). Synthetic routes to Pya are well documented,^15^ and Sisido *et al.* have incorporated Pya into helical structures both as poly(l-Pya)^16^ and as a bis-Pya label in the backbone of a short α-helical polymer.^17^ Dimers of Pya were used by De Schryver and coworkers to analyze dipeptide conformations.15c,^18^ Encouraged by these prior studies, diastereoisomeric dimers **4** and **5**, along with 3-(pyren-1′-yl)alanine (pyren-1′-ylmethyl)amide **6** were our first probe targets.

l- and d-isomers of *N*-BocPya methyl ester were synthesized following the procedures of Egusa *et al.*,^19^ which first provided l- and d-Pya in 80% and 94% ee respectively. Subsequent protection, as a methyl ester to give **2** or as the *N*-Boc derivative to give **1**, was followed by coupling to give target probes **4**, **5** and **6** (Scheme 1a). *N*-Deprotection gave poorly soluble dipeptides or amides that were coupled directly to the C-terminus of enantiomeric CbzPheAib_4_OH helices to give three pairs of diastereoisomeric foldamers **9–14** (Scheme 1).

Two problems were immediately evident with these ‘first generation’ structures. Firstly, no excimer fluorescence was detectable from any of these compounds (see the ESI†). Furthermore, circular dichroism spectra from each member of the three diastereoisomeric pairs were almost identical, indicating that their conformation was similar, despite the opposite absolute configuration of the N-terminal controller. This implies that, despite the reported dominance of N- over C-terminal controllers,^20^ the conformational preference imparted by these chiral fluorescent probes is too powerful, overpowering the influence of the N-terminal CbzPhe residue.

**Fig. 2:**
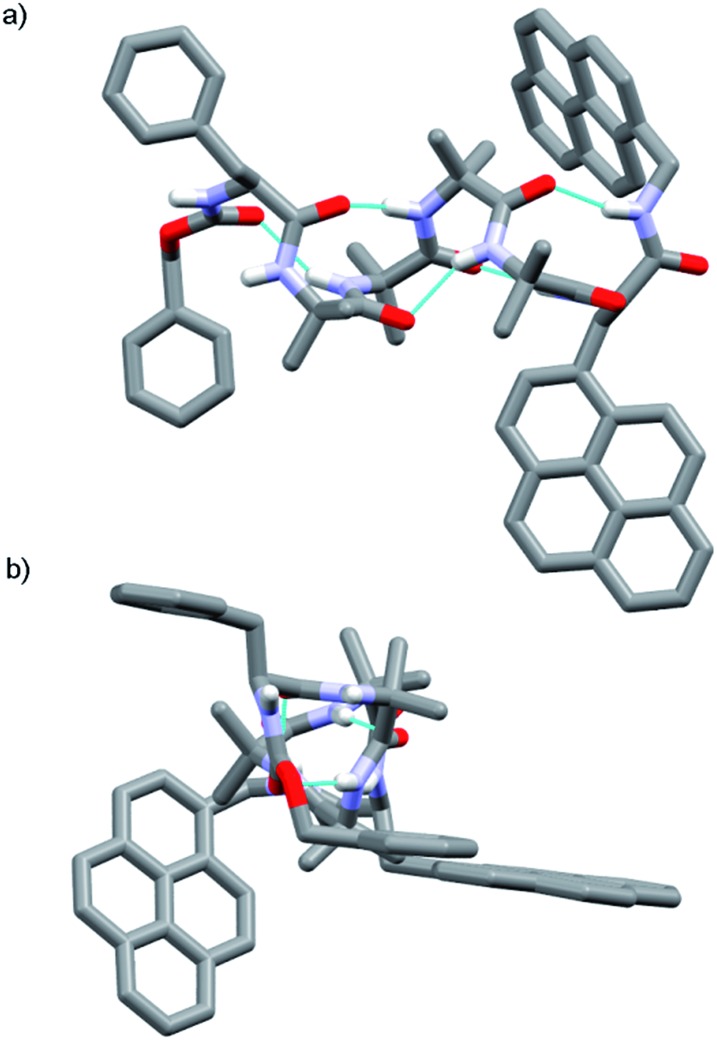
X-ray crystal structure of Cbz(l-Phe)Aib_4_(l-Pya)NHCH_2_Pyr **13**, which adopts a right-handed (*P*) 3_10_-helix. (a) Viewed side-on; (b) view down helix axis.

The X-ray crystal structure of **13** (Fig. 2) supported this interpretation. The Aib_4_ helix adopts a right-handed conformation as a consequence of the single C-terminal l-Pya residue, with the l-Phe incorporated into a type III β-turn, rather than the left-handed helical conformation typically induced by an N-terminal Cbz(l-Phe) residue in a type II β-turn.^4^,^21^

The lack of excimer fluorescence from the first generation probes prompted us to explore structures with the pyren-1-yl-bearing groups closer to each other, separated by a single rotatable bond. Such 1,2-difunctionalised motifs are available either from the natural chiral pool (*e.g.* by transformation of l-tartrate) or by asymmetric synthesis.

### Development of 1,2-aminoalcohol-based probes

**Scheme 2:**
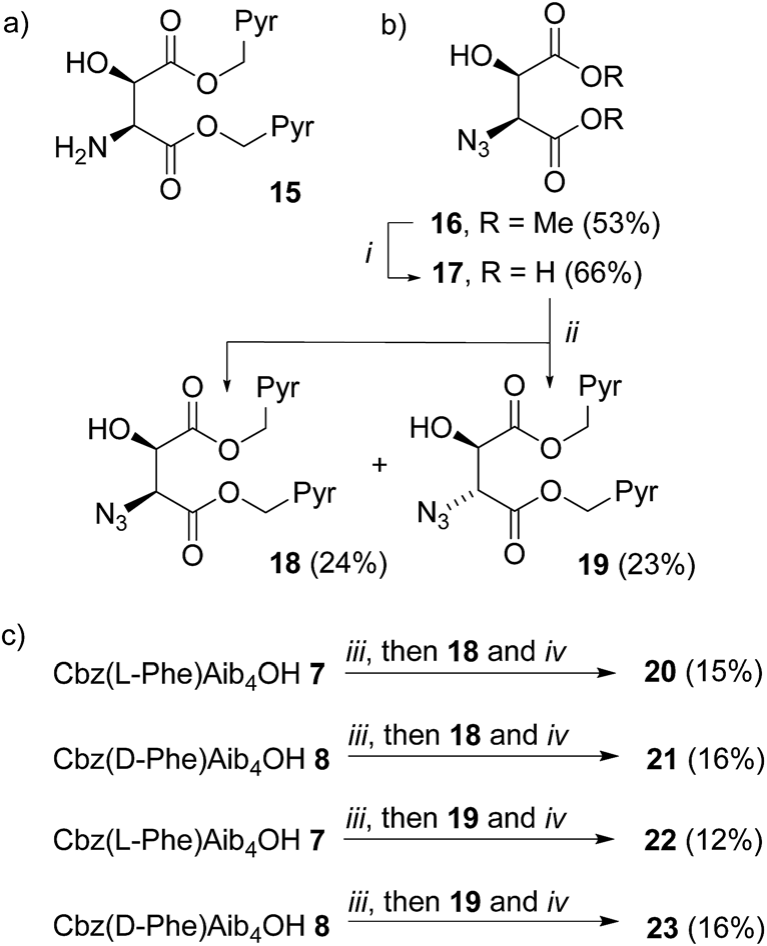
(a) Example of a bis(pyrene) aminoalcohol probe for attachment to the C-terminus of foldamers; (b) synthesis of ‘second generation’ probes **18–19**. Pyr = pyren-1-yl. (i), LiI, THF reflux, 48 h then 1 M HCl. (ii), 1-(Bromomethyl)pyrene, Et_3_N, MeCN, reflux, 72 h; (c) coupling to foldamers **7** and **8**, (iii), EDC, HOBt, THF, RT, 30 min. (iv), PEt_3_, THF, RT, 16 h.

A C-terminal chiral aminoalcohol reporter (Fig. 1d) derived from probe **15** (Scheme 2) would have a structure similar to the probe used by Koert and coworkers to detect conformational changes in a perhydroanthracene ring.^22^ Furthermore, earlier work in our group6a,c had indicated that a hydroxylated C-terminus forms a favorable hydrogen bond with the terminal hydrogen-bond acceptors of the 3_10_-helix. The consequent reduction in conformational freedom allowed a detectable response to changes in screw-sense preference in an analogous NMR spectroscopic probe.

Diester **16** was synthesized from l-tartrate by the method of Breuning *et al.*,^23^ hydrolyzed, and alkylated by heating to reflux with bromomethylpyrene and triethylamine in dry acetonitrile for 72 h. ^1^H NMR spectroscopy of the crude product mixture indicated epimerization to a 1.2 : 1 mixture of the diastereoisomeric azides **18** and **19**. These diastereoisomers were separated by chromatography, and one-pot Staudinger–Vilarrasa coupling of either **18** or **19** to either **7** or **8** yielded sufficient amounts of pure foldamers **20–23** for fluorimetric studies (Scheme 2).

Strong excimer emission in the fluorescence spectra of **20–23** indicates that the pyrene groups are in close proximity. However, there were only small differences in the *E*/*M* ratio between foldamers with either a Cbz(d-Phe) or a Cbz(l-Phe) N-terminal controller. The ^1^H NMR spectra of peptides **20–23** in CDCl_3_ solution revealed similar chemical shifts and coupling constants for the backbone α-protons of the fluorophore unit, hinting that the C-terminal reporter group adopts a similar conformation in all cases (see the ESI†). In contrast, the differences between the resonances of the Cbz(d-Phe) and Cbz(l-Phe) residues the N-terminus are more noticeable, suggesting that, like the first generation probes, these 1,2-aminoalcohol-based reporter groups exert too much control over helical screw-sense.

### Development of 1,2-diamine-based probes

**Scheme 3:**
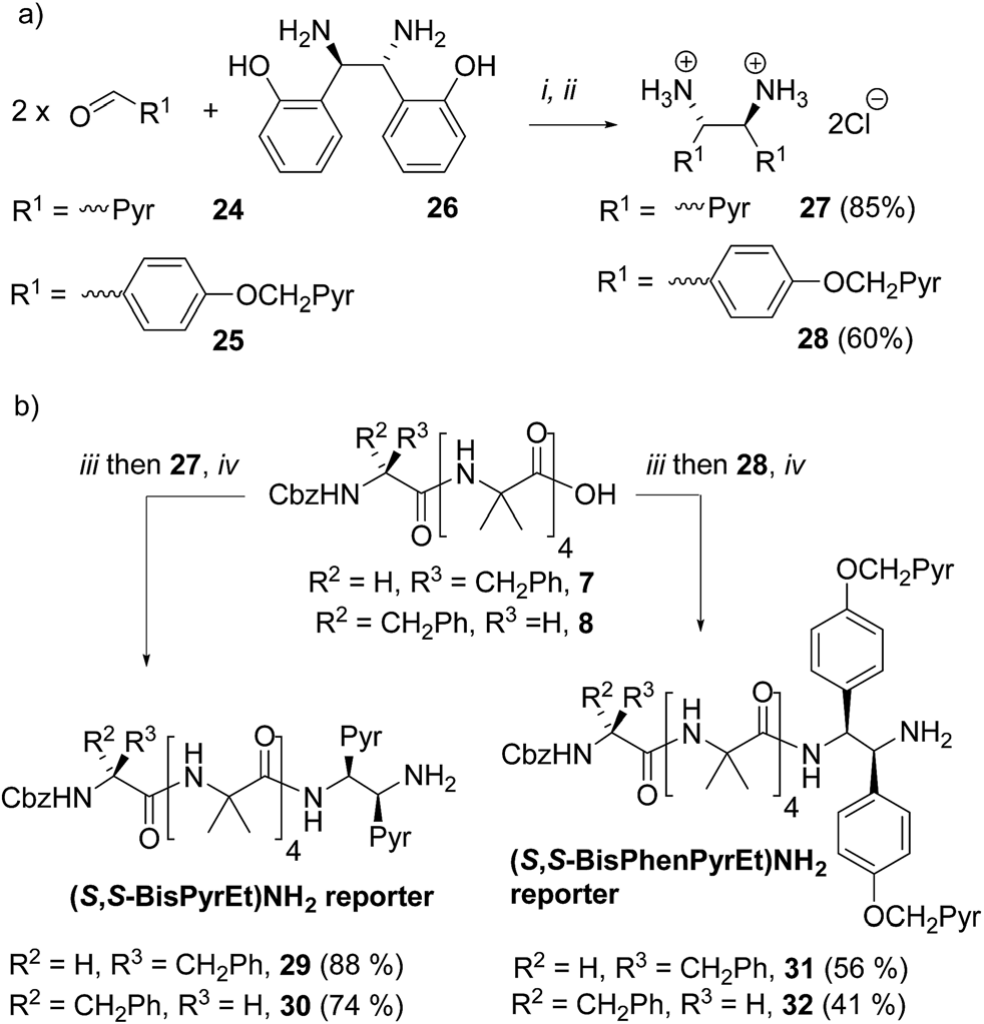
(a) Synthesis of ‘third generation’ probes **27** and **28**. Pyr = pyren-1-yl. (i), DMSO, 3 h. then H_2_O. (ii) THF, Conc. HCl; (b) synthesis of foldamers **29–32** bearing third generation probes. (iii), HOBt, EDC·HCl, CH_2_Cl_2_. (iv), i-Pr_2_NEt, RT, 2 d.

A final ‘third generation’ of bis(pyrene)-containing probes (Fig. 1e and Scheme 3a and b) was designed based around a chiral, 1,2-disubstituted ethylenediamine unit. In diamine **27**, the two pyrene systems are closer than in the previous probe structures. Diamine **28**, with longer, rigid phenolic arms, was made in the expectation that torsional changes around the central C–C bond would be amplified into more significant changes in the relative positions of the pyrene fluorophores.

The diamines were made using the method of Chin and co-workers.^24^ 1-Pyrenylaldehyde **24** was mixed with (1*R*,2*R*)-1,2-bis(2′-hydroxyphenyl)ethylenediamine **26** in DMSO. After 3 h the solution was poured into water to precipitate the crude diimine. After re-dissolution in THF and reaction with HCl, analytically pure (1*S*,2*S*)-1,2-bis(1′-pyrene)ethylenediamine dihydrochloride **27** was obtained. Similarly, 4-(pyren-1′-ylmethoxy)benzaldehyde **25** was treated with **26** using an identical method to give (1*S*,2*S*)-1,2-bis(4′-(pyren-1-ylmethoxy)phenyl)ethane-1,2-diamine dihydrochloride **28** (Scheme 3a).

**Fig. 3:**
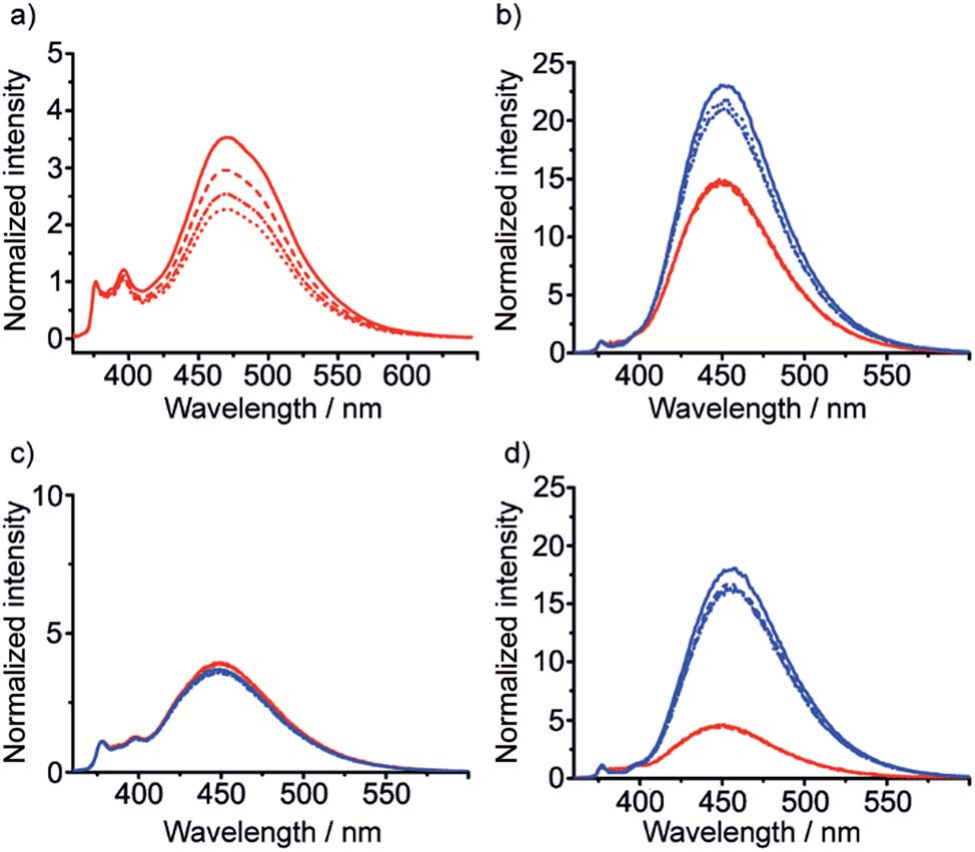
Emission spectra normalized to 1 at 377 nm from (a) **31** in MeOH; (b) **29** (*M* helix, red traces) and **30** (*P* helix, blue traces) in MeOH; (c) **29** (red traces) and **30** (blue traces) in CH_2_Cl_2_; (d) **29** (red traces) and **30** (blue traces) in MeCN. Foldamer concentrations: 10 μM (solid line) 5 μM (dashed line) 2.5 μM (dash-dotted line) and 1.25 μM (dotted line).

These diamine probes were coupled to the enantiomeric pair of CbzPhe capped Aib tetramers **7** and **8** to give foldamers **29–32** (Scheme 3b). Encouragingly all four foldamers showed high levels of excimer emission. However, dilution studies of **31** (10 μM to 1.25 μM) showed that excimer emission from this long (*S*,*S*-BisPhenPyrEt)NH_2_ reporter had a significant component arising from intermolecular interactions, even in methanol (Fig. 3a). In contrast, dilution studies on **29** and **30**, which have foldamers ligated to the shorter diamine probe **27**, showed a smaller contribution to the excimer emission from intermolecular interactions (Fig. 3b–d). Most importantly, the diastereoisomeric pairs of foldamers showed significant, measurable differences in *E*/*M* ratio in CH_3_CN and CH_3_OH, if not CH_2_Cl_2_ (Fig. 3b–d). Further studies were therefore performed on foldamers incorporating the diamine probe **27**. The resulting C-terminal reporter group, denoted as (*S*,*S*-BisPyrEt)NH_2_, had the best performance of the reporters tested by that point.

### Optimization of 1,2-diamine-based probes

**Scheme 4:**
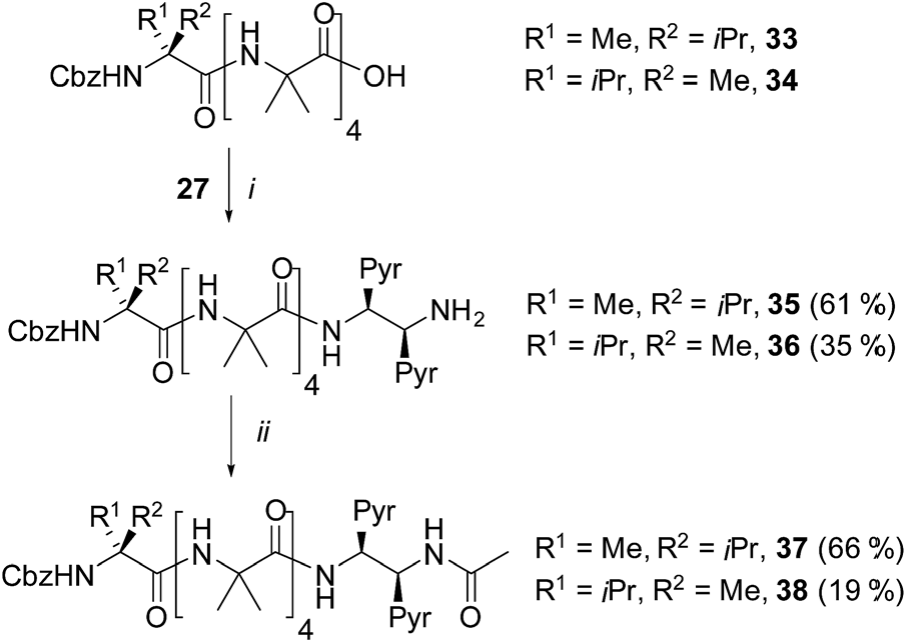
Synthesis of α-MeVal controlled foldamers **35–38**. (i), EDC/HOBt, i-Pr_2_NEt, CH_2_Cl_2_; (ii), Ac_2_O, CH_2_Cl_2_.

The performance of the (*S*,*S*-BisPyrEt)NH_2_ conformational reporter derived from third generation probe **27** was characterized as a function of screw-sense control from the N-terminus. To do this, additional foldamers with an N-terminal Cbz(α-MeVal) residue were synthesized: the ability of these N-terminal residues to induce a preferred screw-sense in an Aib foldamer is known to be relatively solvent-independent.^5^ Either l- or d-Cbz(αMeVal)Aib_4_OH were coupled to probe **27** to give the diastereoisomers **35** and **36** (Scheme 4).^5^

**Fig. 4:**
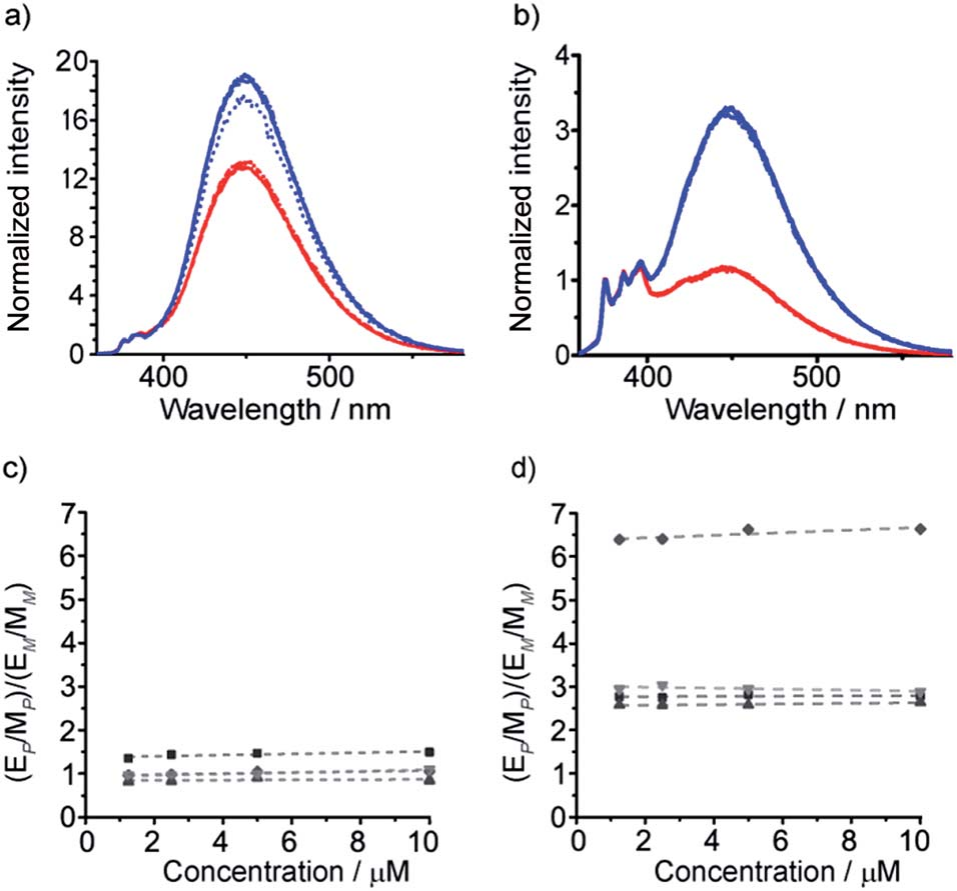
(a, b) Fluorescence emission spectra (normalized to 1 at 377 nm) in MeOH of (a) amino-terminated foldamers **35** (*P* helix, blue trace) and **36** (*M* helix, red trace); (b) acetamide-terminated foldamers **37** (blue) and **38** (red) in MeOH. Spectra shown for 10 μM (solid line) 5 μM (dashed line) 2.5 μM (dash-dotted line) and 1.25 μM (dotted line) of compound; (c, d) sensitivity ratio (*S*_R_) values for (c) **35** and **36**; (d) **37** and **38**. *S*_R_ = ([*E*/*M* for *P*]/[*E*/*M* for *M*]) for the diastereomeric pairs in CH_2_Cl_2_ (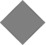) MeOH (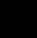) MeCN (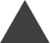) and THF (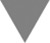) at different concentrations. For **35** and **36** the response in CH_2_Cl_2_ is identical to that in THF.

It was also hoped that C-terminal acetylation would optimize the performance of this reporter group, as the acetamide produced might promote hydrogen bonding from the C-terminal N–H back into the 3_10_-helix. Therefore both **35** and **36** were acetylated to give the diastereoisomeric foldamer pair **37** and **38**, each of which bear a modified C-terminal reporter, denoted as (*S*,*S*-BisPyrEt)NHAc. This acetylation of the (*S*,*S*-BisPyrEt)NH_2_ reporter to produce the (*S*,*S*-BisPyrEt)NHAc reporter resulted in a clear increase in performance (Fig. 4).

Dividing the *E*/*M* ratio of the fluorescent reporter in a *P* helical environment by the *E*/*M* ratio of the same reporter in an *M* helical environment gives a sensitivity ratio (*S*_R_ = (*E*/*M* for *P*-helix)/(*E*/*M* for *M*-helix)) that provides a measure of the probe's ability to report on conformational change: the greater the deviation from 1, the greater the sensitivity. The amine-terminated reporter (*S*,*S*-BisPyrEt)NH_2_ in **35** and **36** had *S*_R_ values close to 1 in the four different solvents tested (Fig. 4c). The results for the acetamide-terminated reporter (*S*,*S*-BisPyrEt)NHAc in **37** and **38** highlighted its superiority. In polar solvents the *E*/*M* ratio for the *P* helix was three times that of the *M* helix. Even in CH_2_Cl_2_, where the amine-terminated reporter in **35** and **36** was insensitive to different conformations, the *E*/*M* ratio for the *P* helix was found to be 6.4 times that of the *M* (Fig. 4d).

**Scheme 5:**
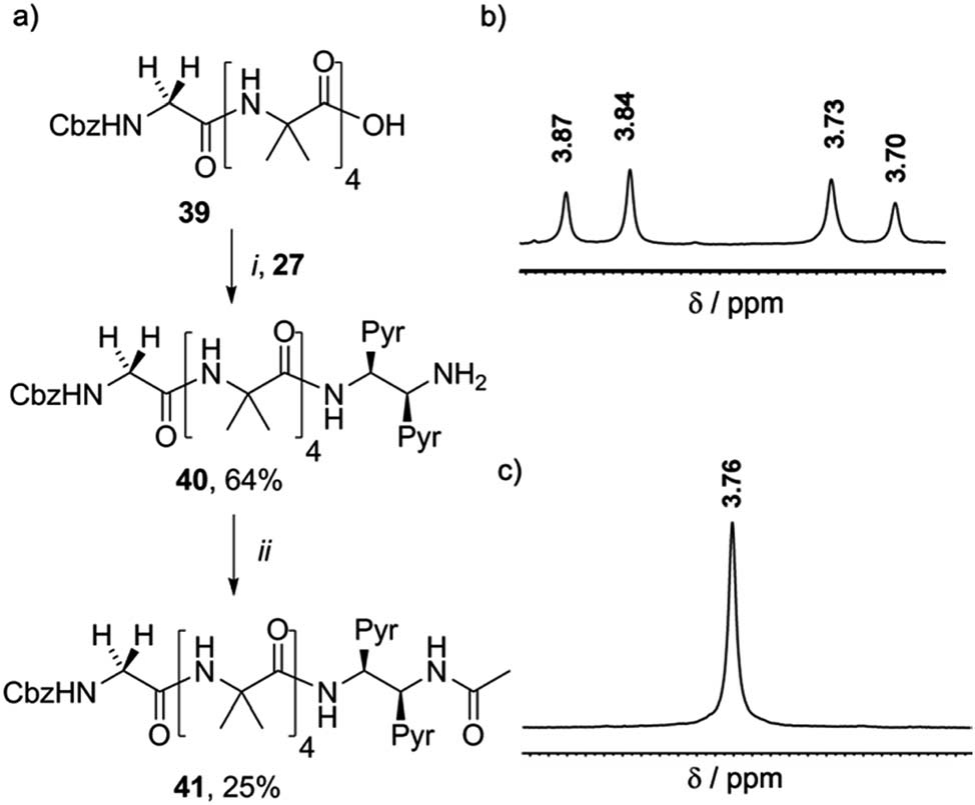
(a) Synthesis of **40** and **41**. (i), EDC/HOBt, i-Pr_2_NEt, CH_2_Cl_2_ (ii), Ac_2_O, CH_2_Cl_2_. (b) Appearance of the ^1^H NMR resonances in CD_3_OD of the Gly CH_2_ of (b) **40** and (c) **41**.

It was expected that the chiral acetylated reporter (*S*,*S*-BisPyrEt)NHAc may itself induce some degree of screw-sense control, but the extent of this conformational influence in solution was unknown. Foldamers **40** and **41** (Scheme 5a) were thus used to measure the screw-sense preference exerted by (*S*,*S*-BisPyrEt)NH_2_ and (*S*,*S*-BisPyrEt)NHAc. The N-terminal CbzGly residue in these compounds acts an NMR reporter of induced screw-sense preference; the fast-exchange chemical shift separation of the AB system arising from the diastereotopic methylene protons of the Gly residue is proportional to the screw-sense preference (defined by the helical excess, *h.e*. = ([*P*-foldamer] – [*M*-foldamer])/[total foldamer]) induced in the helical chain by the chiral C-terminal probe.1*d*

Coupling **27** to CbzGlyAib_4_OH **39** gave CbzGlyAib_4_(*S*,*S*-BisPyrEt)NH_2_**40**, which was acetylated to give GlyAib_4_(*S*,*S*-BisPyrEt)NHAc **41**. The glycine residue of amine-terminated foldamer **40** appears as an AB system with significant anisochronicity (Δ*δ* = 130 ppb), indicating powerful induction of a screw-sense preference by the fluorescent reporter (Scheme 5b): this amino-terminated bis(pyrene) motif in **40** thus behaves as a controller rather than a reporter. In contrast, the CH_2_ group of the glycine residue in acetamide **41** appears as a 2H singlet with no detectable anisochronicity, confirming that this reporter exerts very little control over the screw-sense preference of the Aib helix (Scheme 5c). Whether or not acetylation increases the strength of the C-terminal hydrogen bond, it certainly weakens the ability of the chiral ethylenediamine moiety to control helical screw-sense, while still retaining its ability to respond to its environment: in other words, acetylation successfully transforms the amino-terminated bis(pyrene) motif from a controller into a reporter.

### Structural characterization of C-terminal acetylated 1,2-diamine-based probes

**Scheme 6:**
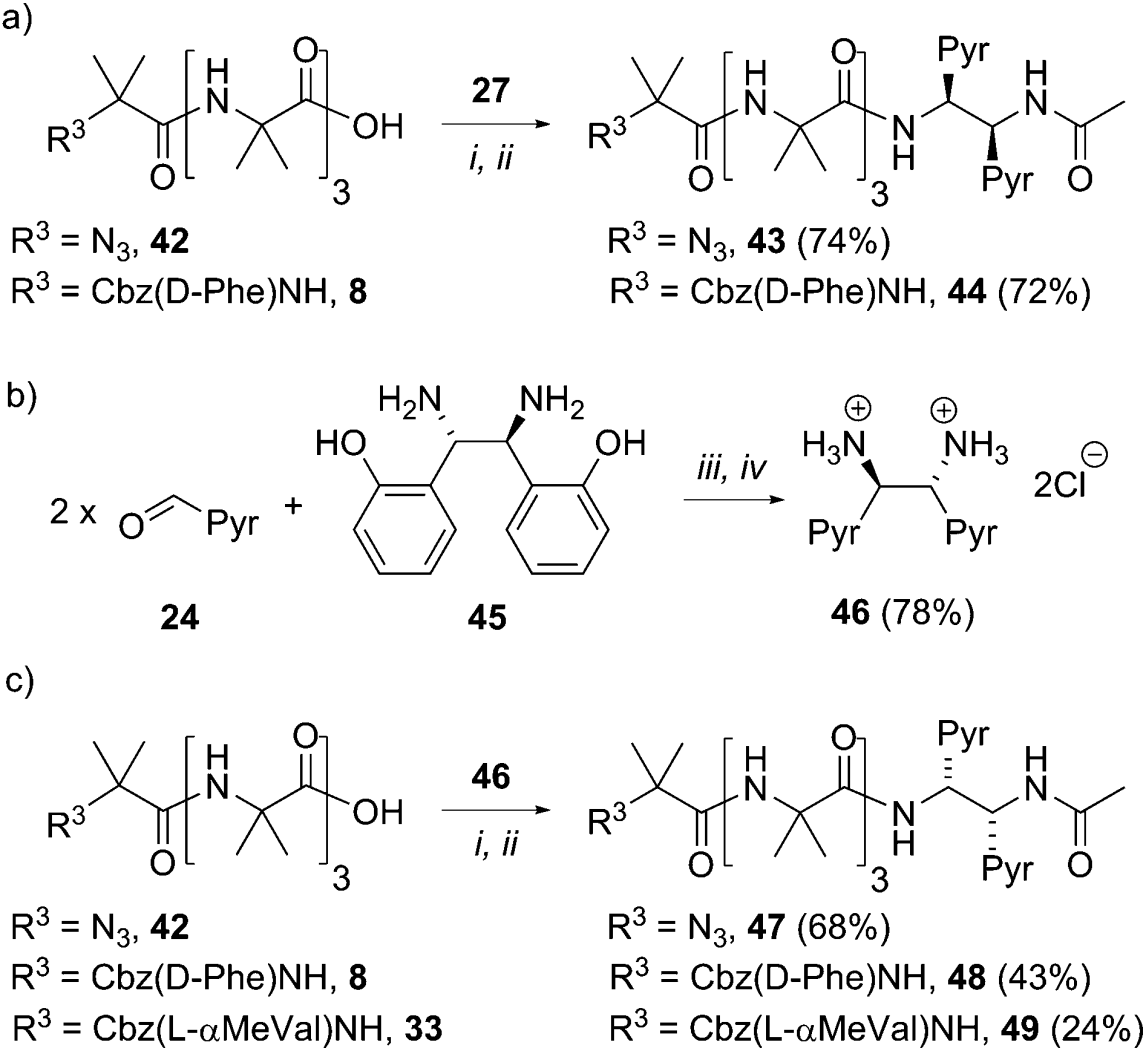
Synthesis of uncontrolled foldamers **43** and **47**, d-Phe controlled foldamers **44** and **48**, and l-α-MeVal controlled foldamer **49**. (a, c) (i), EDC/HOBt, i-Pr_2_NEt, CH_2_Cl_2_; (ii), Ac_2_O, CH_2_Cl_2_. (b) (iii), DMSO, 3 h, then H_2_O. (iv) THF, conc. HCl.

To gain more insight into the molecular structure that links the conformation of the reporter group with the helical sense of the foldamer, five new foldamers terminated with acetylated 1,2-diamine-based probes were synthesized. These compounds included those designed to provide enantiomeric pairs of selected foldamers. They included compounds with no chiral controller at the N-terminus (**43** and **47**), as well as those with quaternary amino acids at the N-terminus (**49**, the enantiomer of **38**, Scheme 6).

To create enantiomers of **38** and **43**, the *R*,*R* stereoisomer of the (BisPyrEt)NHAc probe was synthesized. It was straightforwardly available from (1*S*,2*S*)-1,2-bis(2′-hydroxyphenyl)ethylenediamine **45**, the commercially available enantiomer of **26**. The resulting *R*,*R*-bis(pyrene) probe **46** was then coupled both to uncontrolled Aib tetramer **42**, Cbz(d-Phe) controlled Aib tetramer **8**, and Cbz(l-αMeVal) controlled Aib tetramer **33**. The resulting foldamers **47–49** each bear the (*R*,*R*-BisPyrEt)NHAc reporter.

Of these five compounds, foldamers **43**, **48** and **49** were able to be crystallized and their solid state structures were determined by X-ray crystallography.

**Fig. 5:**
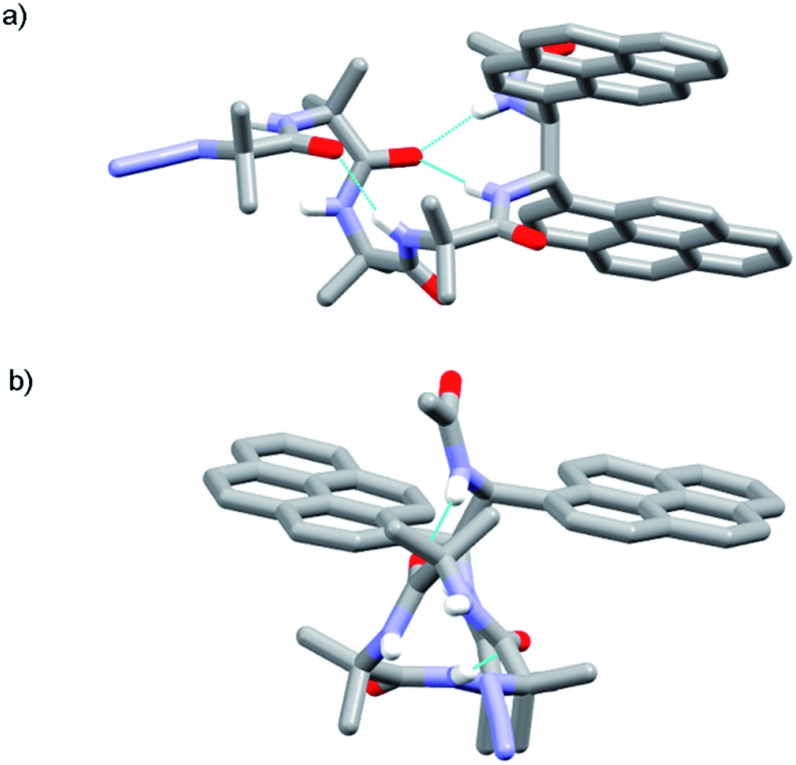
X-ray crystal structure of **43** (a) viewed side-on, showing *M* helical structure with bifurcated NH bond between the carbonyl of Aib-2 and the amide NHs of the probe; (b) viewed along the helix axis from N- to C-terminus, revealing the large separation of the pyrene groups.

The X-ray crystal structure of control compound N_3_Aib_4_(*S*,*S*-BisPyrEt)NHAc **43**, which lacks a chiral N-terminal controller, suggests one reason why the acetylated reporter group is so sensitive to changes in helical conformation. Foldamer **43** (Fig. 5) adopts a left-handed *M* helical conformation, necessarily as a result of the chiral influence of the (*S*,*S*-BisPyrEt)NHAc probe. There is a bifurcated hydrogen bond between the carbonyl of the second Aib residue and both amide NHs of the ethylenediamine probe, coupling the torsion angle of the central C–C bond in the fluorescent probe (PyrC–CPyr torsion angle –169°) with the position of this carbonyl within the helical structure. The large separation of the pyrene groups, with an antiperiplanar relationship between N–C(pyren-1-yl) bonds, results from significant steric interactions within the reporter group. The wide separation of the pyrene groups implies **43** should show a low *E*/*M* ratio. However the net *E*/*M* in solution (2.50 in MeOH) also includes excimer contributions from the *P* screw-sense conformation (which does not appear in the solid state structure), in which the pyrene groups may lie much closer together.

The structure of **43** in the solid state shows that the (*S*,*S*-BisPyrEt)NHAc reporter will favor a *M* helical conformation and the enantiomeric (*R*,*R*-BisPyrEt)NHAc reporter will favor a *P* helical conformation, although studies on **41** in solution show that the inductive strength of the reporter is low. A chiral influence from the N-terminus could enhance or counteract the conformational preference of the reporter. For example, both Cbz(l-αMeVal) and Cbz(d-Phe) residues favor adoption of a *P* 3_10_-helix through the formation of either an N-terminal type III or a type II′ β-turn respectively.^5^,^21^ Therefore, if combined with the (*R*,*R*-BisPyrEt)NHAc reporter, both the N- and C-terminal screw-sense preferences (for a *P* 3_10_-helix) would be “matched”.

**Fig. 6:**
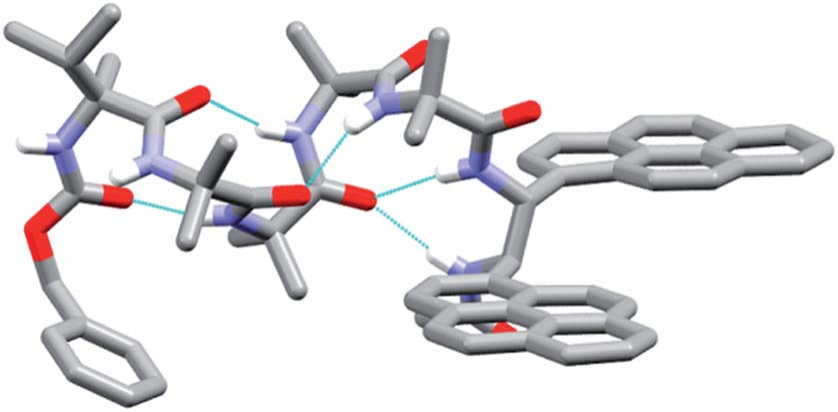
X-ray crystal structure for **49**. Viewed side-on, showing *P* helical structure with bifurcated NH bond between the carbonyl of Aib-2 and amide NHs of the probe.

The X-ray crystal structure of **49** (the enantiomer of **38**) shows “matching” of screw-sense preferences. The foldamer adopts a 3_10_-helix with a *P* screw-sense (Fig. 6), which is the screw-sense favored both by Cbz(l-αMeVal) at the N-terminus, and (given the structure of **43**) by the (*R*,*R*-BisPyrEt)NHAc reporter. There are only *P* helices in the unit cell of **49**, and each foldamer adopts a type III β-turn at the N-terminus with a bifurcated hydrogen bond to the two amide NHs of the ethylenediamine at the C-terminus (as observed in the structure of **43**). The good helical control exerted by αMeVal and large separation of the antiperiplanar pyrene groups (PyrC–CPyr torsion angle +170°) is consistent with the low excimer emission for both **49** and **38** in organic solvents.

**Fig. 7:**
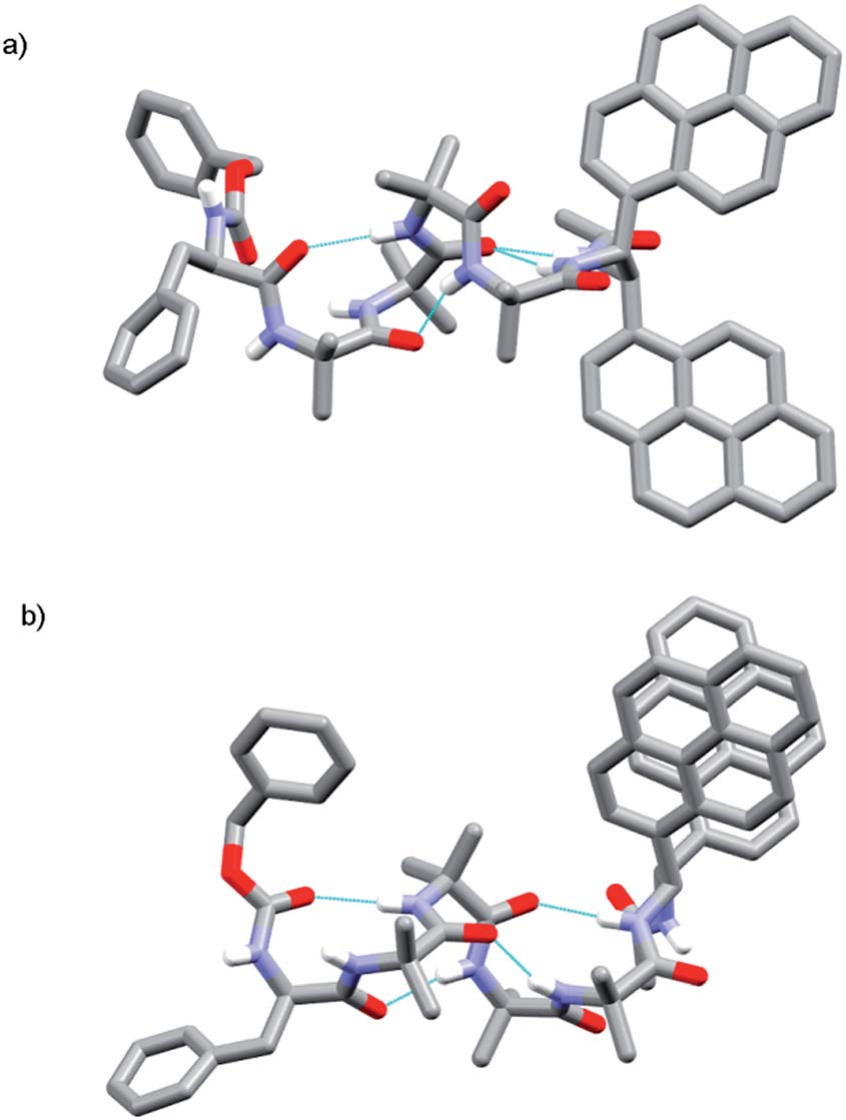
X-ray crystal structure for **48**, showing the two conformers observed in the unit cell. (a) Top view of *P* helical conformer (b) top view of *M* helical conformer.

Foldamer **48** also has “matched” screw-sense preferences, with the d-Phe controller expected to induce a *P* screw-sense. However the X-ray crystal structure of **48** is a remarkable example of where both *P* and *M* screw-senses occur in the unit cell (Fig. 7). Indeed previous studies have shown that Aib tetramers containing N-terminal tertiary amino acids display less uniform structures in the solid state, and can in some case even adopt the opposite helical sense in the solid state compared to that found in solution.^5^ The conformer with the *P* screw-sense has a structure around the bis(pyrene) probe that is analogous to that observed for **43** and **49**, with spatially well-separated pyrene groups (PyrC–CPyr torsion angle +167°) and a bifurcated hydrogen bond to the amides of the C-terminal reporter. At the N-terminus, the chiral d-Phe residue induces a type II′ β-turn, similar to that observed for other Aib foldamers capped by tertiary amino acids.^5^,^21^

In contrast, the conformer with the *M* screw-sense has the pyrene groups of the reporter lying on top of one another in a “V”-shaped geometry, suggesting that *M* 3_10_-helices terminated with (*R*,*R*-BisPyrEt)NHAc or *P* 3_10_-helices terminated with (*S*,*S*-BisPyrEt)NHAc would show strong excimer emission. This change in the torsion angle in the reporter to +69° is accompanied by the loss of the hydrogen bond from the carbonyl of the second Aib to the NH of the C-terminal acetamide. At the N-terminus, the chiral d-Phe residue now adopts a type III β-turn, similar to that induced by the l-αMeVal in **49** but with the opposite sense.

### Applications of 1,2-diamine-based probes: (*S*,*S*-BisPyrEt)NHAc as a reporter of conformation in organic solvents

**Scheme 7:**
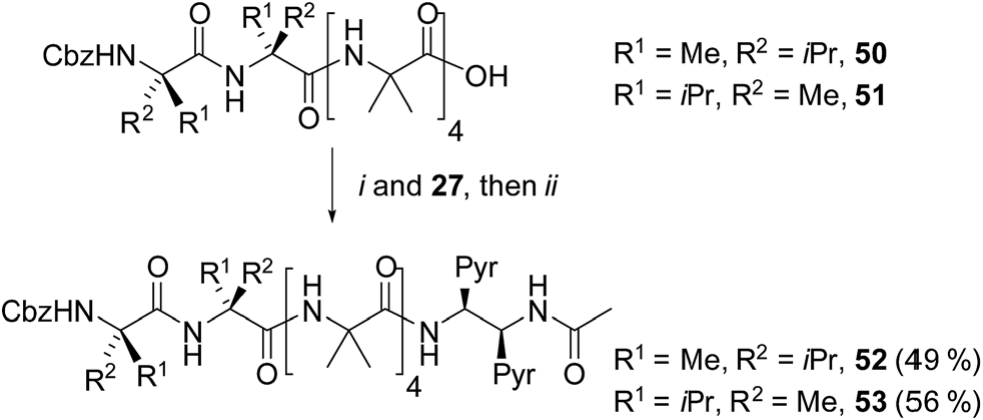
Synthesis of (αMeVal)_2_ controlled foldamers **52** and **53**. (i), EDC/HOBt, i-Pr_2_NEt, CH_2_Cl_2_; (ii), Ac_2_O, CH_2_Cl_2_.

Our next task was to correlate the *E*/*M* response of one of these optimized bis(pyrene) probes, specifically (*S*,*S*-BisPyrEt)NHAc, with the quantified screw-sense preference of the adjacent helix. Two further foldamers **52** and **53** with almost quantitative screw-sense preference were synthesized (Scheme 7). N-terminal Cbz(αMeVal)_2_ controllers induce very high levels of control over the screw-sense of Aib foldamers (almost 100%, especially in non-polar solvents).1c,^5^ Diamine **27** was ligated to **50** and **51**, then acetylated to give *P* helical **52** and *M* helical **53**.

The screw-sense preference of these controllers ranges from ∼100% *M* to ∼100% *P*, therefore covering the entire *E*/*M* response range for the acetamide probe (Table 1). These data show that, as expected from the structures of **43**, **48** and **49** (as well as the structure of **53**, see the ESI†) increasing *M* screw-sense reduces excimer fluorescence from the (*S*,*S*-BisPyrEt)NHAc reporter, consistent with an increase in the population of conformers with widely separated pyrene groups. The response is stronger in methanol than acetonitrile, although the trends are similar (see the ESI†). These data could be fitted to a simple model that assumes only two conformational populations, namely *M* and *P* 3_10_-helices (see the ESI†). This model gave good agreement with the experimental values and gave calculated *E*/*M* values of 1.05 for the *M* helical conformer, 5.50 for the *P* helical conformer and 2.50 for the racemic mixture.

**Table 1:** *E*/*M* values and reported induced screw-sense preferences for foldamers **37**, **38**, **41**, **43**, **44**, **52** and **53**

Compound, [controller]	Δ*δ*^*a*^	*h.e*.^*b*^	*E*/*M*^*c*^
**53**, [Cbz(d-αMeVal)_2_]	383	–95	1.12
**38**, [Cbz(d-αMeVal)]	275	–68	1.38
**41**, [Cbz(Gly)]	0	0	2.47
**43**, [N_3_]	0	0	2.50
**44**, [Cbz(d-Phe)]	209	+52	3.32
**37**, [Cbz(l-αMeVal)]	275	+68	3.81
**52**, [Cbz(l-αMeVal)_2_]	383	+95	5.28

^*a*^Anisochronicity (Δ*δ*) induced by each controller in Aib_4_ foldamers, determined using a C-terminal glycinamide reporter.^5^

^*b*^Corresponding helical excess (*h.e*.).

^*c*^ *E*/*M* ratio measured in MeOH at 10 μM using the (*S*,*S*-BisPyrEt)NHAc reporter; monomer emission at 377 nm, excimer is the maximum emission intensity from 410 to 600 nm.

### Applications of 1,2-diamine-based probes: (*S*,*S*-BisPyrEt)NHAc as a reporter of conformation in the membrane phase

Isotropic organic solvent is often used as a model system for phospholipid bilayers, with the water/bilayer interface modelled by methanol/water mixtures and the center of the bilayer modeled with low polarity solvents such as chloroform.^25^ The development of hydrophobic foldamers bearing bis(pyrene) reporter groups offers a valuable opportunity to ascertain the extent to which organic solvent is a useful model for bilayers and to uncover how the membrane environment may exert a distinctive influence on the conformation of embedded compounds, like peptides and foldamers.

**Fig. 8:**
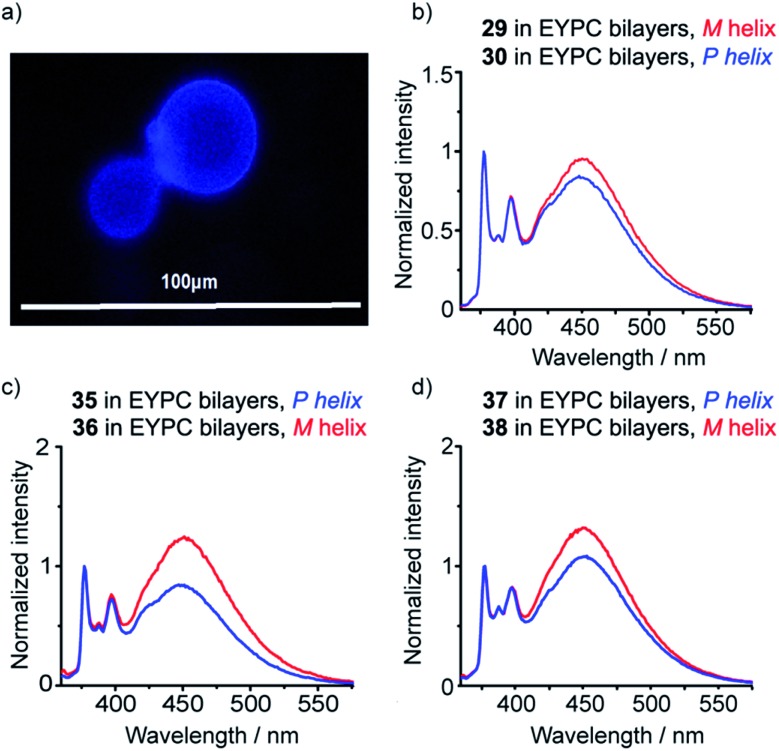
(a) Epi-fluorescence microscopy image of EYPC GUVs with **37** embedded in the bilayers at 1 mol%; (b–d) normalized fluorescence emission spectra for **29**, **30**, **35–38** (10 μM, in EYPC LUVs, 800 nm diameter at 1 mol%, emission normalized to 377 nm).

To verify that these foldamers would indeed successfully embed in vesicle membranes, giant unilamellar vesicles (GUVs) were prepared by electroformation from egg yolk phosphatidylcholine (EYPC) mixed with either **37** or **38** (1 mol%). Fluorescence microscopy images showed bright excimer fluorescence from the membranes of the GUVs, with no visible fluorescence from any foldamer that might not have been incorporated into the bilayer (Fig. 8a). Furthermore, the uniform excimer fluorescence of **37** and **38** suggested the foldamers are evenly dispersed across the vesicle membrane, without visible microdomain formation; microdomains that are enriched in foldamer may produce intermolecular excimers.^26^ To confirm that intermolecular excimer emission is low, a membrane dilution experiment was performed. Foldamer **43** was embedded in the membranes of EYPC large unilamellar vesicles (LUVs, 800 nm diameter) at loadings of 0.2, 0.4, 0.6 and 1 mol%. Any non-embedded foldamer was removed by gel permeation chromatography (GPC) of the vesicular suspensions. The fluorescence spectra of each LUV suspension showed similar *E*/*M* ratios (*E*/*M* values of 1.07, 1.02, 1.01 and 1.10 respectively), with no increase in *E*/*M* as the loading increased (see the ESI†). This mirrors observations for other Aib foldamers in EYPC LUVs,^12^ and suggests that the excimer emission observed is predominantly intramolecular in origin.

To measure how controllers of opposite chirality affect the conformation of foldamers in the membrane phase, (*S*,*S*-BisPyrEt)NH_2_-terminated foldamers **29**, **30**, **35** and **36** and (*S*,*S*-BisPyrEt)NHAc-terminated foldamers **37** and **38** (Schemes 3 and 4) were embedded in the membranes of EYPC LUVs at 1 mol%.

The fluorescence spectra of these vesicular suspensions are shown in Fig. 8b–d. As in organic solvent, the diastereoisomeric pairs of compounds showed distinguishably different fluorescence spectra, indicating that the fluorescent reporter was sensitive to conformational environment even in the phospholipid membrane phase. Some differences were however apparent on comparison with their emission spectra in organic solvents. There was a significant reduction in *E*/*M* values for all foldamers. All values were closer to 1 and the largest observed *E*/*M* in bilayers was 1.47, measured for the (*S*,*S*-BisPyrEt)NH_2_-terminated foldamer with Cbz(d-αMeVal) controller (**36**). These data suggest that conformations with the pyrene groups in close proximity became less favorable. In addition, intriguingly, the helical screw-sense that showed the higher *E*/*M* ratio was now reversed, with *M* helices resulting in greater excimer emission. This change is ascribed to perturbation of the response from the reporter rather than a change in the screw-sense of the foldamer.^27^ The sensitivity ratio for foldamers **29**, **30**, **35–38** in bilayers was therefore below 1. Inversion of the *S*_R_ value for (*S*,*S*-BisPyrEt)NHAc-terminated foldamers **37** and **38** in bilayers (1/*S*_R_ = 1.3) allows meaningful comparison to the *S*_R_ values for **37** and **38** in organic solution (*S*_R_ = 2.7) and reveals a reduced sensitivity to conformational change when in bilayers. However the (*S*,*S*-BisPyrEt)NH_2_-terminated foldamers **35** and **36** did not show a decrease in sensitivity when in bilayers (1/*S*_R_ = 1.5 in bilayers, *S*_R_ = 1.4 in solution).

Although the (*S*,*S*-BisPyrEt)NH_2_ reporter gave a slightly better sensitivity ratio in bilayers compared to the (*S*,*S*-BisPyrEt)NHAc reporter, the latter was more versatile. Foldamers bearing this reporter (such as **37** and **38**) were found to degrade more slowly upon storage at 4 °C than the amine-terminated reporters. Additionally the amino-terminus of the (*S*,*S*-BisPyrEt)NH_2_ reporter could become protonated in buffered solution (pH 7.4) and thereby produce unwanted intermolecular interactions in biological environments. The combination of good membrane insertion, stability and the clear dependence of fluorescence emission on the induced local conformational environment shows that (*S*,*S*-BisPyrEt)NHAc reporters are useful tools for the study of conformational change in membrane-embedded peptides.

The observed differences in the *E*/*M* values between diastereoisomeric foldamers in vesicles show that the conformational relay in the foldamers remains intact in bilayers. Additionally, these synthetic compounds, with only a few chiral centers, can be used to gain new insights into intermolecular interactions with the matrix lipids of the membrane. In particular, comparing *E*/*M* values from enantiomeric foldamers could reveal the often overlooked influence of phospholipid chirality on the conformation of embedded peptides and proteins.

### Application of 1,2-diamine-based probes to assess chiral induction by phospholipids in the membrane phase

Naturally occurring phospholipids, such as the phosphatidylcholines found in EYPC, have an *R* configuration at the central carbon of the glycerol backbone. Although the effect of phospholipid chirality on enantioselective processes in bilayers has been sparsely investigated, phospholipid bilayers have been shown to exhibit differential recognition of enantiomers of amino acids,^28^ dipeptides^29^ and ibuprofen.^30^ The effect of a bilayer on enantioselective reactions at its surface is less clear.^31^,^32^

Reports by Nakagawa and co-workers indicate that phospholipid chirality can influence the conformational equilibria of embedded molecules; helicenes that rapidly interconvert between *M* and *P* helical conformations were found to deracemize in phospholipid bilayers.^33^,^34^ Although there are no reports of the deracemization of achiral peptides in phospholipid bilayers, micelles composed of *N*-dodecylproline were used to induce a *h.e*. of at least 33% in an achiral 3_10_-helical Aib octamer.^35^ Recent vibrational circular dichroism studies of enantiomeric Aib foldamers embedded in phospholipid bilayers hinted that phospholipid chirality may produce diastereoisomeric conformations.^27^ Therefore the availability of two pairs of enantiomeric foldamers bearing bis(pyrene) reporters (**38** and **49**; **43** and **47**) offers a unique opportunity to observe chiral induction due to the phospholipids that constitute the bilayer.

**Fig. 9:**
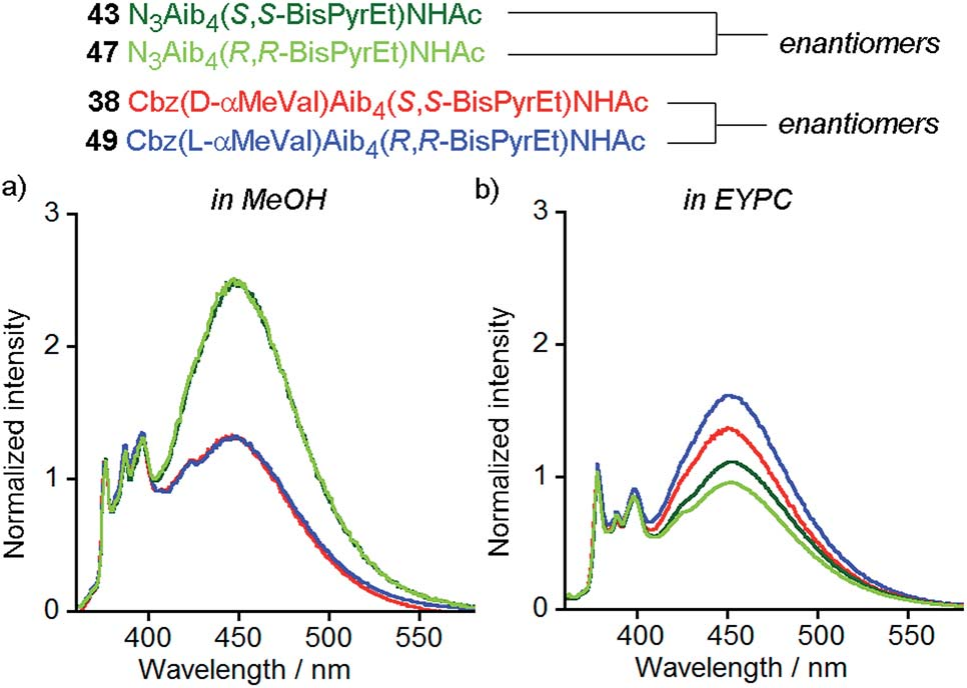
Normalized fluorescence emission spectra (emission normalized to 1 at 377 nm) for **43** (dark green line), **47** (pale green line), **38** (red line) and **49** (blue line) in (a) MeOH (10 μM); (b) EYPC LUVs, 800 nm diameter at 1 mol% (10 μM).

The enantiomers of each pair gave the same fluorescence emission spectra in isotropic organic solvent (methanol), with higher excimer emission from the uncontrolled helices (Fig. 9a). However, at 1 mol% in EYPC vesicles, significant differences in the ratiometric emission from enantiomeric foldamers **43** (*E*/*M* = 1.11 ± 0.02) and **47** (*E*/*M* = 0.96 ± 0.02) were observed across either four or three pairs (respectively) of separately prepared samples. Similarly, a difference was observed between enantiomers **38** (*E*/*M* = 1.37) and **49** (*E*/*M* = 1.62) (Fig. 9b).

The observation of a small but significant difference between the *E*/*M* ratios for **43** and **47**, with the *S*,*S* enantiomer higher than the *R*,*R* shows the chirality of the bilayer can perturb the response of the (BisPyrEt)NHAc reporter. Furthermore, the different *E*/*M* values for **38** and **49** show bilayer chirality also affects the degree of induction from the controller, since for this pair the *R*,*R* reporter gives a higher *E*/*M* value than the *S*,*S* reporter. The difference in *E*/*M* between enantiomers **38** and **49** in a bilayer (Δ(*E*/*M*) = +0.25) is comparable to the difference in *E*/*M* between diastereoisomers **38** and **37** in a bilayer (Δ(*E*/*M*) = –0.29), indicating chiral induction by the bilayer can be significant influence on conformation.

## Conclusions

The synthesis and analysis of several classes of bis(pyren-1-yl) fluorescent probe has shown that 1,2-bis(pyren-1′-yl)ethylenediamines, readily made by an enantiospecific aza-Cope rearrangement, display promising features as probes of conformational change in oligomers. Ligation to the C-terminus of Aib foldamers was followed by acetylation of the free amino group, providing a versatile reporter of peptide conformation.

The acetamide of (*S*,*S*)-1,2-bis(pyren-1′-yl)ethylenediamine succeeded as a fluorescent reporter of conformation where earlier generations of probes did not for several key reasons. Unlike C-terminal pyrenylalanine residues, the chirality in these bis(pyren-1′-yl)ethylenediamine reporters did not overwhelm the remote chiral influence provide by the N-terminal residue. The closer proximity of the two pyrene units gave substantially greater excimer fluorescence than designs that had the pyrene units spaced further apart. Acetylation of the C-terminal amine reduced the control exerted by the bis(pyren-1′-yl)ethylenediamine on the Aib foldamer, providing an optimized reporter that is more responsive to differences in screw-sense preference.

The optimized bis(pyrene) fluorescent reporter permits conformational interchange between *M* and *P* helicities in Aib foldamers to be monitored in real time in both organic solvents and bilayers. In bilayers, excimer fluorescence from the reporter group allowed the localization of foldamers in vesicle membranes to be confirmed by fluorescence microscopy. The synthesis of enantiomers of chiral peptide foldamers bearing this bis(pyrene) fluorescent reporter was used to provide a unique biophysical insight, revealing for the first time that the chirality of phospholipids in a bilayer can have a significant influence on the conformation of oligomers, such as foldamers and peptides, in the membrane.

The design of this probe permits rapid and continuous measurement of conformational change, a distinct advantage over previous probes that required monitoring by solid state ^19^F NMR spectroscopy. These advantages have been realized in the development of a functioning mimic of a G protein coupled receptor, which was able to undergo conformational change in a bilayer upon binding of a chemical signaling ligand.^12^ Using fluorescence to measure conformational change is also more amenable to studies of foldamers in cellular membranes, where cellular location and conformational switching can both be measured. The probe structure described in this work is a promising lead compound for such studies in cells.

## Conflicts of interest

There are no conflicts to declare.

## Supplementary Material

Supplementary information

Crystal structure data
