# Supplementary material for: Bis-pyrene probes of foldamer conformation in solution and in phospholipid bilayers
Source: Chem Sci. 2018 Jul 17;9(33):6860–70. doi: 10.1039/c8sc02532k (PMC6114994; doi:10.1039/c8sc02532k)
Supplement: Supplementary file 1 [file SC-009-C8SC02532K-s001.pdf]

Electronic Supplementary Material (ESI) for Chemical Science.
This journal is © The Royal Society of Chemistry 2018

Electronic Supplementary Information

Bis-pyrene probes of foldamer conformation in solution and in phospholipid bilayers

Francis G. A. Lister,[1] Natasha Eccles,[1,2] Sarah J. Pike,[1,2,3] Robert A. Brown,[1] George F. S. Whitehead,[1] James Raftery,[1] Simon J. Webb*,[1,2] and Jonathan Clayden*[4]

1. School of Chemistry, University of Manchester, Oxford Road, Manchester M13 9PL, U.K.
2. Manchester Institute of Biotechnology, University of Manchester, 131 Princess St, Manchester M1 7DN, U.K.
3. Faculty of Life Sciences, University of Bradford, Bradford, West Yorkshire, BD7 1DP, U.K.
4. School of Chemistry, University of Bristol, Cantock's Close, Bristol BS8 1TS, UK.

*. Email: S.Webb@manchester.ac.uk, j.clayden@bristol.ac.uk

Table of Contents

1. Chemical synthesis: Instruments **7**

2. Materials and General Experimental Procedures for Synthesis **8**

2.1 Materials *8*

2.2. General experimental procedures *8*

General procedure 1: Coupling of pyrene probe to the C terminus of peptides 9

General procedure 2: Sequential deprotection of Boc-pyrene probe and coupling to peptide C-terminus 9

General procedure 3: Acetylation of C terminal amines 10

General procedure 4: Sequential coupling and acetylation of pyrene probe at peptide C terminus 10

General procedure 5: Coupling of C-deprotected peptides with azide *via* Staudinger Vilarrasa ligation 10

2.3. Abbreviations *11*

3. Synthetic Procedures **12**

Synthesis of Boc(L-Pya)(D-Pya)OMe **4** 12

Synthesis of Boc(L-Pya)(L-Pya)OMe **5** 13

Synthesis of Boc(L-Pya)NHCH$_2$Pyr **6** 14

Synthesis of Cbz(L-Phe)Aib$_4$(L-Pya)(L-Pya)OMe **9** 15

Synthesis of Cbz(D-Phe)Aib$_4$(L-Pya)(L-Pya)OMe **10** 16

Synthesis of Cbz(L-Phe)Aib$_4$(L-Pya)(D-Pya)OMe **11** 17

Synthesis of Cbz(D-Phe)Aib$_4$(L-Pya)(D-Pya)OMe **12** 18

Synthesis of Cbz(L-Phe)Aib$_4$(L-Pya)NHCH$_2$Pyr **13** 19

Synthesis of Cbz(D-Phe)Aib$_4$(L-Pya)NHCH$_2$Pyr **14** 20

Synthesis of (2*S*,3*R*)-2-azido-3-hydroxysuccinic acid **17** 21

Synthesis of (2*S*,3*R*)-bis(pyren-1-ylmethyl) 2-azido-3-hydroxysuccinate **18** (N$_3$(2*S*,3*R*-BisPyrSucc)OH) 22

Synthesis of (2*R*,3*R*)-bis(pyren-1-ylmethyl) 2-azido-3-hydroxysuccinate **19** (N$_3$(2*R*,3*R*-BisPyrSucc)OH) 23

Synthesis of Cbz-L-PheAib$_4$(2*S*,3*R*-BisPyrSucc)OH **20** 24
Synthesis of Cbz-D-PheAib$_4$(2*S*,3*R*-BisPyrSucc)OH **21** 25
Synthesis of Cbz-L-PheAib$_4$(2*R*,3*R*-BisPyrSucc)OH **22** 26
Synthesis of Cbz-D-PheAib$_4$(2*R*,3*R*-BisPyrSucc)OH **23** 27
Synthesis of *p*-(1-pyrenylether) benzaldehyde **25** 28
Synthesis of (1*S*,2*S*)-1,2-bis(4-(pyren-1-ylmethoxy)phenyl)ethane-1,2-diamine.2HCl **28** (*S*,*S*-BisPhenPyrEt) 29
Synthesis of Cbz(L-Phe)Aib$_4$(*S*,*S*-BisPyrEt)NH$_2$ **29** 30
Synthesis of Cbz(D-Phe)Aib$_4$(*S*,*S*-BisPyrEt)NH$_2$ **30** 31
Synthesis of Cbz(L-Phe)Aib$_4$(*S*,*S*-BisPhenPyrEt)NH$_2$ **31** 32
Synthesis of Cbz(D-Phe)Aib$_4$(*S*,*S*-BisPhenPyrEt)NH$_2$ **32** 33
Synthesis of N$_3$Aib$_4$(*S*,*S*-BisPyrEt)NHAc **43** 34
Synthesis of Cbz(D-Phe)Aib$_4$(*S*,*S*-BisPyrEt)NHAc **44** 35
Synthesis of (1*R*,2*R*)-1,2-(1-pyrene)ethylenediamine dihydrochloride **46** 36
Synthesis of N$_3$Aib$_4$(*R*,*R*-BisPyrEt)NHAc **47** 37
Synthesis of Cbz-D-PheAib$_4$(*R*,*R*-BisPyrEt)NHAc **48** 38
Synthesis of Cbz(L-αMeVal)Aib$_4$(*R*,*R*-BisPyrEt)NHAc **49** 39

4. Spectroscopic studies of foldamers in organic solvents and vesicles 40

4.1 General proceduresfor the preparation of phospholipid vesicles 40
4.1.1. Preparation of 800 nm diameter egg yolk phosphatidylcholine (EYPC) large unilamellar vesicles (LUVs) containing foldamer. 40
4.1.2. Preparation of egg yolk phosphatidylcholine (EYPC) giant unilamellar vesicles (GUVs) containing foldamers **37** and **38**. 41

4.2. Fluorescence spectroscopy and spectrophotometry experimental details 41
4.2.1. Spectroscopic solvents 41
4.2.2. Spectroscopy and microscopy: Instruments. 41
4.2.3. Preparation of stock solutions of **9-14**, **20-23**, **29**, **30**, **35-38**, **41**, **43**, **44**, **52** and **53**. 42
4.2.4. Fluorescence spectra for **20-23** 43
4.2.5. Fluorescence spectra for **9-14** 44
4.2.6. CD spectra for **9-14** 45
4.2.7. Fluorescence and CD spectra for **29** and **30** 46

4.2.8. Fluorescence spectra for **29** and **30** at different concentrations 47
4.2.9. Fluorescence spectra for **35** and **36** at different concentrations 48
4.2.10. Fluorescence spectra for **37** and **38** at different concentrations.....49
4.2.11. Sensitivity ratios (S_R) for **35**, **36**, **37** and **38** at different concentrations 50
4.2.12. Sensitivity ratios (S_R) for **35**, **36**, **37** and **38** in EYPC vesicles.....50
4.2.13 E/M values and helical excess (*h.e.*) for foldamers **37**, **38**, **41**, **43**, **44**, **52** and **53** in MeOH and CH_3CN.....51
4.2.14. Fitting of E/M values for foldamers in MeOH as a function of helical excess.....52
4.2.15. Fluorescence microscopy images of **37** and **38** in membranes of EYPC GUVs. 53
4.2.16. E/M values from **43** in EYPC LUVs at loadings of 0.2, 0.4, 0.6 and 1.0 mol%.....54

5. NMR spectra of novel compounds 55

^{1}H NMR spectrum of Boc(L-Pya)(D-Pya)OMe **4** 55
13C NMR spectrum of Boc(L-Pya)(D-Pya)OMe **4** 55
^{1}H NMR spectrum of Boc(L-Pya)(L-Pya)OMe **5**.....56
^{13}C NMR spectrum of Boc(L-Pya)(L-Pya)OMe **5**.....56
^{1}H NMR spectrum of Boc(L-Pya)NHCH_2Pyr **6** 57
^{13}C NMR spectrum of Boc(L-Pya)NHCH_2Pyr **6**.....57
^{1}H NMR spectrum of Cbz(D-Phe)Aib_4(L-Pya)(L-Pya)OMe **9** 58
^{13}C NMR spectrum of Cbz(D-Phe)Aib_4(L-Pya)(L-Pya)OMe **9** 58
^{1}H NMR spectrum of Cbz(L-Phe)Aib_4(L-Pya)(L-Pya)OMe **10** 59
^{13}C NMR spectrum of Cbz(L-Phe)Aib_4(L-Pya)(L-Pya)OMe **10**.....59
^{1}H NMR spectrum of Cbz(L-Phe)Aib_4(L-Pya)(D-Pya)OMe **11** 60
^{13}C NMR spectrum of Cbz(L-Phe)Aib_4(L-Pya)(D-Pya)OMe **11** 60
^{1}H NMR spectrum of Cbz(D-Phe)Aib_4(L-Pya)(D-Pya)OMe **12**.....61
^{13}C NMR spectrum of Cbz(D-Phe)Aib_4(L-Pya)(D-Pya)OMe **12**.....61
^{1}H NMR spectrum of Cbz(L-Phe)Aib_4(L-Pya)NHCH_2Pyr **13** 62
^{13}C NMR spectrum of Cbz(L-Phe)Aib_4(L-Pya)NHCH_2Pyr **13** 62
^{1}H NMR spectrum of Cbz(D-Phe)Aib_4(L-Pya)NHCH_2Pyr **14**.....63
^{13}C NMR spectrum of Cbz(D-Phe)Aib_4(L-Pya)NHCH_2Pyr **14** 63
^{1}H NMR spectrum of (2*S*,3*R*)-2-azido-3-hydroxysuccinic acid **17** 64
^{13}C NMR spectrum of (2*S*,3*R*)-2-azido-3-hydroxysuccinic acid **17** 64
^{1}H NMR spectrum of (2S,3R)-bis(pyren-1-ylmethyl) 2-azido-3-hydroxysuccinate **18**.....65
^{13}C NMR spectrum of (2S,3R)-bis(pyren-1-ylmethyl) 2-azido-3-hydroxysuccinate **18**.....65

^{1}H NMR spectrum of (2R,3R)-bis(pyren-1-ylmethyl) 2-azido-3-hydroxysuccinate **19**......66
^{13}C NMR spectrum of (2R,3R)-bis(pyren-1-ylmethyl) 2-azido-3-hydroxysuccinate **19**....66
^{1}H NMR spectrum of Cbz(L-Phe)Aib$_4$(2*S*,3*R*-BisPyrSucc)OH **20**....67
^{13}C NMR spectrum of Cbz(L-Phe)Aib$_4$(2*S*,3*R*-BisPyrSucc)OH **20**....67
^{1}H NMR spectrum of Cbz(D-Phe)Aib$_4$(2*S*,3*R*-BisPyrSucc)OH **21**....68
^{13}C NMR spectrum of Cbz(D-Phe)Aib$_4$(2*S*,3*R*-BisPyrSucc)OH **21**....68
^{1}H NMR spectrum of Cbz(L-Phe)Aib$_4$(2*R*,3*R*-BisPyrSucc)OH **22**....69
^{13}C NMR spectrum of Cbz(L-Phe)Aib$_4$(2*R*,3*R*-BisPyrSucc)OH **22**....69
^{1}H NMR spectrum of Cbz(D-Phe)Aib$_4$(2*R*,3*R*-BisPyrSucc)OH **23**....70
^{13}C NMR spectrum of Cbz(D-Phe)Aib$_4$(2*R*,3*R*-BisPyrSucc)OH **23**....70
^{1}H NMR spectrum of *p*-(1-pyrenylether) benzaldehyde **25**....71
^{13}C NMR spectrum of *p*-(1-pyrenylether) benzaldehyde **25**....71
^{1}H NMR spectrum of (1*S*,2*S*)-1,2-bis(4-(pyren-1-ylmethoxy)phenyl)ethane-1,2-diamine dihydrochloride **28**....72
^{13}C NMR spectrum of (1*S*,2*S*)-1,2-bis(4-(pyren-1-ylmethoxy)phenyl)ethane-1,2-diamine dihydrochloride **28**....72
^{1}H NMR spectrum of Cbz(L-Phe)Aib$_4$(*S*,*S*-BisPyrEt)NH$_2$ **29**....73
^{13}C NMR spectrum of Cbz(L-Phe)Aib$_4$(*S*,*S*-BisPyrEt)NH$_2$ **29**....73
^{1}H NMR spectrum of Cbz(D-Phe)Aib$_4$(*S*,*S*-BisPyrEt)NH$_2$ **30**....74
^{13}C NMR spectrum of Cbz(D-Phe)Aib$_4$(*S*,*S*-BisPyrEt)NH$_2$ **30**....74
^{1}H NMR spectrum of Cbz(L-Phe)Aib$_4$(*S*,*S*-BisPhenPyrEt)NH$_2$ **31**....75
^{13}C NMR spectrum of Cbz(L-Phe)Aib$_4$(*S*,*S*-BisPhenPyrEt)NH$_2$ **31**....75
^{1}H NMR spectrum of Cbz(D-Phe)Aib$_4$(*S*,*S*-BisPhenPyrEt)NH$_2$ **32**....76
^{13}C NMR spectrum of Cbz(D-Phe)Aib$_4$(*S*,*S*-BisPhenPyrEt)NH$_2$ **32**....76
^{1}H NMR spectrum of N$_3$Aib$_4$(*S*,*S*-BisPyrEt)NHAc **43**....77
^{13}C NMR spectrum of N$_3$Aib$_4$(*S*,*S*-BisPyrEt)NHAc **43**....77
^{1}H NMR spectrum of Cbz(D-Phe)Aib$_4$(*S*,*S*-BisPyrEt)NHAc **44**....78
^{13}C NMR spectrum of Cbz(D-Phe)Aib$_4$(*S*,*S*-BisPyrEt)NHAc **44**....78
^{1}H NMR spectrum of (1*R*,2*R*)-1,2-(1-pyrene)ethylenediamine dihydrochloride **46**....79
^{13}C NMR spectrum of (1*R*,2*R*)-1,2-(1-pyrene)ethylenediamine dihydrochloride **46**....79
^{1}H NMR spectrum of N$_3$Aib$_4$(*R*,*R*-BisPyrEt)NHAc **47**....80
^{13}C NMR spectrum of N$_3$Aib$_4$(*R*,*R*-BisPyrEt)NHAc **47**....80
^{1}H NMR spectrum of Cbz(D-Phe)Aib$_4$(*R*,*R*-BisPyrEt)NHAc **48**....81

^{13}C NMR spectrum of Cbz(D-Phe)Aib_4(*R*,*R*-BisPyrEt)NHAc **48** 81
^{1}H NMR spectrum of Cbz(L-αMeVal)Aib_4(*R*,*R*-BisPyrEt)NHAc **49** 82
^{13}C NMR spectrum of Cbz(L-αMeVal)Aib_4(*R*,*R*-BisPyrEt)NHAc **49** 82

6. X-ray crystal structure data **83**

*6.1. Crystal data and refinement for **13*** *83*

*6.2. Crystal data and structure refinement for **43*** *86*

*6.3. Crystal data and structure refinement for **48*** *89*

*6.4. Crystal data and structure refinement for **49*** *93*

*6.5. Crystal data and structure refinement for **53*** *96*

7. References **100**

1. Chemical synthesis: Instruments

All ^1H and ^{13}C nuclear magnetic resonance (NMR) spectra were obtained using Bruker AVANCE 400, 500 or 600 MHz spectrometers. Chemical shifts are quoted in parts per million (ppm) and coupling constants (J) are quoted in Hz to the nearest 0.5 Hz. ^1H NMR were referenced to the residual deuterated solvent peak ($CDCl_3$ 7.27; CD_3OD 3.31; $(CD_3)_2SO$ 2.50 ppm) and ^{13}C NMR were referenced to the carbon resonance of the solvent ($CDCl_3$ 77.0; CD_3OD 49.05; $(CD_3)_2SO$ 39.52 ppm). Multiplicities are denoted as s (singlet), d (doublet), t (triplet), q (quartet), m (multiplet) or denoted as br. (broad), or some combination of these, where appropriate. Where ^1H NMR spectra were run in CD_3OD, D_2O exchangeable protons (NH, OH) are reported only where observed. Assignments were made using 2D ^1H-COSY and HMQC experiments. The anisochronicity, in parts per billion (ppb), of AB systems arising from the germinal ^1H nuclei of the $GlyNH_2$ diastereotopic NMR probe was given by $\upsilon_0\Delta\delta = [(f_1\text{-} f_3)^2 – J_{AB}^2]^{1/2} = [(f_2\text{-} f_4)^2\text{-}J_{AB}^2]^{1/2} = [(f_1\text{-} f_4)\ (f_2\text{-} f_3)]^{1/2}$ where $f_{1,2,3,4}$ are the observed resonant frequencies in order of the four lines comprising the AB multiplet, J_{AB} is the coupling constant and υ_0 is the spectrometer frequency. In molecules containing multiple spin systems of the form ABX, the separate systems, identified by COSY, are denoted with prime (′) and double prime (″) e.g. A of ABX or B′ of ABX′.

Melting points (mp) were determined on a GallenKamp apparatus and are uncorrected. Optical rotation ($[\alpha]_D^{25}$) were obtained on a JASCO J-815 spectropolarimeter at 25°C using a cell with pathlength of 0.25 dm. solvent and concentration are stated with individual readings. Infra-red spectra (IR) were recorded on an ATi Perkin Elmer Spectrum RX1 FT-IR. Only absorption maxima (ν_{max}) of interest are reported and quoted in wavenumbers (cm^{-1}). Low and high resolution mass spectra were recorded by staff at the University of Manchester. Electrospray (ES) spectra were recorded on a Waters Platform II. High resolution mass spectra (HRMS) were recorded on a Thermo Finnigan MAT95XPand are accurate to ±0.001 Da.

2. Materials and General Experimental Procedures for Synthesis

2.1 Materials

All reactions were carried out in oven-dried glassware under an atmosphere of nitrogen using standard anhydrous techniques. All reagents were obtained from commercially available sources and used without further purification, or where indicated prepared internally. Air- and moisture- sensitive liquids and solutions were transferred *via* syringe or stainless steel cannula. Anhydrous dichloromethane and tetrahydrofuran (THF) were obtained by distillation from calcium hydride, and sodium wire with a benzophenone indicator, respectively. Other anhydrous reaction solvents were obtained using Innovative Technologies Puresolve P5-mp-5 solvent purification system. Reactions performed at 0 °C were done so using an ice bath. All products were dried on a rotary evaporator followed by connection to a high vacuum system to remove any residual solvent. Flash chromatography was performed on silica gel (Merck 60H, 40-60 nm, 230 – 300 mesh). Analytical thin layer chromatography (TLC) was performed on Macherey Nagel alugram SIL G/UV254 and were visualised by UV (254 nm) and ninhydridrin or phosphomolybdic acid dips where appropriate.

1-Pyrenemethanol, 1-pyrenecarbaldehyde **24**, (1*R*,2*R*)-1,2-bis(2-hydroxyphenyl)ethylenediamine **26**, (1*S*,2*S*)-1,2-bis(2-hydroxyphenyl)ethylenediamine **49** and Acylase 1 from *Aspergillus melleus* were obtained from Sigma-Aldrich UK.

2.2. General experimental procedures

N-Boc-L-Pyrenylalanine **1**, L-pyrenylalanine methyl ester **2** and D-pyrenylalanine methyl ester **3** were prepared according to a literature procedure.[1] 1-Bromomethylpyrene was prepared from 1-pyrenemethanol according to a literature procedure.[2] Aminoalcohol **15** was prepared *in situ* by reduction of corresponding azide **18** with PEt_3. (2*S*,3*R*)-Dimethyl 2-azido-3-hydroxysuccinate **16** was prepared according to a literature procedure.[3]

The syntheses of H-Aib_4O^tBu,[4] Cbz(L-Phe)Aib_4OH **7**,[5] Cbz(D-Phe)Aib_4OH **8**,[5] (1*S*,2*S*)-1,2-(1-pyrene)ethylenediamine dihydrochloride **27**,[6,7] Cbz(L-αMeVal)Aib_4OH **33**,[6] Cbz(D-αMeVal)Aib_4OH **34**,[4] Cbz(L-αMeVal)Aib_4(*S,S*-BisPyrEt)NH_2 **35**,[6] Cbz-(D-αMeVal)Aib_4(*S,S*-BisPyrEt)NH_2 **36**,[6] Cbz(L-αMeVal)Aib_4(*S,S*-BisPyrEt)NHAc **37**,[6] Cbz(D-αMeVal)Aib_4(*S,S*-BisPyrEt)NHAc **38**,[6] CbzGlyAib_4OH **39**,[8] CbzGlyAib_4(*S,S*-BisPyrEt)NH_2 **40**,[6] CbzGlyAib_4(*S,S*-BisPyrEt)NHAc **41**,[6] N_3Aib_4OH **42**,[8] Cbz(L-αMeVal)$_2Aib_4$OH **45**,[6] Cbz-(D-αMeVal)$_2Aib_4$OH

46,[4,6] Cbz(L-αMeVal)$_2$Aib$_4$(*S,S*-BisPyrEt)NHAc **47**,[6] and Cbz(D-αMeVal)$_2$Aib$_4$*(S,S*-BisPyrEt)NHAc **48**,[6] have been reported previously.

General procedure 1:

Coupling of pyrene probe to the C terminus of peptides

The peptide R-Aib$_n$-OH (1 eq.), HOBt (1.2 eq.), (1*S*,2*S*)-1,2-(1-pyrene)ethylenediamine dihydrochloride **7** (1.1 eq.), EDC.HCl (1.1 eq.) and CH_2Cl_2 (30 mL/mmol) were combined, giving a suspension. DIPEA (3.7 eq.) was added, giving a homogenous solution which was stirred for 2 days. The reaction mixture was concentrated and re-dissolved in EtOAc (150 mL/mmol) and washed with $NaHCO_3$ (3 × 30 mL/mmol) and brine (3 × 30 mL/mmol), dried ($MgSO_4$), filtered and concentrated to give the crude product. This was purified by the method specified for each individual compound.

General procedure 2:

Sequential deprotection of Boc-pyrene probe and coupling to peptide C-terminus

Deprotection: The Boc protected *bis* pyrene fragment (0.039 mmol, 1.0 eq) was suspended in anhydrous DCM (25 mL) and cooled to at 0 °C. TFA (1 mL) was added dropwise with stirring and the solution was left to stir for 2 h, warming to RT. The reaction completion was confirmed by TLC and the solvent was concentrated under reduced pressure. Repeat azeotroping with Et_2O removed residual TFA. The crude salt was placed under high vacuum for an hour. The deprotected salt was dissolved in 1 mL of a suitable mixture of DCM/DMF and used directly in the coupling procedure.

Coupling: A suspension of Cbz-Phe-Aib$_4$OH (0.039 mmol, 1.0 eq.) and HOBt (1.1 eq.) in anhydrous DCM 0.5 mL was cooled to 0 °C. To this was added EDC (1.0 eq.). Once the suspension had fully dissolved, the solution of de-protected amine was added to the reaction and left to stir for three days. After checking reaction completion by TLC the solvent was concentrated under reduced pressure. The residue was dissolved in EtOAc (25 mL). The solution was then washed sequentially with $KHSO_4$ (3 × 5 mL), $NaHCO_3$ (3×5 mL), brine (5 mL) and dried ($MgSO_4$). Removal of the solvent under reduced pressure gave the crude product which was purified by column chromatography.

General procedure 3:

Acetylation of C terminal amines

The peptide (1 eq.) was dissolved in CH_2Cl_2 (40 mL/mmol). Ac_2O (1.5 eq.) was added and the resulting solution stirred for 16 h. The solution was concentrated and the crude product purified by the method specified for each individual compound.

General procedure 4:

Sequential coupling and acetylation of pyrene probe at peptide C terminus

The peptide R-Aib_n-OH (1 eq.), HOBt (1.3 eq.), (1*S*,2*S*)-1,2-(1-pyrene)ethylenediamine dihydrochloride (1.1 eq.), EDC.HCl (1.1 eq.) and CH_2Cl_2 (30 mL/mmol) were combined, giving a suspension. NEt_3 (5 eq.) was added giving a homogenous solution which was stirred for 2 days. The reaction mixture was diluted with CH_2Cl_2 (90 mL/mmol) and washed with distilled water (4×18 mL/mmol), dried (Na_2SO_4), filtered and concentrated to give the crude residue. This was re-dissolved in CH_2Cl_2 (20 mL/mmol), Ac_2O (1.5 eq.) was added and the resulting solution was stirred at RT for 16 h. The solvent was removed under reduced pressure, and the crude product purified by the method specified for each individual compound.

General procedure 5:

Coupling of C-deprotected peptides with azide *via* Staudinger Vilarrasa ligation

The C-deprotected peptide fragment (1.0 equiv) and HOBt (1.2 equiv) were dissolved in dry THF (30 mL/mmol). EDC (1.2 equiv) was added *via* syringe and the mixture stirred for 30 min. A solution of the azide fragment (1.0 equiv) in dry THF (30 mL/mmol) was added, followed by the dropwise addition of PEt_3 (1.0M solution in THF, 2.0 equiv). The mixture 0.38was stirred overnight at room temperature then quenched with $NaHCO_3$ (sat. solution, 30 mL/mmol). The mixture was extracted with CH_2Cl_2 (3 × 100 mL/mmol) and the combined organic extracts washed with HCl (1N, 100 mL/mmol), $NaHCO_3$ (sat. solution, 2 × 100 mL/mmol) and brine (100 mL/mmol), dried over $MgSO_4$, filtered and concentrated under reduced pressure to yield a crude product that was purified by column chromatography using the appropriate mixture of eluents.

2.3. Abbreviations

Ac = acetate, Aib = aminoisobutyric acid, Boc = *tert*-Butyloxycarbonyl, *S*,*S*-BP = (1*S*,2*S*)-1,2-bis(1-pyrenyl)ethan-1-amine, br = broad, Bu = butyl, Cbz = carboxybenzyl, CD = circular dichroism, d = doublet, DCM = dichloromethane, DIPEA = *N*,*N'*-diisopropylethylamine, DMF = *N,N'*-dimethylformamide, DMSO = dimethylsulfoxide, EDC = *N*-(3-dimethylaminopropyl)-*N*-ethylcarbodiimide, eq. = equivalent(s), ES = electrospray, Et = ethyl, Gly = glycine, HRMS = high resolution mass spectrometry, HOBt = 1-hydroxybenzotriazole; Hz = Hertz, IPA = isopropyl alcohol, IR = Infra-red, m = multiplet, Me = methyl, mp = melting point, αMv = α-methylvaline, NMR = nuclear magnetic resonance, Phe = phenylalanine, py = pyridine, Pya = 3-(1'-pyrenyl)alanine, Pyr = 1-pyrenyl; q = quartet, RT = room temperature, s = singlet, TFA = trifluoroacetic acid, THF = tetrahydrofuran, TLC = thin layer chromatography.

3. Synthetic Procedures

Synthesis of Boc(L-Pya)(D-Pya)OMe 4

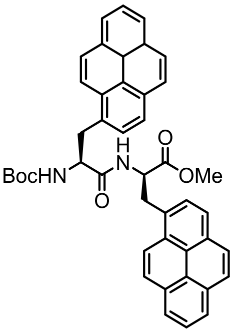

Boc-L-Pya (156 mg, 0.39 mmol, 1 eq.) was dissolved in 2 mL dry DCM and to this solution was added HOBt (71.6 mg, mmol, 1.3 eq.) and the suspension was cooled to 0 °C. EDC (71 mg, mmol, 1 eq.) was added to the suspension which was left to stir for 30 mins at 0 °C until the solid dissolved into solution. At this point D-Pya-OMe (151 mg, mmol, 1.1 eq.) was added followed by DIPEA (0. 27 mL, 2.5 eq.). The reaction was left to stir for 12 h. The reaction solvent was removed under reduced pressure to give a crude residue. This was purified by column chromatography, eluting with a solvent gradient of 1-2.5% acetone in chloroform to give the title compound as an off-white solid (137 mg, 50%) **mp** 214 °C; **$[\alpha]_D^{25}$** = +146.4 (c = 1.0; DMSO); **IR** (ATR, cm^{-1}) 3305, 1737, 1685, 1655; **^{1}H NMR** (500 MHz, $(CD_3)_2SO$): δ_H 8.83 (d, *J* = 7.9, 1H, NH), 8.39 - 8.22 (m, 7H, PyrH), 8.19 - 7.96 (m, 10H, PyrH), 7.76 (d, *J* = 7.9, 1H, PyrH), 6.84 (d, *J* = 9.1, 1H, NH), 4.82 (td, X' of ABX, *J* = 8.5, 5.4, 1H, $^{\alpha}$CH), 4.48 (td, X of ABX' *J* = 9.0, 4.5, 1H, $^{\alpha}$C′H), 3.87 (dd, A of ABX *J* = 14.0, 5.7, 1H, Pyr-$^{\beta}$CH_AH$_B$), 3.65 (s, 3H, OMe), 3.59 (dd, B of ABX *J* = 14.0, 9.0, 1H, Pyr-$^{\beta}$CH$_A$$H_B$), 3.45 (dd, A' of ABX' *J* = 14.0, 4.5, 1H, Pyr-$^{\beta}$C′H$_A$H$_B$), 3.11 (dd, B' of ABX' *J* = 14.0, 9.8, 1H, Pyr-$^{\beta}$C′H_AH$_B$), 1.14 (s, 7H, Boc), 0.78 (br. s, 2H, Boc) ppm; **^{13}C NMR** (125 MHz, $(CD_3)_2SO$): δ_C 171.8, 171.4, 154.9, 132.1, 131.4, 130.79, 130.76, 130.33, 130.28, 129.9, 129.5, 128.7, 128.6, 128.5, 128.4, 127.7, 127.4, 127.3, 127.1, 126.9, 126.7, 126.2, 126.1, 125.2, 125.1, 124.9, 124.8, 124.6, 124.4, 124.2, 124.02, 123.99, 123.96, 123.5, 122.9, 79.2, 77.9, 55.1, 53.5, 52.1, 35.2, 34.5, 27.9 ppm; **MS** (ES^+) 697 (100%; $[M+Na]^+$); **HRMS** (ES^+, TOF) Calcd for $C_{44}H_{38}N_2O_5 + Na^+$ = 697.2673, found 697.2679.

Synthesis of Boc(L-Pya)(L-Pya)OMe 5

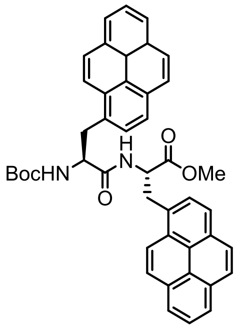

Boc-L-Pya (149 mg, 0.38 mmol, 1 eq.) was dissolved in 2 mL dry DCM (68.8 mg, mmol, 1.3 eq) HOBt was added and the suspension was cooled to 0 °C. EDC (67 µL, mmol, 1 eq.) was added to the suspension which was left to stir for 30 mins at 0 °C until the solid dissolved. At this point L-Pya-OMe (130 mg, 0.38 mmol, 1 eq.) was added followed by DIPEA (0.25 mL, 2.5 eq). The reaction was left to stir for 12 h. The reaction solvent was removed under reduced pressure to give a crude residue. This residue was purified by column chromatography, eluting with a solvent gradient of 1-2.5% acetone in chloroform to give the title compound as an off-white solid (150 mg, 57%) **Rf** 0.45 (5% acetone/chloroform) **mp** 212-214 °C; **$[\alpha]_D^{25}$** = -85.2 (c = 1.0; DMSO); **IR** (ATR, cm^{-1}) 3313, 1737, 1659; **^{1}H NMR** (500 MHz, $(CD_3)_2SO$): δ_H 8.57 (d, *J* = 7.6, 1H, NH), 8.39 (d, *J* = 9.1, 1H, PyrH), 8.34 - 8.24 (m, 6H, PyrH), 8.21 (d, *J* = 7.9, 1H, PyrH), 8.18 - 8.03 (m, 8H, PyrH), 7.97 (d, *J* = 7.9, 1H, PyrH), 7.81 (d, *J* = 7.9, 1H, PyrH), 6.98 (d, *J* = 8.8, 1H, NH), 4.81 (dd, X of ABX, *J* = 14.5, 7.9, 1H, $^{\alpha}$CH), 4.43 (td, X' of ABX', *J* = 9.0, 5.0, 1H, $^{\alpha}$C′H), 3.88 (dd, A of ABX, *J* = 14.0, 6.1, 1H, Pyr-$^{\beta}$CH_AH_B), 3.72 (dd, B of ABX, *J* = 14.0, 8.5, 1H, Pyr-$^{\beta}$CH_AH_B), 3.58 (dd, A' of ABX', *J* = 14.0, 5.0, 1H, Pyr-$^{\beta}$C′H_AH_B), 3.53 (s, 3H, OCH_3), 3.40 (dd, B' of ABX' *J* = 14.0, 9.5, 1H, Pyr-$^{\beta}$C′H_AH_B) 1.16 (s, 7H, Boc) 0.75 (s, 2H, Boc); **^{13}C NMR** (125 MHz, $(CD_3)_2SO$): δ_C 171.7, 171.4, 154.9, 132.1, 131.4, 130.81, 130.76, 130.3 (2C), 129.9, 129.6, 128.64, 128.62, 128.5, 128.3, 127.7, 127.4, 127.3, 127.2, 126.9, 126.7, 126.2, 126.1, 125.2, 125.1, 125.0, 124.8, 124.7, 124.5, 124.2, 124.1, 124.03, 124.01, 123.3, 123.0, 78.1, 55.8, 51.9, 35.2, 34.4, 27.9 ppm; **MS** (ES^+) 697 (100%; $[M+Na]^+$); **HRMS** (ES^+ TOF) Calcd for $C_{44}H_{38}N_2O_5+H^+$ = 675.2853, found 675.2853.

Synthesis of Boc(L-Pya)NHCH$_2$Pyr 6

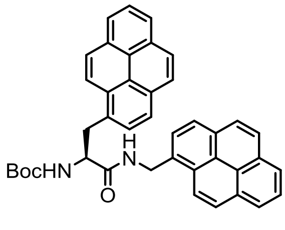

Boc-L-Pya **1** (50 mg, 0.39 mmol, 1 eq.) was dissolved in 1 mL dry DCM. 22.9 mg (1.3 eq) HOBt was added and the suspension was cooled to 0 °C. 33.5 μL (1 eq) of EDC was added to the suspension which was left to stir for 30 mins at 0 °C until the solid dissolved into solution. At this point 1-pyrenemethylamine hydrochloride (51.1 mg, 1.5 eq) was added followed by 102 μL DIPEA (2.5 eq). The reaction was left to stir for 12 h. The reaction solvent was removed under reduced pressure to give a residue. This was washed sequentially with $KHSO_4$ (3 × 20 mL) $NaHCO_3$ (3 × 20 mL) and brine (20 mL). The organic layer was then dried ($MgSO_4$) and removed under reduced pressure do give a crude residue. This was triturated with MeCN and dried under reduced pressure to give the desired product (72.9 mg, 97%) **mp** 210-212 °C; **$[\alpha]_D^{25}$** = +13.2 (C=1 in DMSO); **IR** (ATR, cm^{-1}) 3275, 3039, 2967, 2929, 1683, 1644, 1522, 1290, 1167, 841; **^{1}H NMR** (500 MHz, $(CD_3)_2SO$): δ_H 8.73 (t, *J* = 5.5, 1H, N*H*CH$_2$Pyr), 8.47 (d, *J* = 9.1, 1H, PyrH), 8.02 - 8.32 (m, 15H, PyrH), 7.97 (d, *J* = 7.9, 1H,), 7.84 (d, *J* = 7.9, 1H), 7.23 (d, *J* = 8.5, 1H, NH-Boc), 5.02 (d, *J* = 5.7, 2H, NHC*H*$_2$Pyr), 4.55 (td, X of ABX *J* = 9.0, 6.3, 1H, $^{\alpha}$CH-Pya), 3.81 (dd, A of ABX, *J* = 13.6, 5.7, 1H, $^{\beta}$C*H*$_A$H$_B$-Pya), 3.55 (dd, B of ABX, *J* = 14.0, 9.0, 1H, $^{\beta}$CH$_A$*H*$_B$-Pya), 1.21 (s, 7H, Boc), 0.82 (br. s., 2H, Boc); **^{13}C NMR** (100 MHz, $(CD_3)_2SO$): δ_C 171.9, 155.7, 132.9, 132.7, 131.3, 131.2, 130.8, 130.7, 130.5, 130.1, 129.2, 129.1, 128.4, 127.91, 127.86, 127.82, 127.77, 127.4, 127.2, 126.8, 126.7, 126.6, 125.7, 125.6, 125.5, 125.3, 125.07, 124.99, 124.6, 124.5, 124.4, 124.3, 124.0, 123.6, 78.6, 56.7, 41.0, 35.8, 28.5; **MS** (ES$^+$) 625 (5%; [M+Na]$^+$) **HRMS** (ES$^+$ TOF) Calcd for $C_{41}H_{34}N_2O_3+H^+$ = 603.2642, found 603.2628.

Synthesis of Cbz(L-Phe)Aib$_4$(L-Pya)(L-Pya)OMe 9

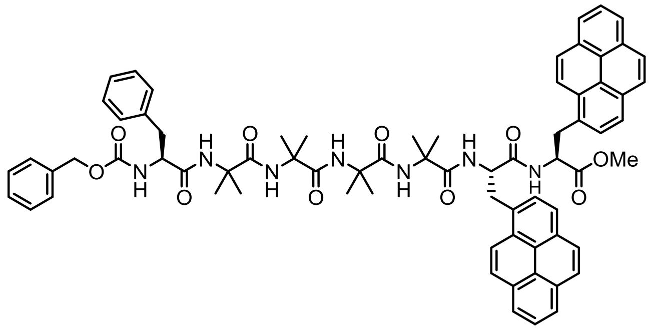

Cbz-L-PheAib$_4$OH **7** (25.0 mg, 0.039 mmol 1 eq.) was coupled to Boc-L-Pya-L-Pya**4** (1.2 eq.) using General procedure 2: Deprotected **4** was added to the activated acid in 0.5 mL of anhydrous DCM. The resulting residue was purified using column chromatography (1-5% IPA/DCM) to give the title compound (34.5 mg, 75%) as a white solid **mp** 122-126 °C; **[α]$_D$** = -17.1 (c = 1.0, $CHCl_3$), **IR** (ATR, cm^{-1}) 3296, 2921, 2852, 1655, 1530, 1455, 1260, 1022; **^{1}H NMR** (500 MHz, $CDCl_3$): δ_H 8.51 (d, J = 9.1, 1H, PyrH), 8.47 - 8.40 (m, 2H, PyrH + NH), 8.26 - 7.93 (m, 16H, PyrH), 7.63 (br. s., 2H, 2×NH), 7.34 - 7.23 (m, 8H, ArH), 7.19 - 7.14 (m, 2H, ArH), 7.08 (s, 1H, NH), 6.62 (s, 1H, NH), 5.64 (d, J = 2.8, 1H, NH), 5.15 (apparent t, J = 7.5, 1H, $^{\alpha}$CH), 5.08 (d, J = 12.3, 1H, PhCH_AH$_B$O), 4.99 - 4.91 (m, 2H $^{\alpha}$CH' + PhCH$_A$$H_B$O), 4.36 (dd, J = 15.1, 2.8, 1H, Ar-$^{\beta}$C'H_AH$_B$), 4.18 - 4.08 (m, 2H, Ar-$^{\beta}$CH_AH$_B$+ $^{\alpha}$CH''), 3.99 (dd, J = 13.9, 6.9, 1H, Ar-$^{\beta}$CH$_A$$H_B$), 3.69 (t, J = 12.6, 1H, Ar-$^{\beta}$C'H$_A$$H_B$), 3.45 (s, 3H, OCH_3), 3.11 (dd, J = 14.3, 5.2, 1H, Ar-$^{\beta}$C''H_AH$_B$), 2.89 (dd, J = 14.3, 8.7, 1H, Ar-$^{\beta}$C''H$_A$$H_B$), 1.65 (s, 3H, CH_3), 1.57 (s, 3H, CH_3), 1.53 (s, 3H, CH_3), 1.34 (s, 6H, 2×CH_3), 1.30 (s, 3H, CH_3), 1.23 (s, 3H, CH_3), 1.05 (s, 3H, CH_3). **^{13}C NMR** (125 MHz, $CDCl_3$): δ_C 175.8, 175.4, 175.3, 173.8, 172.5, 172.2, 171.7, 157.1, 136.0, 135.6, 133.1, 131.7, 131.5, 131.0, 130.6, 130.1, 129.4, 129.2 (2C), 129.1 (2C), 128.71 (2C), 128.65, 128.60, 128.2 (2C), 127.9, 127.8, 127.7, 127.6, 127.5, 127.1, 126.7, 125.9, 125.8, 125.2, 125.1 (2C), 125.01, 124.95, 124.8, 124.3, 123.6, 123.5, 67.6, 58.0, 57.2, 57.0, 56.7, 56.6, 55.2, 54.7, 52.1, 36.8, 35.9, 34.7, 26.94, 26.90, 26.8, 26.4, 26.3, 23.31, 23.27, 23.2 ppm; three aromatic resonance were not observed; **MS** ES$^+$ 1218 (15%, [M+Na]$^+$).

Synthesis of Cbz(D-Phe)Aib$_4$(L-Pya)(L-Pya)OMe 10

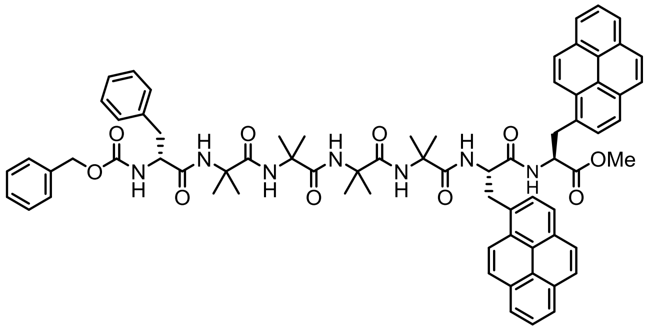

Cbz-D-PheAib$_4$OH **8** (25.0 mg, 0.039 mmol 1 eq.) was coupled to Boc-L-Pya-L-Pya **4** (1.2 eq.) using General procedure 1: Deprotected **4** was added to the activated acid in 0.5 mL of anhydrous DCM. The resulting residue was purified using column chromatography (1-5% IPA/DCM) to give the title compound (40.0 mg, 88%) as a white solid **mp** 148-153 °C **[α]$_D$** = +53.2 (*c*=1 in $CHCl_3$); **IR** (ATR, cm^{-1}) 3293, 2924, 2853, 1655, 1530; **^{1}H NMR** (400 MHz, $CDCl_3$): δ_H = 8.47 (d, *J* = 9.3, 1H, PyrH), 8.41 - 8.28 (m, 2H, PyrH + NH), 7.87 - 8.22 (m, 16H, PyrH), 7.87 - 7.78 (m, 2H, 2×NH), 7.59 (s, 1H, NH), 7.54 (s, 1H, NH), 7.31 - 7.22 (m, 8H, ArH), 7.13 - 7.00 (m, 3H, 2×ArH + NH), 6.53 (s, 1H, NH), 5.51 (d, *J* = 5.0, 1H, NH), 5.14 - 5.11 (m, *J* = 7.6, 1H $^{\alpha}$CH), 5.04 (d, *J* = 13.0, 1H, A of AB, PhCH_AH$_B$O), 4.96 (d, *J* = 13.0, 1H, B of AB, PhCH$_A$$H_B$O), 4.84 (t, *J* = 7.7, 1H, $^{\alpha}$C′H), 4.22 (dd, *J* = 14.5, 3.2, 1H, Ar-$^{\beta}$C′H_AH$_B$), 4.10 (dd, *J* = 13.9, 7.8, 1H, Ar-$^{\beta}$CH_AH$_B$) 3.98 - 3.91 (m, 1H, $^{\alpha}$C″H) 3.93 (dd, *J* = 13.9, 6.3, 1H, Ar-$^{\beta}$CH$_A$$H_B$), 3.62 (dd, *J* = 14.5, 11.1, 1H, Ar-$^{\beta}$C′H$_A$$H_B$)3.48 (s, 3H, OCH_3), 3.04 (dd, *J* = 14.0, 6.6, 1H, Ar-$^{\beta}$C″H_AH$_B$), 2.89 (dd, *J* = 14.0, 8.7, 1H, Ar-$^{\beta}$C″H$_A$$H_B$) 1.58 (s, 3H, CH_3), 1.53 (s, 3H, CH_3), 1.51 (s, 3H, CH_3), 1.29 (s, 3H, CH_3), 1.28 (s, 3H, CH_3), 1.15 (s, 3H, CH_3), 1.05 (s, 3H, CH_3) 0.95 (s, 3H, CH_3); **^{13}C NMR** (125 MHz, $CDCl_3$): δ_C 175.5, 175.2, 173.5, 172.4, 172.2, 171.3, 165.6, 156.7, 135.8, 135.7, 134.3, 133.3, 131.9, 131.5, 131.4, 131.1, 130.6, 130.2, 129.9, 129.5, 129.3 (2C), 129.2 (2C), 128.9 (2C), 128.8, 128.7, 128.2 (2C), 127.9, 127.71 (2C), 127.65, 127.5, 127.0, 126.6, 125.9, 125.7, 125.19, 125.16, 125.03, 124.98, 124.8, 124.7, 124.3, 123.8, 123.7, 67.7, 63.2, 57.8, 57.2, 57.0, 56.9, 55.2, 54.2, 52.1, 36.7, 36.0, 34.7, 32.1, 29.5, 26.9, 24.0, 23.9, 23.3, 22.9 ppm; **MS** ES$^+$ 1218 (15%, $[M+Na]^+$).

Synthesis of Cbz(L-Phe)Aib$_4$(L-Pya)(D-Pya)OMe 11

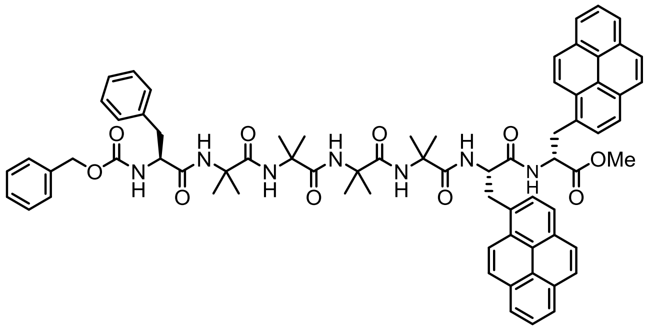

Cbz-L-PheAib$_4$OH **7** (25.0 mg, 0.039 mmol 1 eq.) was coupled to Boc-L-Pya-D-Pya **5** (1.2 eq.) using General procedure 1: Deprotected **5** was added to the activated acid in 1 mL of 1:1 anhydrous DCM/DMF. The resulting residue was purified using column chromatography (1-5% IPA/DCM) to give the title compound (18.7 mg, 41%) as a white solid **mp** 217-219 °C **[α]$_D$** = +12.8 (*c*=1: $CHCl_3$) **IR** 3275, 2919, 2850, 1645, 1534, 1455 **^{1}H NMR** (500 MHz, $CDCl_3$): δ_H 8.47-8.38 (m, 3H, 2 × PyrH + NH), 8.19 - 7.93 (m, 17H, 16 × PyrH + NH) 7.71 (br. s., 1H, NH), 7.47 (br. s., 1H, NH), 7.27 - 7.35 (m, 8H, ArH), 7.17-7.13 (m, 2H, ArH), 7.15 (br. s., 1H, NH), 7.03 (br. s., 1H, NH), 6.33 (br. s., 1H, NH), 5.31 (br. s., 1H, NH), 5.11 (d, *J* = 11.3, 1H, PhCH_AH$_B$O), 4.92 - 5.02 (m, 2H, PhCH$_A$$H_B$O + $^\alpha$CH), 4.89 (m, 1H, $^\alpha$C′H), 4.41 (d, *J* = 14.5, 1H, Ar-$^\beta$C′H_AH$_B$), 4.20 - 4.11 (m, 1H, $^\alpha$C′′H), 4.12 (dd, *J* = 14.2, 6.6, 1H, Ar-$^\beta$CH$_A$H$_B$), 3.99 (dd, *J* = 14.2, 7.9, 1H, Ar-$^\beta$CH$_A$$H_B$), 3.69 (dd, *J* = 13.7, 12.5, 1H, Ar-$^\beta$C′H$_A$$H_B$), 3.58 (s, 3H, OCH_3), 3.12 (dd, *J* = 13.7, 3.0, 1H, Ar-$^\beta$C′′H$_A$$H_B$), 2.89 (dd, *J* = 13.9, 8.2, 1H, Ar-$^\beta$C′′H$_A$$H_B$), 1.55 (br. s., 3H, CH_3) 1.53 (s, 3H, CH_3), 1.49 (s, 3H, CH_3), 1.35 (br. s., 3H, CH_3), 1.26 (br. s., 3H, CH_3), 1.20 (br. s., 6H, 2×CH_3), 1.09 (s, 3H, CH_3) **^{13}C NMR** (100 MHz, $CDCl_3$): δ_C 176.0, 175.8, 175.0, 173.5, 173.0, 172.6, 171.5, 157.0, 135.7, 135.1, 132.5, 131.31, 131.27, 131.2, 130.9, 130.8, 130.4, 130.1, 129.04 (2C), 128.96 (2C), 128.7, 128.62 (2C), 128.59, 128.1 (2C), 127.7, 127.64, 127.58 (2C), 127.5, 127.3, 126.8, 126.6, 125.7, 125.6, 125.0 (2C), 124.92, 124.85 (2C), 124.8, 124.7, 124.1, 123.4, 123.1, 67.7, 57.7, 57.0, 56.8, 56.7, 56.5, 55.2, 55.0, 52.2, 36.7, 34.6, 34.5, 33.4, 31.9, 29.6, 29.6, 29.4, 29.4, 23.3, 22.7 ppm; **MS** (ES$^+$, $CHCl_3$) 1219 (20%, [M+Na]$^+$).

Synthesis of Cbz(D-Phe)Aib$_4$(L-Pya)(D-Pya)OMe 12

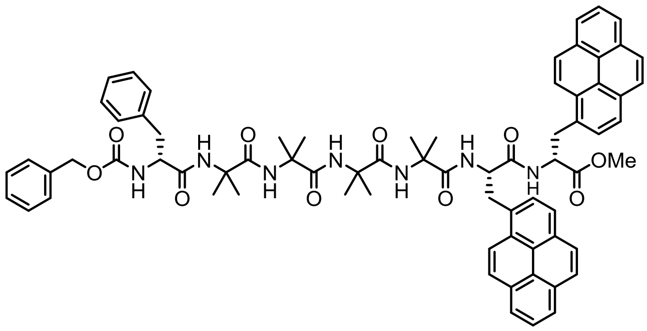

Cbz-D-PheAib$_4$OH **8** (25.0 mg, 0.039 mmol 1 eq.) was coupled to Boc-L-Pya-D-Pya **5** (1.2 eq.) using General procedure 1: Deprotected **5** was added to the activated acid in 1 mL of 1:1 anhydrous DCM/DMF. The resulting residue was purified using column chromatography (1-5% IPA/DCM) to give the title compound (25.5 mg, 56%) as a white solid **mp** 226-228 °C; **$[\alpha]_D^{25}$** = +28.0 (*c*=1; $CHCl_3$); **IR** 3293, 2923, 2852, 1652, 1532; **^{1}H NMR** (500 MHz, $CDCl_3$): δ_H 8.49 (d, *J* = 9.1, 1H, PyrH), 8.44 (d, *J* = 9.1, 1H, PyrH), 8.39 (d, *J* = 7.3, 1H, NH), 7.91 - 8.19 (m, 18H, 16×PyrH + 2×NH), 7.68 (s, 1H, NH), 7.52 (s, 1H, NH), 7.36 - 7.21 (m, 8H, ArH), 7.09 - 7.03 (m, *J* = 7.9, 2H, ArH), 6.28 (s, 1H, NH), 5.42 (d, *J* = 4.7, 1H, NH), 5.06 (d, *J* = 12.3, 1H, PhCH_AH$_B$O), 4.98 (dd, *J* = 15.0, 7.6, 1H, $^\alpha$CH), 4.97 (d, *J* = 12.3, 1H, PhCH$_A$$H_B$O) 4.87 (ddd, *J* = 11.0, 8.2, 2.5, 1H, $^\alpha$C′H), 4.44 (dd, *J* = 14.8, 1.9, 1H, Ar-$^\beta$C′H_AH$_B$), 4.14 (dd, *J* = 14.0, 7.4, 1H, Ar-$^\beta$CH_AH$_B$), 3.99 (dd, *J* = 14.0, 8.0, 1H, Ar-$^\beta$CH$_A$$H_B$), 3.89 (dd, *J* = 12.3, 7.3, 1H, $^\alpha$C″H), 3.66 (dd, *J* = 15.1, 11.7, 1H, Ar-$^\beta$C′H$_A$$H_B$), 3.56 (s, 3H, OCH_3), 3.03 (dd, *J* = 13.6, 6.9, 1H, Ar-$^\beta$C″H_AH$_B$), 2.90 (dd, *J* = 13.7, 8.4, 1H, Ar-$^\beta$C″H$_A$$H_B$), 1.60 (s, 3H, CH_3), 1.59 (s, 3H, CH_3), 1.53 (s, 3 H, CH_3), 1.31 (s, 3 H, CH_3), 1.25 (s, 6H, 2×CH_3), 1.24 (s, 3H, CH_3), 1.16 (s, 3H, CH_3) **^{13}C NMR** (125 MHz, $CDCl_3$): δ_C 175.8, 175.1, 174.9, 173.6, 172.8, 172.5, 171.2, 156.4, 135.9, 135.7, 133.1, 131.7, 131.3, 131.2, 131.0, 130.9, 130.4, 130.0, 129.1 (2C), 129.0, 128.9 (2C), 128.6 (2C), 128.5, 127.9 (2C), 127.69, 127.65, 127.6, 127.5, 127.4, 127.3, 126.7, 126.5, 125.7, 125.6, 125.0, 124.9, 124.83, 124.82, 124.79, 124.6, 124.1, 123.6, 123.3, 67.4, 57.5, 57.0, 56.8 (2C), 56.75, 55.1, 55.0, 51.9, 36.4, 34.8, 29.7, 29.3, 27.1, 26.8, 26.7, 23.3 (2C), 22.84, 22.76 ppm; **MS** ES$^+$ (100%, $[M+Na]^+$) 1218.

Synthesis of Cbz(L-Phe)Aib$_4$(L-Pya)NHCH$_2$Pyr 13

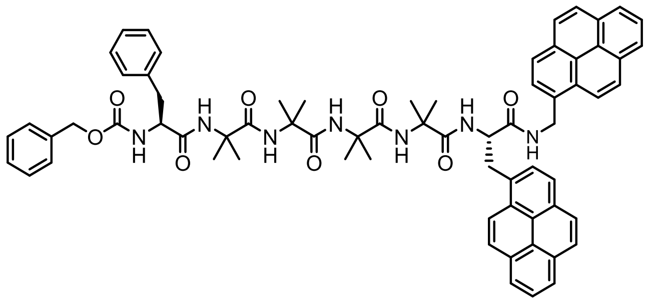

Cbz-L-PheAib$_4$OH **7** (25.0 mg, 0.039 mmol 1 eq.) was coupled to Boc-L-Pya-NH-Pyr **6** (1.2 eq.) using General procedure 2: Deprotected **6** was added to the activated acid in 1 mL of 1:1 anhydrous DCM/DMF. Purification with flash chromatography (1-5% IPA:DCM) gave the title compound (32.9 mg, 75%) as an off white solid **mp** 144-147 °C; **[α]$_D$** = +6.4 (*c*=1; $CHCl_3$); **IR** (ATR, cm^{-1}) 3287, 3040, 2921, 2850, 1651, 1527, 1455, 1234, 1171; **^{1}H NMR** (500 MHz, $CDCl_3$): δ_H = 8.63 (d, *J* = 9.5, 1H, PyrH), 8.51 (d, *J* = 9.1, 1H, PyrH), 8.39 (t, *J* = 5.8, 1H, NH, N*H*CH$_2$Pyr), 8.24 - 8.11 (m, 7H, PyrH), 8.10 - 7.95 (m, 7H, PyrH), 7.84 (s, 1H, NH), 7.82 (s, 1H, NH), 7.50 (s, 1H, NH), 7.39 - 7.27 (m, 10H, ArH), 7.16 - 7.12 (m, 2H, ArH), 6.95 (s, 1H, NH), 6.17 (s, 1H, NH), 5.49 (dd, *J* = 15.1, 6.6, 1H, A of ABX, NHCH_AH$_B$Pyr), 5.20 (dd, *J* = 15.1, 5.4, 1H, B of ABX, NHCH$_A$$H_B$Pyr), 5.16 (d, *J* = 3.5, 1H, NH), 5.13 (d, *J* = 12.3, 1H, PhCH_AH$_B$O), 5.08 (ddd, *J* = 11.3, 8.8, 2.5, 1H, $^{\alpha}$CH), 4.96 (d, *J* = 12.3, 1H, PhCH$_A$$H_B$O), 4.63 (dd, *J* = 14.8, 2.5, 1H, Ar-$^{\beta}$CH_AH$_B$), 4.10 (ddd, *J* = 8.3, 5.3, 3.2, 1H, $^{\alpha}$C′H), 3.66 (dd, *J* = 14.8, 11.7, 1H, Ar-$^{\beta}$CH$_A$$H_B$), 3.10 (dd, *J* = 14.3, 5.5, 1H, Ar-$^{\beta}$C′H_AH$_B$), 2.88 (dd, *J* = 14.2, 8.5, 1H, Ar-$^{\beta}$C′H$_A$$H_B$), 1.43 (s, 3H, CH_3), 1.36 (s, 3H, CH_3), 1.30 (s, 3H, CH_3), 1.27 (s, 6H, CH_3), 1.19 (s, 3H, CH_3), 1.11 (s, 3H, CH_3), 0.96 (s, 3H, CH_3); **^{13}C NMR** (125 MHz, $CDCl_3$): δ_C 175.6, 175.3, 174.7, 173.3, 172.5, 171.2, 157.1, 135.7, 135.1, 133.5, 132.8, 131.5, 131.4, 131.2, 131.1, 130.7, 130.2, 129.4, 129.23, 129.1, 129.0, 128.9, 128.4, 128.0, 127.9, 127.8, 127.7, 127.5 (2C), 126.8, 126.7, 126.7, 125.8, 125.7, 125.2, 125.1, 125.03, 124.96, 124.8, 124.7, 124.2, 123.9, 123.8, 68.1, 57.8, 57.1, 56.81, 56.78, 55.3, 54.2, 41.7, 37.0, 35.4, 27.1, 27.0, 26.6, 23.5, 23.2, 23.1, 22.8, 22.6 ppm; four aromatic resonances were not observed; **MS** (ES$^+$) 1146 (100%, [M+Na]$^+$).

Synthesis of Cbz(D-Phe)Aib$_4$(L-Pya)NHCH$_2$Pyr 14

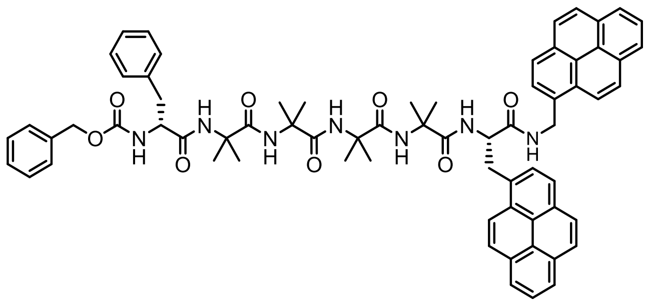

Cbz-D-PheAib$_4$OH **8** (25.0 mg, 0.039 mmol 1 eq.) was coupled to Boc-L-Pya-NH-Pyr **6** (1.2 eq.) using General procedure 2: Deprotected **6** was added to the activated acid in 1 mL of 1:1 anhydrous DCM/DMF. Purification with flash chromatography (1-5% IPA:DCM) gave the title compound (23.3 mg, 53%) as an off white solid. **mp** 110-112 °C; **[α]$_D$** = +11.6 (*c* = 1.0, $CHCl_3$); **IR** (ATR, cm^{-1}) 3295, 2924, 2852, 1656, 1527, 1455, 1208, 1164; **^{1}H NMR** (500 MHz, $CDCl_3$): δ_H 8.57 (d, *J* = 9.5, 1H, PyrH), 8.49 - 8.44 (m, 2H, NH + PyrH), 8.21 - 8.10 (m, 7 H, PyrH), 8.09 - 7.93 (m, 9H, PyrH), 7.91 (s, 1H, NH), 7.89 (s, 1H, NH), 7.57 (s, 1H, NH), 7.44 (s, 1H, NH), 7.23 - 7.27 (m, 3H, ArH), 7.23 - 7.16 (m, 5H, ArH), 7.08 (s, 1H, NH), 7.02 – 6.98 (m, 2H, ArH), 6.61 (s, 1H, NH), 5.52 (d, *J* = 4.7, 1H), 5.47 (dd, *J* = 15.0, 6.6, 1H, NHCH_AH$_B$Pyr), 5.13 (dd, *J* = 15.0, 5.2, 1H, NHCH$_A$$H_B$Pyr), 5.03 – 4.97 (m, 1H, $^\alpha$CH), 4.99 (d, *J* = 12.3, 1H, A of AB PhCH_AH$_B$O), 4.89 (d, *J* = 12.3, 1H, B of AB PhCH$_A$$H_B$O), 4.54 (dd, *J* = 14.5, 2.5, 1H, Ar-$^\beta$CH_AH$_B$), 3.89 (dd, *J* = 12.6, 7.3, 1H, $^\alpha$C′H), 3.65 (dd, *J* = 14.5, 11.5, 1H, Ar-$^\beta$CH$_A$$H_B$), 2.96 (dd, *J* = 13.6, 6.6, 1H, Ar-$^\beta$C′H_AH$_B$), 2.81 (dd, *J* = 13.7, 8.4, 1H, Ar-$^\beta$C′H$_A$$H_B$), 1.43 (s, 3H, CH_3), 1.31 (s, 3H, CH_3), 1.26 (s, 3H, CH_3) 1.21 (s, 3H, CH_3), 1.20 (s, 3H, CH_3), 1.14 (s, 3H, CH_3), 1.08 (br. s, 3H, CH_3), 0.97 (s, 3H, CH_3) ppm; **^{13}C NMR** (125 MHz, $CDCl_3$): δ_C 175.7, 175.5, 175.1, 173.8, 172.5, 171.4, 156.3, 136.0, 135.8, 132.9, 132.4, 131.3, 131.2, 130.88, 130.87, 130.5, 130.1, 129.1, 129.0, 128.72, 128.66, 128.55 (3C), 128.4, 127.9, 127.8 (2C), 127.6, 127.5, 127.4, 127.1, 126.7, 126.6, 126.5, 125.7, 125.0, 124.88, 124.86 (2C), 124.81 (2C), 124.78, 124.71, 124.2, 123.5, 123.5, 67.1, 57.2, 56.9, 56.6, 56.6, 56.5, 55.7, 41.5, 36.4, 35.3, 27.0, 26.7, 26.6, 26.4, 23.3, 22.8, 22.7, 22.6 ppm; **MS** (ES$^-$, MeOH): 1236.6 (100%, [M+TFA−H]$^-$).

Synthesis of (2*S*,3*R*)-2-azido-3-hydroxysuccinic acid 17

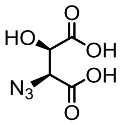

Anhydrous lithium iodide (3.16 g, 23.60 mmol) was added to a stirred solution of (2*S*,3*R*)-dimethyl 2-azido-3-hydroxysuccinate **16**(0.60 g, 2.95 mmol) in dry THF (30 mL), and heated to reflux for 48 h. The mixture was allowed to cool to room temperature and the precipitate was collected by filtration and washed repeatedly with cold Et_2O. The precipitate was washed through the filter with MeOH and concentrated under reduced pressure. The residue was diluted with 1N HCl (20 mL) and stirred for 1 h, then extracted with EtOAc (4 × 20 mL). The combined organic extracts were dried over $MgSO_4$ and concentrated under reduced pressure to yield the title compound **17** as a yellow oil, which was used without further purification (368 mg, 1.97 mmol, 66%). **$[\alpha]_D$**^{25} = +53.3 (*c* = 0.15, MeOH); **^{1}H NMR** (400 MHz, CD_3OD) δ_H 4.57 (d, *J* = 3.0 Hz, 1H, C(*H*)OH), 4.37 (d, *J* = 3.0 Hz, 1H, C(*H*)N_3) ppm; **^{13}C NMR** (100 MHz, CD_3OD) δ_C 173.9, 170.7, 73.3, 66.0 ppm; **IR** ν_{max} = 3372, 3257, 2942, 2118, 1721, 1629 cm^{-1}; **MS** (ES^-, MeOH) *m/z* = 174 ($[M-H]^-$, 100%); **HRMS** (ES^-, MeOH) Calcd for $C_4H_4N_3O_5$ = 174.0156, found 174.0159.

Synthesis of (2*S*,3*R*)-bis(pyren-1-ylmethyl) 2-azido-3-hydroxysuccinate 18 (N_3(2*S*,3*R*-BisPyrSucc)OH)

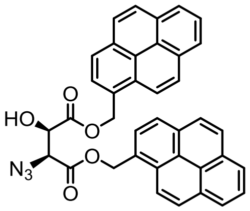

1-Bromomethylpyrene (602 mg, 2.03 mmol) was dissolved in dry MeCN (30 mL). (2*S*,3*R*)-2-azido-3-hydroxysuccinic acid **17** (170 mg, 0.97 mmol) and Et_3N (0.284 mL, 2.03 mmol) were added and the suspension heated to reflux for 72 h. The mixture was allowed cooled to room temperature and the solvent removed under reduced pressure. The residue was re-dissolved in CH_2Cl_2 (50 mL), washed with brine (10 mL), dried over $MgSO_4$, concentrated under reduced pressure and purified by silica gel column chromatography (CH_2Cl_2) to yield the title compound **18** as a yellow solid (137 mg, 0.23 mmol, 24%). **TLC** SiO_2/CH_2Cl_2 R_f = 0.16; **mp** 91 – 93 °C; $[\alpha]_D^{25}$ = +39.1 (*c* = 0.43, $CHCl_3$); **^{1}H NMR** (400 MHz, $CDCl_3$) δ_H 8.11 – 8.02 (m, 4H, Ar*H*), 7.97 – 7.90 (m, 4H, Ar*H*), 7.88 – 7.83 (m, 8H, Ar*H*), 7.70 – 7.65 (m, 2H, Ar*H*), 5.69 (d, A of AB(i), *J* = 12.0 Hz, 1H, PyrC$H_A$$H_B$ (i)), 5.63 (d, A of AB(ii), *J* = 12.5 Hz, 1H, PyrC$H_A$$H_B$ (ii)), 5.55 (d, B of AB(ii), *J* = 12.5 Hz, 1H, PyrC$H_A$$H_B$ (ii)), 5.53 (d, B of AB(i), *J* = 12.5 Hz, 1H, PyrC$H_A$$H_B$ (i)), 4.74 (dd, *J* = 5.5, 3.0 Hz, 1H, C(*H*)OH), 4.43 (d, *J* = 3.0 Hz, 1H, C(*H*)N_3), 3.39 (d, *J* = 5.5 Hz, 1H, O*H*) ppm; **^{13}C NMR** (100 MHz, $CDCl_3$) δ_C 170.7, 166.9, 131.6, 131.5, 131.79, 130.76, 130.21, 130.18, 129.0, 128.9, 128.11, 128.07, 127.7, 127.68, 127.18, 127.15, 126.92, 126.89, 126.7, 126.5, 125.8, 125.39, 125.37, 125.29, 125.27, 124.3, 124.14, 124.13, 124.12, 124.1, 122.0, 121.9, 72.2, 66.6, 66.2, 64.5 ppm; **IR** ν_{max} = 3480, 3362, 3044, 2115, 1742, 1605, 1590, 1186 cm^{-1}; **MS** (ES^+, MeCN) *m/z* = 626 ($[M+H]^+$, 90%).

Synthesis of (2*R*,3*R*)-bis(pyren-1-ylmethyl) 2-azido-3-hydroxysuccinate 19 (N_3(2*R*,3*R*-BisPyrSucc)OH)

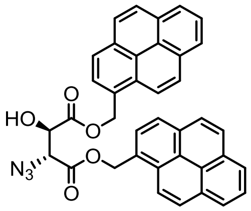

The title compound **19** was also isolated from the reaction: "Synthesis of (2*S*,3*R*)-bis(pyren-1-ylmethyl) 2-azido-3-hydroxysuccinate **18** after silica gel column chromatography (CH_2Cl_2) as a yellow solid (133 mg, 0.22 mmol, 23%). **TLC** SiO_2/CH_2Cl_2 R_f = 0.23; **mp** 87 – 90 °C; $[\alpha]_D^{25}$ = +7.8 (*c* = 0.77, $CHCl_3$); **^{1}H NMR** (400 MHz, $CDCl_3$) δ_H 8.20 – 7.95 (m, 18H, Ar*H*), 5.99 (d, A of AB(i), *J* = 12.0 Hz, 1H, PyrCH_AH_B (i)), 5.95 (d, A of AB(ii), *J* = 12.5 Hz, 1H, PyrCH_AH_B (ii)), 5.88 (d, B of AB(ii), *J* = 12.5 Hz, 1H, PyrCH_AH_B (ii)), 5.85 (d, B of AB(i), *J* = 12.0 Hz, 1H, PyrCH_AH_B (i)), 4.78 (dd, *J* = 6.0, 2.5 Hz, 1H, C(*H*)OH), 4.23 (d, *J* = 2.5 Hz, 1H, C(*H*)N_3), 3.31 (d, *J* = 6.0 Hz, 1H, O*H*) ppm; **^{13}C NMR** (100 MHz, $CDCl_3$) δ_C 170.0, 167.4, 132.1, 132.0, 131.02, 131.01, 130.48, 130.45, 129.59, 129.56, 128.58, 128.4, 128.1, 128.02, 128.98, 127.9, 127.21, 127.19, 127.16, 126.9, 126.2, 126.1, 125.7, 125.63, 125.58, 125.5, 124.73, 124.69, 124.5, 124.4, 122.5, 122.27, 72.0, 67.1, 66.7, 63.4 ppm; **IR** ν_{max} = 3463, 3042, 3022, 2962, 2925, 2854, 2117, 1741, 1214 cm^{-1}; **MS** (ES^+, MeCN) *m/z* = 626 ($[M+H]^+$, 100%).

Synthesis of Cbz-L-PheAib$_4$(2*S*,3*R*-BisPyrSucc)OH 20

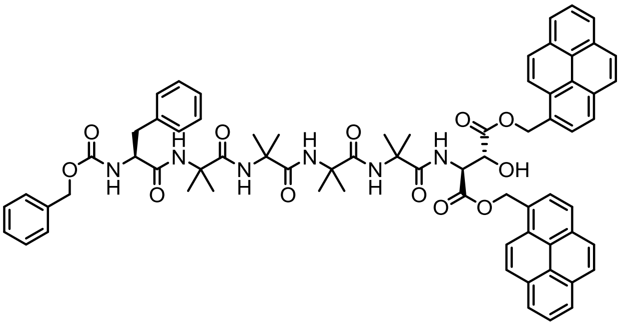

From a solution of Cbz-L-PheAib$_4$-OH **7** (53 mg, 0.083 mmol), HOBt (13 mg, 0.099 mmol), EDC (18 µL, 0.099 mmol), N_3(2*S*,3*R*-BisPyrSucc)OH **18** (50 mg, 0.083 mmol), PEt_3 (166 µL of a 1.0 M solution in THF, 0.166 mmol) in dry THF (6 mL), following general procedure 5 and after purification by silica gel column chromatography (EtOAc:CH_2Cl_2 0:100 → 30:70) the title compound **20** was obtained as a pale yellow amorphous solid (15 mg, 0.012 mmol, 15%). **TLC** SiO_2/EtOAc:CH_2Cl_2 (30:70) R_f = 0.18; **$[\alpha]_D^{25}$** = –8.8 (*c* = 1.0, $CHCl_3$); **^{1}H NMR** (400 MHz, $CDCl_3$) δ_H 8.02 – 7.57 (m, 20H, Ar*H* + 2 × N*H*), 7.47 (s, 1H, N*H*), 7.36 – 7.27 (m, 8H, Ar*H*), 7.16 – 7.14 (m, 2H, Ar*H*), 7.05 (s, 1H, N*H*), 6.12 (s, 1H, N*H*), 5.83 (d, A of AB(i), *J* = 12.5 Hz, 1H, PyrCH_AH_B), 5.69 (d, *J* = 5.0 Hz, 1H, O*H*), 5.52 (d, B of AB(i), *J* = 12.5 Hz, 1H, PyrCH_AH_B), 5.51 (d, A of AB(ii), *J* = 13.0 Hz, 1H, PyrCH_AH_B), 5.45 (dd, *J* = 9.0, 2.0 Hz, 1H, NHC(*H*)RC(H)ROH), 5.34 (d, *J* = 5.0 Hz, 1H, N*H*Phe), 5.28 (d, B of AB(ii), *J* = 13.0 Hz, 1H, PyrCH_AH_B), 5.10 (d, A of AB(iii), *J* = 12.5 Hz, 1H, PhCH_AH_BO), 5.02 (d, B of AB(iii), *J* = 12.5 Hz, 1H, PhCH_AH_BO), 4.85 (dd, *J* = 5.0, 2.5 Hz, 1H, NHC(H)RC(*H*)ROH), 3.99 (m, X of ABX, 1H, Phe-α-*H*), 3.11 (dd, A of ABX, *J* = 13.5, 7.0 Hz, 1H, PhCH_AH_BC(H)R_2), 2.99 (dd, B of ABX, *J* = 13.5, 8.0 Hz, 1H, PhCH_AH_BC(H)R_2), 1.57 (s, 3H, CH_3), 1.56 (s, 3H, CH_3), 1.48 (s, 3H, CH_3), 1.47 (s, 3H, CH_3), 1.46 (s, 3H, CH_3), 1.34 (s, 3H, CH_3), 1.26 (s, 3H, CH_3), 1.20 (s, 3H, CH_3) ppm; **^{13}C NMR** (100 MHz, $CDCl_3$) δ_C 175.8, 175.3, 174.4, 173.6, 171.4, 170.0, 168.7, 156.5, 136.0, 135.6, 131.3, 130.9, 130.4, 129.1, 128.9 128.8, 128.6, 127.9, 127.8, 127.6, 127.2, 127.1, 127.0, 126.1, 125.6, 125.5, 125.1, 125.0, 124.9, 124.3 124.2, 122.6, 122.0, 72.3, 67.4, 65.3, 64.9, 57.4, 57.1, 57.0, 56.7, 56.6, 36.3, 29.7, 27.3, 26.8, 26.4, 26.3, 23.3, 23.2, 23.0 ppm; fifteen aromatic resonances and one CH_2 resonance were obscured or not observed; **IR** ν_{max} = 3305, 3044, 2986, 2931, 1750, 1710, 1659, 1532 cm^{-1}; **MS** (ES$^+$, MeCN) *m/z* = 1216 ([M+NH_4]$^+$, 100%).

Synthesis of Cbz-D-PheAib$_4$(2*S*,3*R*-BisPyrSucc)OH 21

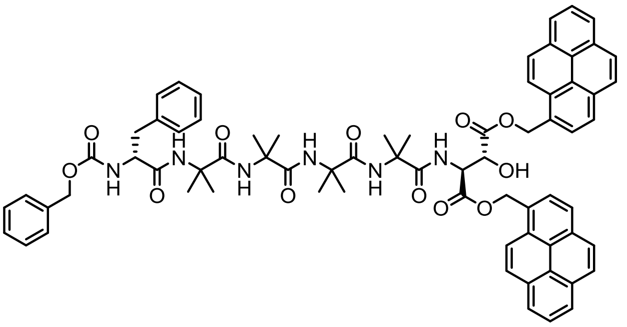

From a solution of Cbz-D-PheAib$_4$-OH **8** (53 mg, 0.083 mmol), HOBt (13 mg, 0.099 mmol), EDC (18 µL, 0.099 mmol), N_3(2*S*,3*R*-BisPyrSucc)OH **18** (50 mg, 0.083 mmol), PEt_3 (166 µL of a 1.0 M solution in THF, 0.166 mmol) in dry THF (6 mL), following general procedure 5 and after purification by silica gel column chromatography (EtOAc:CH_2Cl_2 0:100 → 30:70) the title compound **21** was obtained as a pale yellow amorphous solid (16 mg, 0.013 mmol, 16%). **TLC** SiO_2/EtOAc:CH_2Cl_2 (30:70) R_f = 0.17; **$[\alpha]_D$**^{25} = +1.3 (*c* = 1.0, $CHCl_3$); **^{1}H NMR** (400 MHz, $CDCl_3$) δ_H 8.02 – 7.56 (m, 20H, Ar*H* + 2 × N*H*), 7.52 (s, 1H, N*H*), 7.38 – 7.30 (m, 8H, Ar*H*), 7.21 – 7.19 (m, 2H, Ar*H*), 7.05 (s, 1H, N*H*), 6.23 (s, 1H, N*H*), 5.84 (d, A of AB(i), *J* = 12.5 Hz, 1H, PyrCH_AH_B), 5.73 (d, *J* = 5.0 Hz, 1H, O*H*), 5.52 (d, B of AB(i), *J* = 12.5 Hz, 1H, PyrCH_AH_B), 5.52 (d, A of AB(ii), *J* = 13.0 Hz, 1H, PyrCH_AH_B), 5.48 (dd, *J* = 9.0, 2.5 Hz, 1H, NHC(*H*)RC(H)ROH), 5.28 (d, B of AB(ii), *J* = 13.0 Hz, 1H, PyrCH_AH_B), 5.21 (d, *J* = 3.0 Hz, 1H, N*H*Phe), 5.15 (d, A of AB(iii), *J* = 12.0 Hz, 1H, PhCH_AH_BO), 4.99 (d, B of AB(iii), *J* = 12.0 Hz, 1H, PhCH_AH_BO), 4.87 (dd, *J* = 5.0, 2.5 Hz, 1H, NHC(H)RC(*H*)ROH), 4.16 (m, X of ABX, 1H, Phe-α-*H*), 3.16 (dd, A of ABX, *J* = 14.0, 5.5 Hz, 1H, PhCH_AH_BC(H)R_2), 2.93 (dd, B of ABX, *J* = 14.0, 8.5 Hz, 1H, PhCH_AH_BC(H)R_2), 1.58 (s, 3H, CH_3), 1.57 (s, 3H, CH_3), 1.51 (s, 3H, CH_3), 1.49 (s, 3H, CH_3), 1.46 (s, 3H, CH_3), 1.41 (s, 3H, CH_3), 1.35 (s, 3H, CH_3), 1.11 (s, 3H, CH_3) ppm; **^{13}C NMR** (100 MHz, $CDCl_3$) δ_C 175.6, 175.2, 174.4, 173.3, 171.4, 170.0, 168.7, 156.9, 135.6, 135.1, 131.3, 130.95, 130.93, 130.86, 130.44, 130.42, 129.2, 129.02, 128.96, 128.7 (2C), 128.3, 128.2 (2C), 128.02, 127.99, 127.98, 127.7, 127.6, 127.4, 127.2, 127.11, 127.10, 126.1, 125.6, 125.5, 125.06, 125.03, 124.99, 124.9, 124.4, 124.3, 124.24, 124.18, 124.1, 122.6, 122.0, 72.3, 67.8, 65.3, 64.9, 57.7, 57.2, 57.0, 56.7, 56.6, 56.5, 36.8, 27.3, 26.9, 26.34, 26.33, 23.4, 23.26, 23.24, 23.18 ppm; one aromatic resonance was not observed; **IR** ν_{max} = 3304, 3037, 2985, 2932, 1751, 1659, 1532 cm^{-1}; **MS** (ES$^+$, MeCN) *m/z* = 1216 ([M+NH_4]$^+$, 100%).

Synthesis of Cbz-L-PheAib$_4$(2*R*,3*R*-BisPyrSucc)OH 22

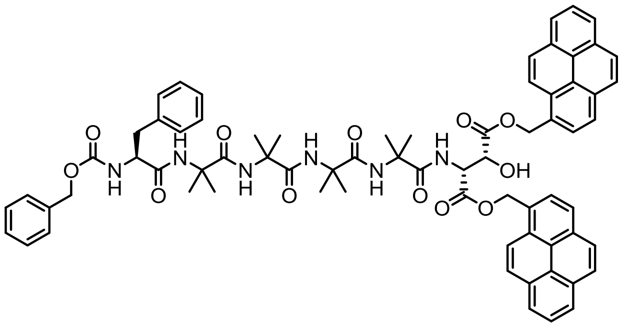

From a solution of Cbz-L-PheAib$_4$-OH **7** (53 mg, 0.083 mmol), HOBt (13 mg, 0.099 mmol), EDC (18 µL, 0.099 mmol), N_3(2*R*,3*R*-BisPyrSucc)OH **19** (50 mg, 0.083 mmol), PEt_3 (166 µL of a 1.0 M solution in THF, 0.166 mmol) in dry THF (6 mL), following general procedure 5 and after purification by silica gel column chromatography (EtOAc:CH_2Cl_2 0:100 → 30:70) the title compound **22** was obtained as a pale yellow amorphous solid (12 mg, 0.010 mmol, 12%). **TLC** SiO_2/EtOAc:CH_2Cl_2 (30:70) R_f = 0.19; **$[\alpha]_D^{25}$** = -13.6 (*c* = 1.0, $CHCl_3$); **^{1}H NMR** (400 MHz, $CDCl_3$) δ_H 8.03 – 7.57 (m, 20H, Ar*H* + 2 × N*H*), 7.48 (s, 1H, N*H*), 7.35 – 7.27 (m, 8H, Ar*H*), 7.16 – 7.14 (m, 2H, Ar*H*), 7.06 (s, 1H, N*H*), 6.15 (s, 1H, N*H*), 5.83 (d, A of AB(i), *J* = 12.5 Hz, 1H, PyrCH_AH$_B$), 5.70 (d, *J* = 5.0 Hz, 1H, O*H*), 5.52 (d, B of AB(i), *J* = 12.5 Hz, 1H, PyrCH$_A$$H_B$), 5.51 (d, A of AB(ii), *J* = 13.0 Hz, 1H, PyrCH_AH$_B$), 5.46 (dd, *J* = 9.0, 2.0 Hz, 1H, NHC(*H*)RC(H)ROH), 5.36 (d, *J* = 4.5 Hz, 1H, N*H*Phe), 5.29 (d, B of AB(ii), *J* = 13.0 Hz, 1H, PyrCH$_A$$H_B$), 5.09 (d, A of AB(iii), *J* = 12.0 Hz, 1H, PhCH_AH$_B$O), 5.02 (d, B of AB(iii), *J* = 12.0 Hz, 1H, PhCH$_A$$H_B$O), 4.85 (dd, *J* = 5.0, 2.0 Hz, 1H, NHC(H)RC(*H*)ROH), 3.99 (m, X of ABX, 1H, Phe-α-*H*), 3.11 (dd, A of ABX, *J* = 14.0, 7.5 Hz, 1H, PhCH_AH$_B$C(H)R$_2$), 2.98 (dd, B of ABX, *J* = 13.5, 8.0 Hz, 1H, PhCH$_A$$H_B$C(H)R$_2$), 1.57 (s, 3H, C$H_3$), 1.56 (s, 3H, C$H_3$), 1.48 (s, 3H, C$H_3$), 1.47 (s, 3H, C$H_3$), 1.45 (s, 3H, C$H_3$), 1.34 (s, 3H, C$H_3$), 1.26 (s, 3H, C$H_3$), 1.20 (s, 3H, C$H_3$) ppm; **^{13}C NMR** (100 MHz, $CDCl_3$) δ_C 175.2, 174.3, 173.9, 173.4, 171.8, 170.4, 168.7, 156.5, 135.5, 131.3, 131.1, 130.9, 130.8, 130.5, 130.4, 129.1, 129.0, 128.8, 128.7, 128.6, 128.3, 128.1, 128.0, 127.9, 127.6, 127.5, 127.4, 127.2, 127.1, 126.1, 125.62, 125.55, 125.3, 125.1, 125.05, 124.99, 124.9, 124.8, 124.5, 124.4, 124.3, 124.2, 124.1, 124.0, 122.7, 122.0, 121.7, 121.2, 74.3, 72.4, 67.5, 65.3, 64.9, 57.6, 57.1, 57.0, 56.8, 56.7, 56.6, 27.3, 26.8, 26.5, 26.3, 24.5, 23.2, 23.1 ppm; one CH_3 resonance was not resolved; **IR** ν_{max} = 3304, 2986, 2929, 1747, 1711, 1657, 1531 cm^{-1}; **MS** (ES^+, MeCN) *m/z* = 1216 ($[M+NH_4]^+$, 100%).

Synthesis of Cbz-D-PheAib$_4$(2*R*,3*R*-BisPyrSucc)OH 23

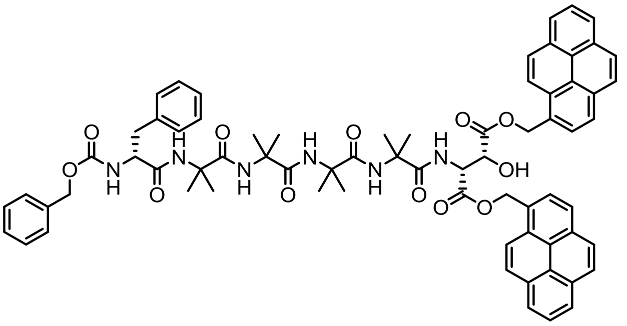

From a solution of Cbz-L-PheAib$_4$-OH **7** (53 mg, 0.083 mmol), HOBt (13 mg, 0.099 mmol), EDC (18 µL, 0.099 mmol), N_3(2*R*,3*R*-BisPyrSucc)OH **19** (50 mg, 0.083 mmol), PEt_3 (166 µL of a 1.0 M solution in THF, 0.166 mmol) in dry THF (6 mL), following general procedure 5 and after purification by silica gel column chromatography (EtOAc:CH_2Cl_2 0:100 → 30:70) the title compound **23** was obtained as a pale yellow amorphous solid (16 mg, 0.013 mmol, 16%). **TLC** SiO_2/EtOAc:CH_2Cl_2 (30:70) R_f = 0.18; **[α]$_D$**^{25} = -2.8 (*c* = 1.0, $CHCl_3$); **^{1}H NMR** (400 MHz, $CDCl_3$) δ_H 8.02 – 7.57 (m, 20H, Ar*H* + 2 × N*H*),7.53 (s, 1H, N*H*), 7.35 – 7.28 (m, 8H, Ar*H*), 7.20 – 7.18 (m, 2H, Ar*H*), 7.05 (s, 1H, N*H*), 6.28 (s, 1H, N*H*), 5.83 (d, A of AB(i), *J* = 12.5 Hz, 1H, PyrC*H$_A$*H$_B$), 5.73 (d, *J* = 5.0 Hz, 1H, O*H*), 5.52 (d, B of AB(i), *J* = 12.5 Hz, 1H, PyrCH$_A$*H$_B$*), 5.51 (d, A of AB(ii), *J* = 13.0 Hz, 1H, PyrC*H$_A$*H$_B$), 5.48 (dd, *J* = 9.5, 2.0 Hz, 1H, NHC(*H*)RC(H)ROH), 5.28 (d, B of AB(ii), *J* = 13.0 Hz, 1H, PyrCH$_A$*H$_B$*), 5.26 (d, *J* = 3.5 Hz, 1H, N*H*Phe), 5.14 (d, A of AB(iii), *J* = 12.0 Hz, 1H, PhC*H$_A$*H$_B$O), 4.98 (d, B of AB(iii), *J* = 12.0 Hz, 1H, PhCH$_A$*H$_B$*O), 4.87 (dd, *J* = 5.0, 2.5 Hz, 1H, NHC(H)RC(*H*)ROH), 4.16 (m, X of ABX, 1H, Phe-α-*H*), 3.16 (dd, A of ABX, *J* = 14.0, 5.5 Hz, 1H, PhC*H$_A$*H$_B$C(H)R$_2$), 2.93 (dd, B of ABX, *J* = 14.0, 8.5 Hz, 1H, PhCH$_A$*H$_B$*C(H)R$_2$), 1.58 (s, 3H, C*H$_3$*), 1.57 (s, 3H, C*H$_3$*), 1.50 (s, 3H, C*H$_3$*), 1.48 (s, 3H, C*H$_3$*), 1.45 (s, 3H, C*H$_3$*), 1.40 (s, 3H, C*H$_3$*), 1.35 (s, 3H, C*H$_3$*), 1.11 (s, 3H, C*H$_3$*) ppm; **^{13}C NMR** (100 MHz, $CDCl_3$) δ_C 175.6, 175.2, 174.4, 173.3, 171.3, 170.0, 168.7, 156.9, 135.6, 135.1, 131.3, 130.9, 130.8, 130.4, 129.1, 129.0 (2C), 128.9 (2C), 128.7 (2C), 128.3, 128.2 (2C), 128.0, 127.95, 127.9, 127.7, 127.6, 127.4, 127.2, 127.1 (2C), 126.1, 125.6, 125.5, 125.3, 125.1, 125.03, 124.98, 124.9, 124.4, 124.3, 124.24, 124.2, 124.1, 122.6, 122.0, 72.3, 67.8, 65.3, 64.9, 58.5, 57.7, 57.2, 57.0, 56.7, 56.6, 56.5, 27.3, 26.9, 26.4, 23.4, 23.2, 23.1, 20.8, 18.4 ppm; **IR** ν_{max} = 3303, 3029, 2985, 2928, 1750, 1705, 1657, 1531 cm^{-1}; **MS** (ES$^+$, MeCN) *m/z* = 1216 ([M+NH$_4$]$^+$, 100%).

Synthesis of *p*-(1-pyrenylether) benzaldehyde 25

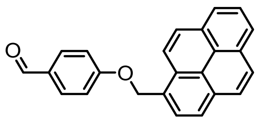

To a solution of 4-hydroxylbenzaldehyde (1.0 g, 8.2 mmol) and **1** (2.4 g, 8.2 mmol) in MeCN (30 mL) was added K_2CO_3 (2.3 g, 16 mmol) and the reaction mixture was refluxed for 16 h. After this time, the excess solvent was removed under reduced pressure and the resulting residue dissolved in EtOAc (50 mL) and washed with 1 M NaOH (2 × 100 mL) and then water (100 mL). The organic layer was dried over $MgSO_4$ and concentrated down under reduced pressure and the desired product was isolated as white solid after recrystallisation from dichloromethane and ether. **m.p.** 187-188 ºC**;** ^{1}H NMR (300 MHz, $CDCl_3$): δ 9.92 (1H, s, C(O)*H*), 8.28 -8.17 (5H, m, Ar*H*), 8.12 – 8.03 (4H, Ar*H*), 7.88 (2H, d, J = 8.8 Hz, Ar*H*), 7.21 (2H, d, *J* = 8.8 Hz, Ar*H*), 5.84 (2H, s, C*H*$_2$); ^{13}C-NMR (75 MHz, $CDCl_3$) δ_C 190.8, 163.9, 132.1, 131.8, 131.2, 130.7, 130.3, 129.3, 128.6, 128.3, 127.9, 127.4, 126.8, 126.2, 125.7, 125.6, 125.0, 124.7, 124.6, 122.7, 115.3, 69.0; **IR** ν_{max}(film)/cm^{-1} = 3041, 2926, 2737, 1688, 1597, 1576, 1507, 1312, 1214, 1244, 1157, 994, 843, 829, 756, 723; MS (ES$^+$, MeOH) *m/z* = 335 ([M-H]$^-$ 100 %).

Synthesis of (1*S*,2*S*)-1,2-bis(4-(pyren-1-ylmethoxy)phenyl)ethane-1,2-diamine.2HCl 28 (*S*,*S*-BisPhenPyrEt)

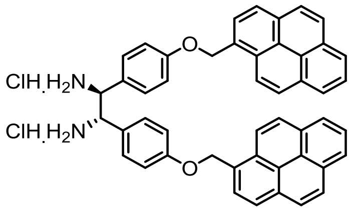

(1*S*,2*S*)-1,2-Bis(4-(pyren-1-ylmethoxy)phenyl)ethane-1,2-diamine dihydrochloride was synthesised following a modification of Method B reported by Chin and co-workers.[7] *p*-(1-pyrenylether) benzaldehyde **25** (144.5 mg, 0.43 mmol, 2.1 eq) was added to a solution of (1*R*,2*R*)-1,2-Bis(2-hydroxyphenyl)ethylenediamine **26** (50 mg, 0.21 mmol, 1 eq) in 4.25 mL of DMSO. After stirring for 24 h the mixture was poured into 12.5 mL of rapidly stirred distilled water, leading to the precipitation of a yellow solid. DCM (10 mL) was added to the mixture until the precipitate had dissolved in the organic layer which was collected. The aqueous layer was further extracted with additional DCM (2 × 10 mL). The organic layers were combined, washed with water (2×10 mL), brine (10 mL) and dried ($MgSO_4$). Removal of DCM under reduced pressure gave a 272 mg of a yellow solid. The yellow solid was dissolved in 150 mL of THF, which was concentrated to 25 mL. Insoluble impurities were removed with a cotton wool plug. 4 drops of conc. HCl were added with stirring to the solution, leading to the formation of a precipitate. The mixture was left stirring for a further 3 h. The precipitate was collected, washed with THF and dried under reduced pressure to give the title compound (91 mg, 60%) as an orange solid. **mp** decomposes > 226 °C; **m.p.** 226-228 ºC (decomposed)**;** ^{1}H NMR (400 MHz, DMSO-d_6): δ 9.03 (s, br, 6H, NH_3), 8.35 – 8.19 (m, 16H, Ar*H*, Pyr), 8.10 – 8.06 (m, 2H, Ar*H*, Pyr), 7.31 (d, J = 8.4 Hz, 2H, Ar*H*), 7.11 (d, J = 8.4 Hz, 2H, Ar*H*), 5.80 (s, 4H, OCH_2), 4.97 (s, 2H, C*H*); ^{13}C NMR (75 MHz, DMSO-d_6): δ_C 158.7, 130.8, 130.6, 130.1(2C), 129.9 (2C), 127.8, 127.5(2C), 127.2, 126.3, 125.5, 125.4, 124.6, 123.7, 123.2, 114.9, 67.0, 25.0 ppm; **IR** ν_{max}(film)/cm^{-1} = 3434, 2250, 2124, 1624, 1053, 1025, 1006, 820, 758; MS (ES$^+$, MeOH) *m/z* = 697 ([M+Na]$^+$ 100%; HRMS (ES$^+$,MeOH) %); Calcd for $C_{48}H_{38}N_2O_2Na$ = 697.2843, found 697.2826.

Synthesis of Cbz(L-Phe)Aib$_4$(*S,S*-BisPyrEt)NH$_2$ 29

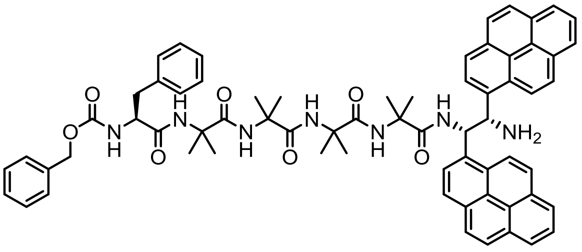

Cbz-L-Phe-Aib$_4$-OH **7** (43.7 mg 0.068 mmol, 1 eq) was coupled to (1*S*,2*S*)-1,2-bis(1-pyrene)ethylenediamine dihydrochloride **27** (40.0 mg, 1.1 eq) using General procedure 1. The resulting residue was purified by crystallisation (DCM/Et_2O) to give 37.3 mg (50%) of the title compound as an off-white solid. **mp** 182-185 °C; **$[\alpha]_D^{24}$** = +234.3 (c = 1.0, $CHCl_3$); **IR** (ATR, cm^{-1}) 3305, 3040, 2962, 2928, 1652,1526; **^{1}H NMR** (500 MHz, $CDCl_3$) δ_H 8.60 (m, 2H, PyrH), 8.50 (d, *J* = 7.3, 1H, PyrH), 8.40 (d, *J* = 8.8, 1H, C(O)N*H*CH-Pyr), 8.27 (d, *J* = 5.4, 1H, PyrH), 8.07 - 7.95 (m, 5H, PyrH), 7.90 - 7.81 (m, 7H, PyrH), 7.81 - 7.75 (m, 2H, PyrH), 7.71 (m, 1H, NH), 7.66 (s, 1H, NH), 7.38 - 7.29 (m, 5H, ArH+ NH), 7.26 - 7.14 (m, 6H, ArH), 6.94 - 6.79 (m, 2H, NH + C(O)NHC*H*-Pyr), 6.30 (br. s., 1H, NH, Phe), 6.00 (d, *J* = 8.5, 1H, NH_2CH-Pyr), 5.19 (d, *J* = 12.5, A of AB, 1H, PhC$H_A$$H_B$O) 5.03 (d, *J* = 12.5, 1H, B of AB, 1H, PhCH$_A$$H_B$O Cbz), 4.23 - 4.15 (m, X of ABX, 1H $^{\alpha}$C-Phe), 3.13 (dd, A of ABX, *J* = 14.2, 5.4, 1H, $^{\beta}$CH-Phe), 2.96 (dd, B of ABX, *J* = 14.2, 8.8, 1H, $^{\beta}$CH-Phe), 1.73 (s, 3H, CH_3), 1.68 (s, 3H, CH_3), 1.60 (s, 3H, CH_3), 1.52 (s, 3H, CH_3), 1.43 (s, 3H, CH_3), 1.35 (s, 3H, CH_3) 1.31 (s, 3H, CH_3), 1.09 (s, 3H, CH_3) ppm; **^{13}C NMR** (125 MHz, $CDCl_3$): 175.5, 175.2, 175.0, 173.8, 171.9, 157.1, 136.4, 136.2, 135.8, 135.2, 132.4, 131.1, 131.0, 130.9, 130.6, 130.5, 130.0, 129.9, 129.0, 128.8, 128.6, 128.52, 128.47, 128.4, 127.9, 127.3, 127.2, 127.1, 127.1, 126.6, 126.6, 125.6, 125.41, 125.36, 124.9, 124.7, 124.7, 124.6, 124.51, 124.47, 124.4, 123.2, 122.9, 77.6, 77.2, 67.3, 57.7, 57.2, 57.0, 56.8, 56.5, 36.4, 26.9, 26.6, 26.3, 25.9, 23.7, 23.5, 23.8, 23.0 ppm; two aromatic resonances were not observed; **MS** (ES$^+$) 1082 (100%, $[M+H]^+$); **HRMS** (ESI$^+$ ORBITRAP) calcd for $C_{67}H_{68}N_7O_7$ 1082.5175 found 1082.5162.

Synthesis of Cbz(D-Phe)Aib$_4$(*S*,*S*-BisPyrEt)NH$_2$ 30

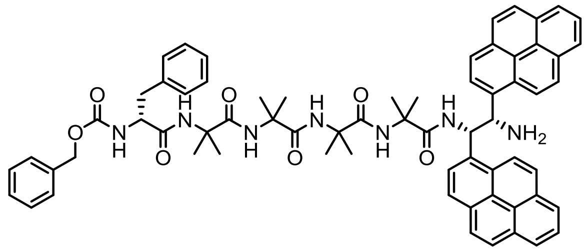

Cbz-D-Phe-Aib$_4$-OH (43.7 mg 0.068 mmol, 1 eq.) was coupled to (1*S*,2*S*)-1,2-bis(1-pyrene)ethylenediamine dihydrochloride **27** (40.0 mg 1.1 eq.) using General procedure 1. The resulting residue was purified by crystallisation (DCM/Et_2O) to give 40.6 mg (55%) of the title compound as an off-white solid. **mp** 173-176 °C; **$[\alpha]_D^{24}$** = +333.8 (c = 1.1, $CHCl_3$) **IR** (ATR, cm^{-1}) 3295, 3038, 2981,2963, 1651, 1528; **^{1}H NMR** (500 MHz, $CDCl_3$): δ_H 8.58 (d, *J* = 9.5, 1H, PyrH), 8.50 (d, *J* = 6.5, 1H, PyrH), 8.46 - 8.35 (m, 2H, 2 × PyrH), 8.31 (d, *J* = 8.6, 1H, C(O)N*H*CH-Pyr), 8.07 (br. s, 1H, NH), 8.02 (d, *J* = 7.5, 1H, PyrH), 7.98 (d, *J* = 7.4, 1H, PyrH), 7.96 - 7.73 (m, 12H, 9 × PyrH, NH, *NH_2*CHPyr), 7.74 – 7.69 (m, 4H, 3 × PyrH, NH), 7.69 - 7.63 (m, 3H, 2×ArH + NH), 7.62 (s, 1H, NH), 7.52 - 7.45 (m, 2H, 2 × ArH), 7.45 - 7.38 (m, 1H, ArH), 7.20 (d, *J* = 7.5, 2H, 2 × ArH), 7.03 (t, *J* = 7.4, 2H, 2 × ArH), 6.93 - 6.84 (m, 2H, ArH + C(O)NHC*H*-Pyr), 6.15 (d, *J* = 8.3, 1H, NH_2C*H*Pyr), 5.56 (d, *J* = 13.0, 1H, A of AB, PhC$H_A H_B$O), 5.39 (d, *J* = 13.1 Hz, 1H, B of AB, PhC$H_A H_B$O), 4.35 - 4.28 (m, 1H, $^{\alpha}$CH-Phe), 3.35 (d, *J* = 10.0, 1H, $^{\beta}$CH-Phe), 3.13 (t, *J* = 12.2, 1H $^{\beta}$CH-Phe), 1.79 (s, 3H, CH_3), 1.67 (s, 3H, CH_3), 1.66 (s, 3H, CH_3), 1.61 (s, 6H, 2×CH_3), 1.47 (s, 3H, CH_3), 1.36 (s, 3H, CH_3), 1.35 (s, 3H, CH_3); **^{13}C NMR** (125 MHz, $CDCl_3$): 176.4, 175.6, 175.1, 175.0, 172.6, 157.0, 138.4, 137.6, 135.6, 134.8, 131.04, 130.96, 130.44, 130.41, 130.0, 129.9, 129.7, 128.8 (x2), 128.7, 128.5, 128.2, 128.0, 127.3, 127.1 (x2), 126.9, 126.8, 126.7, 126.3, 125.4, 125.3, 124.7 (x2), 124.62 (x2), 124.57 (x2), 124.5 (x2), 124.4 (x2), 123.1, 122.6, 77.1, 66.6 (x2), 57.1, 57.0 (x2), 56.7, 56.6, 35.2, 27.7, 27.4, 27.1, 26.9, 23.7, 23.2, 23.1, 22.5 ppm; two aromatic resonances were not observed; **MS** (ES$^+$, MeOH): 1083 (100%, $[M+H]^+$); **HRMS** (ES$^+$, MeOH) *m/z* calcd. for $C_{67}H_{68}N_7O_7$ $[M+H]^+$ = 1082.5175, found 1082.5155.

Synthesis of Cbz(L-Phe)Aib$_4$(*S,S*-BisPhenPyrEt)NH$_2$ 31

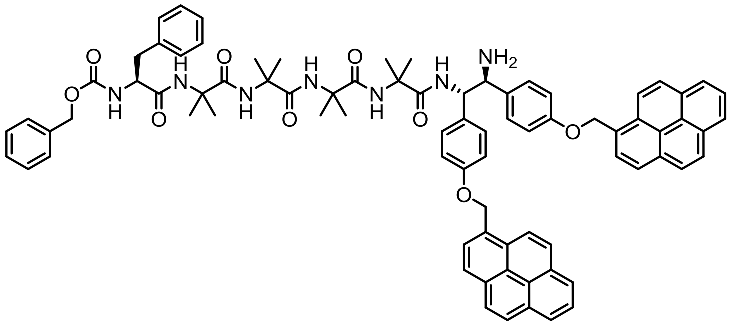

Cbz-L-Phe-Aib$_4$-OH (30.0 mg, 0.047 mmol, 1 eq) was coupled to (1S,2S)-1,2-bis(4-(pyren-1-ylmethoxy)phenyl)ethane-1,2-diamine dihydrochloride **28** (38.5 mg, 1.1 eq) using General procedure 1. The resulting residue was purified by column chromatography (1-4% MeOH/DCM) to give 43 mg (71%) of the title compound as a white solid. mp 138-140 °C; **[α]$_D$^{24}** = -31.2 (c=1 in $CHCl_3$); IR (ATR, cm^{-1}) 3039, 2983, 2926, 2854, 2471, 1646, 1513, 1420, 1236; **^{1}H NMR** (500 MHz, CD_3OD/10% $CDCl_3$) δ_H 8.14 – 8.03 (m, 8H, 8 × PyrH), 8.02 – 7.94 (m, 7H, 7 × PyrH), 7.90 (d, *J* = 4.1, 1H, PyrH), 7.88 (d, *J* = 4.0, 1H, PyrH), 7.87 (d, *J* = 4.0, 1H, PyrH), 7.34 – 7.30 (m, 4H, ArH), 7.29-7.21 (m, 10H, 6 × ArH, *m*-ArCH, Phe-O-, C*H*NH$_2$, C*H*CHNH$_2$), 7.18 (d, *J* = 8.6, 2H, *m*-ArCH, Ph-O-), 6.99 (d, *J* = 8.7, 2H, *o*-ArCH, Ph′-O-), 6.83 (d, *J* = 8.6, 2H, *o*-ArCH, Ph′-O-), 5.64 (d, *J* = 11.9, 1H, A of AB, OC*H*$_A$H$_B$-Pyr), 5.60 (d, *J* = 11.9, 1H, B of AB, OCH$_A$*H*$_B$-Pyr), 5.56 (d, *J* = 11.7, 1H, A' of AB', OC*H*$_A$H$_B$'-Pyr), 5.50 (d, *J* = 11.7, 1H, B' of AB', CH$_A$*H*$_B$'-Pyr), 5.14 (d, *J* = 12.6, 1H, A'' of AB'', PhC*H*$_A$H$_B$O), 5.07 (d, *J* = 12.6, 1H, B'' of AB'', PhCH$_A$*H*$_B$O), 4.22 (t, *J* = 7.6, 1H, X of ABX, ^{β}C*H*$_A$H$_B$-Phe), 3.06 (dd, *J* = 13.7, 7.6, 1H, A of ABX, ^{β}CH$_A$*H*$_B$-Phe), 2.98 (dd, *J* = 13.7, 7.6, 1H, B of ABX, ^{β}CH$_A$*H*$_B$, Phe), 1.50 (s, 3H, CH_3), 1.48 (s, 3H, CH_3), 1.45 (s, 3H, CH_3), 1.38 (s, 3H, CH_3), 1.38 (s, 3H, CH_3), 1.32 (s, 3H, CH_3), 1.27 (s, 3H, CH_3), 1.26 (s, 3H, CH_3). **^{13}C NMR** (125 MHz, CD_3OD/10% $CDCl_3$) δ_C 178.6, 177.9, 176.5, 176.4, 174.0, 172.8, 160.5, 159.7, 158.5, 155.4, 154.9, 151.9, 137.8, 137.6, 132.7, 132.3, 131.8, 130.9 130.7, 130.3, 130.0, 129.4, 129.0, 128.9, 128.4, 128.2, 127.9, 127.0, 126.6, 126.3, 125.8, 125.4, 123.8, 116.6, 115.9, 69.7, 69.5, 67.7, 61.7, 58.7, 58.2, 57.6, 57.5, 57.4, 57.3, 38.0, 30.6, 27.0, 26.7, 25.8, 24.5, 24.1, 23.6, 23.3; nineteen aromatic resonances were not observed; **MS** (ES+, MeOH): 1295 (100%, [M+Na]$^+$).

7.25 – 7.11 (m, 14H, C*H*CHNH$_2$, C*H*NH$_2$, 9×ArH, *m*-ArCH, Phe-O-),

Synthesis of Cbz(D-Phe)Aib$_4$(*S*,*S*-BisPhenPyrEt)NH$_2$ 32

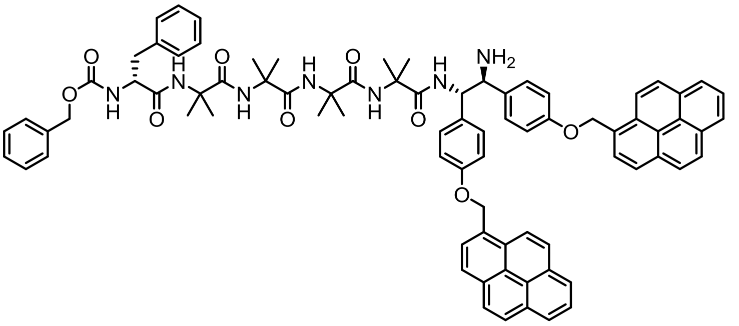

Cbz-D-Phe-Aib$_4$-OH (50 mg 0.078 mmol, 1 eq) was coupled to (1S,2S)-1,2-bis(4-(pyren-1-ylmethoxy)phenyl)ethane-1,2-diamine dihydrochloride **28** (63.0 mg 1.1 eq) using General procedure 1. The resulting residue was purified by column chromatography (1-4% MeOH/DCM) to give 74.8 mg (74%) of the title compound as a white solid. **mp** 151-153 °C; **[α]$_D$** = -61.2 (c=1; CHCl$_3$); **^{1}H NMR** (400 MHz, CD$_3$OD/ 10% CDCl$_3$) δ_H 8.01 – 7.91 (m, 8H, PyrH), 7.89 – 7.75 (m, 11H, 10 × PyrH, NH), 7.25 – 7.11 (m, 14H, C*H*CHNH$_2$, C*H*NH$_2$, 9×ArH, *m*-ArCH, Phe-O-), 7.09 (d, *J* = 8.7, 2H, CH$_2$ *m*-ArCH, Phe′-O-), 6.88 (d, *J* = 8.7, 2H, CH$_2$ *o*-ArCH, Phe′-O-), 6.73 (d, *J* = 8.7, 2H, CH$_2$*o*-ArCH, Phe-O-), 5.50 (d, *J* = 12.2, 1H, A of AB, OC*H*$_A$H$_B$-Pyr), 5.45 (d, *J* = 12.2, 1H, B of AB, OCH$_A$*H*$_B$-Pyr), 5.41 (d, *J* = 11.8, 1H, A' of AB', OC*H*$_a$'H$_b$-Pyr), 5.35 (d, *J* = 11.8, 1H, B' of AB', OCH$_a$'*H*$_b$-Pyr), 5.04 (d, *J* = 12.6, 1H, A'' of AB'', PhC*H*$_A$H$_B$O), 4.98 (d, *J* = 12.6, 1H, B'' of AB'', PhCH$_A$*H*$_B$O), 4.12 (t, *J* = 7.5, 1H, X of ABX, $^\alpha$CH, Phe), 2.96 (dd, *J* = 14.0, 7.5, 1H, A of ABX, $^\beta$C*H*$_A$H$_B$-Phe), 2.88 (dd, *J* = 14.0, 8.3, 1H, A of ABX, $^\beta$C*H*$_A$H$_B$-Phe), 1.41 (s, 3H, CH$_3$), 1.39 (s, 3H, CH$_3$), 1.36 (s, 3H, CH$_3$), 1.30 (s, 3H, CH$_3$), 1.29 (s, 3H, CH$_3$), 1.22 (s, 3H, CH$_3$), 1.18 (s, 3H, CH$_3$), 1.16 (s, 3H, CH$_3$). **^{13}C NMR** (125 MHz, CD$_3$OD) δ_C 176.3, 175.2, 174.9, 172.7, 157.5, 156.8, 138.5, 137.3, 134.2, 133.4, 131.4, 131.1, 130.6, 129.8, 129.2, 129.1, 129.0, 128.6, 128.2, 127.9, 127.5, 127.3, 127.1, 126.8, 126.7, 126.3, 125.9, 125.2, 124.8, 124.5, 124.5, 123.0, 114.1, 68.5, 66.4, 60.6, 60.3, 57.0, 56.9, 56.8, 56.7, 56.2, 35.2, 29.7, 27.8, 27.3, 26.9, 23.8, 23.1, 23.0, 22.5; twenty-one aromatic resonances and one CH$_2$ resonance were obscured or not observed**;** **MS** (ES$^+$, MeOH): 1295 (100%, [M+Na]$^+$).

Synthesis of N_3Aib$_4$(*S*,*S*-BisPyrEt)NHAc 43

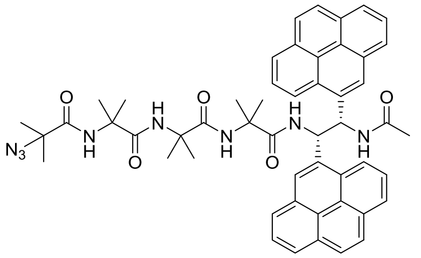

N_3Aib$_4$OH (30.0 mg, 0.078 mmol, 1 eq) was coupled to (1*S*,2*S*)-1,2-bis(1-pyrene)ethylenediamine dihydrochloride **27** (47.1 mg, 0.085 mmol, 1.1 eq) using General Procedure 4. The resulting residue was purified by HPLC (Eclipse XD8-C18, 5 µm, 9.4 × 250 mm, 45-75% MeCN:H_2O) to give 48 mg, 71% of the title compound as an off-white solid. **$[\alpha]_D$** = -60 (C= 1.16; $CHCl_3$)**;** **IR** (ATR, cm^{-1}): 3341, 2111, 1659, 1642, 1521, 1508, 1259, 843; **^{1}H NMR** (400 MHz, $CDCl_3$): δ_H 8.64 (d, *J* = 9.4, 1H, CO**NH**CHPyr), 8.60 (d, *J* = 8.1, 1H, CHPyr**NH**OAc), 8.27 (d, J = 8.8, 1H, PyrH), 8.24 (d, J = 9.5, 1H, PyrH), 8.12 (d, *J* = 9.2, 1H, PyrH), 8.08 – 7.98 (m, 7H, PyrH × 7), 7.92-7.83 (m, 7H, PyrH × 7), 7.61 (d, *J* = 6.9, 1H, PyrH), 6.99 (t, *J* = 8.4, 1H, NH**CH**Pyr), 6.82 (t, 1H, **CH**PyrNHOAc), 6.77 (s, 1H, NH), 6.19 (s, 1H, NH), 2.12 (s, 3H, $COCH_3$), 1.63 (s, 3H, CH_3), 1.57 (s, 3H, CH_3), 1.54 (s, 6H, CH_3 × 2), 1.38 (s, 3H, CH_3), 1.29 (s, 3H, CH_3), 1.23 (s, 3H, CH_3), 1.19 (s, 3H, CH_3) ppm; **^{13}C NMR** (101 MHz, $CDCl_3$): 175.1, 173.4, 173.0, 172.9, 170.4, 134.7, 133.5, 132.7, 131.2, 131.0, 130.7, 130.6, 130.5, 130.4, 128.7, 128.4, 127.6, 127.37, 127.37, 127.2, 127.1, 126.8, 125.6, 125.5, 125.2, 124.9, 124.8 (2C), 124.7 (2C), 124.65 (2C), 124.63, 124.58, 123.2, 122.8, 66.3, 58.4, 57.7, 57.0, 56.9, 29.7, 26.7, 25.7, 24.3, 24.19, 24.16, 23.9, 23.8, 23.5 ppm; one aromatic resonance and one CH were not resolved**;** **MS** (ES^+, MeOH) 867.4 (100%, $[M-H]^+$); **HRMS** (EMR Orbitrab +ive, MeOH) *m/z* calcd. for $C_{52}H_{52}O_5N_8K$ ($[M+K]^+$) = 907.3697 found 907.3681.

Synthesis of Cbz(D-Phe)Aib$_4$(*S,S*-BisPyrEt)NHAc 44

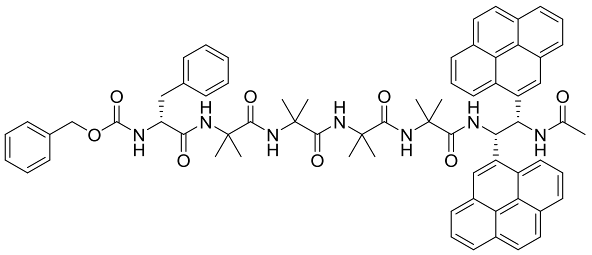

Cbz-D-PheAib$_4$(*S,S*-BisPyrEt)NH$_2$ **8** (61 mg, 0.058 mmol, 1 eq) was acetylated using General Procedure 3. The crude residue was purified by HPLC (Eclipse XD8-C18, 5 µm, 9.4 × 250 mm, 65-95% MeCN:H_2O) to give 46.8 mg, 72% of the title compound as an off-white solid. **[α]$_D$** = +10 (C= 1, $CHCl_3$); **IR** (ATR, cm^{-1}): 3305, 3038, 2961, 2925, 1648, 1502, 1258, 1228, 1021, 843; **^{1}H NMR** (400 MHz: MeOD): δ_H 8.91 (br d, *J* = 8.5, 1H, CO, **NH**CHPyr), 8.79 (d, *J* = 8.5, 1H, CHPyr**NH**OAc), 8.60 (d, *J* = 8.0, 1H, PyrH), 8.48 (d, *J* = 9.5, 1H, PyrH), 8.34 (d, *J* = 8.1, 1H, PyrH), 8.24 (d, *J* = 9.1, 1H, PyrH), 8.16 (s, 1H, PyrH), 8.03 (dd, *J* =13.4, 8.2, 2H, PyrH × 2), 7.99 (s, 2H, NH × 2), 7.95 - 7.90 (m, 4H, PyrH × 4), 7.84 - 7.76 (m, 8H, PyrH × 7, NH), 7.42 - 7.30 (m, 10H, ArH × 10), 6.84 - 6.75 (m, 2H, NH**CH**Pyr, **CH**PyrNHOAc), 5.11 (s, 2H, CH_2(Cbz)), 4.23 (t, *J* = 7.7, 1H, $^{\alpha}$CHPhe), 3.02 (qt, *J* = 13.6, 7.8, 2H, $^{\beta}CH_2$Phe), 2.08 (s, 3H, CH_3), 1.60 (s, 3H, CH_3), 1.59 (s, 3H, CH_3), 1.56 (s, 3H, CH_3), 1.47 (s, 3H, CH_3), 1.45 (s, 3H, CH_3), 1.31 (s, 3H, CH_3), 1.22 (s, 6H, CH_3 × 2) ppm; **^{13}C NMR** (100 MHz: MeOD): δ_c 176.6, 176.0, 173.4, 175.2, 172.9, 171.4, 157.3, 137.3, 136.8, 136.6, 133.8, 133.4, 131.1, 131.0, 130.6, 130.5, 130.4, 130.3, 129.1 (2C), 128.3, 128.17 (2C), 128.15, 128.1 (2C), 127.7, 127.2, 127.0, 126.9, 126.8, 126.7, 126.5, 125.4, 125.3, 125.2, 124.69, 124.65, 124.46, 124.44, 124.39, 124.3, 124.26, 124.2, 122.2, 122.0, 66.3, 57.2, 57.1, 56.8, 56.6, 56.3, 53.8, 53.2, 36.4, 25.5, 25.2 (2C), 24.7, 23.5, 23.2, 22.5 (2C), 21.7 ppm; one aromatic resonance was not resolved; **MS** (ES$^+$, MeOH) 1146.6 (100%, [M+Na]$^+$); **HRMS** (ES$^+$, MeOH) *m/z* calcd. for $C_{69}H_{69}O_8N_7$ ([M+H]$^-$) = 1124.5288 found 1124.5142.

Synthesis of (1*R*,2*R*)-1,2-(1-pyrene)ethylenediamine dihydrochloride 46

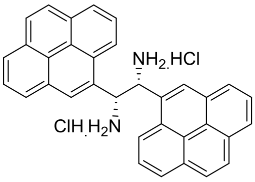

2,2'-((*1S,2S*)-1,2-Diaminoethane-1,2-diyl)diphenol (100 mg, 0.41 mmol) and 1-pyrenecarboxaldehyde (0.82 mmol) was stirred at ambient temperature in DMSO for 24h. The reaction mixture was poured into 60.0 mL of rapidly stirred distilled water and lead to the precipitation of a yellow solid. The yellow solid was collected, washed with distilled water and dried under vacuum for 1h. The solid was re-dissolved in THF and conc. HCl (0.10 mL) was added dropwise to the stirred solution. The mixture was stirred for 3 h and the product precipitated out of solution as an air sensitive pale yellow solid. The precipitate was collected by vacuum filtration and dried under vacuum for 1 h before subsequent use (78% 173 mg). **$[\alpha]_D$** = -21 (C= 0.5, DMSO). **IR** (ATR, cm^{-1}): 2781, 2590, 1588, 1516, 846, 632. **^{1}H NMR** (400 MHz, DMSO-d_6): δ_H 9.69 (br s, 6H, NH_3Cl × 2), 8.78 (2H, d, *J = 9*.3, PyrH × 2), 8.68 (2H, d, *J* = 7.6, PyrH × 2), 8.39 (2H, d, *J* = 9.4, PyrH × 2), 8.32 (2H, d, *J* = 7.6, PyrH × 2), 8.19 (2H, d, *J* = 7.4, PyrH × 2), 8.04 (2H, t, *J* = 7.6, PyrH × 2), 7.93 (4H, d, *J = 15.6, 8.6*, PyrH ×2), 7.80 (2H, d, *J* = 9.0, PyrH ×2), 6.91 (br s, 2H, CH ×2) ppm. **^{13}C NMR** (100 MHz, DMSO-d_6): δc 130.9, 130.4, 129.7, 128.9, 128.8, 128.7, 128.2, 127.3, 127.0, 126.4, 126.0, 125.6, 125.0, 123.7, 123.4, 122.9, 52.8 ppm. **MS** (ES^+, MeOH) 462 (70%, $[M-(HCl)_2+2H]^+$). **HRMS** (ES^+, MeOH) *m/z* calcd. for $C_{34}H_{24}N_2$ $[M-(HCl)_2+H]^+$) = 461.2012, found 461.2001.

Synthesis of N_3Aib_4(*R*,*R*-BisPyrEt)NHAc 47

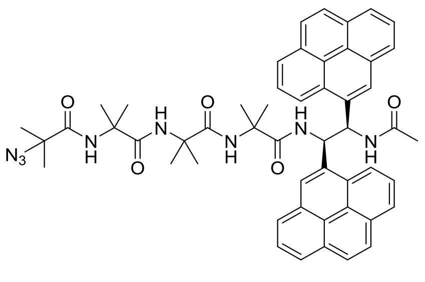

N_3Aib_4OH (30.0 mg, 0.078 mmol, 1 eq) was coupled to (*1R,2R*)-1,2-bis(4-(pyren-1-ylmethoxy)phenyl)ethane-1,2-diamine dihydrochloride **50** (47.1 mg, 0.085 mmol, 1.1 eq) using General procedure 4. The resulting residue was purified by HPLC (Eclipse XD8-C18, 5 µm, 9.4 × 250 mm, 45-75% MeCN:H_2O) to give 46.3 mg, 68% of the title compound as an off-white solid. Spectroscopic data matches **43** except the **$[\alpha]_D$** = 7.8 (C =1.2, MeOH). **^{1}H NMR** (400 MHz, $CDCl_3$): δ_H 8.64 (d, *J* = 9.4, 1H, CO**NH**CHPyr), 8.60 (d, *J* = 8.1, 1H, CHPyr**NH**OAc), 8.27 (d, J = 8.8, 1H, PyrH), 8.24 (d, J = 9.5, 1H, PyrH), 8.12 (d, *J* = 9.2, 1H, PyrH), 8.08 – 7.98 (m, 7H, PyrH × 7), 7.92-7.83 (m, 7H, PyrH × 7), 7.61 (d, *J* = 6.9, 1H, PyrH), 6.99 (t, *J* = 8.4, 1H, NH**CH**Pyr), 6.82 (t, 1H, **CH**PyrNHOAc), 6.77 (s, 1H, NH), 6.19 (s, 1H, NH), 2.12 (s, 3H, $COCH_3$), 1.63 (s, 3H, CH_3), 1.57 (s, 3H, CH_3), 1.54 (s, 6H, CH_3 × 2), 1.38 (s, 3H, CH_3), 1.29 (s, 3H, CH_3), 1.23 (s, 3H, CH_3), 1.19 (s, 3H, CH_3) ppm; **^{13}C NMR** (101 MHz, $CDCl_3$): 175.2, 173.3, 173.0, 172.9, 170.4, 134.8, 133.5, 131.2, 131.0, 130.7, 130.6, 130.5, 130.4, 128.7, 128.4, 127.6, 127.38, 127.35, 127.2, 127.1, 126.8, 125.6, 125.5, 125.2, 124.9, 124.8 (2C), 124.7 (2C), 124.65, 124.63 (2C), 124.58, 123.2, 122.8, 63.8, 57.3, 57.0, 57.0, 56.9, 29.7, 26.8, 25.7, 24.3, 24.19, 24.16, 23.9, 23.8, 23.5 ppm**;** two aromatic resonances were not resolved. **MS** (ES$^+$, MeOH) 867.4 (100%, $[M-H]^+$); **HRMS** (EMR Orbitrab +ive, MeOH) *m/z* calcd. for $C_{52}H_{52}O_5N_8K$ ($[M+K]^+$) = 907.3697 found 907.3692.

Synthesis of Cbz-D-PheAib$_4$(*R,R*-BisPyrEt)NHAc 48

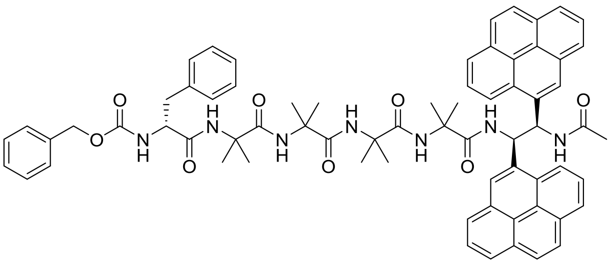

Cbz-D-PheAib$_4$OH (42.2 mg, 0.066 mmol, 1 eq) was coupled to (*1R,2R*)-1,2-bis(4-(pyren-1-ylmethoxy)phenyl)ethane-1,2-diamine dihydrochloride **50** (38.9 mg, 0.073 mmol, 1.1 eq) using General Procedure 4. The crude residue was purified by HPLC (Eclipse XD8-C18, 5 µm, 9.4 × 250 mm, 65-95% MeCN:H_2O) to give 32 mg, 43% of the title compound as an off-white solid. **IR** (ATR, cm^{-1}): 3305, 3040, 2979, 2932, 1654, 1508, 1259, 1026, 843; **^{1}H NMR** (400 MHz, MeOD): δ_H 9.20 (br d, *J* = 9.0, 1H, CO**NH**CHPyr), 8.77 ((d, *J* = 9.0, 1H, CHPyr**NH**OAc), 8.62 (d, *J* = 9.4, 1H, PyrH), 8.55 (d, *J* = 9.4, 1H, PyrH), 8.51 (d, *J* = 8.1, 1H, PyrH), 8.41 (d, *J* = 8.0, 1H, PyrH), 8.25 (1H, s, NH), 8.14-8.06 (9H, m, PyrH × 9), 7.96-7.89 (m, 7H, PyrH × 5, NH × 2), 7.36-7.22 (m, 10H, ArH × 10), 6.86 (dd, *J* = 9.0, 5.2, 1H, **CH**PyrNHOAc), 6.76 (dd, *J* = 9.0, 5.2, 1H, NH**CH**Pyr), 5.11 (s, 2H, CH_2(Cbz)), 4.26 (t, *J* = 7.28, $^{\alpha}$CHPhe), 3.02 (dd, *J* = 30.7, 13.8, 1H, $^{\beta}CH_2$Phe, H^A of ABX system), 3.01 (dd, *J* = 30.7, 13.3, 1H, $^{\beta}CH_2$Phe, H^A of ABX system), 1.96 (3H, s, OCH_3), 1.62 (3H, s, CH_3), 1.60 (3H, s, CH_3), 1.52 (3H, s, CH_3), 1.50 (3H, s, CH_3), 1.44 (3H, s, CH_3), 1.38 (3H, s, CH_3), 1.36 (3H, s, CH_3), 1.30 (3H, s, CH_3); **^{13}C NMR** (101 MHz, MeOD): δ_C 176.9, 176.0, 175.3, 175.0, 172.9, 171.0, 157.2, 136.8, 136.6, 133.5, 133.4, 131.2, 130.6, 130.5, 129.2 (2C), 128.2 (3C), 128.12 (2C), 128.10, 128.0, 127.7, 127.4, 127.3, 127.2, 127.0, 126.9, 126.8, 126.5, 125.6, 125.5, 125.4, 124.9, 124.8, 124.7, 124.6, 124.47, 124.44, 124.4, 124.3, 124.1, 122.2, 121.9, 66.3, 57.3, 57.2, 56.88, 56.86, 56.6, 56.2, 53.6, 36.9, 29.4, 25.5, 25.2 (2C), 24.7, 23.4 (2C), 22.7, 21.4 ppm; two aromatic resonances were not observed. **MS** (ES^+, MeOH) 1146.6 (100%, $[M+H]^+$); **HRMS** (ES^+, MeOH) *m/z* calcd. for $C_{69}H_{69}O_8N_7$ ($[M-H]^-$) = 1122.5129 found 1122.5159.

Synthesis of Cbz(L-αMeVal)Aib$_4$(*R*,*R*-BisPyrEt)NHAc 49

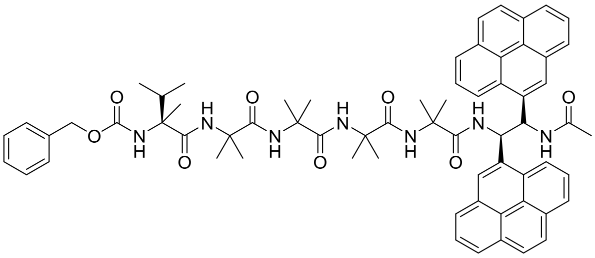

Cbz-L-Phe-Aib$_4$-OH (50 mg, 0.083 mmol, 1 eq) was coupled to (*1R,2R*)-1,2-bis(4-(pyren-1-ylmethoxy)phenyl)ethane-1,2-diamine dihydrochloride **50** (49 mg, 0.092 mmol, 1.1 eq) using General procedure 4. The resulting residue was purified by HPLC (Eclipse XD8-C18, 5 µm, 9.4 × 250 mm, 68-100% MeCN:H_2O)to give 21.6 mg, 24% of the title compound as an off white solid. **[α]$_D$** = +3.12 (C= 1, MeOH); **IR** (ATR, cm^{-1}): 3294, 3040, 2963, 2926, 1655, 1520, 1259, 1028, 843; **^{1}H NMR** (400 MHz, $CDCl_3$): δ_H 8.63 (d, *J* = 8.1, 1H, PyrH), 8.49 (d, *J* = 9.6, 1H, PyrH), 8.34 (d, *J* = 9.4, 1H, CO**NH**CHPyr), 8.30 (d, *J* = 10.7, 1H, PyrH), 8.13 (d, *J* = 9.4, 1H, PyrH), 8.01 (dd, *J* = 8.2, 4.5, 2H, PyrH), 8.03-7.71 (m, 14H, PyrH × 11, NH × 2, CHPyr**NH**OAc), 7.43 (br s, 2H, NH × 2) 7.29-7.23 (m, 5H, ArH × 5), 6.87 (br t, *J* = 8.5, 1H, **CH**PyrNHOAc), 6.7 (t, *J* = 6.7, 1H, NH**CH**Pyr), 6.20 (s, 1H, NH), 5.19 (s, 1H, NH), 5.10 (d, *J* = 12.2, 1H, ^{A}CH(Cbz)), 4.91 (d, *J* = 12.2, 1H, $^\beta$CH(Cbz)), 2.08(s, 3H, $^\alpha CH_3$, αMeVal), 1.72-1.63 (m, 1H, $^\beta$CH, αMeVal), 1.59 (s, 3H, OCH_3), 1.54 (s, 3H, CH_3), 1.47 (s, 3H, CH_3), 1.37 (s, 3H, CH_3), 1.30 (s, 3H, CH_3), 1.25 (s, 3H, CH_3), 1.20 (s, 3H, CH_3), 1.19 (s, 3H, CH_3), 0.92 (s, 3H, CH_3), 0.78 (d, *J* = 6.8, 3H, $^\beta$CH(**CH_3**) αMeVal), 0.74 (d, *J* = 6.93, 3H, $^\beta$CH(**CH_3**) αMeVal) ppm; **^{13}C NMR** (125 MHz, $CDCl_3$): δ_c: 175.7, 175.2, 174.8, 173.7, 172.6, 170.7, 156.1, 135.9, 135.2, 134.5, 131.2, 131.1, 130.7, 130.7, 130.6, 130.42, 130.48, 128.77, 128.73 (2C), 128.67, 128.3, 128.2 (2C), 127.6, 127.4, 127.3, 127.1, 126.9, 126.6, 125.5, 125.3, 125.0, 124.8, 124.72, 124.71 (2C), 124.65, 124.64, 124.5, 122.1, 122.9, 67.5, 63.0, 57.2, 57.1, 56.8, 56.5, 54.0, 52.5, 35.6, 29.7, 23.2, 22.9, 22.4, 21.9, 16.5, 16.2 ppm; one aromatic resonance was not resolved; **MS** (ES$^+$, MeOH) 1112.6 (100%, [M+H]$^+$); **HRMS** (ES$^+$, MeOH): *m/z* calcd. for $C_{66}H_{71}O_8N_7$ ([M+H]$^-$) = 1090.5442 found 1090.5306.

4. Spectroscopic studies of foldamers in organic solvents and vesicles

4.1 General proceduresfor the preparation of phospholipid vesicles

MilliQ water, HPLC grade or above solvents were used for all experiments. Lipid stock solutions were either used as purchased (Avanti, Sigma-Aldrich) or prepared from solid lipids using spectrophotometric grade chloroform. Aliquots were added using Gilson® pipettes or Hamilton syringe for corrosive solvents.

4.1.1. Preparation of 800 nm diameter egg yolk phosphatidylcholine (EYPC) large unilamellar vesicles (LUVs) containing foldamer.

LUVs were prepared by following an standard extrusion protocol.[6] Aliquots of EYPC stock solution (32.5 μL of 100 mg/mL in $CHCl_3$, prepared by dissolving 20 mg of EYPC in 200 μL of $CHCl_3$) and foldamer solution (42 to 46 μL as appropriate, 1 mg/mL, MeOH) were co-evaporated to give mixed films of 0.0042 mmol lipid with 4.2×10^{-5} mmol of foldamer. These films were placed under high vacuum for 4 h to remove residual organic solvent. The films were then re-hydrated by addition of MOPS buffer (1.2 mL, 20 mM MOPS, pH = 7.3) followed by vortex mixing to give a homogeneous suspension of multilamellar vesicles (MLVs). The turbid MLV suspensions were then extruded 19 times through a single polycarbonate membrane (800 nm diameter pores, Avanti Polar Lipids) at room temperature in an Avestin Liposofast extruder, to give a 3.5 mM suspension of 800 nm large unilamellar vesicles with a 1 mol % loading (35 μM) of foldamer.

This suspension was then purified by gel permeation chromatography. 1 mL of the suspension was diluted with 1.5 ml of MOPs buffer and loaded onto the GPC column (Sephadex G-25). The suspension was run onto the column, before eluting the vesicles with 3.5 mL of MOPS buffer solution giving a 3.5 mL vesicle suspension of EYPC LUVs (1 mM lipid) and with a 1 mol % loading of the requisite peptide (10 μM).

Suspensions of 800 nm EYPC vesicles containing foldamer (2 mL) were added to a quartz cuvette and measurements were taken. If required, the suspensions (1 mL) were diluted to give suspensions at 5, 2.5, and 1 μM foldamer concentrations (each 2 mL).

4.1.2. Preparation of egg yolk phosphatidylcholine (EYPC) giant unilamellar vesicles (GUVs) containing foldamers 37 and 38.

GUVs were prepared by following an standard electroformation protocol.[9] GUVs composed of EYPC with 1 mol % loading of **37** or **38** were made by slight modification of literature protocols.[9a] The desired phospholipid in chloroform (18 µL, 10 mM) and **37** (20 µL, 1 mM) were mixed and spotted onto an indium tin oxide (ITO) glass slide. The chloroform was evaporated to leave a thin lipid film. The electroformation chamber was assembled, glucose solution (30 µL, 300 mM) was added and the chamber sealed. Vesicles were electroformed at 30 °C for EYPC in glucose solution (300 mM). After electroformation, GUV suspensions (30 µL) were mixed with a sucrose solution (30 µL, 300 mM) in a custom-built glass chamber and the GUVs visualized using *epi*-fluorescence microscopy.

4.2. Fluorescence spectroscopy and spectrophotometry experimental details

4.2.1. Spectroscopic solvents

MilliQ water, HPLC grade or spectrophotometric grade solvents were used for all experiments. Aliquots of solutions were added using Gilson® pipettes (aqueous solutions) or Hamilton® syringes (organic solvents and corrosive solutions).

4.2.2. Spectroscopy and microscopy: Instruments.

All spectrophotometric experiments were performed in Hellma® quartz fluorescence cuvettes with a 10 mm pathlength (3.5 mL capacity). All fluorescence experiments were performed on a Varian Cary Eclipse fluorimeter equipped with a temperature controller. Experiments were performed with an excitation wavelength of 346 nm with excitation slits of 5 nm and emission slits of 2.5 nm for solvents and 5.0 nm for vesicle suspensions. Unless otherwise stated, spectra were recorded at 25 °C and under ambient oxygen concentrations. All UV-visible spectrophotometric measurements were performed at 25 °C using a temperature controlled JASCO V-660 spectrophotometer. Circular dichroism (CD) measurements were performed using either an Applied Photophysics Chirascan qCD (0.01 mM foldamer in CH_3CN at 25 °C, in a 10 mm cell) or a JASCO J-815 spectropolarimeter (0.25 mM foldamer in CH_3CN at 20 °C, in a 1 mm cell). Fluorescence microscopy was carried out using a Zeiss Axio Imager A1 and pictures taken using a Canon Powershot G6 digital camera.

4.2.3. Preparation of stock solutions of 9-14, 20-23, 29, 30, 35-38, 41, 43, 44, 52 and 53.

Stock solutions of foldamers (1 mg/mL) were prepared in methanol. These stock solutions were stored at 5 °C in the dark between experiments. Appropriate aliquots of these stock solutions were added to 2 mL of either organic solvent in a quartz cuvette to give a final compound concentration of 10 μM. Aliquots of the appropriate solutions in organic solvent (1 mL) were serially diluted as required to give organic solvent solutions (2 mL) at 5, 2.5, and 1.25 μM foldamer concentrations respectively.

4.2.4. Fluorescence spectra for 20-23

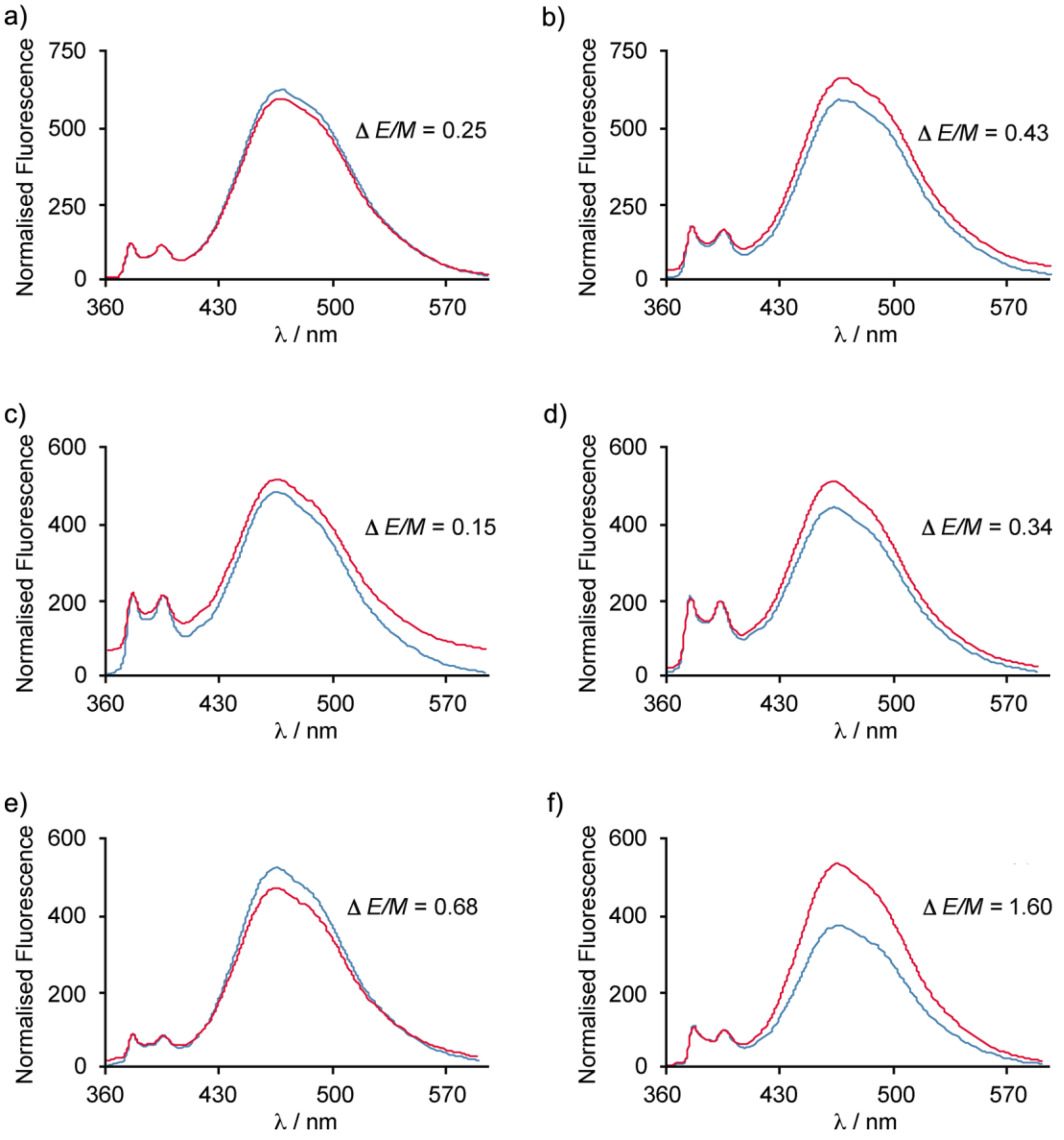

Figure S1. Fluorescence spectra of (a) **20** (blue trace) and **21** (red trace) in CH_3CN; (b) **22** (blue trace) and **23** (red trace) in CH_3CN, (c) **20** (blue trace) and **21** (red trace) in CH_3OH; (d) **22** (blue trace) and **23** (red trace) in CH_3OH; (e) **20** (blue trace) and **21** (red trace) in CH_2Cl_2; (f) **22** (blue trace) and **23** (red trace) in CH_2Cl_2;. Intensities are normalized to 1 at 395 nm, corresponding to monomer emission. Excimer emission intensity was measured at 467 nm. All foldamer concentrations are 10 μM.

4.2.5. Fluorescence spectra for 9-14

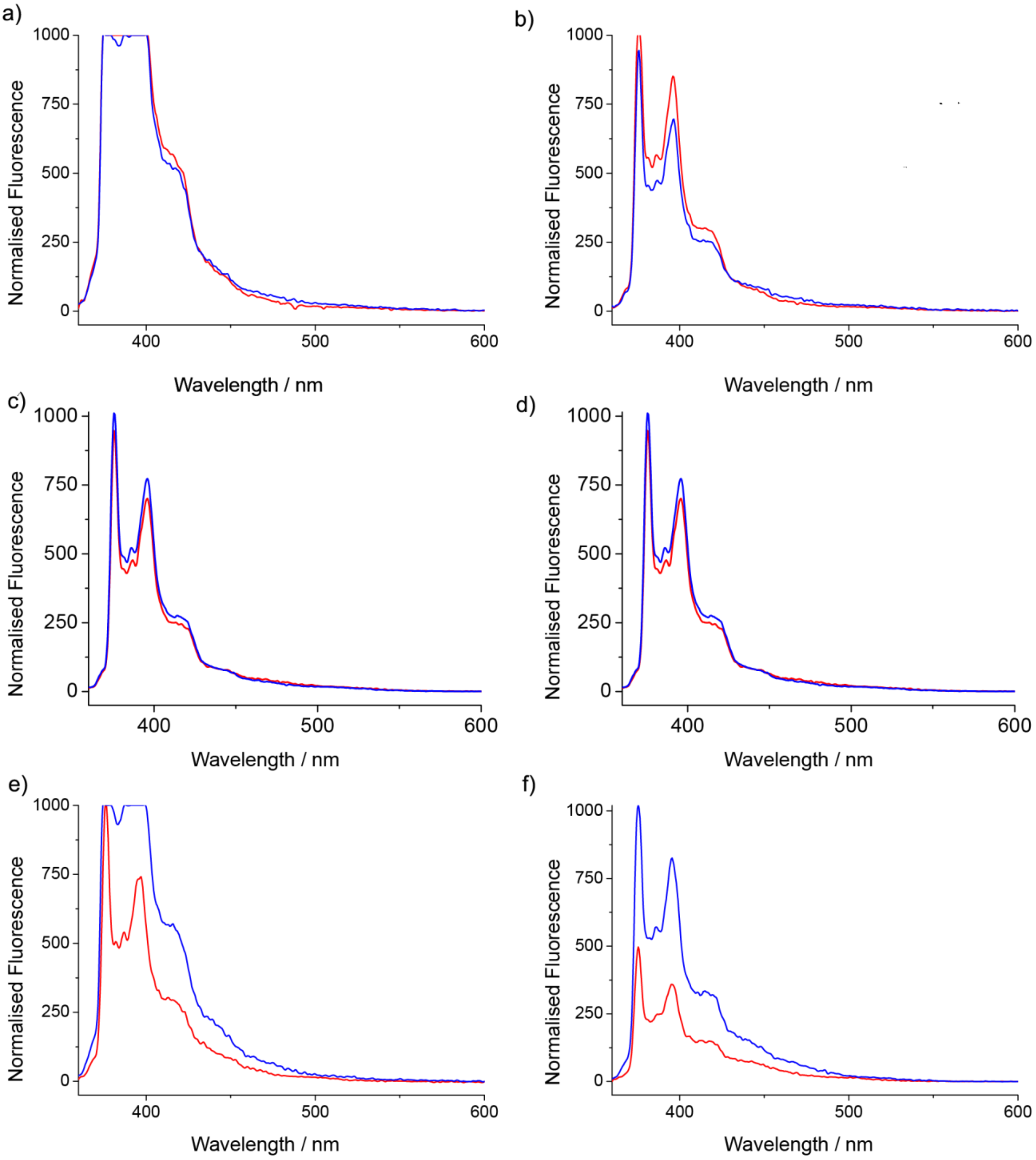

Figure S2: Fluorescence emission spectra of probes **4**, **5** and **6** when attached to Cbz-L-PheAib$_4$or Cbz-D-PheAib$_4$; compounds **9**, **11**, **13** and **10**, **12**, **14** respectively. All concentrations 20 μM. (a) Cbz(L-Phe)Aib$_4$(L-Pya)(L-Pya)OMe **11** (red) and Cbz(D-Phe)Aib$_4$(L-Pya)(L-Pya)OMe **12** (blue) in CH_2Cl_2; (b) Cbz(L-Phe)Aib$_4$(L-Pya)(L-Pya)OMe **11** (red) and Cbz(D-Phe)Aib$_4$(L-Pya)(L-Pya)OMe **12** (blue) in CH_3CN; (c) Cbz(L-Phe)Aib$_4$(L-Pya)(D-Pya)OMe **9** (red) and Cbz(D-Phe)Aib$_4$(L-Pya)(D-Pya)OMe **10** (blue) in CH_2Cl_2; (d) Cbz(L-Phe)Aib$_4$(L-Pya)(D-Pya)OMe **9** (red) and Cbz(D-Phe)Aib$_4$(L-Pya)(D-Pya)OMe **10** (blue) in CH_3CN; (e) Cbz(L-Phe)Aib$_4$(L-Pya)NHCH$_2$(pyrene) **13** (red) and Cbz(D-Phe)Aib$_4$(L-Pya)NHCH$_2$(pyrene) **14** (blue) in CH_2Cl_2; (f) Cbz(L-Phe)Aib$_4$(L-Pya)NHCH$_2$(pyrene) **13** (red) and Cbz(D-Phe)Aib$_4$(L-Pya)NHCH$_2$(pyrene) **14** (blue) in CH_3CN.

4.2.6. CD spectra for 9-14

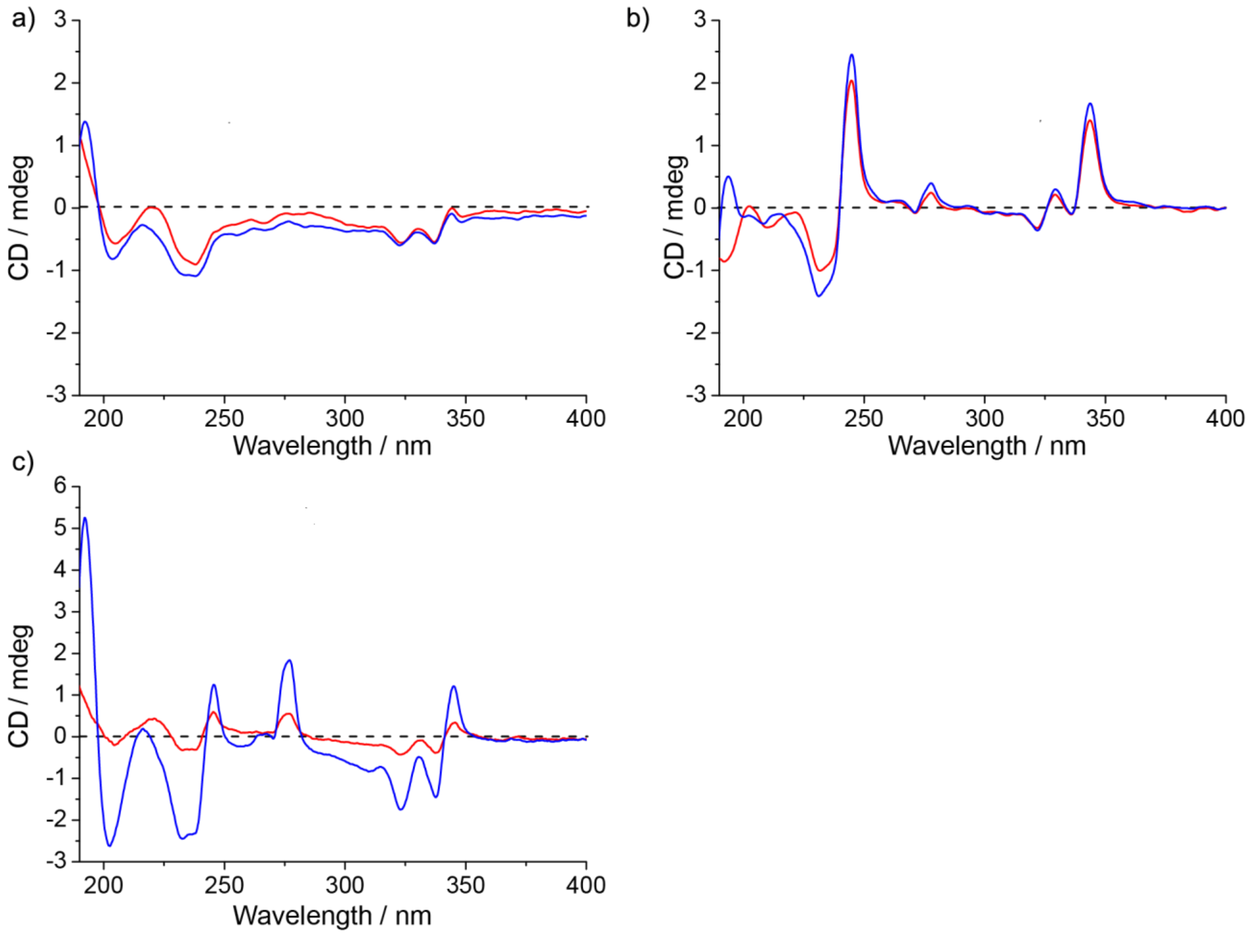

Figure S3: CD spectra for compounds (20 μM in MeCN) (a) Cbz(L-Phe)Aib$_4$(L-Pya)(L-Pya)OMe **11** (red) and Cbz(D-Phe)Aib$_4$(L-Pya)(L-Pya)OMe **12** (blue); (b) Cbz(L-Phe)Aib$_4$(L-Pya)(D-Pya)OMe **9** (red) and Cbz(D-Phe)Aib$_4$(L-Pya)(D-Pya)OMe **10** (blue); (c) Cbz(L-Phe)Aib$_4$(L-Pya)NHCH$_2$(pyrene) **13** (red) and Cbz(D-Phe)Aib$_4$(L-Pya)NHCH$_2$(pyrene) **14** (blue).

4.2.7. Fluorescence and CD spectra for 29 and 30

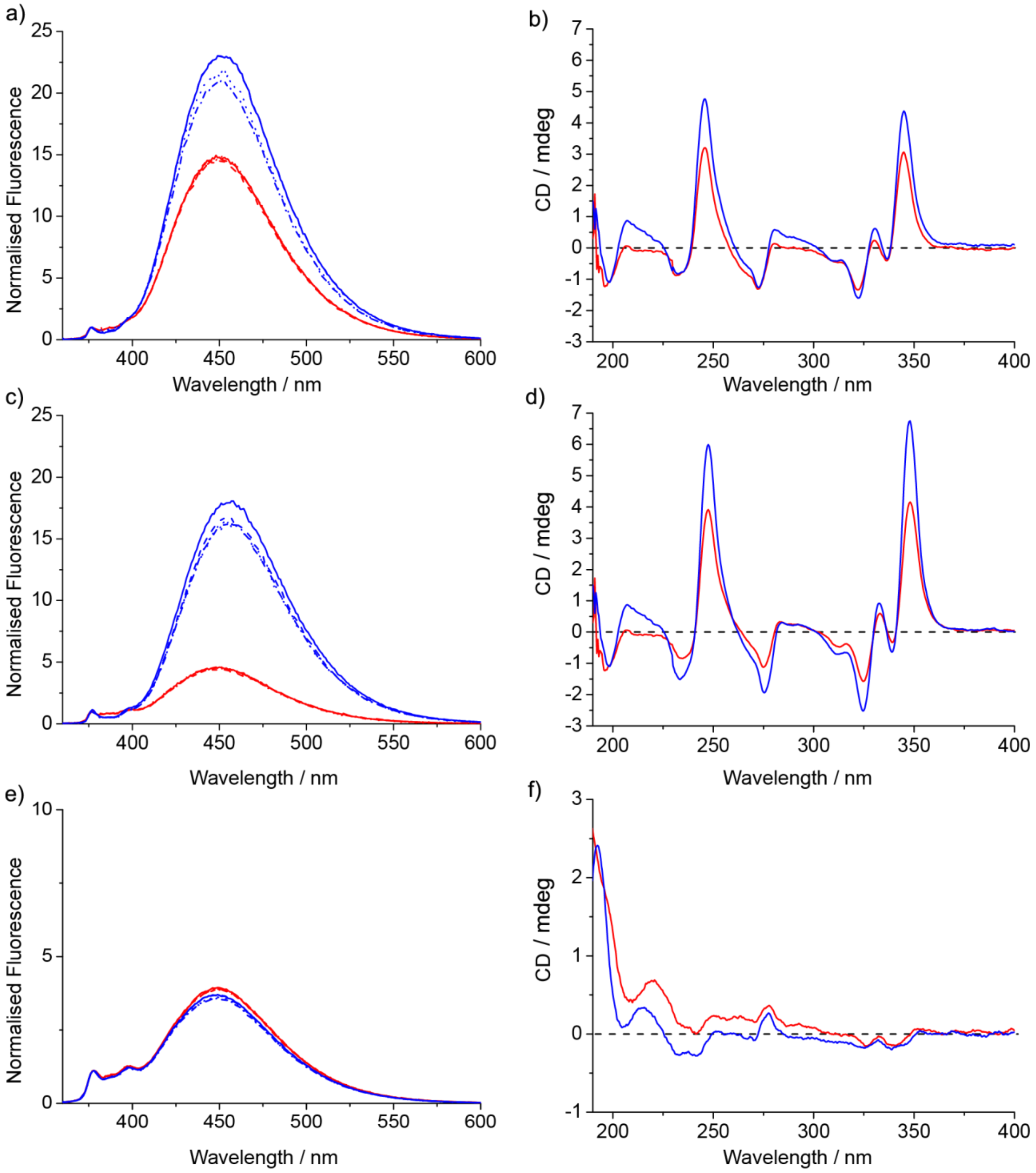

Figure S4: (a, c, e) Fluorescence spectra and (b, d, f) circular dichroism of compounds Cbz(L-Phe)Aib_4(*S,S*-BisPyrEt)NH_2 **29** (red) and Cbz(D-Phe)Aib_4(*S,S*-BisPyrEt)NH_2 **30** (blue) in (a,b) MeOH, (c,d) CH_3CN and (e,f) CH_2Cl_2. Fluorescence measured at 10 μM (———) 5 μM (— — —) 2.5 μM (— · —) and 1.25 μM (···) concentrations and normalised to 1 at 377 nm.

4.2.8. Fluorescence spectra for 29 and 30 at different concentrations

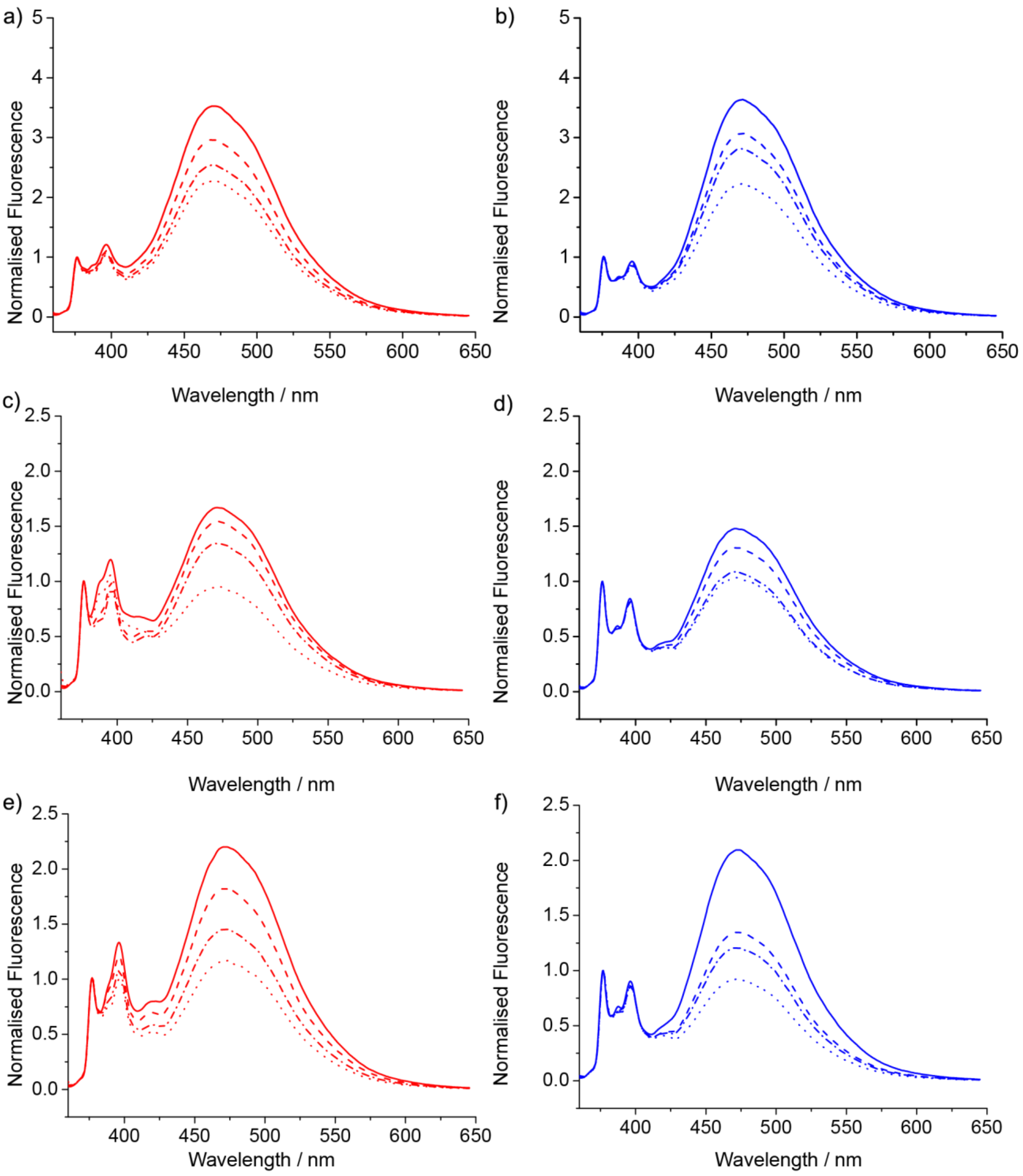

Figure S5: Fluorescence spectra of compounds Cbz(L-Phe)Aib$_4$(*S*,*S*-BisPyrEt)NH_2 **29** (red) and Cbz(D-Phe)Aib$_4$(*S*,*S*-BisPyrEt)NH_2 **30** (blue) in CH_3OH (a,b) CH_3CN (c,d) and CH_2Cl_2 (e,f). Fluorescence measured at 10 μM (———) 5 μM (— — —) 2.5 μM (— · —) and 1.25 μM (···) concentrations and normalised to 377 nm.

4.2.9. Fluorescence spectra for 35 and 36 at different concentrations

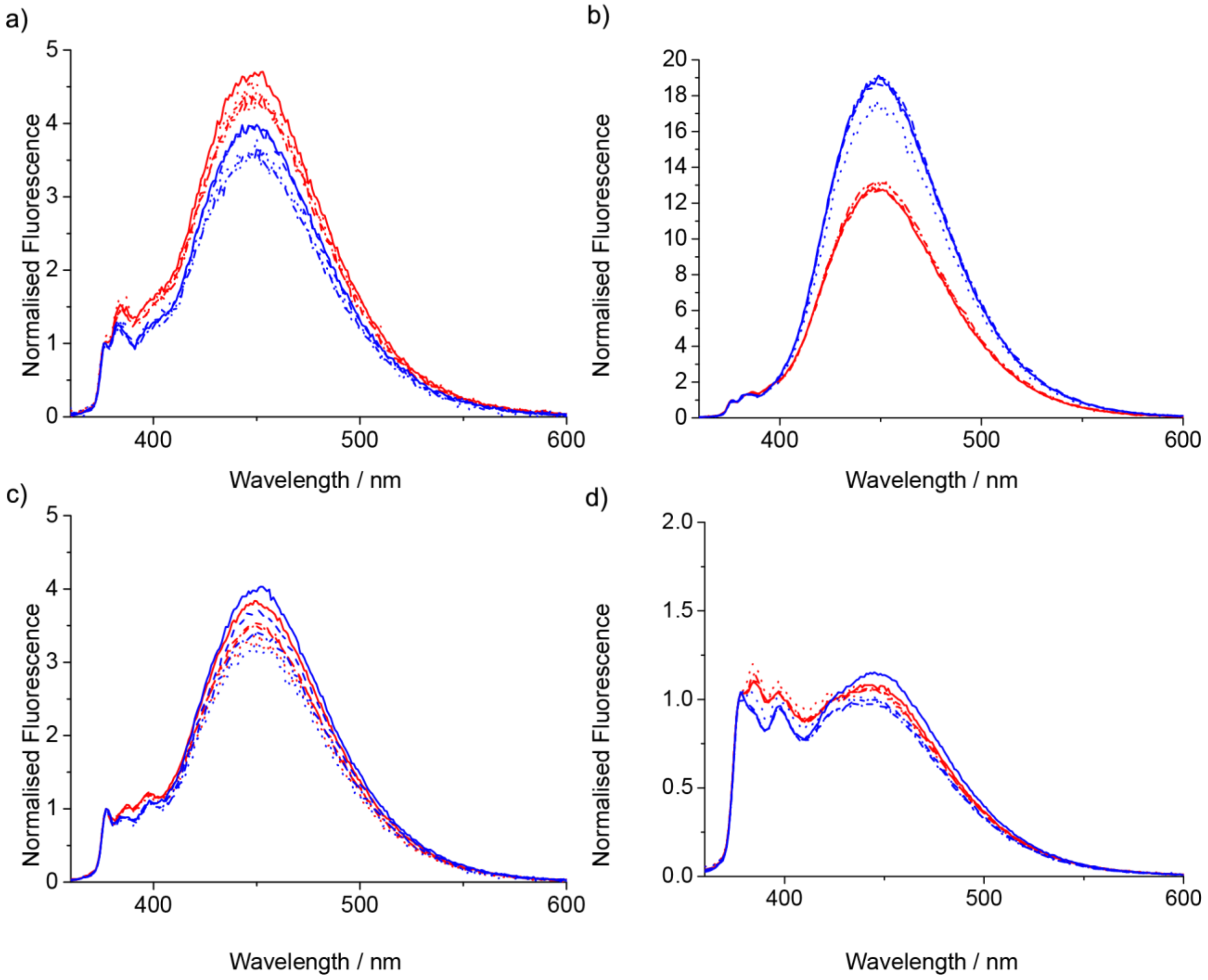

Figure S6: Fluorescence spectra of compounds Cbz(D-αMeVal)Aib$_4$(*S,S*-BisPyrEt)NH$_2$ **36** (red) and Cbz(L-αMeVal)Aib$_4$(*S,S*-BisPyrEt)NH$_2$ **35** (blue) in (a) CH_3CN, (b) MeOH, (c) CH_2Cl_2 and (d) THF. Fluorescence measured at 10 µM (▬▬▬) 5 µM (▬ ▬ ▬) 2.5 µM (▬ ▪ ▬) and 1.25 µM (▪▪▪) concentrations and normalised to 1 at 377 nm.

4.2.10. Fluorescence spectra for 37 and 38 at different concentrations

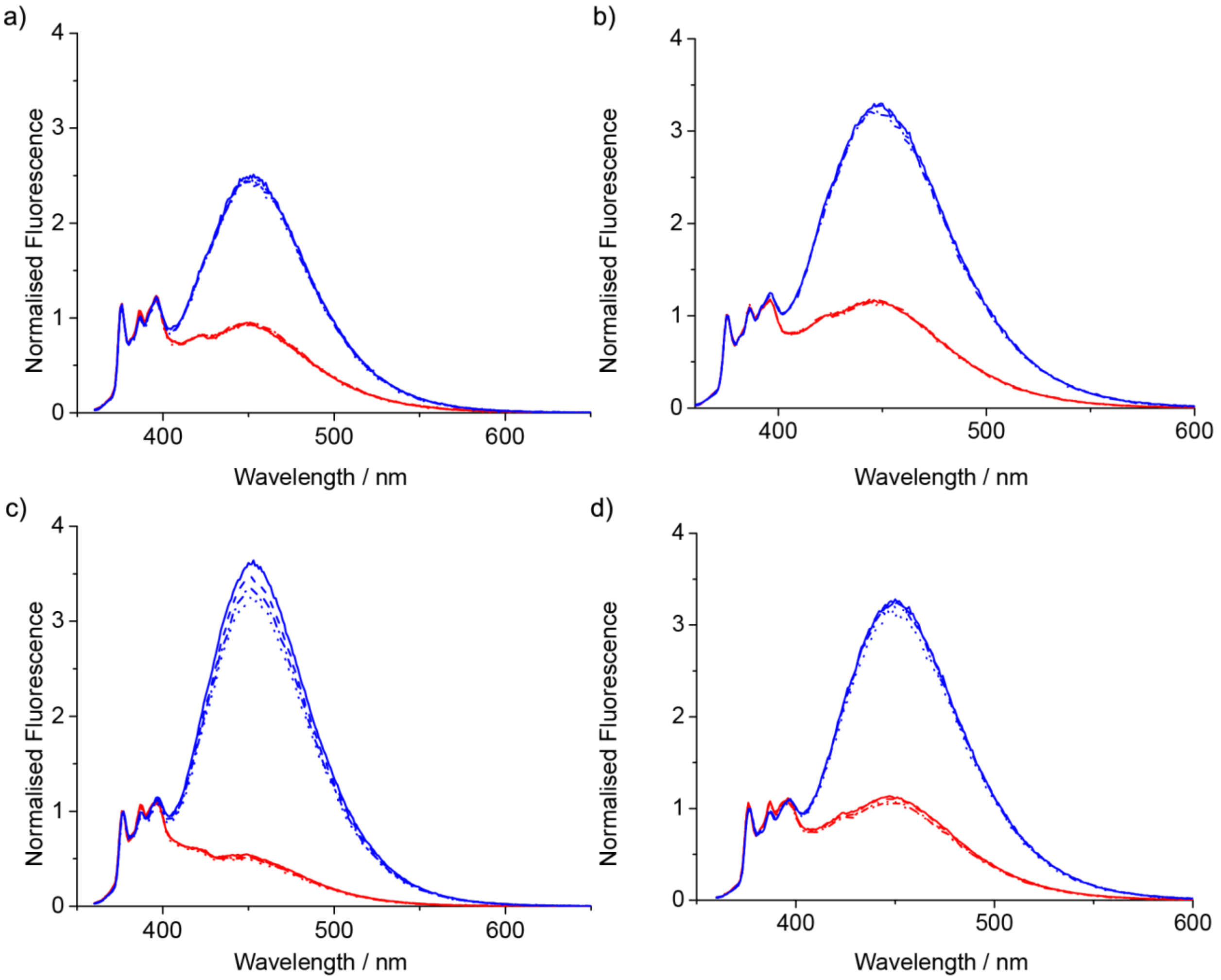

Figure S7: Fluorescence spectra of compounds Cbz(D-αMeVal)Aib$_4$(*S,S*-BisPyrEt)NHAc **38** (red) and Cbz(L-αMeVal)Aib$_4$(*S,S*-BisPyrEt)NHAc **37** (blue) in CH_3CN (**A**) MeOH (**B**) CH_2Cl_2 (**C**) and THF (**D**). Fluorescence measured at 10 µM (———) 5 µM (— — —) 2.5 µM (— · —) and 1.25 µM (···) concentrations and normalised to 1 at 377 nm.

4.2.11. Sensitivity ratios (S_R) for 35, 36, 37 and 38 at different concentrations

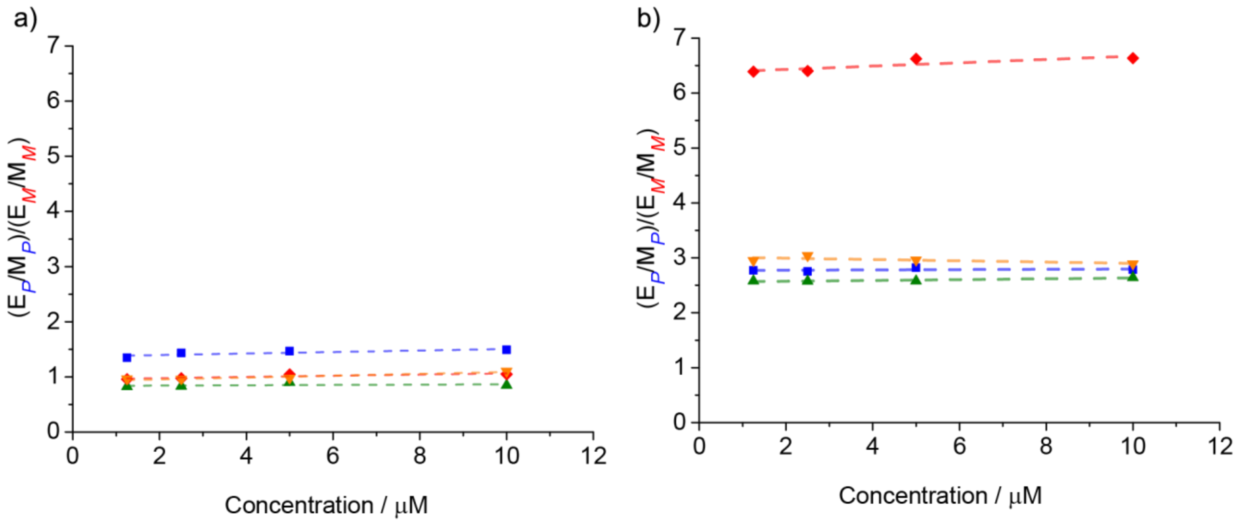

Figure S8: Ratiometric comparison of the fluorescence response of (a) amine-terminated bispyrene reporter in Cbz(D-αMeVal)Aib_4(*S,S*-BisPyrEt)NH_2 **36** and Cbz(L-αMeVal)Aib_4(*S,S*-BisPyrEt)NH_2 **35** and (b) acetamide-terminated bispyrene reporter in Cbz(D-αMeVal)Aib_4(*S,S*-BisPyrEt)NHAc **38** and Cbz(L-αMeVal)Aib_4(*S,S*-BisPyrEt)NHAc **37** when the foldamers are in dissolved CH_2Cl_2 (◆) MeOH (■) CH_3CN (▲) and THF (▼) at different concentrations. The ratiometric response in CH_2Cl_2 for the amine-terminated bispyrene reporter is identical to that in THF.

4.2.12. Sensitivity ratios (S_R) for 35, 36, 37 and 38 in EYPC vesicles

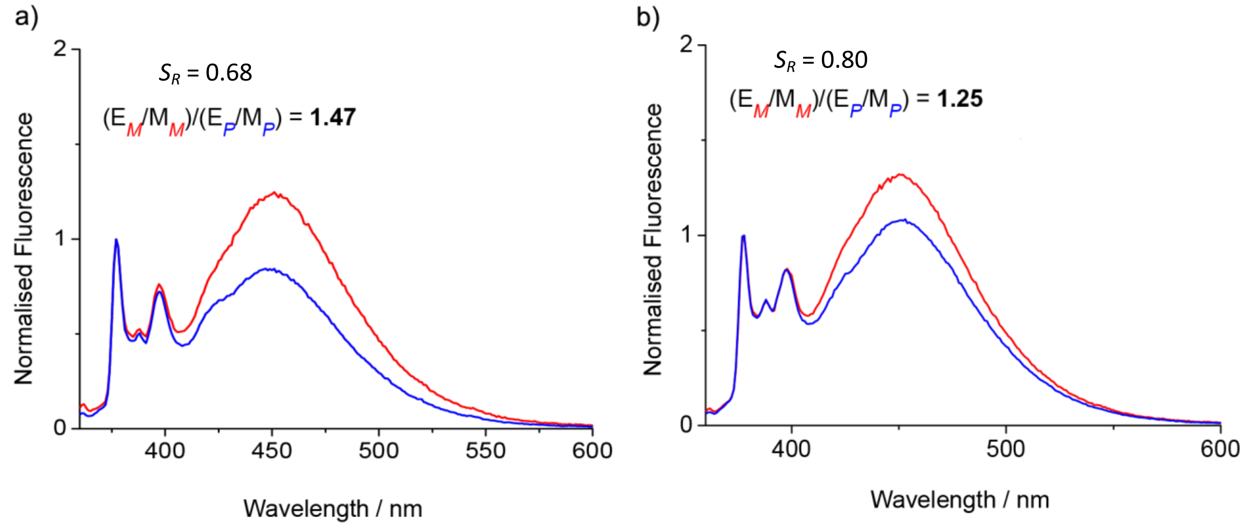

Figure S9: Four αMeVal-controlled controlled helices were loaded into to 800 nm EYPC vesicles at 1 mol % (10 µM). Fluorescence response from (a) Cbz(D-αMeVal)Aib_4(*S,S*-BisPyrEt)NH_2 **36** (red) and Cbz(L-αMeVal)Aib_4(*S,S*-BisPyrEt)NH_2 **35** (blue); (b) Cbz(D-αMeVal)Aib_4(*S,S*-BisPyrEt)NHAc **38** (red), Cbz(L-αMeVal)Aib_4(*S,S*-BisPyrEt)NHAc **37** (blue),

4.2.13 E/M values and helical excess (h.e.) for foldamers 37, 38, 41, 43, 44, 52 and 53 in MeOH and CH_3CN.

Compound, *[Controller]*	Δδ[a]	*h.e.*[b]	E/M in MeOH[c]	E/M in CH_3CN[c]
**53**, *[Cbz(D-αMeVal)$_2$]*	383	-95	1.12	0.71
**38**, *[Cbz(D-αMeVal)]*	275	-68	1.38	1.23
**41**, *[Cbz(Gly)]*	0	0	2.47	1.72
**43**, *[N_3]*	0	0	2.50	n/d
**44**, *[Cbz(D-Phe)]*	209	+52	3.32	n/d
**37**, *[Cbz(L-αMeVal)]*	275	+68	3.81	2.51
**52**, *[Cbz (L-αMeVal)$_2$]*	383	+95	5.28	3.83

Table S1. E/M values and reported induced screw-sense preferences[10] for foldamers **37**, **38**, **41**, **43**, **44**, **52** and **53** in different solvents at 10 μM. *a* Anisochronicity induced by each *Controller* in Aib_4 foldamers, determined using a C-terminal $GlyNH_2$ probe in MeOH.[10] *b* Corresponding *M*:*P* screw sense ratio; *c E/M* ratio in MeOH and CH_3CN measured using the (*S*,*S*-BisPyrEt)NHAc probe. n/d = not determined

4.2.14. Fitting of E/M values for foldamers in MeOH as a function of helical excess

Assumptions:

- Only *M* helix and *P* helix are contributing to the observed E/M.
- Excimer (or monomer) emission is the weighted sum of the excimer (or monomer) emission from each screw sense.
- Let E and M represent the excimer and monomer emission (respectively) from a fixed concentration of foldamer (e.g .1 μM), which is the respective sums for emission from the fraction of foldamer in a *P* helix (f_P) and the fraction of foldamer in an *M* helix (f_M).
- Let the ratio $(E/M)_f$ be the E/M ratio as a function of foldamer helical excess, expressed below as the fraction of *P* helical foldamer (f_P).

(1)

$$(E/M)_f = \frac{f_P E_P + (1 - f_P)E_M}{f_P M_P + (1 - f_P)M_M}$$

$$\text{let } \frac{E_P}{M_P} = (E/M)_{+1} \quad \text{and} \quad \frac{E_M}{M_M} = (E/M)_{-1}$$

Eqn. (1)

$$(E/M)_f = \frac{f_P (E/M)_{+1} M_P + (1 - f_P)(E/M)_{-1} M_M}{f_P M_P + (1 - f_P)M_M}$$

(2) If $f_P = 0.5$ (i.e. *h.e.* = 0), then

$$(E/M)_0 = \frac{(E/M)_{+1} M_P + (E/M)_{-1} M_M}{M_P + M_M}$$

$$(M_P)(E/M)_0 + (M_M)(E/M)_0 = (E/M)_{+1} M_P + (E/M)_{-1} M_M$$

$$(M_M)(E/M)_0 - (E/M)_{-1} M_M = (E/M)_{+1} M_P - (M_P)(E/M)_0$$

$$M_M = \frac{(E/M)_{+1} M_P - (M_P)(E/M)_0}{(E/M)_0 - (E/M)_{-1}}$$

Eqn. (2)

$$M_M = (M_P)\left[\frac{(E/M)_{+1} - (E/M)_0}{(E/M)_0 - (E/M)_{-1}}\right]$$

(3) Combine equations (1) and (2)

Eqn. (3)

$$(E/M)_f = \frac{f_P (E/M)_{+1} + (1 - f_P)(E/M)_{-1}\left[\frac{(E/M)_{+1} - (E/M)_0}{(E/M)_0 - (E/M)_{-1}}\right]}{f_P + (1 - f_P)\left[\frac{(E/M)_{+1} - (E/M)_0}{(E/M)_0 - (E/M)_{-1}}\right]}$$

We know $(E/M)_0$ (a value of 2.50) and can estimate $(E/M)_{-1}$ and $(E/M)_{+1}$ (1.05 and 5.5 respectively) from data for αMeVal controlled foldamers, so we can calculate (E/M) as a function of f_P.

(4) To obtain $(E/M)_{h.e.}$, (E/M) as a function of h.e., use:

$$h.e./100 = f_P - f_M$$

$$h.e. = 200 f_P - 100$$

at 1 mol%.

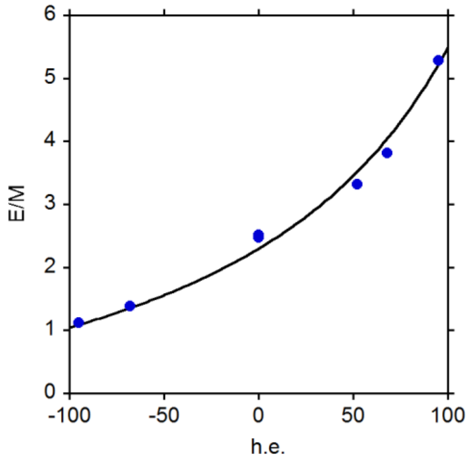

Figure S10: E/M values measured using the (*S*,*S*-BisPyrEt)NHAc reporter (monomer at 377 nm, excimer max. value 410-600 nm) as a function of helical excess (h.e.) for foldamers **37**, **38**, **41**, **43**, **44**, **52** and **53** in MeOH (1 μM). Measured values (blue circles) and calculated curve (black line) using $(E/M)_0$ = 2.50 (h.e. = 0), $(E/M)_{-1}$ = 1.05 (h.e. = −100) and $(E/M)_{+1}$ = 5.50 (h.e. = +100) respectively.

4.2.15. Fluorescence microscopy images of 37 and 38 in membranes of EYPC GUVs.

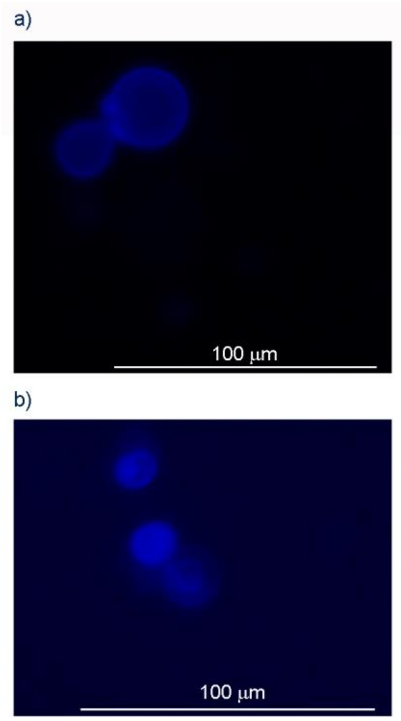

Figure S11: *Epi*-fluorescence microscopy images of EYPC GUVs with (a) **37** and (b) **38** embedded in the bilayers at 1 mol%.

4.2.16. E/M values from 43 in EYPC LUVs at loadings of 0.2, 0.4, 0.6 and 1.0 mol%

To determine the extent of any intermolecular excimer formation, a membrane dilution experiment was performed; the E/M ratio was measured at different membrane loadings of the foldamer **43** (but the same bulk concentration). Mixed thin films with different amounts of EYPC (2.1×10^{-2} mmol, 1.05×10^{-2} mmol, 7×10^{-3} mmol, 4.2×10^{-3} mmol) were made by mixing appropriate aliquots of **43** (4.2×10^{-5} mmol, 1 mg/mL, MeOH) and EYPC stock solutions (100 mg/mL, $CHCl_3$), followed by removal of the solvent under reduced pressure. The films produced were placed under high vacuum for 4 h to remove residual organic solvent. The films were then re-hydrated by addition of MOPS buffer (1.2 mL, 20 mM MOPS, pH = 7.3) followed by vortex mixing to give a homogeneous suspension of multilamellar vesicles (MLVs). The turbid MLV suspensions were extruded 19 times through an 800 nm polycarbonate membrane at room temperature in an Avestin Liposofast extruder, to give a suspension of 800 nm large unilamellar vesicles with 0.2, 0.4, 0.6 and 1 mol % loading (35 µM) of **43**. The suspensions were purified by gel permeation chromatography (Sephadex G-25) to give 3.5 mL of EYPC LUV suspensions (1 mM, 1.66 mM, 2.5 mM and 5 mM lipid), each with foldamer **43** (10 µM). The emission spectra from each vesicle suspension were measured, and the E/M values calculated (Figure S12).

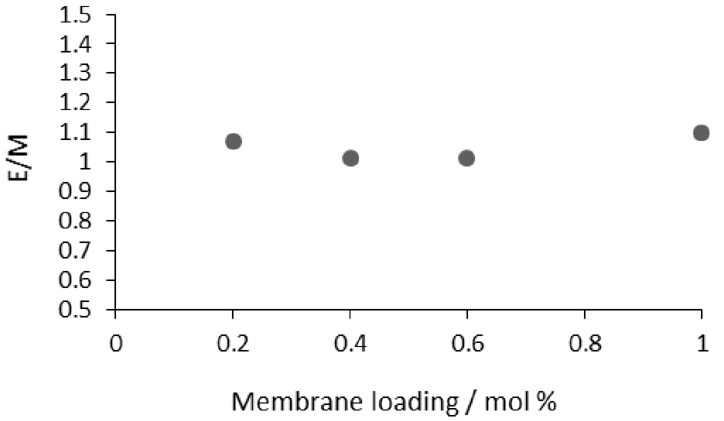

Figure S12: E/M values for EYPC LUVs containing different membrane loadings of foldamer **43**. Excimer emission measured at 455 nm.

5. NMR spectra of novel compounds

¹H NMR spectrum of Boc(L-Pya)(D-Pya)OMe 4

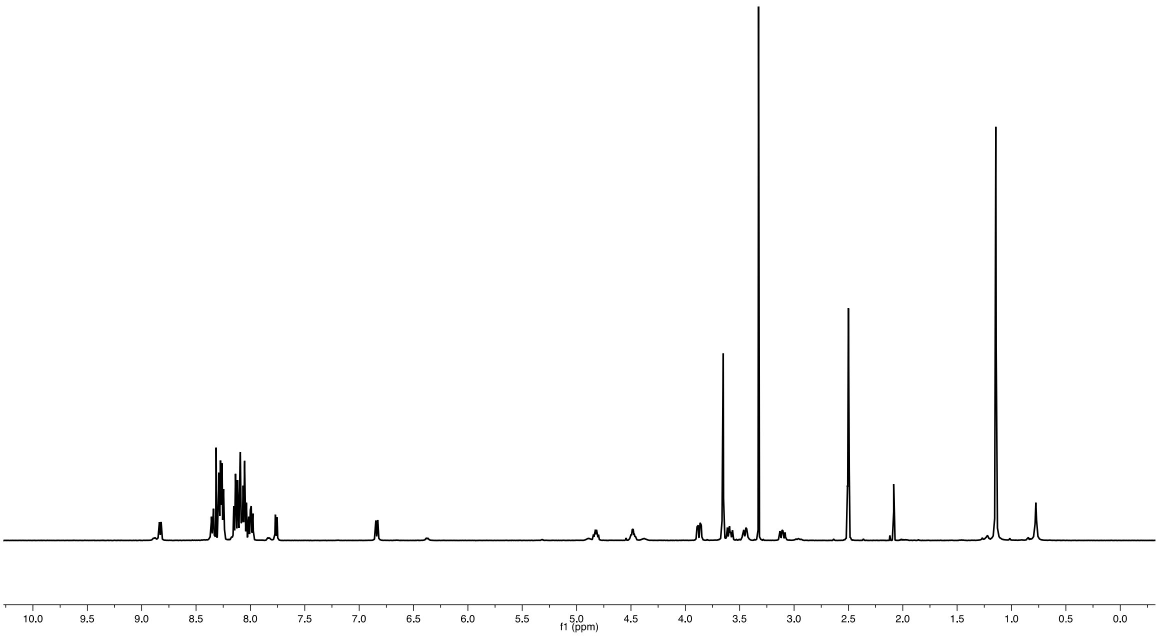

¹³C NMR spectrum of Boc(L-Pya)(D-Pya)OMe 4

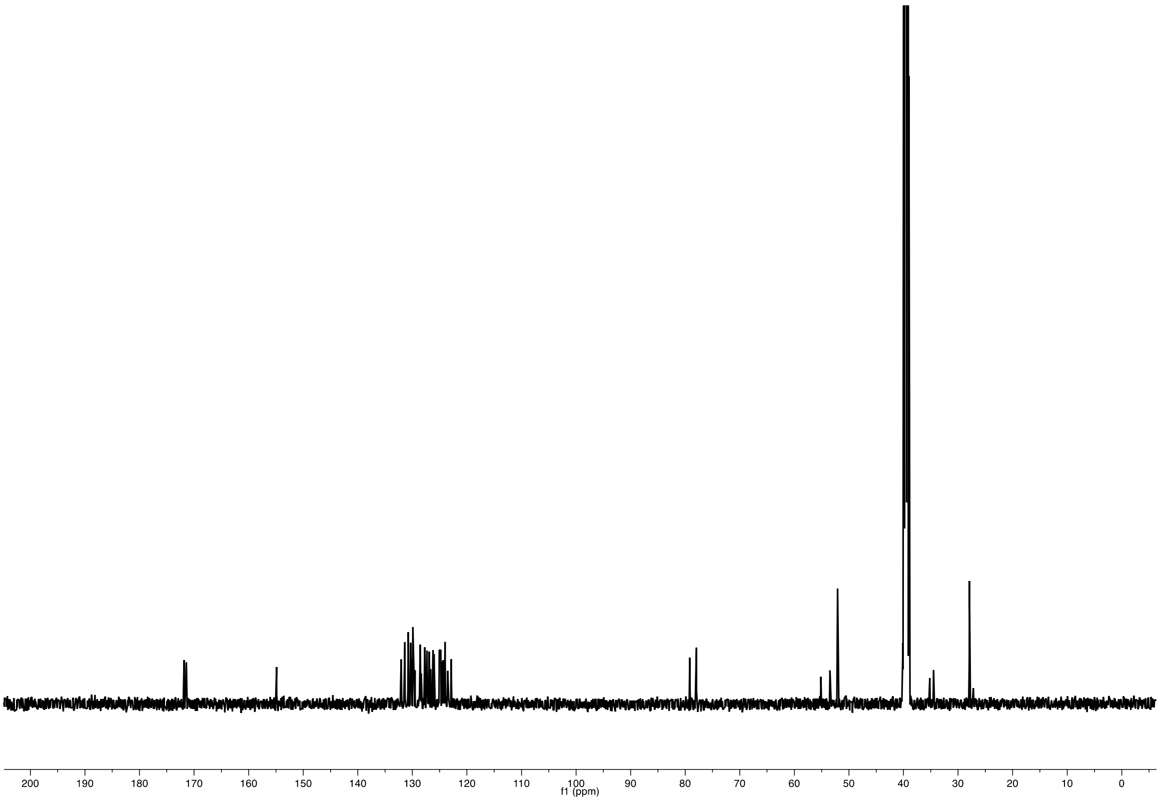

^{1}H NMR spectrum of Boc(L-Pya)(L-Pya)OMe 5

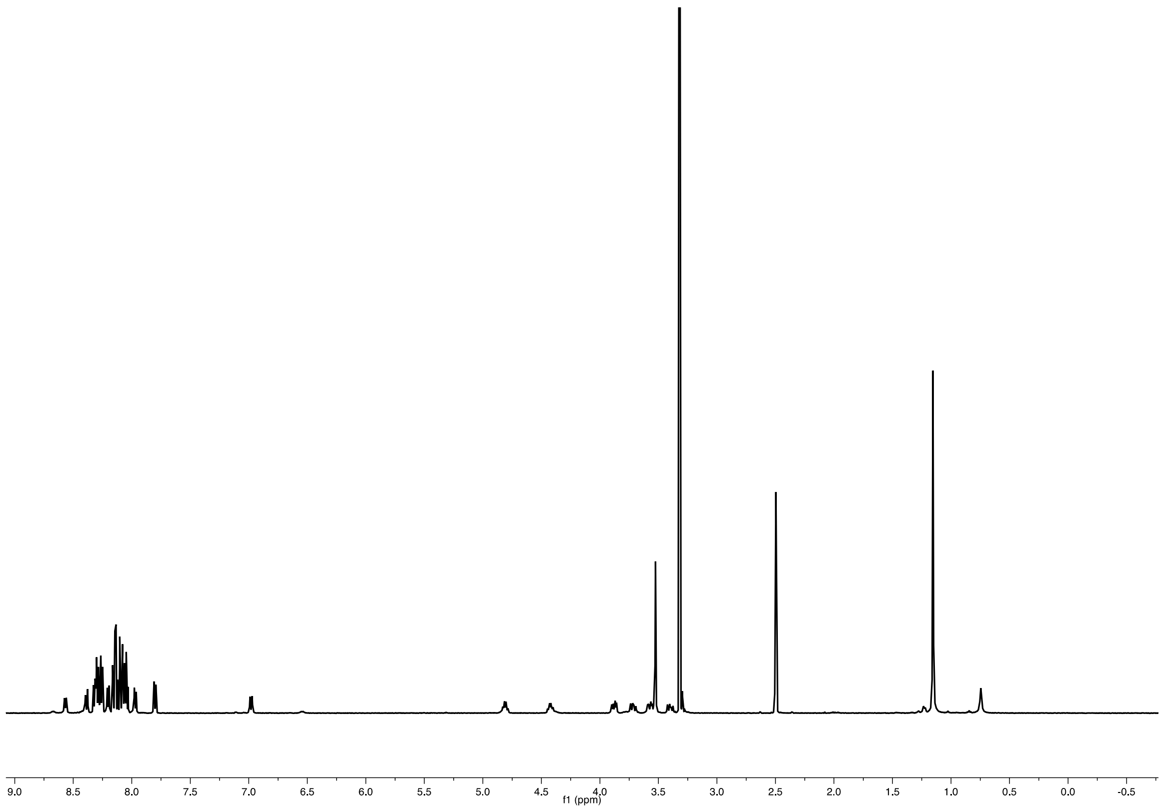

^{13}C NMR spectrum of Boc(L-Pya)(L-Pya)OMe 5

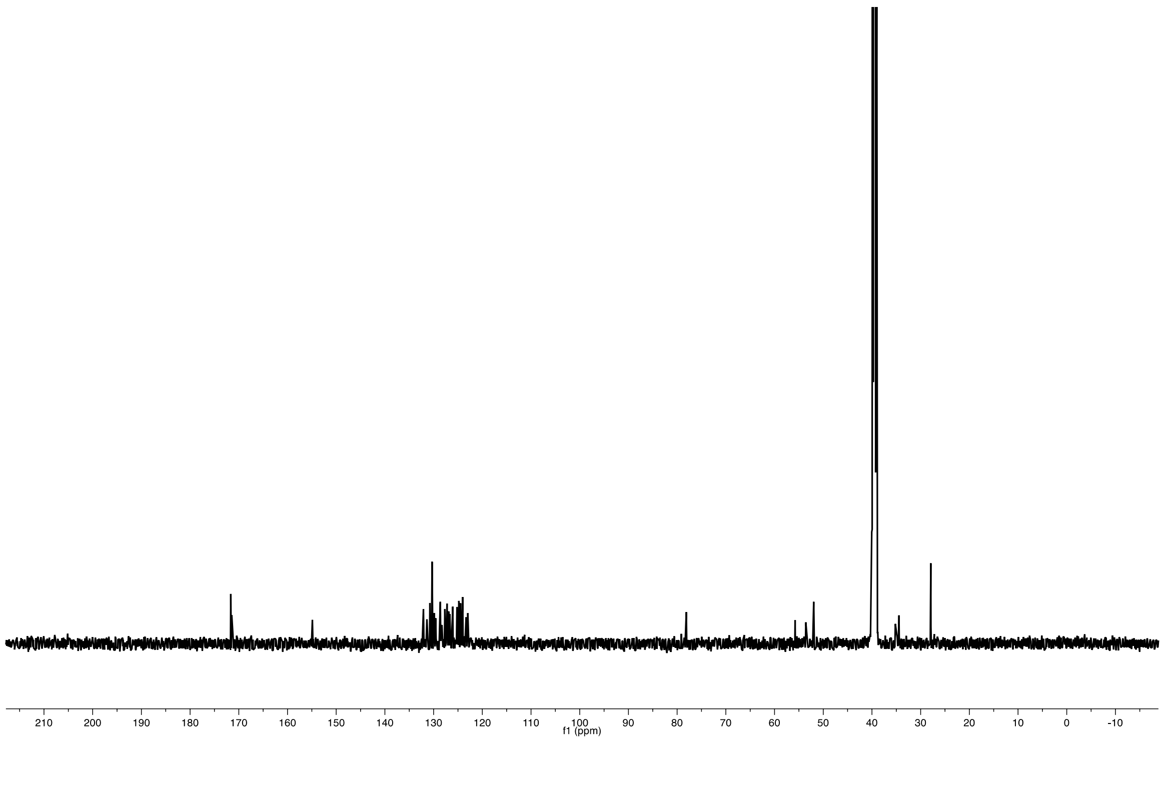

^{1}H NMR spectrum of Boc(L-Pya)NHCH$_2$Pyr 6

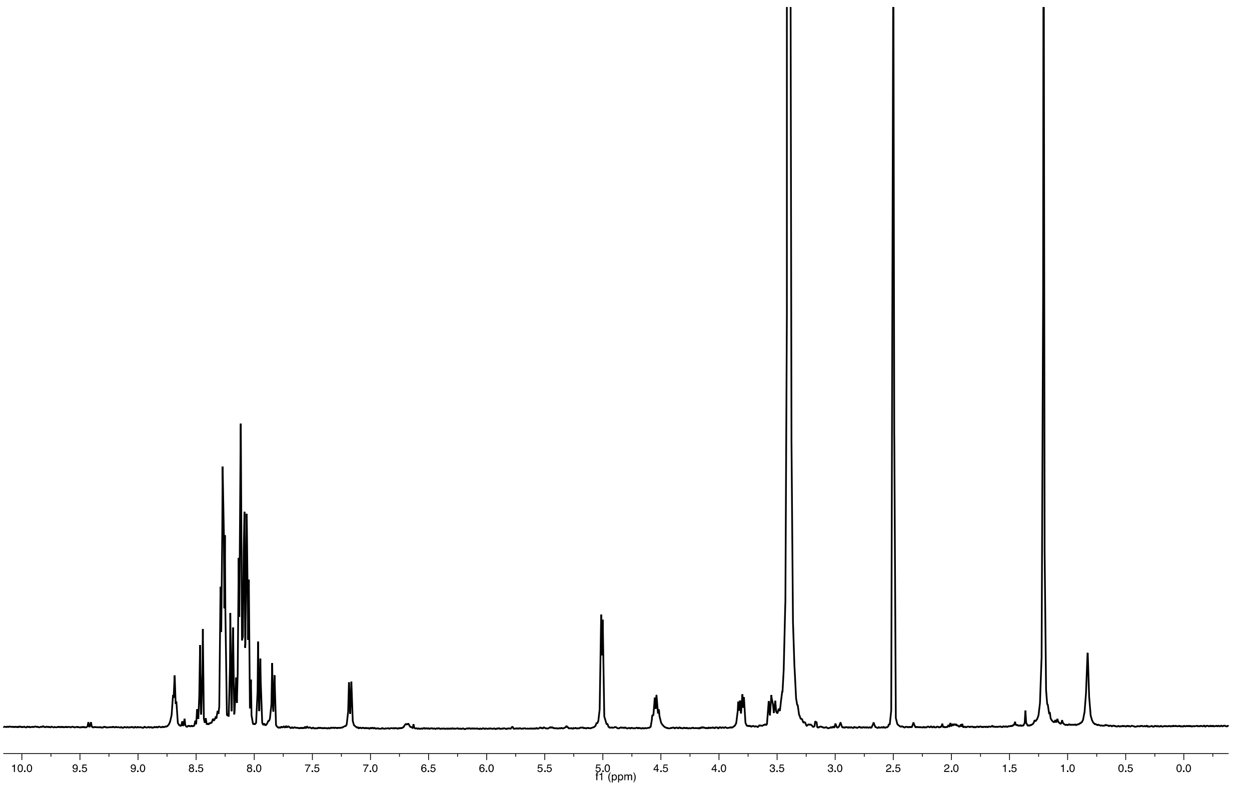

^{13}C NMR spectrum of Boc(L-Pya)NHCH$_2$Pyr 6

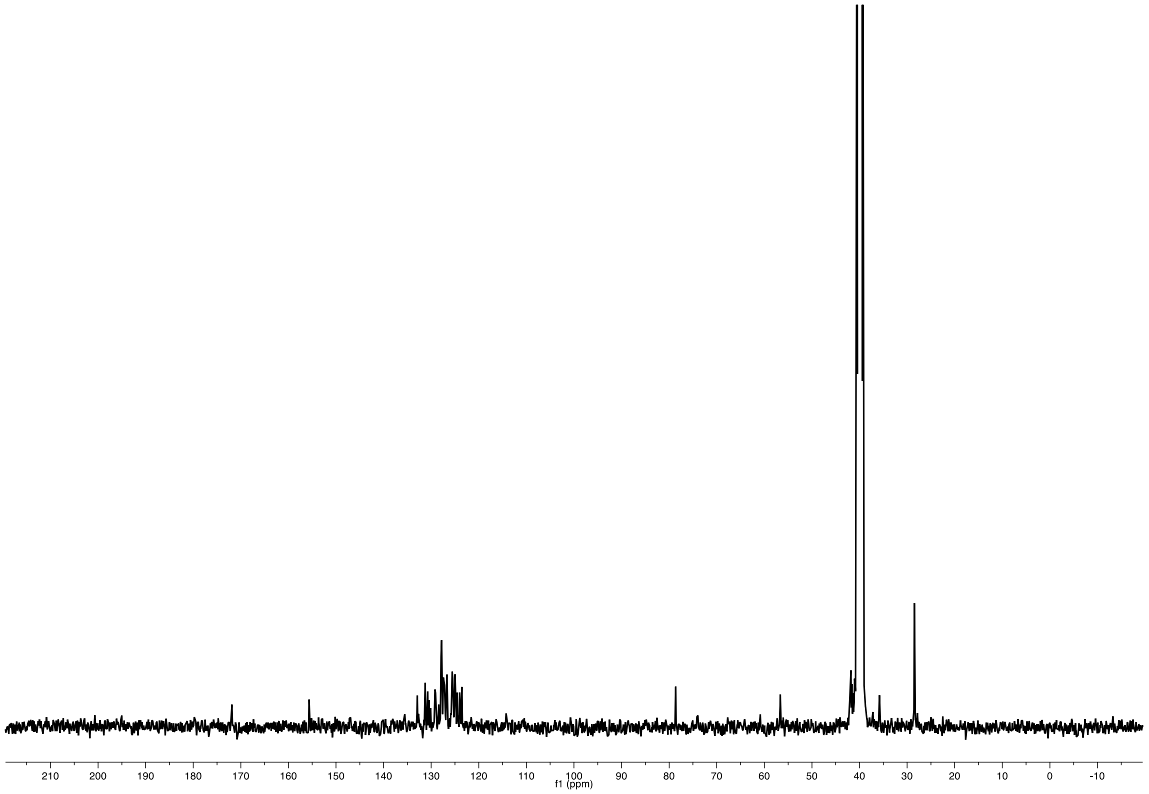

^{1}H NMR spectrum of Cbz(D-Phe)Aib$_4$(L-Pya)(L-Pya)OMe 9

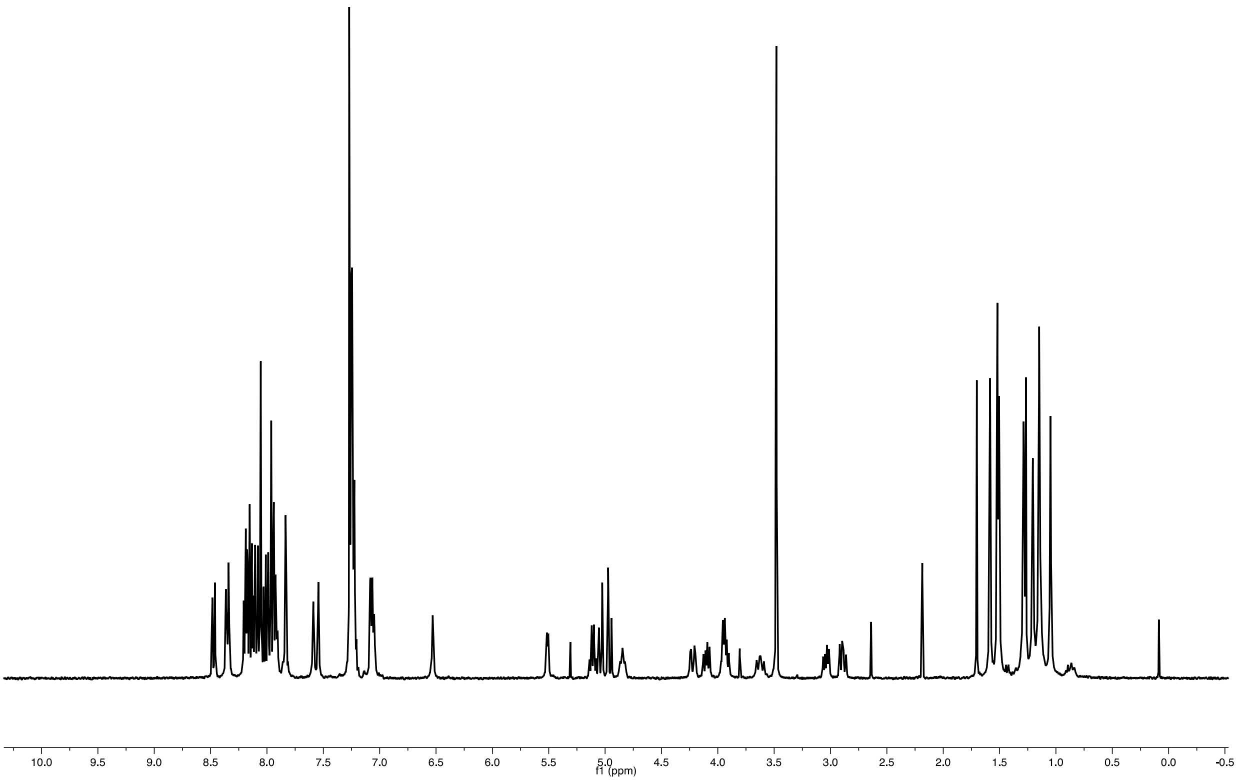

^{13}C NMR spectrum of Cbz(D-Phe)Aib$_4$(L-Pya)(L-Pya)OMe 9

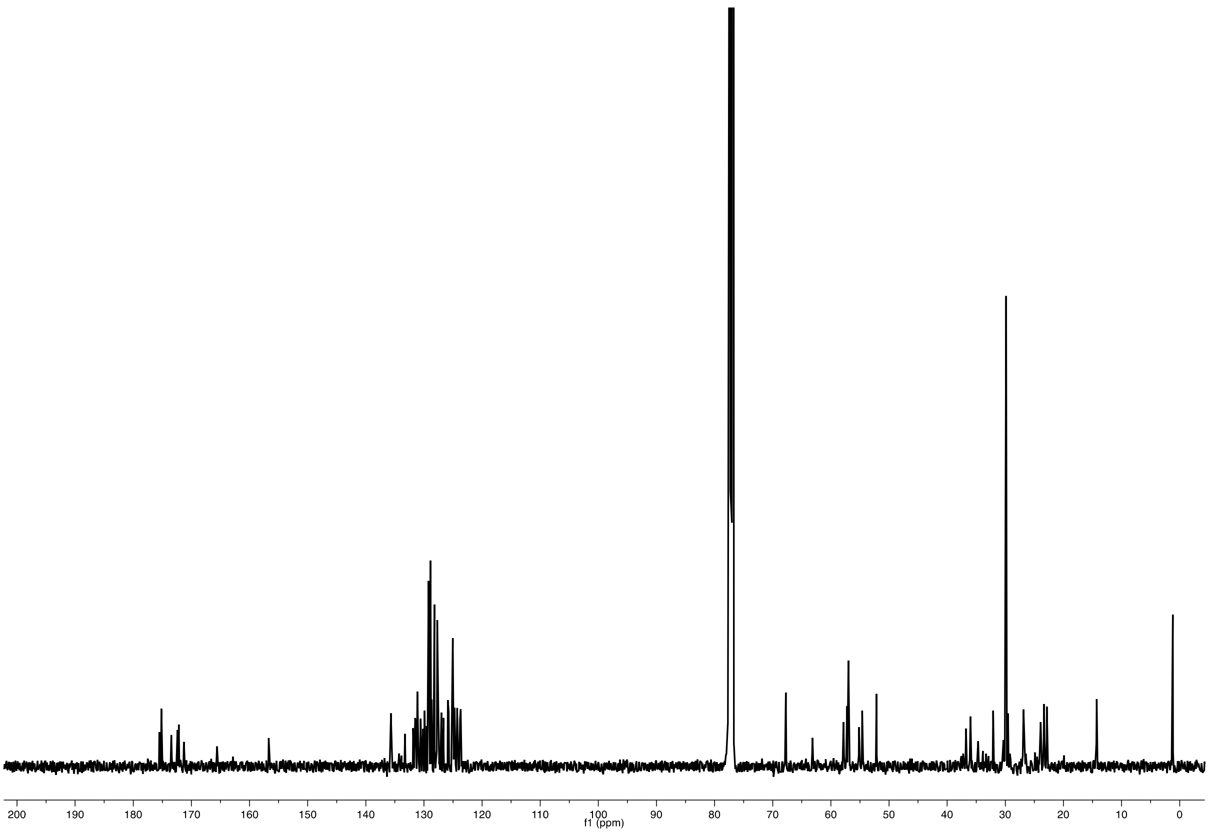

^{1}H NMR spectrum of Cbz(L-Phe)Aib$_4$(L-Pya)(L-Pya)OMe 10

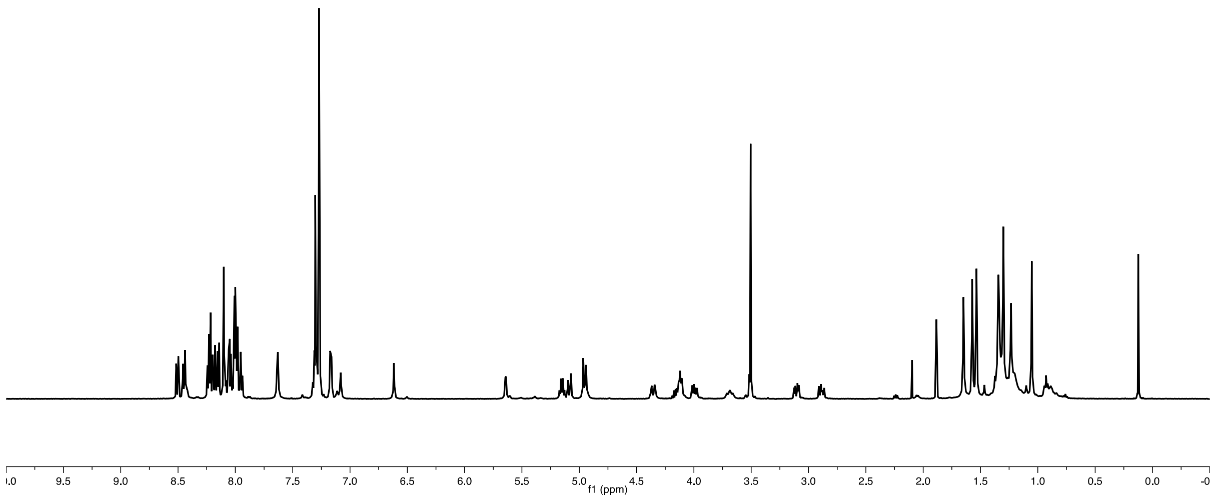

^{13}C NMR spectrum of Cbz(L-Phe)Aib$_4$(L-Pya)(L-Pya)OMe 10

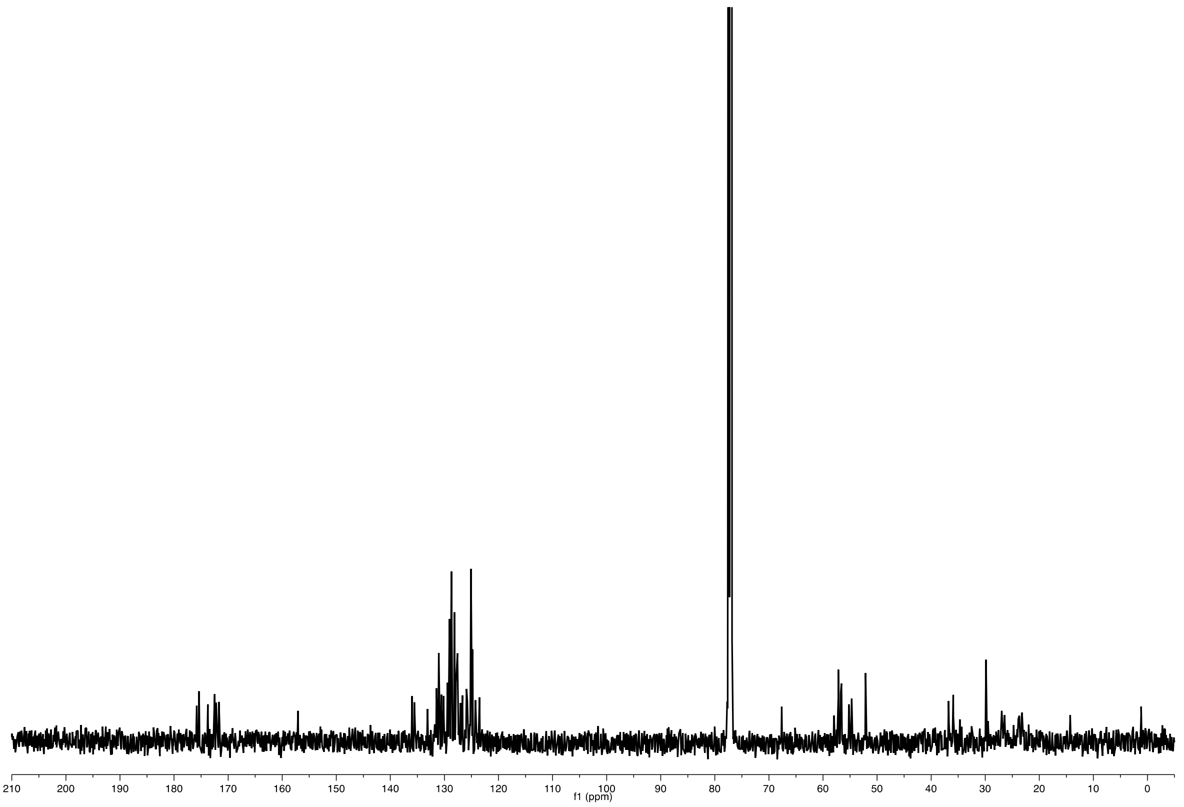

^{1}H NMR spectrum of Cbz(L-Phe)Aib$_4$(L-Pya)(D-Pya)OMe 11

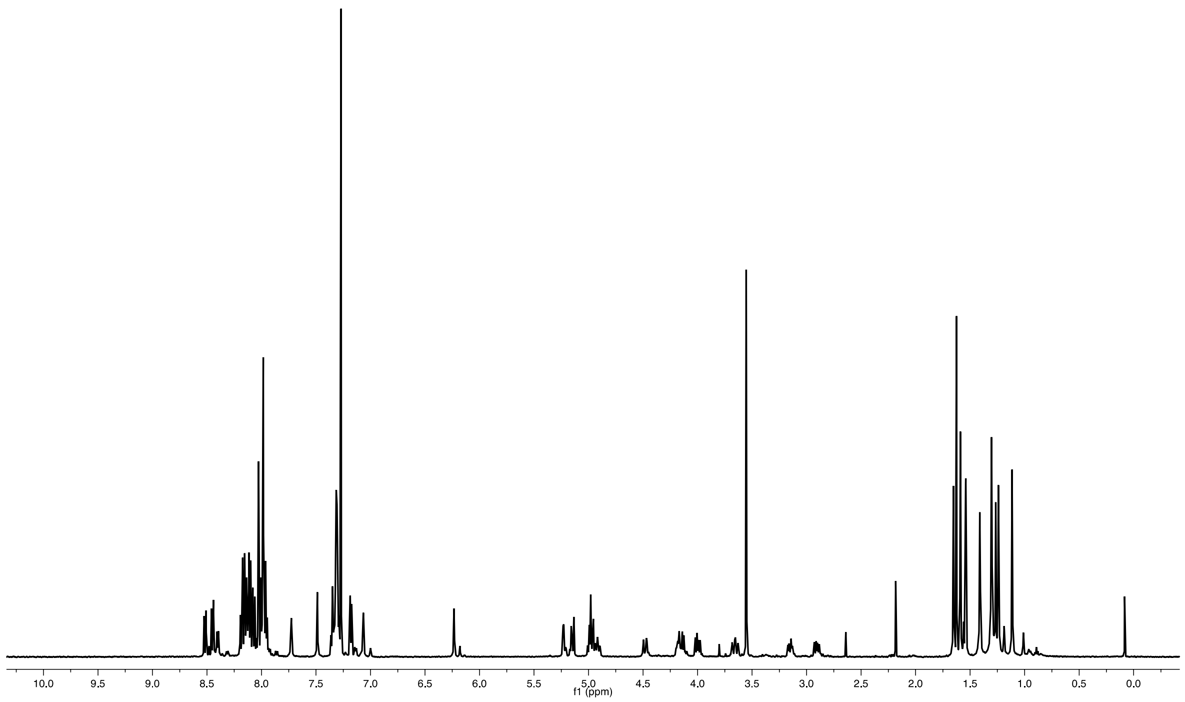

^{13}C NMR spectrum of Cbz(L-Phe)Aib$_4$(L-Pya)(D-Pya)OMe 11

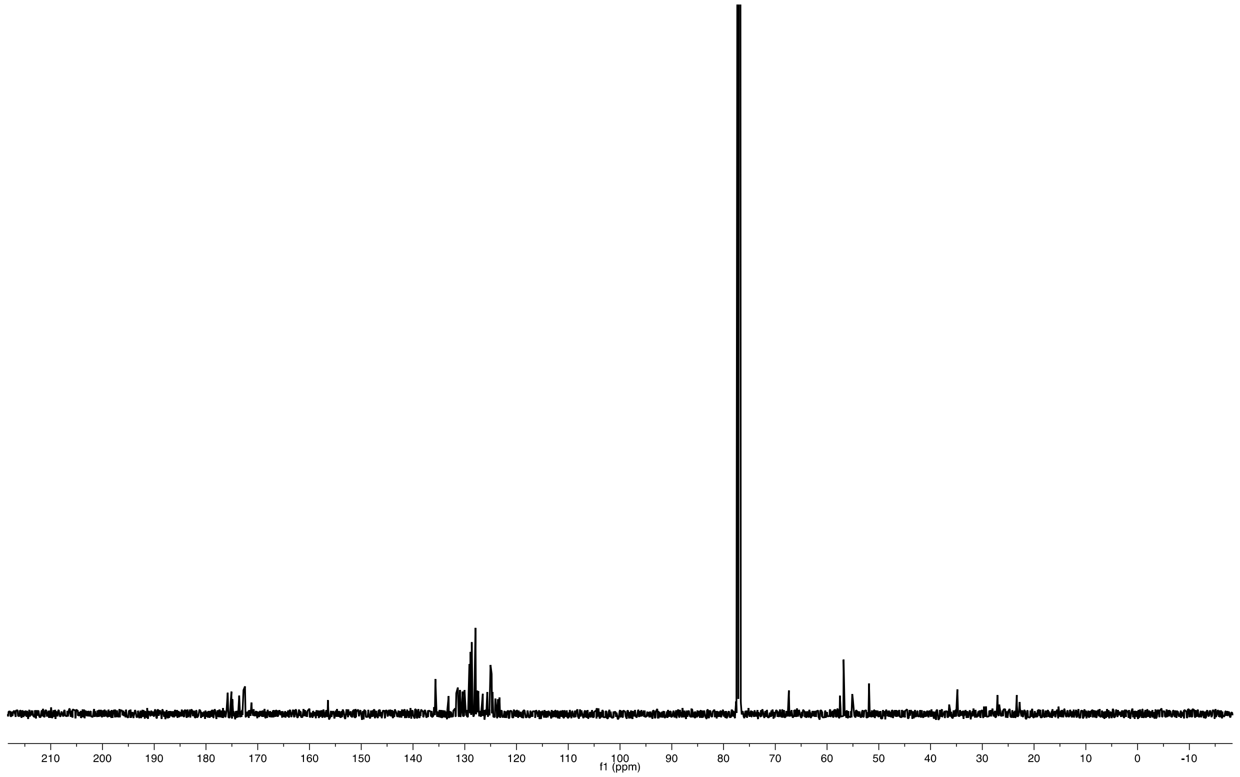

^{1}H NMR spectrum of Cbz(D-Phe)Aib$_4$(L-Pya)(D-Pya)OMe 12

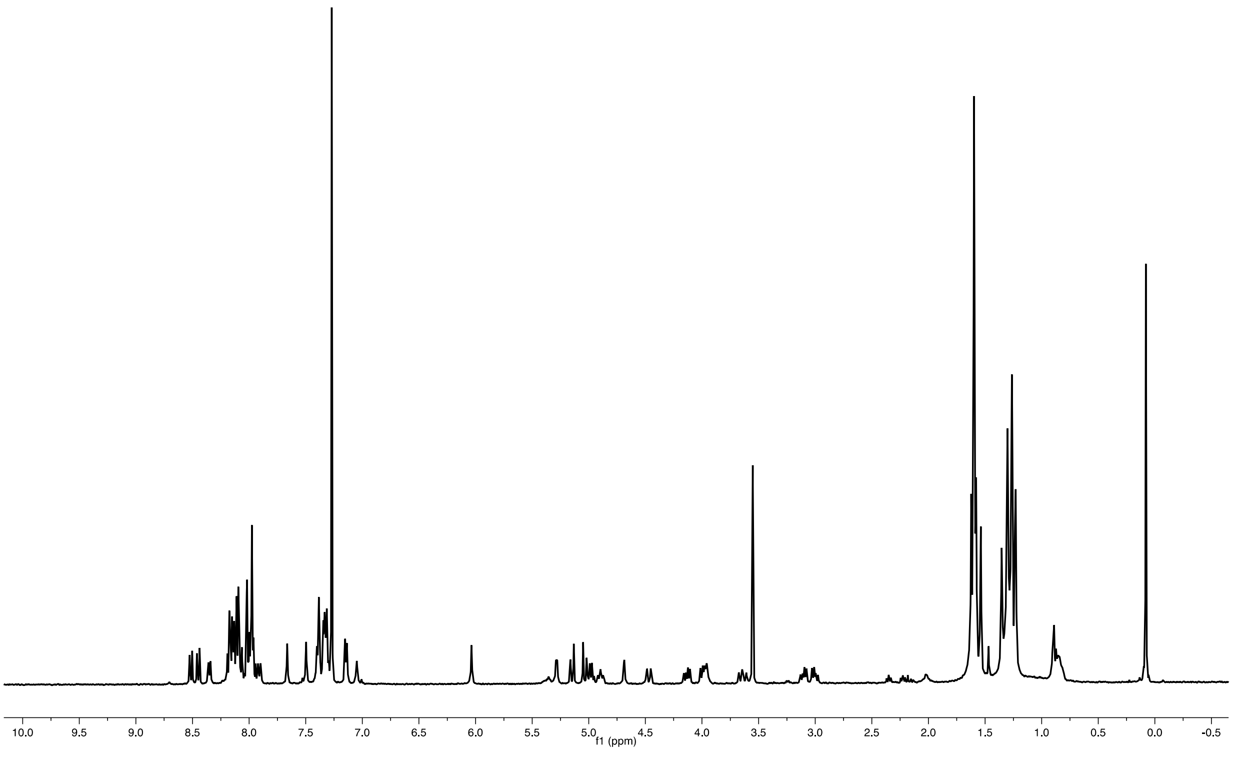

^{13}C NMR spectrum of Cbz(D-Phe)Aib$_4$(L-Pya)(D-Pya)OMe 12

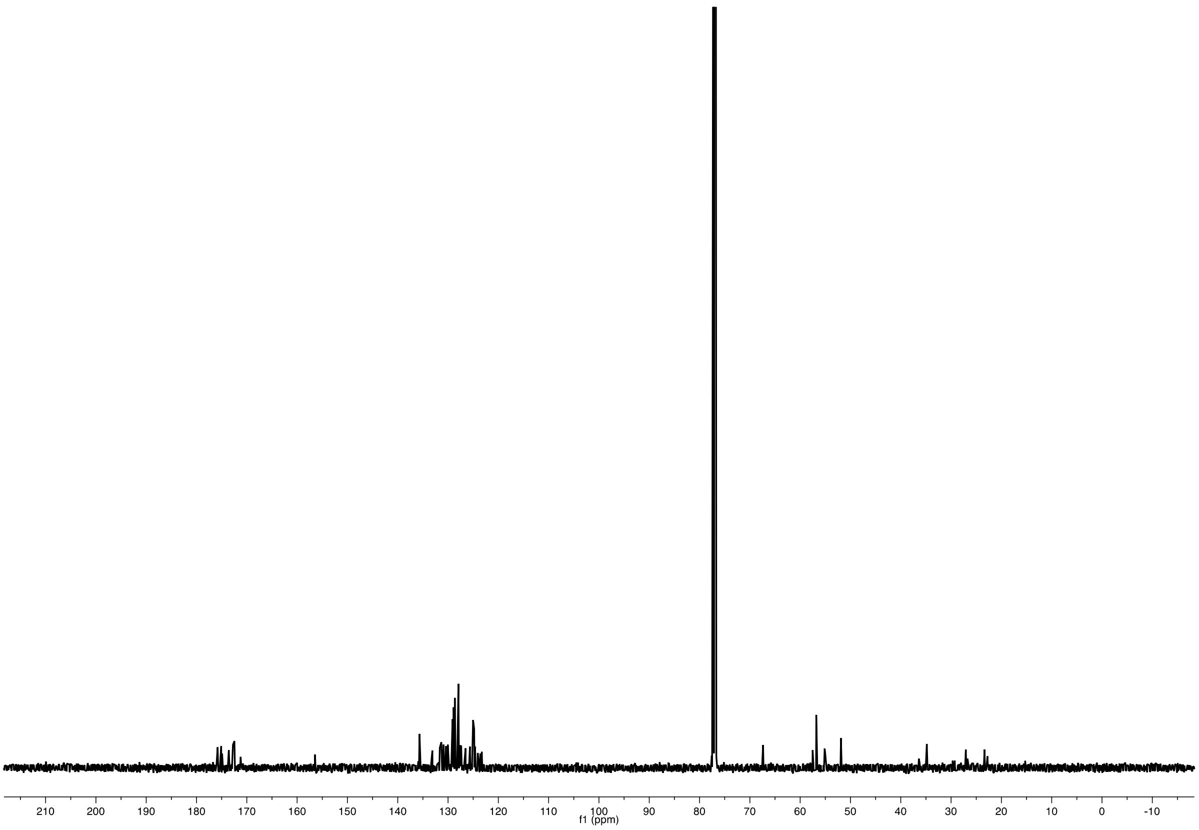

^{1}H NMR spectrum of Cbz(L-Phe)Aib$_4$(L-Pya)NHCH$_2$Pyr 13

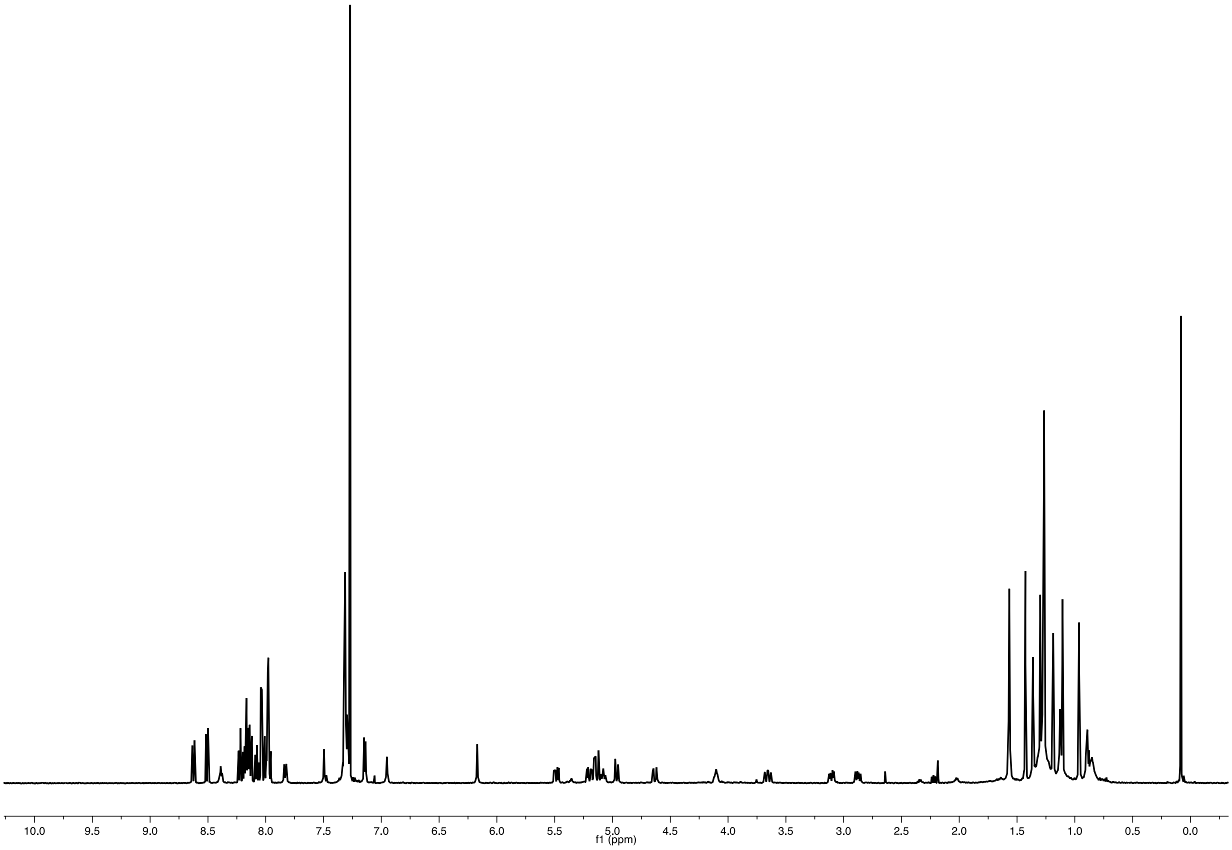

^{13}C NMR spectrum of Cbz(L-Phe)Aib$_4$(L-Pya)NHCH$_2$Pyr 13

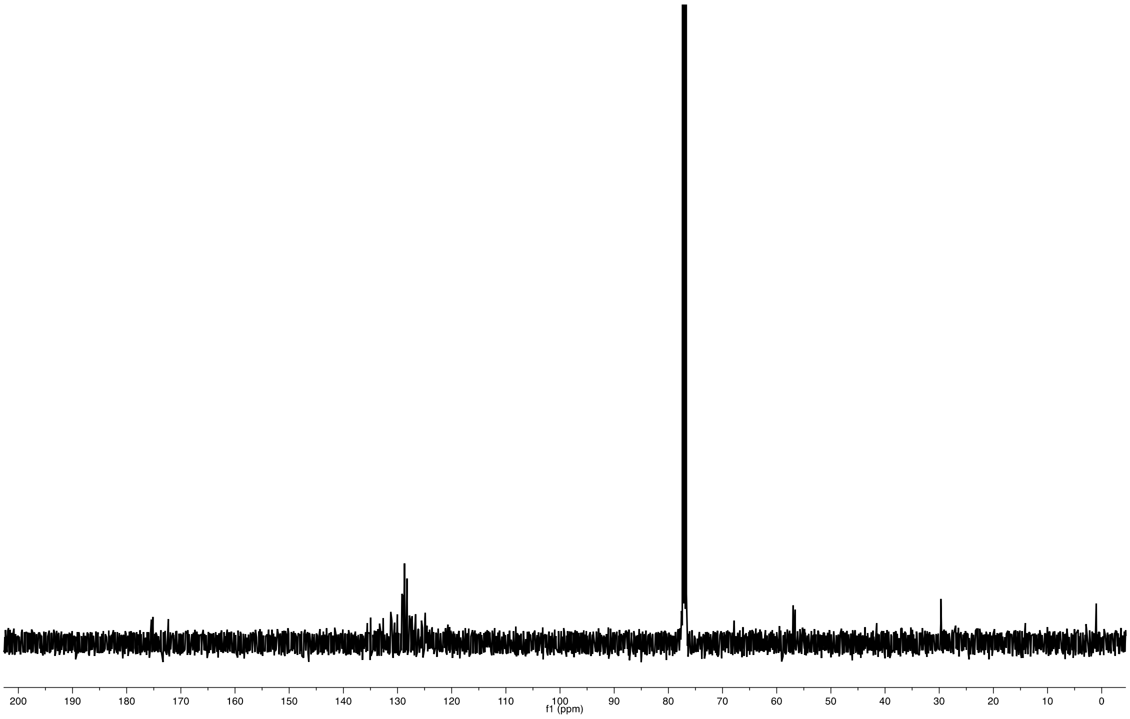

^{1}H NMR spectrum of Cbz(D-Phe)Aib$_4$(L-Pya)NHCH$_2$Pyr 14

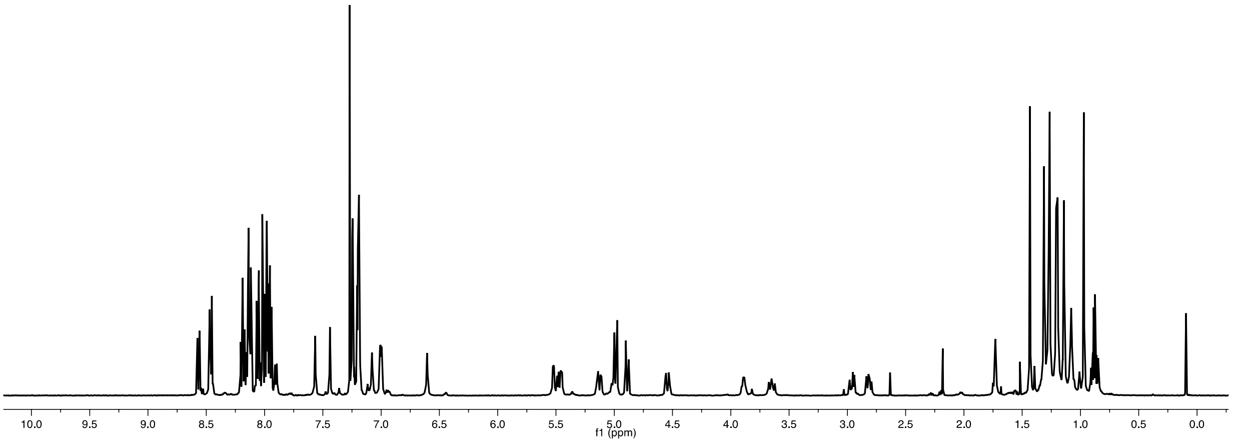

^{13}C NMR spectrum of Cbz(D-Phe)Aib$_4$(L-Pya)NHCH$_2$Pyr 14

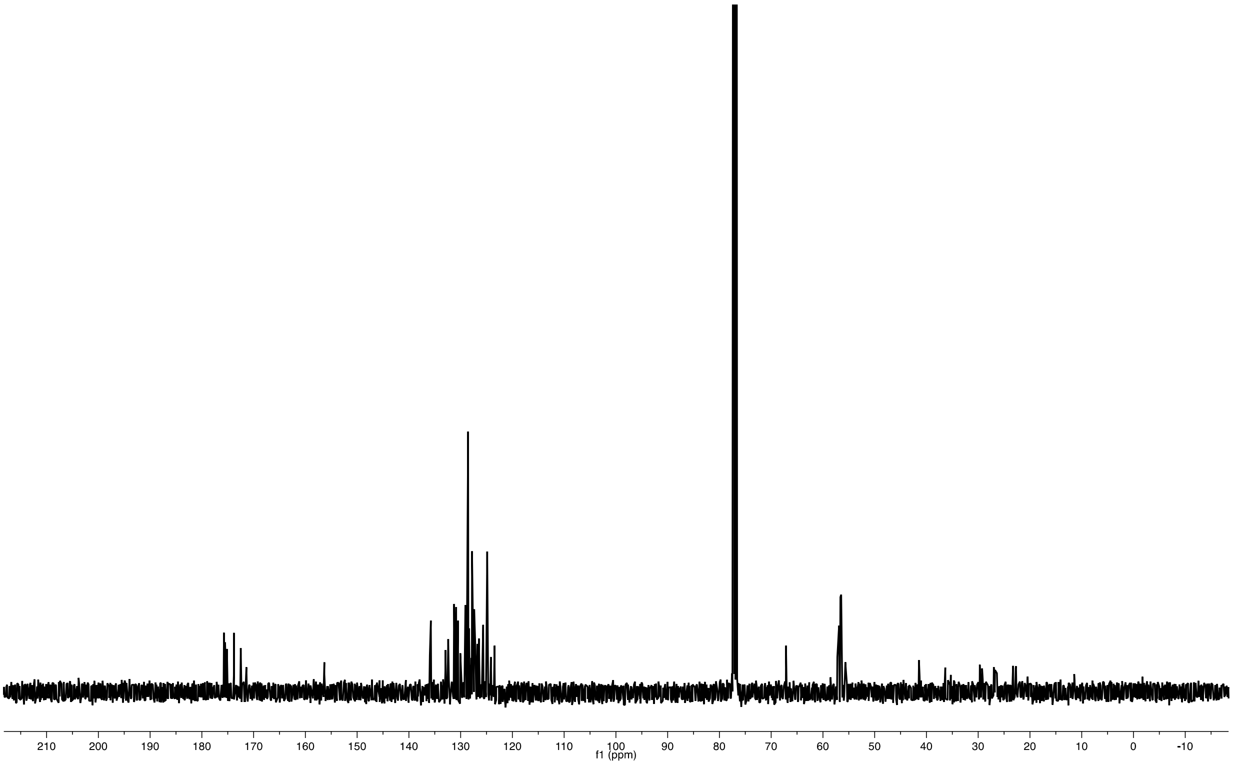

^{1}H NMR spectrum of (2*S*,3*R*)-2-azido-3-hydroxysuccinic acid 17

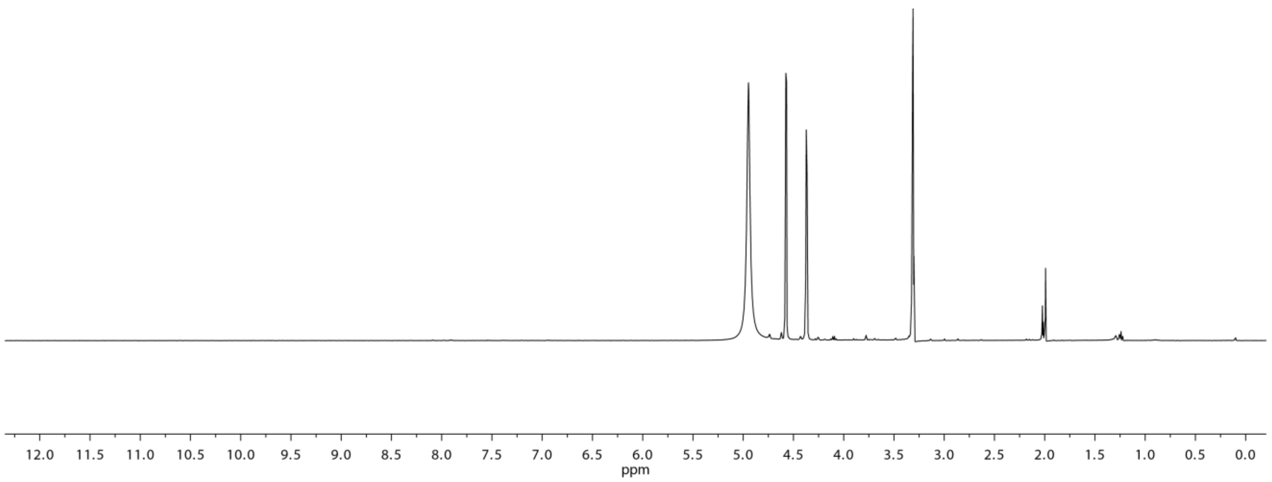

^{13}C NMR spectrum of (2*S*,3*R*)-2-azido-3-hydroxysuccinic acid 17

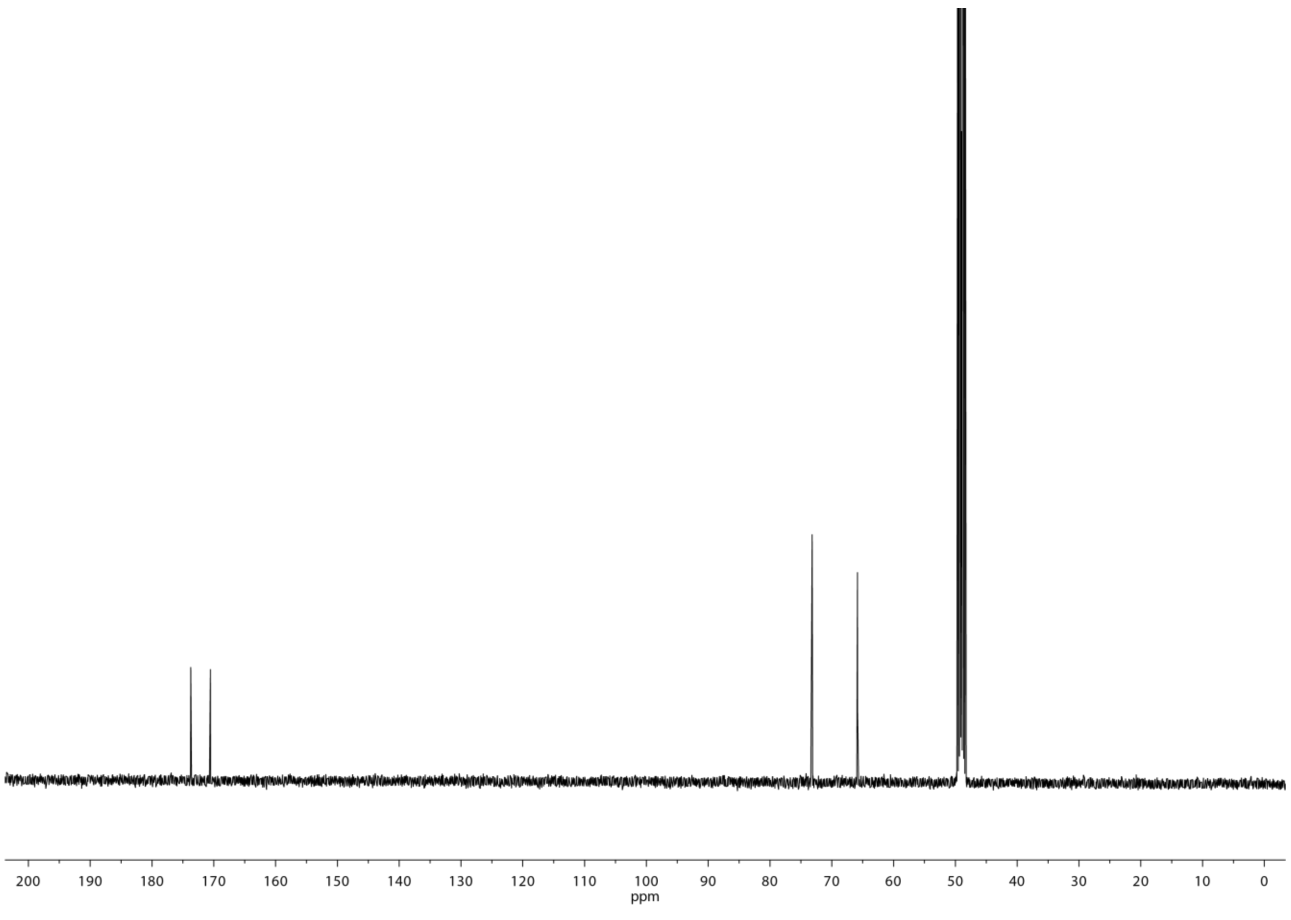

^{1}H NMR spectrum of (2S,3R)-bis(pyren-1-ylmethyl) 2-azido-3-hydroxysuccinate 18

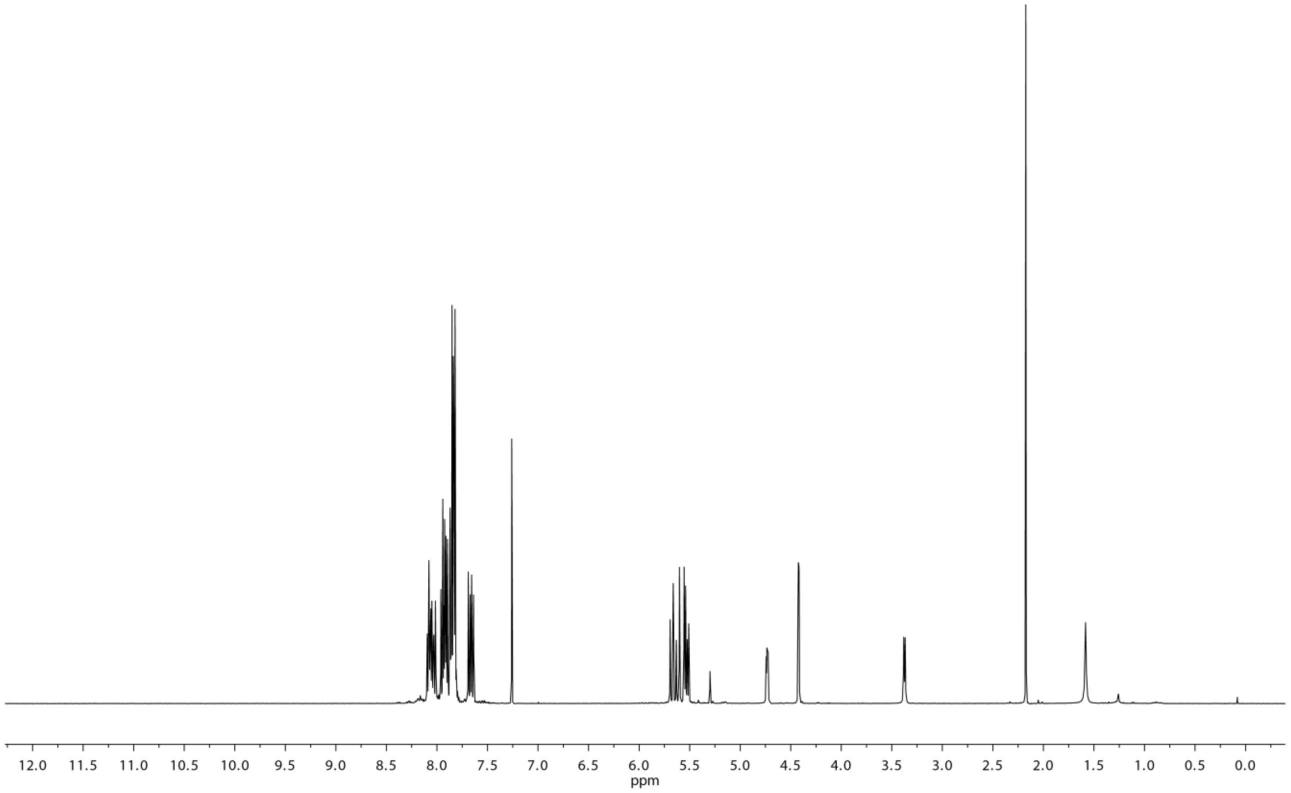

^{13}C NMR spectrum of (2S,3R)-bis(pyren-1-ylmethyl) 2-azido-3-hydroxysuccinate 18

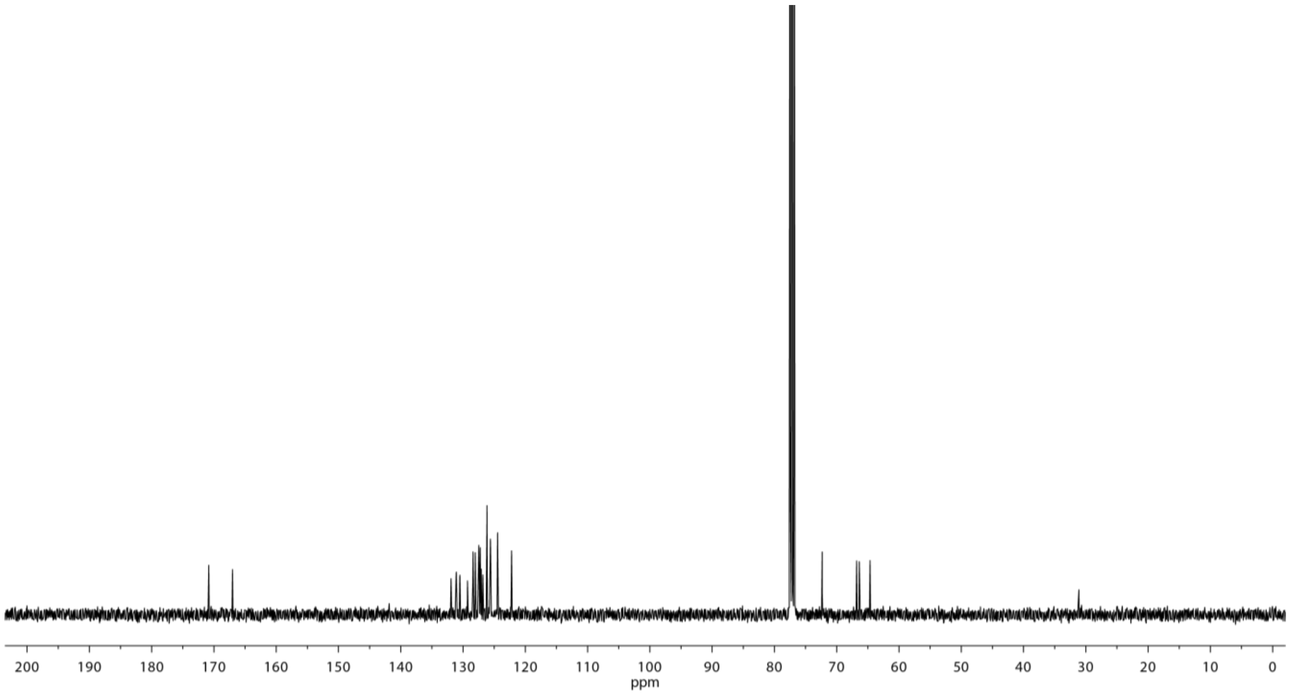

^{1}H NMR spectrum of (2R,3R)-bis(pyren-1-ylmethyl) 2-azido-3-hydroxysuccinate 19

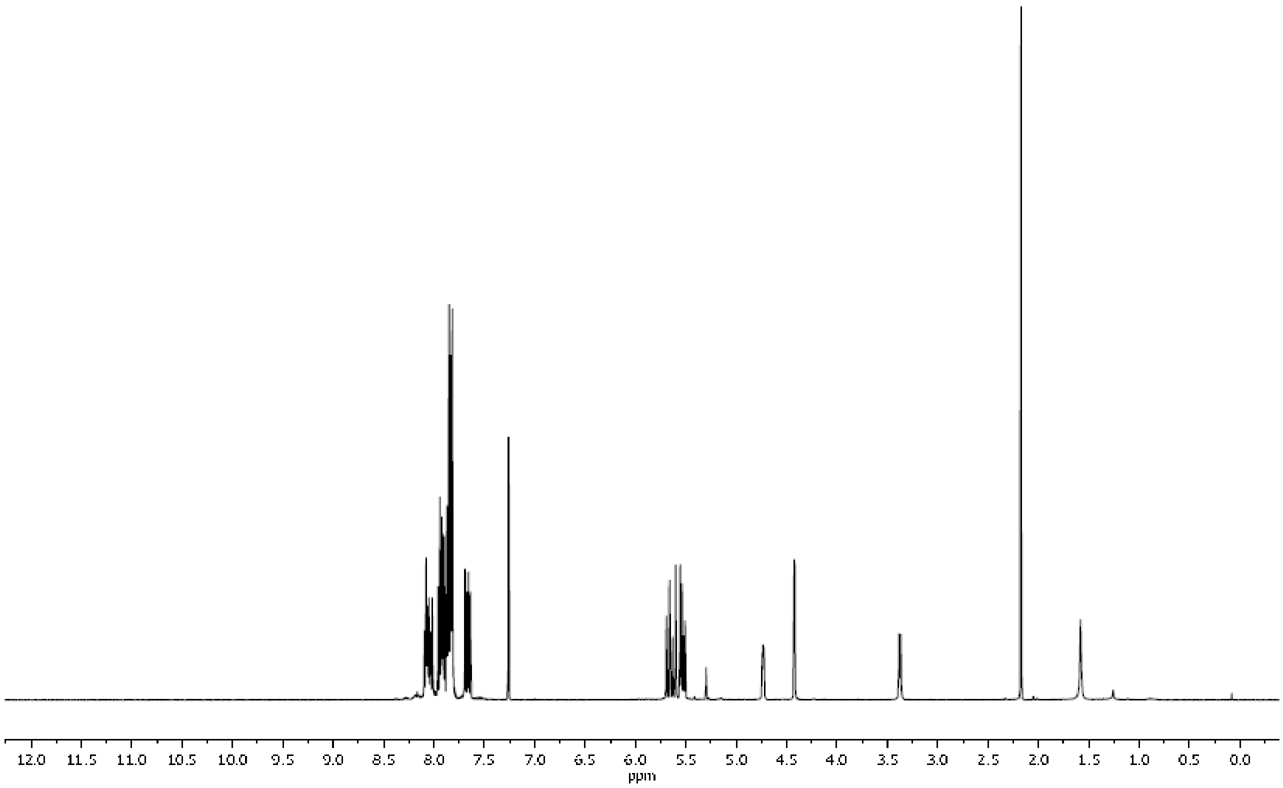

^{13}C NMR spectrum of (2R,3R)-bis(pyren-1-ylmethyl) 2-azido-3-hydroxysuccinate 19

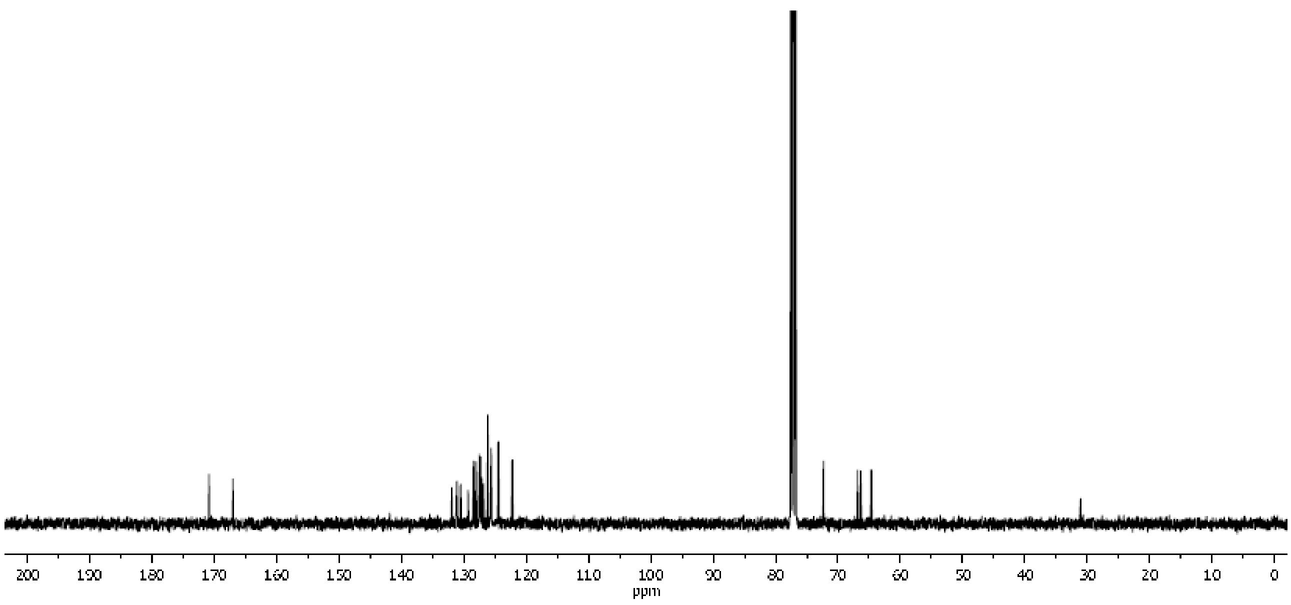

^{1}H NMR spectrum of Cbz(L-Phe)Aib$_4$(2*S*,3*R*-BisPyrSucc)OH 20

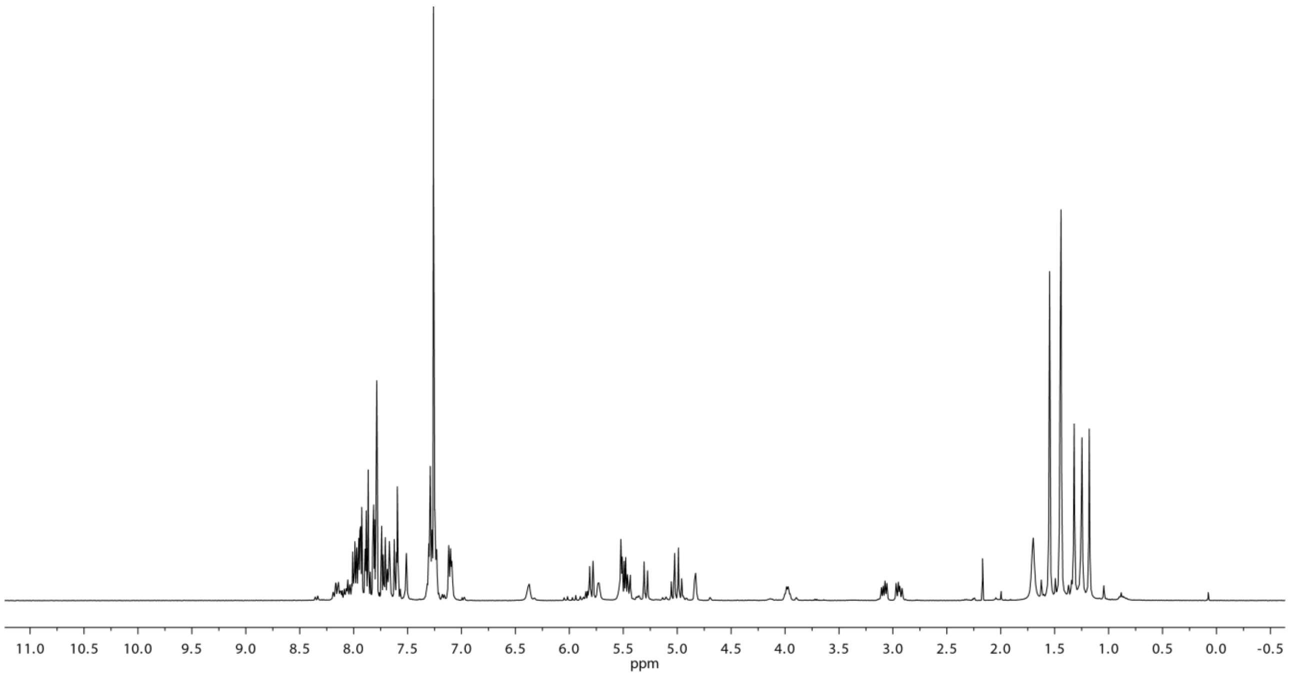

^{13}C NMR spectrum of Cbz(L-Phe)Aib$_4$(2*S*,3*R*-BisPyrSucc)OH 20

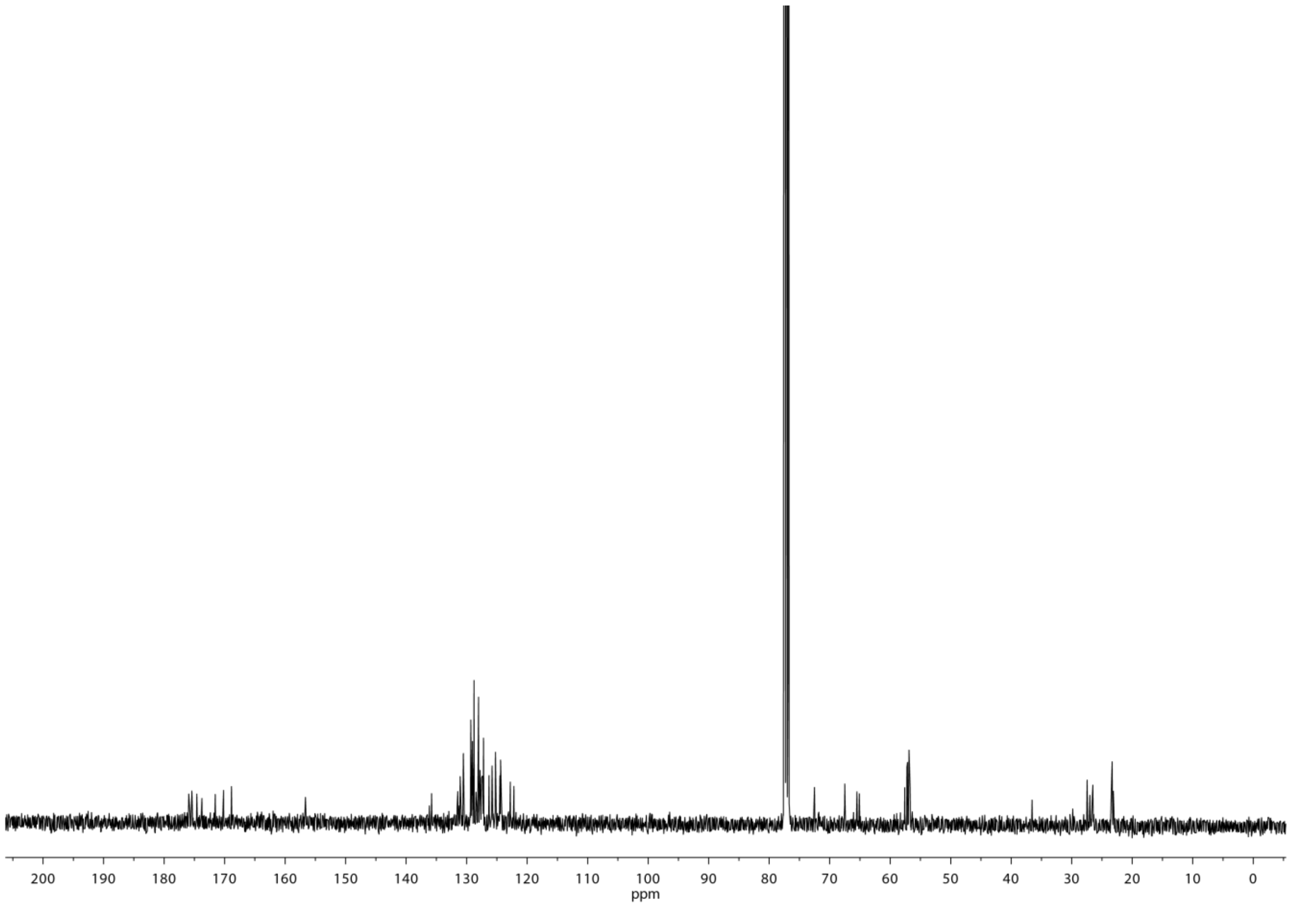

^{1}H NMR spectrum of Cbz(D-Phe)Aib$_4$(2*S*,3*R*-BisPyrSucc)OH 21

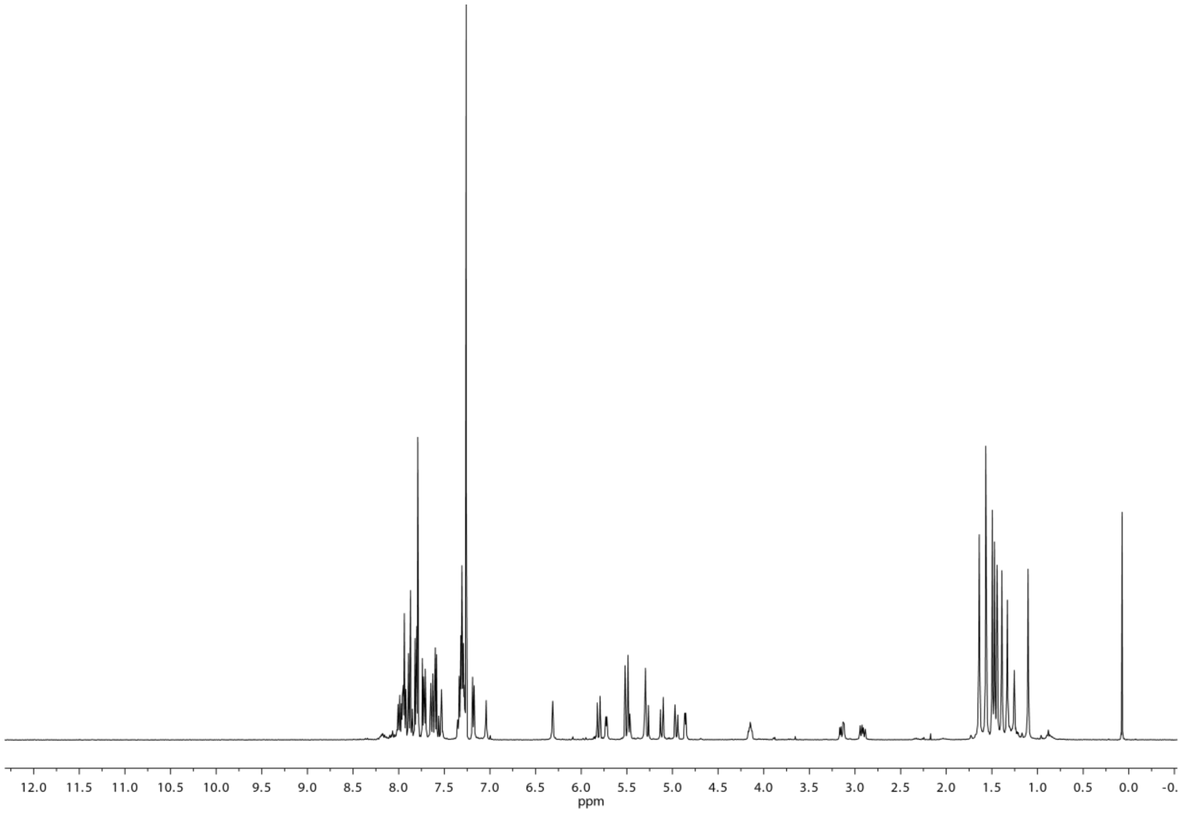

^{13}C NMR spectrum of Cbz(D-Phe)Aib$_4$(2*S*,3*R*-BisPyrSucc)OH 21

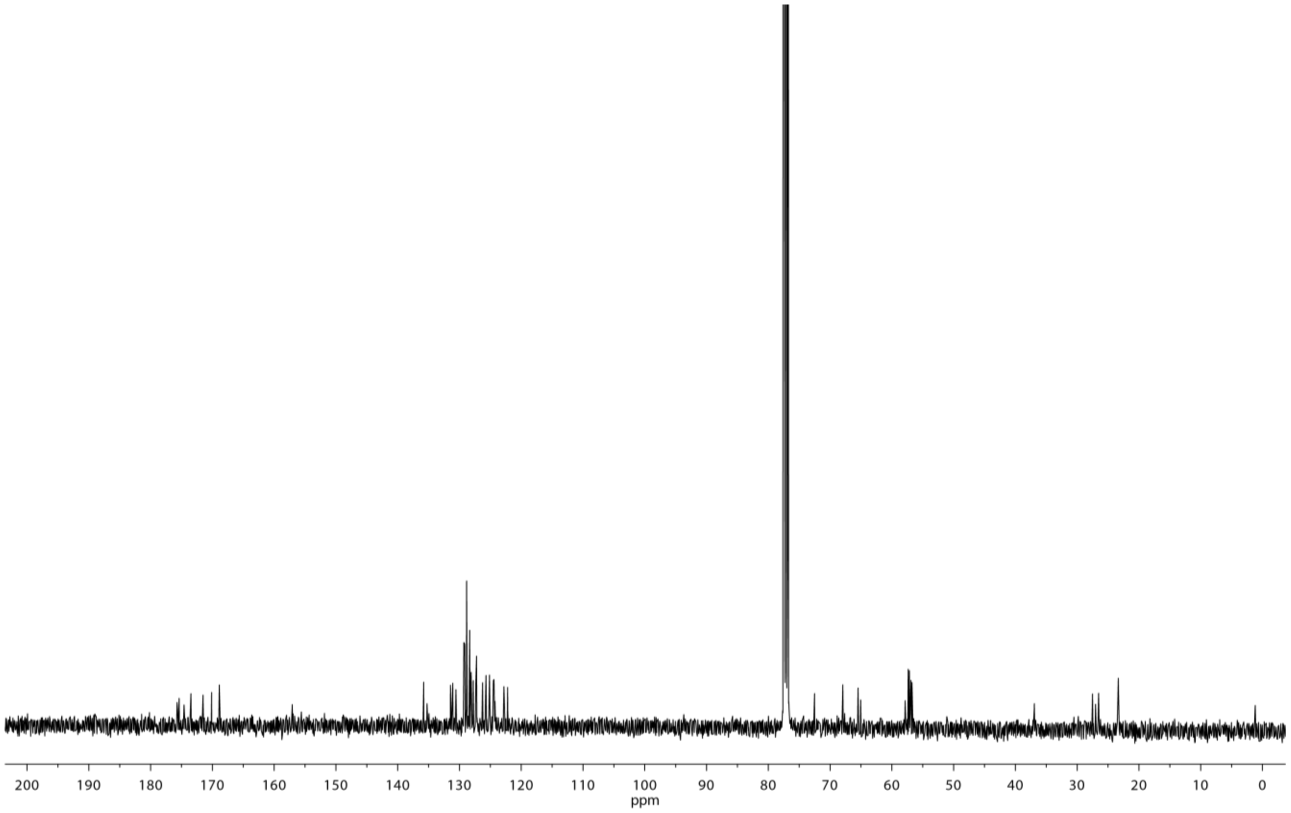

^{1}H NMR spectrum of Cbz(L-Phe)Aib$_4$(2*R*,3*R*-BisPyrSucc)OH 22

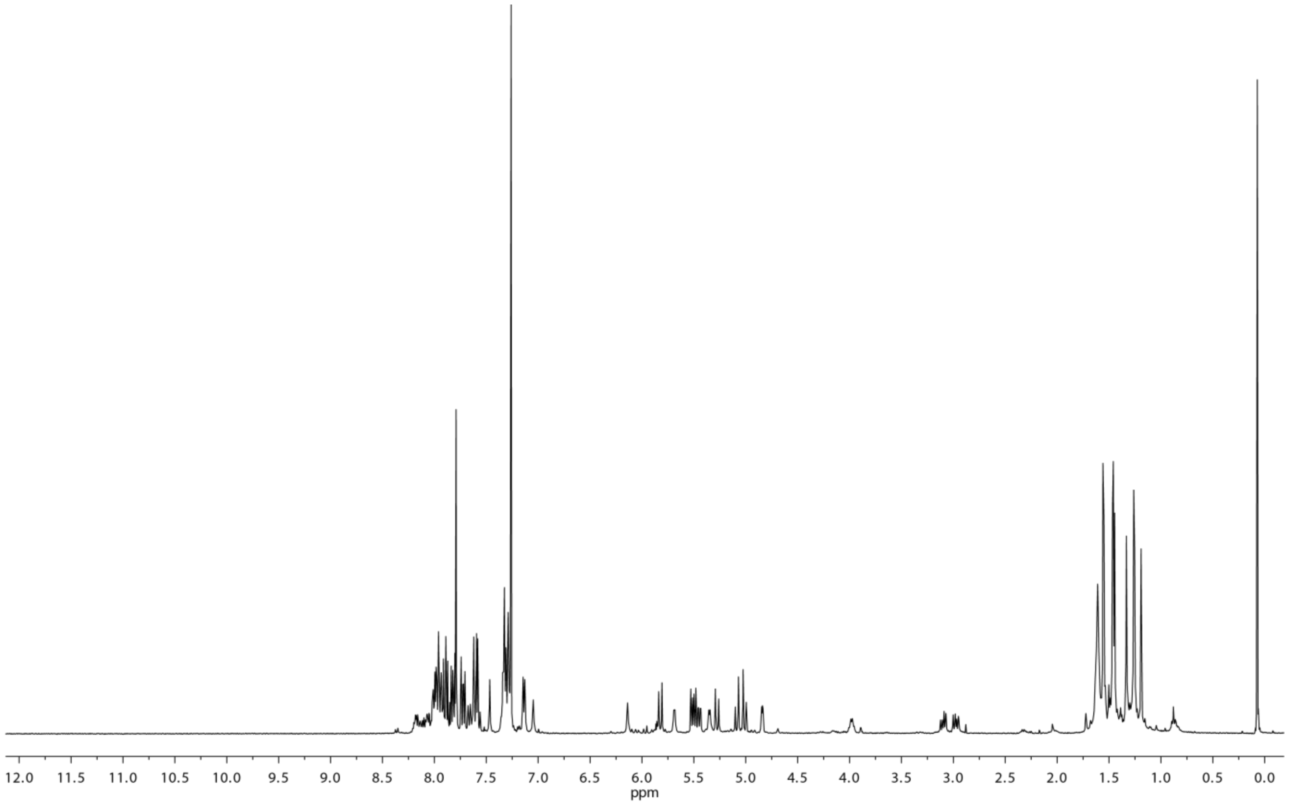

^{13}C NMR spectrum of Cbz(L-Phe)Aib$_4$(2*R*,3*R*-BisPyrSucc)OH 22

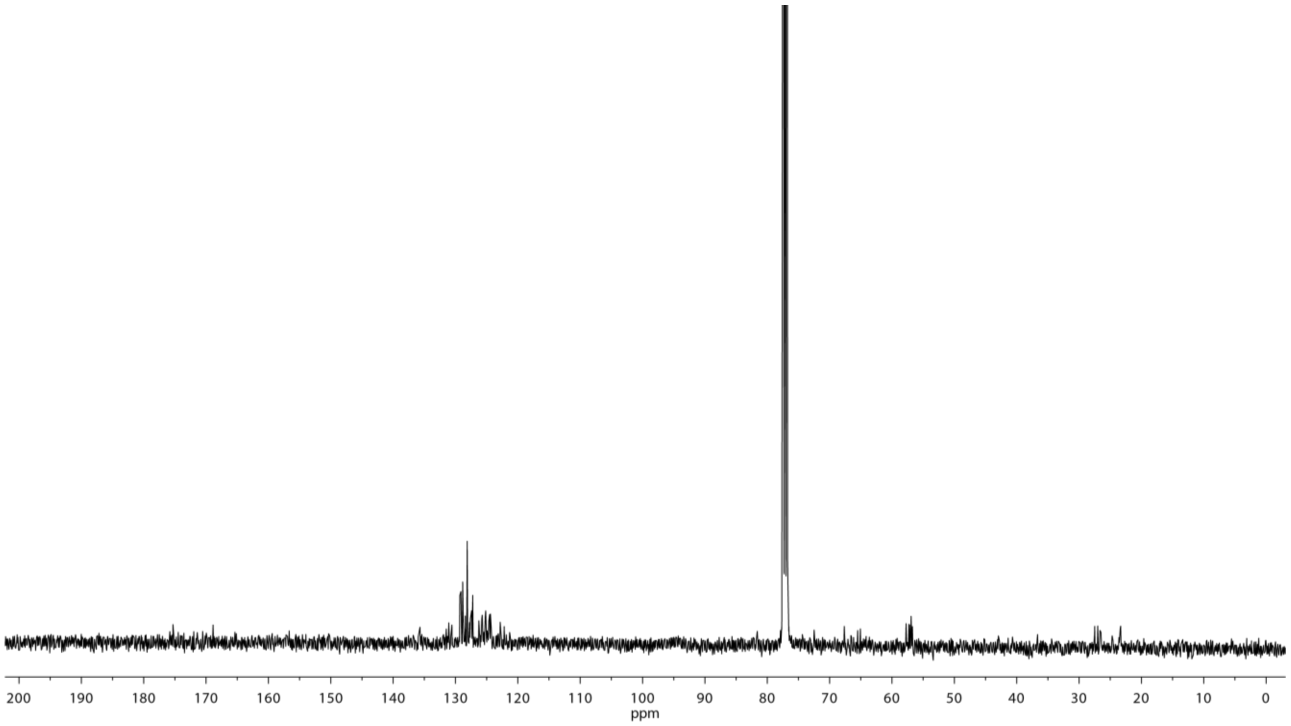

^{1}H NMR spectrum of Cbz(D-Phe)Aib$_4$(2*R*,3*R*-BisPyrSucc)OH 23

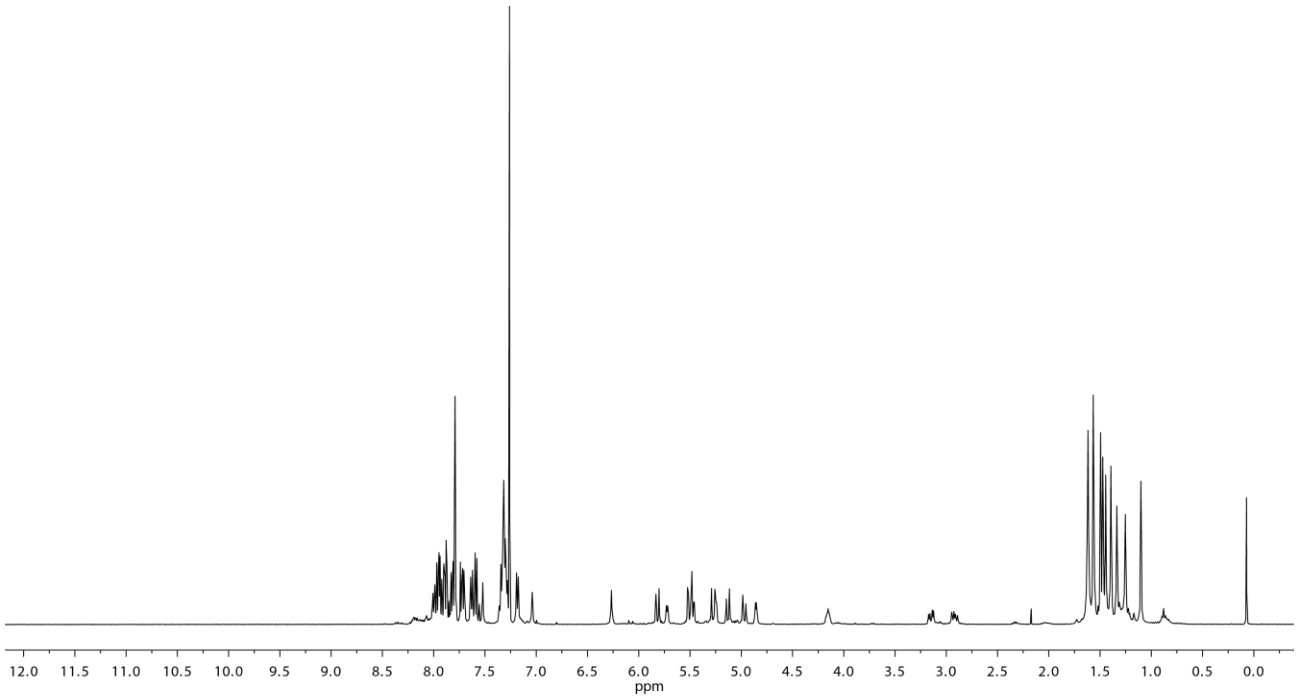

^{13}C NMR spectrum of Cbz(D-Phe)Aib$_4$(2*R*,3*R*-BisPyrSucc)OH 23

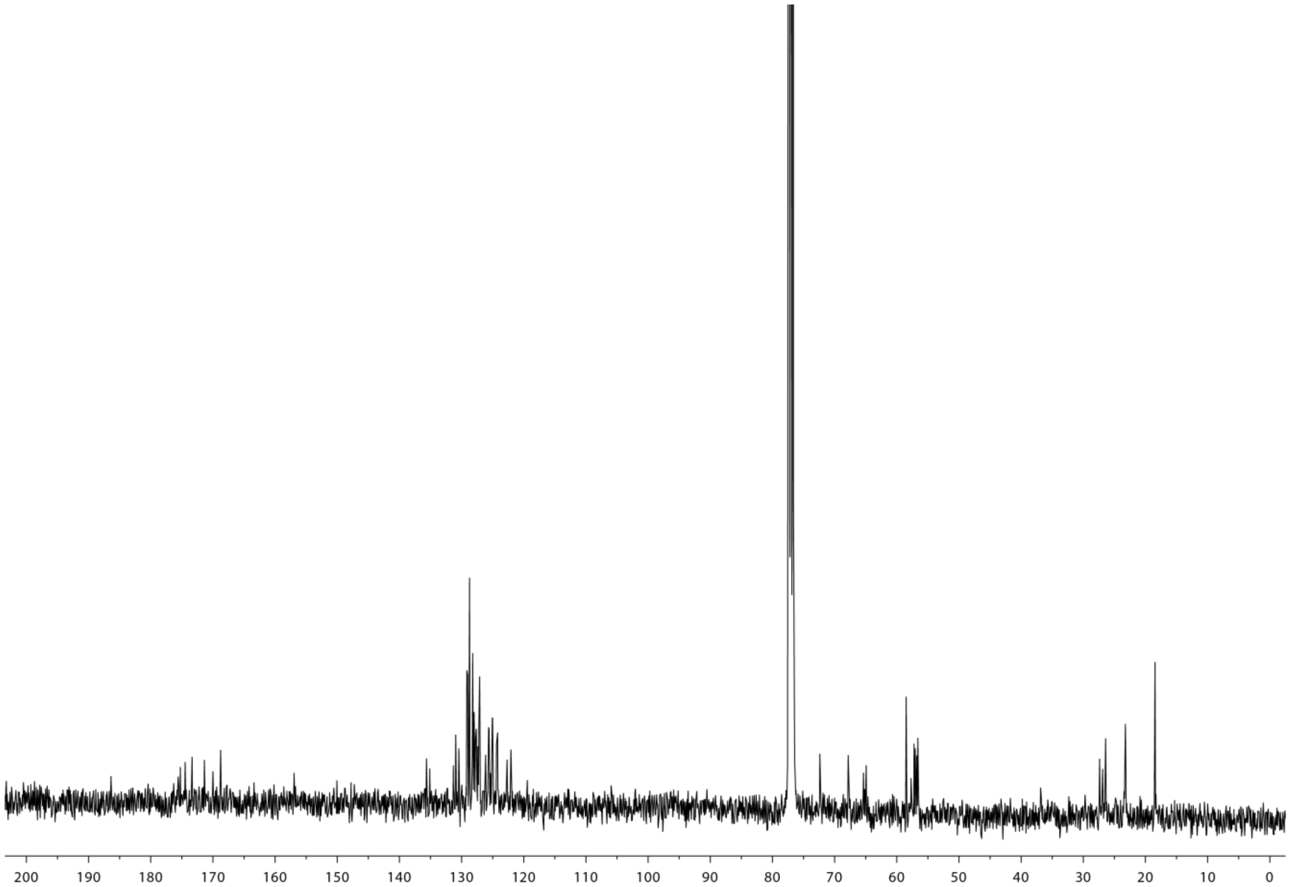

^{1}H NMR spectrum of *p*-(1-pyrenylether) benzaldehyde 25

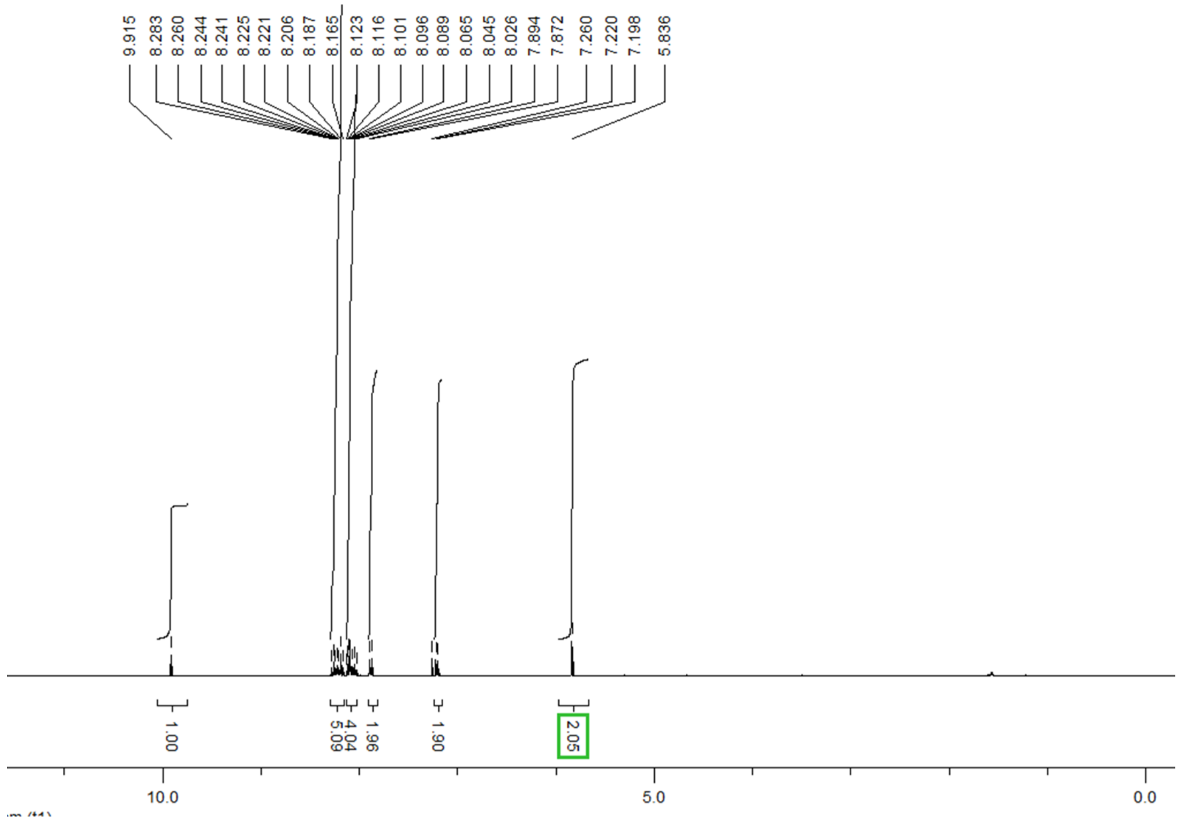

^{13}C NMR spectrum of *p*-(1-pyrenylether) benzaldehyde 25

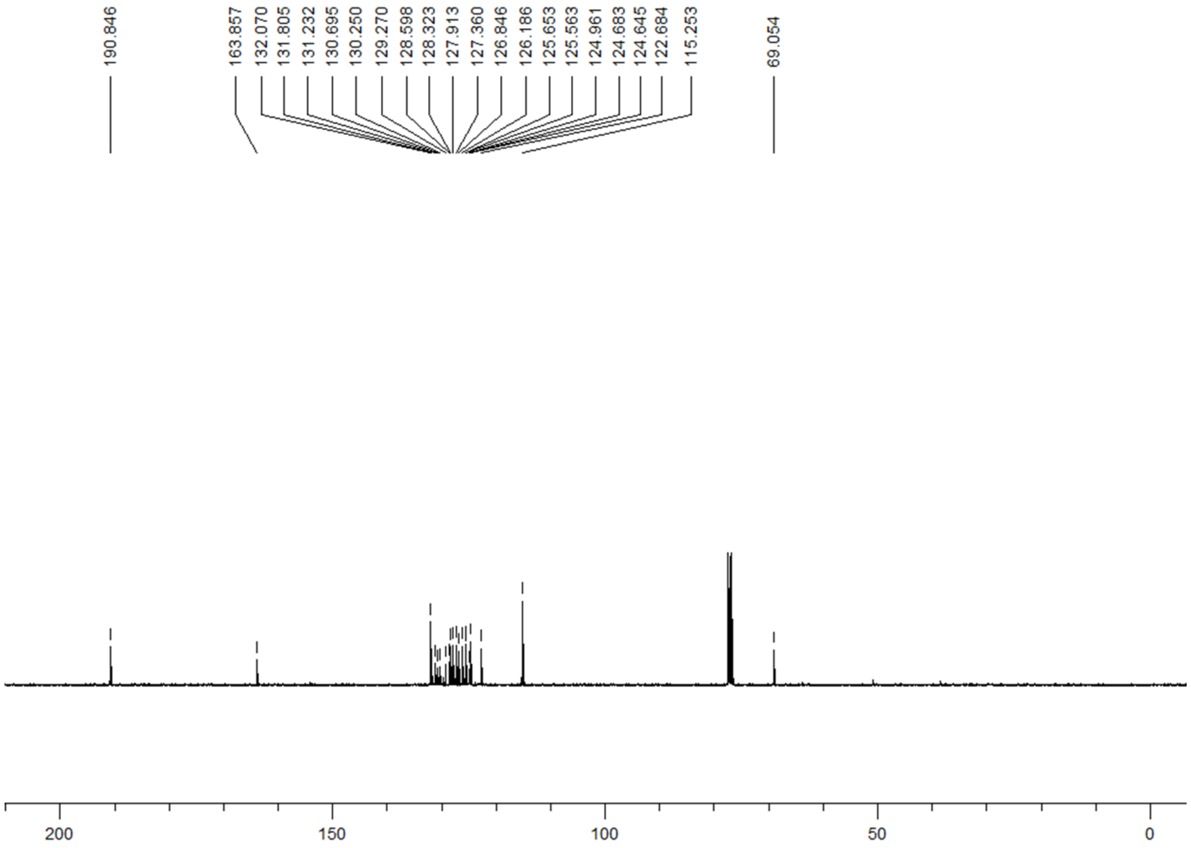

^1H NMR spectrum (400 MHz, DMSO-d_6, 300 K) of (1*S*,2*S*)-1,2-bis(4-(pyren-1-ylmethoxy)phenyl)ethane-1,2-diamine dihydrochloride 28

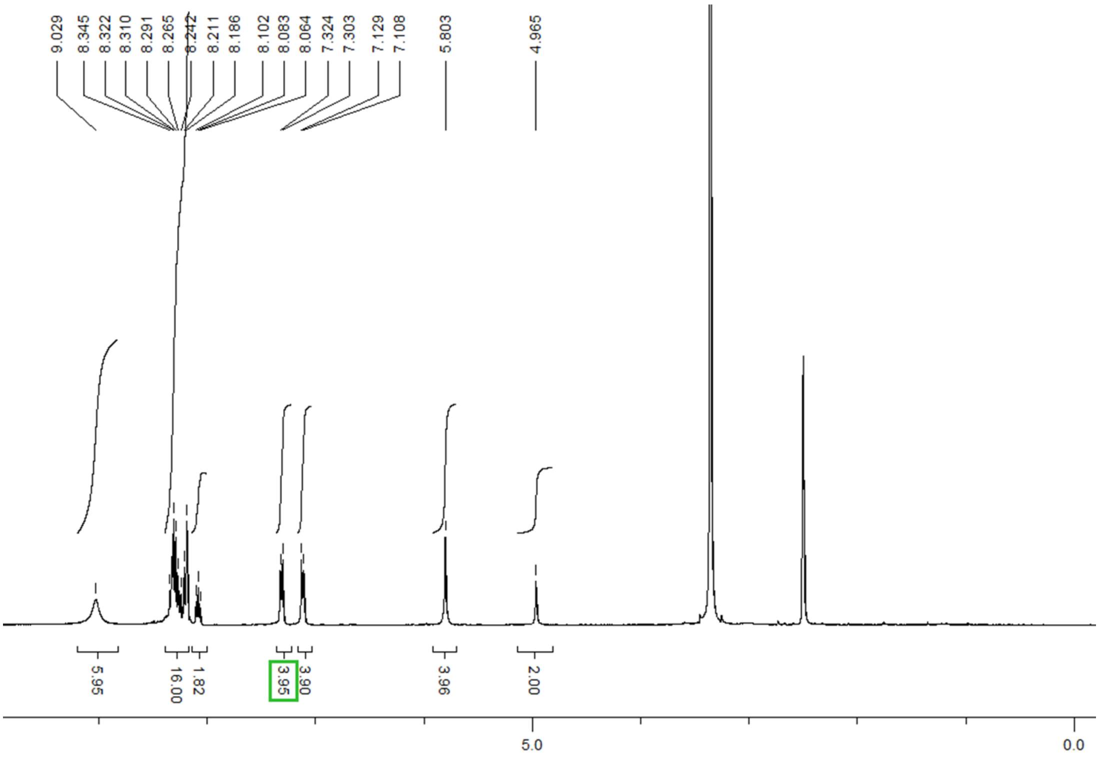

^{13}C NMR spectrum (75 MHz, DMSO-d_6, 300 K) of (1*S*,2*S*)-1,2-bis(4-(pyren-1-ylmethoxy)phenyl)ethane-1,2-diamine dihydrochloride 28

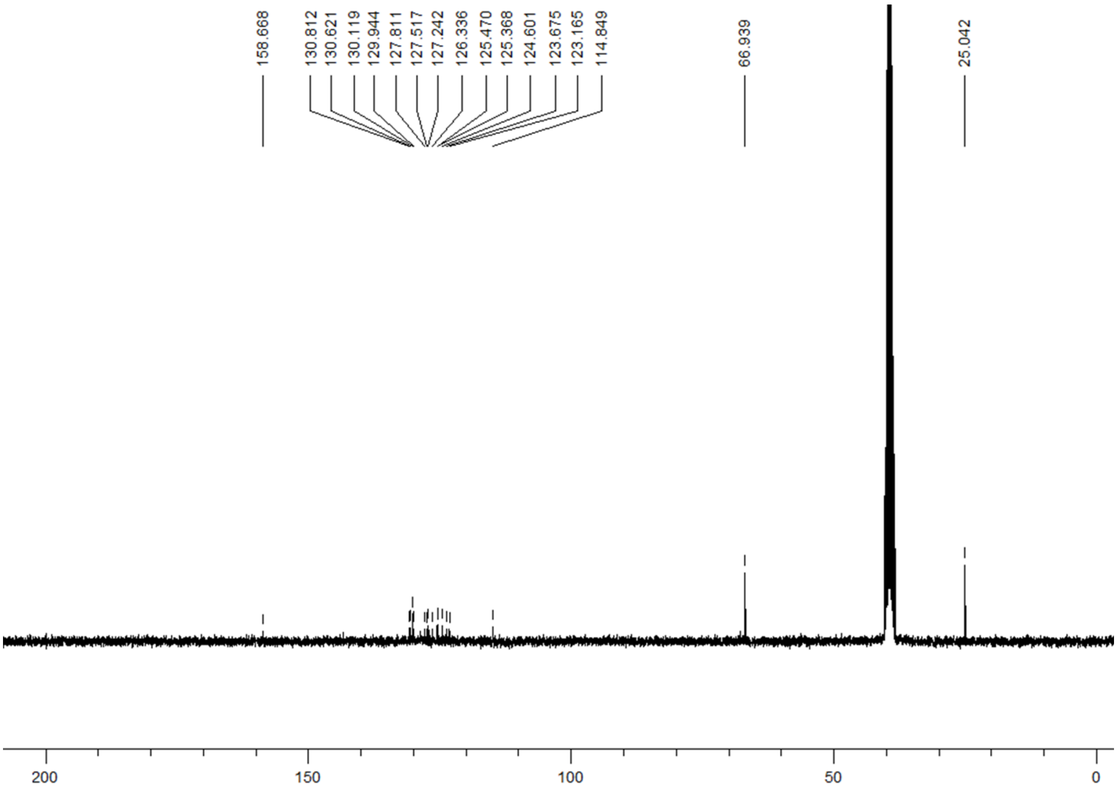

^{1}H NMR spectrum of Cbz(L-Phe)Aib$_4$(*S*,*S*-BisPyrEt)NH$_2$ 29

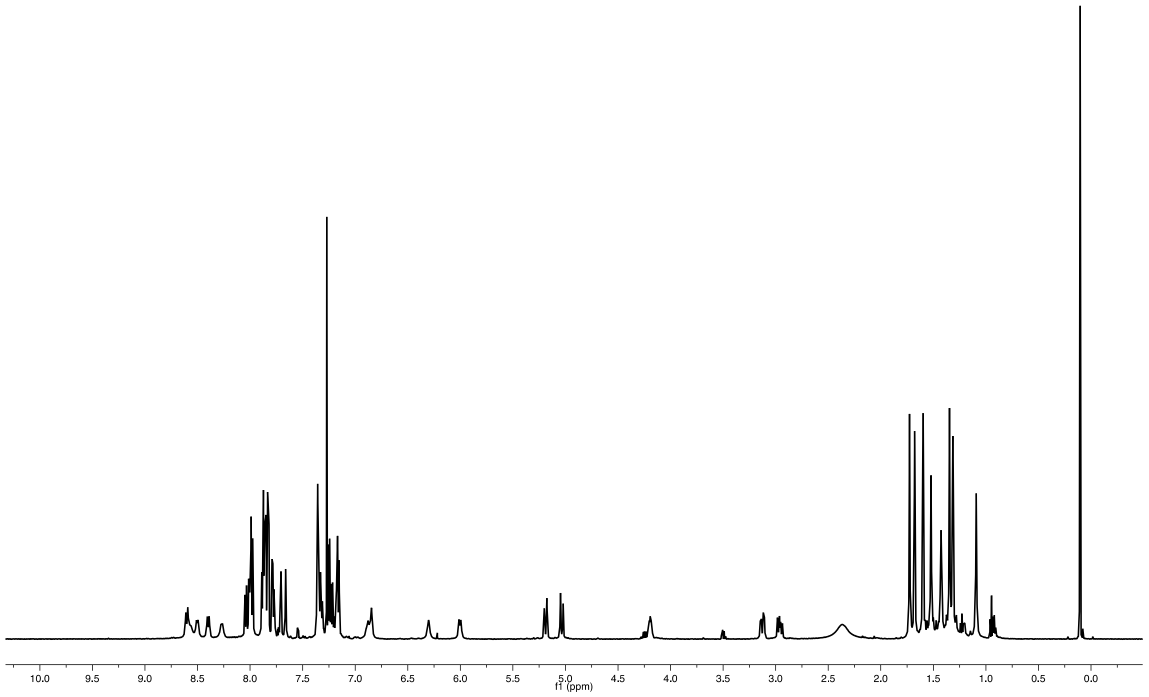

^{13}C NMR spectrum of Cbz(L-Phe)Aib$_4$(*S*,*S*-BisPyrEt)NH$_2$ 29

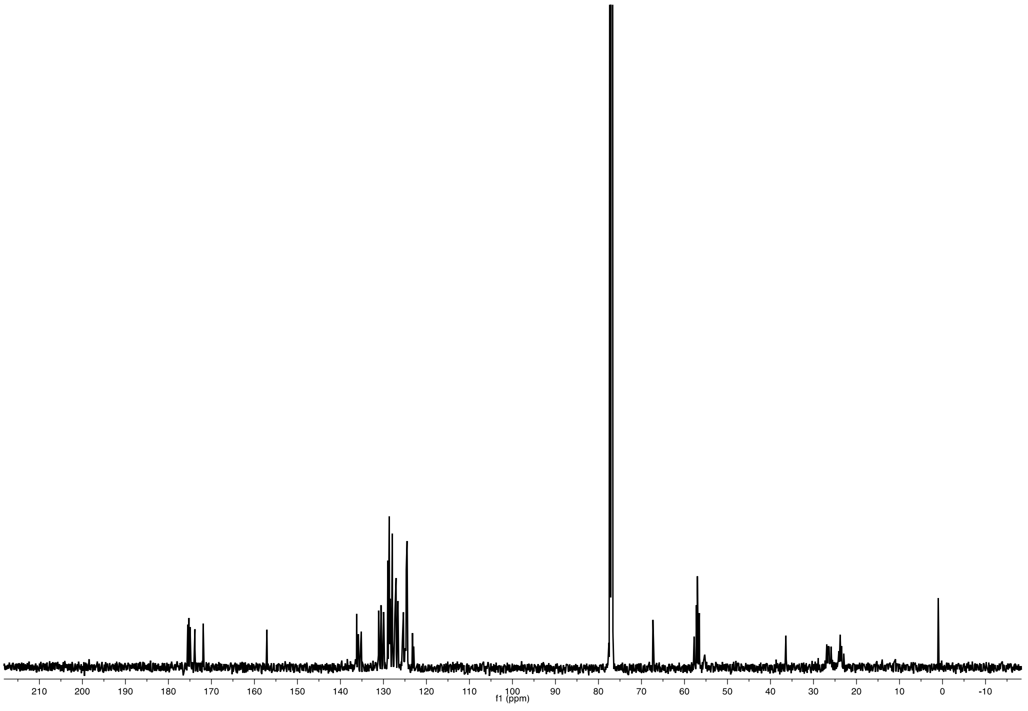

^{1}H NMR spectrum of Cbz(D-Phe)Aib$_4$(*S*,*S*-BisPyrEt)NH$_2$ 30

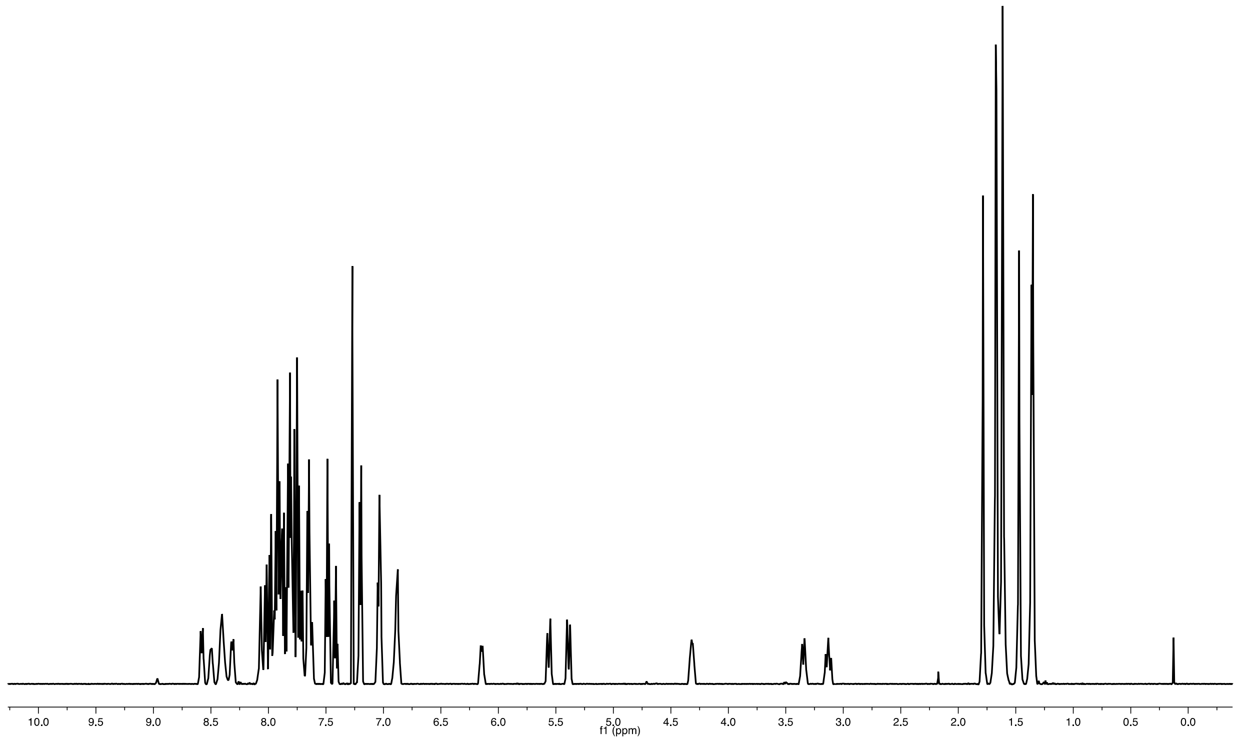

^{13}C NMR spectrum of Cbz(D-Phe)Aib$_4$(*S*,*S*-BisPyrEt)NH$_2$ 30

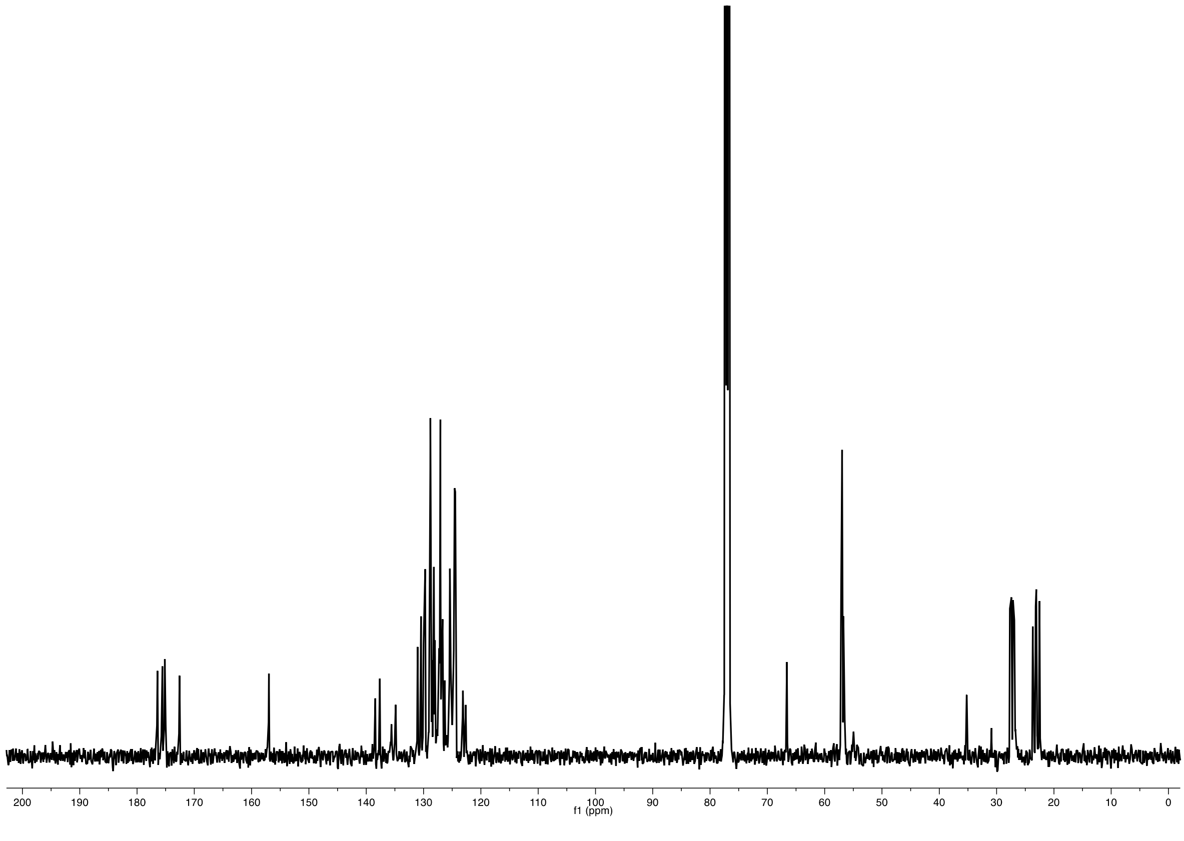

^{1}H NMR spectrum of Cbz(L-Phe)Aib$_4$(*S*,*S*-BisPhenPyrEt)NH$_2$ 31

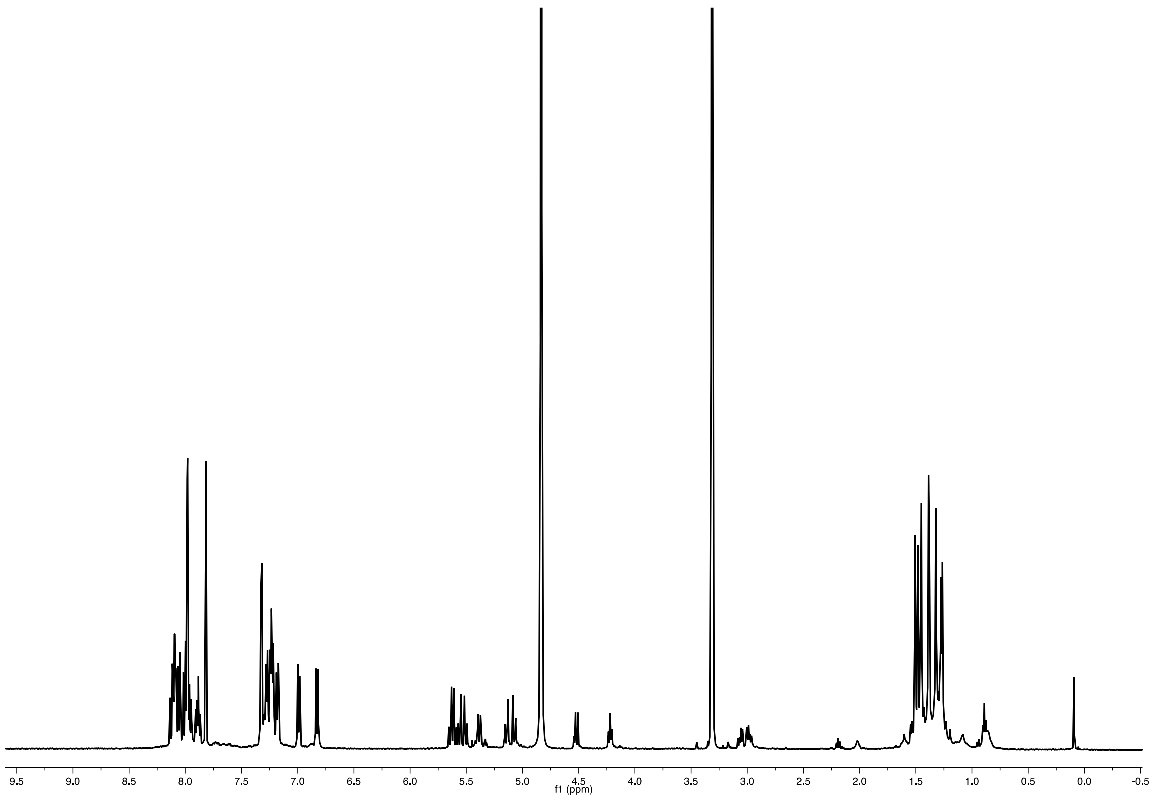

^{13}C NMR spectrum of Cbz(L-Phe)Aib$_4$(*S*,*S*-BisPhenPyrEt)NH$_2$ 31

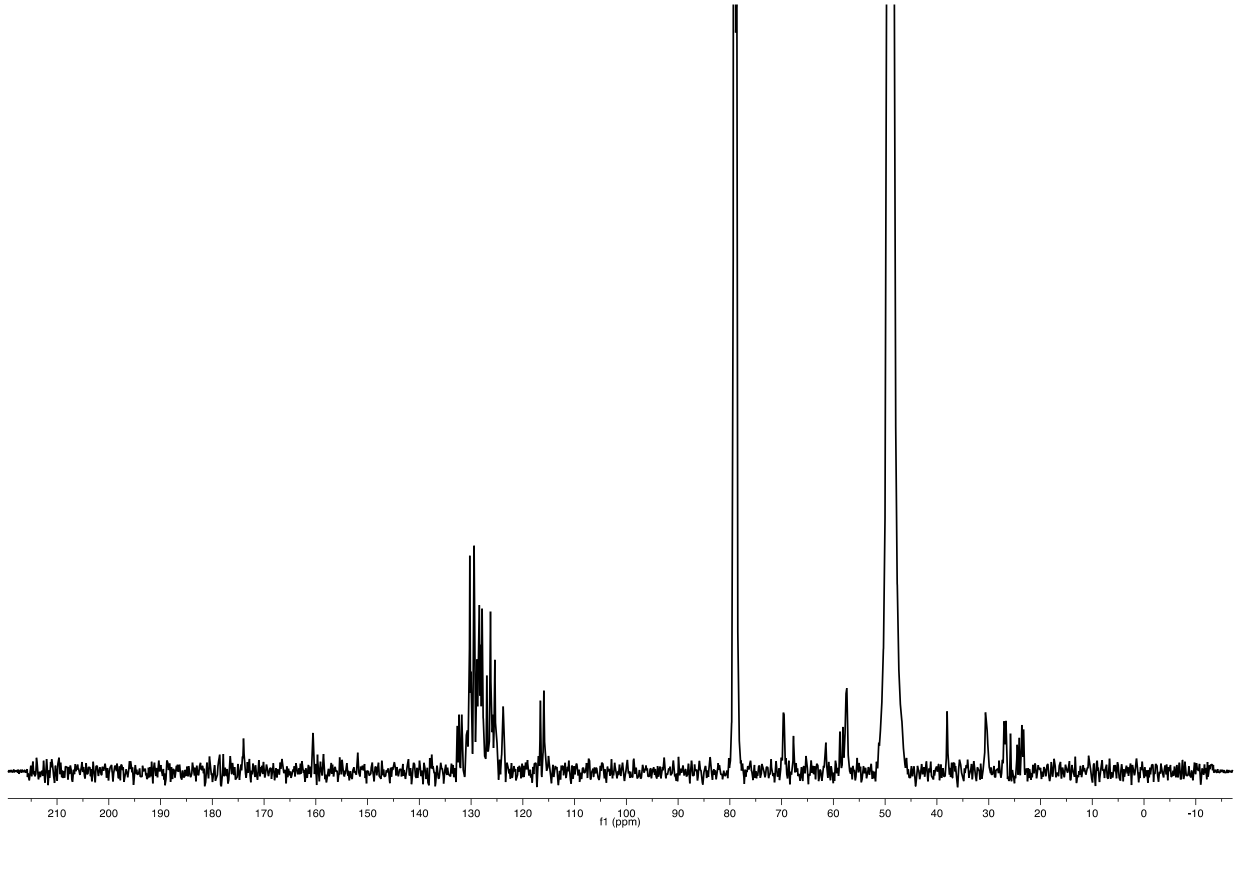

^{1}H NMR spectrum of Cbz(D-Phe)Aib$_4$(*S*,*S*-BisPhenPyrEt)NH$_2$ 32

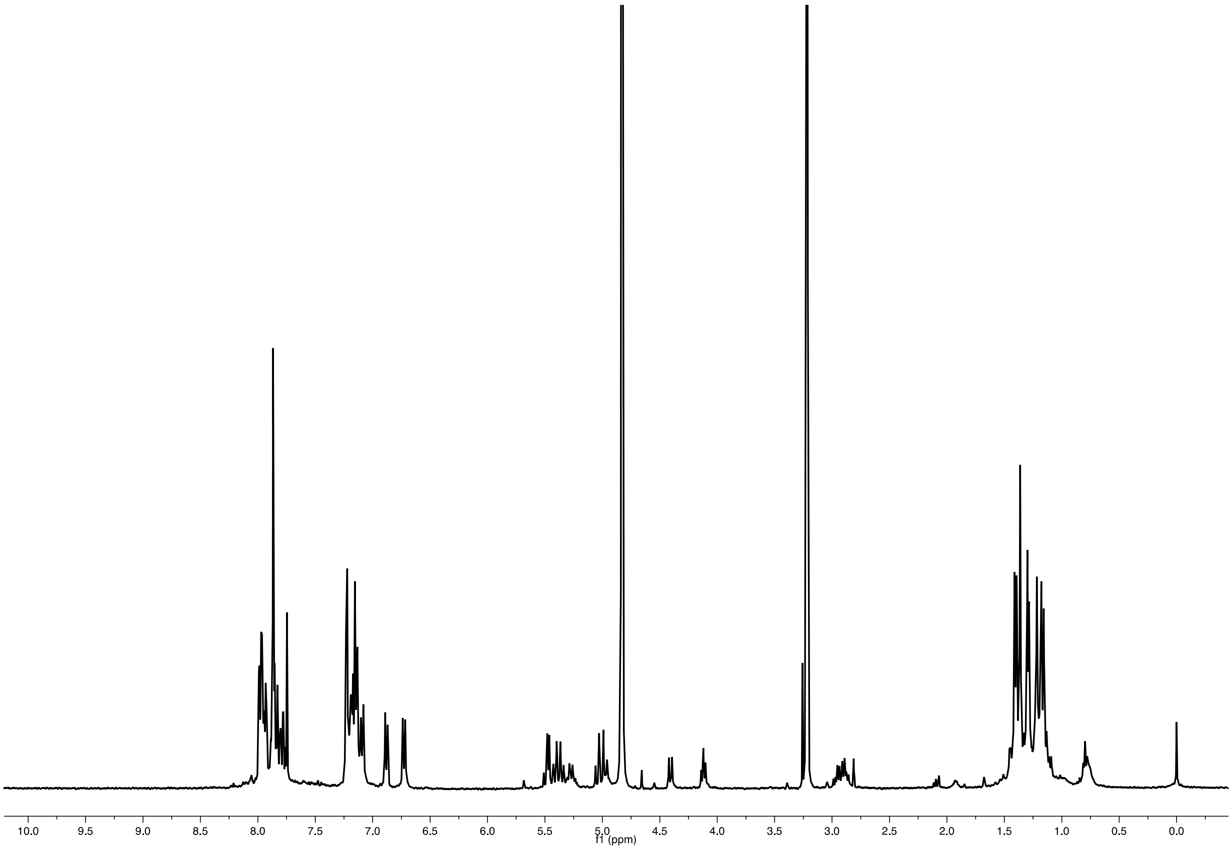

^{13}C NMR spectrum of Cbz(D-Phe)Aib$_4$(*S*,*S*-BisPhenPyrEt)NH$_2$ 32

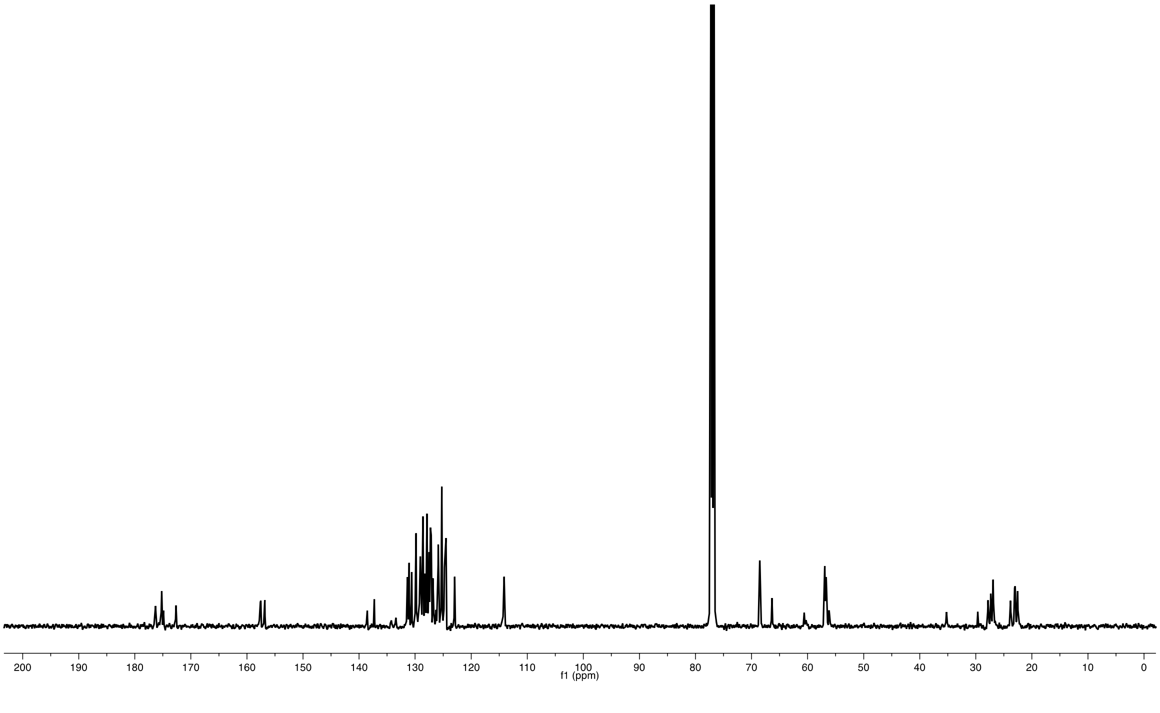

^{1}H NMR spectrum of N_3Aib_4(*S,S*-BisPyrEt)NHAc 43

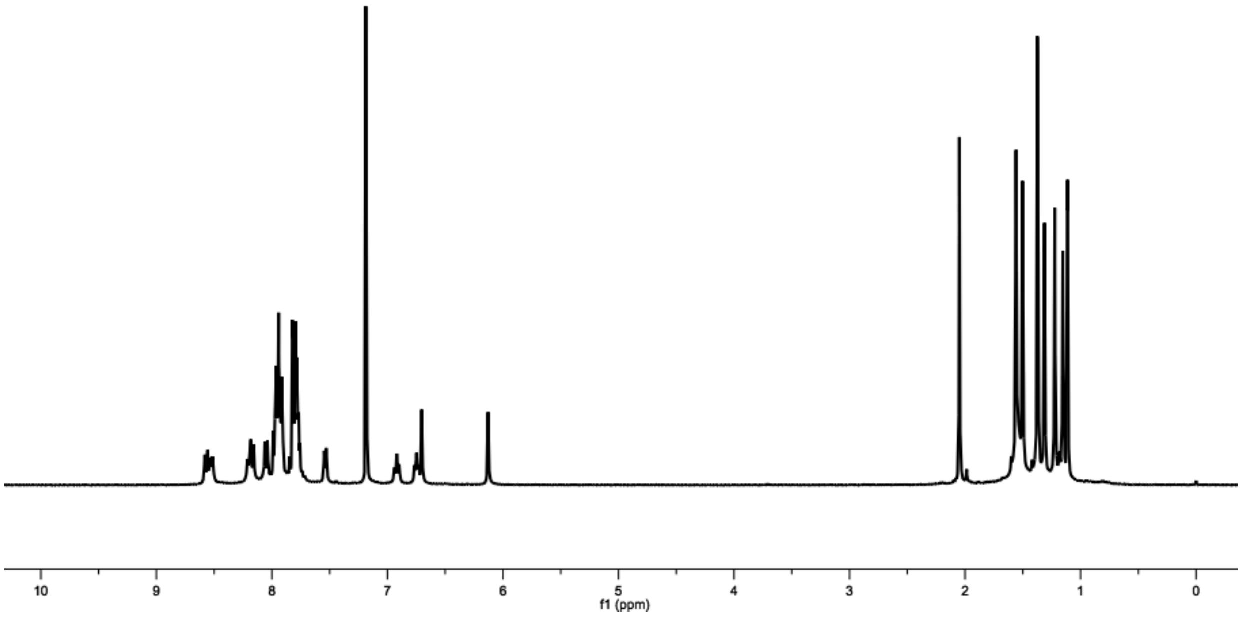

^{13}C NMR spectrum of N_3Aib_4(*S,S*-BisPyrEt)NHAc 43

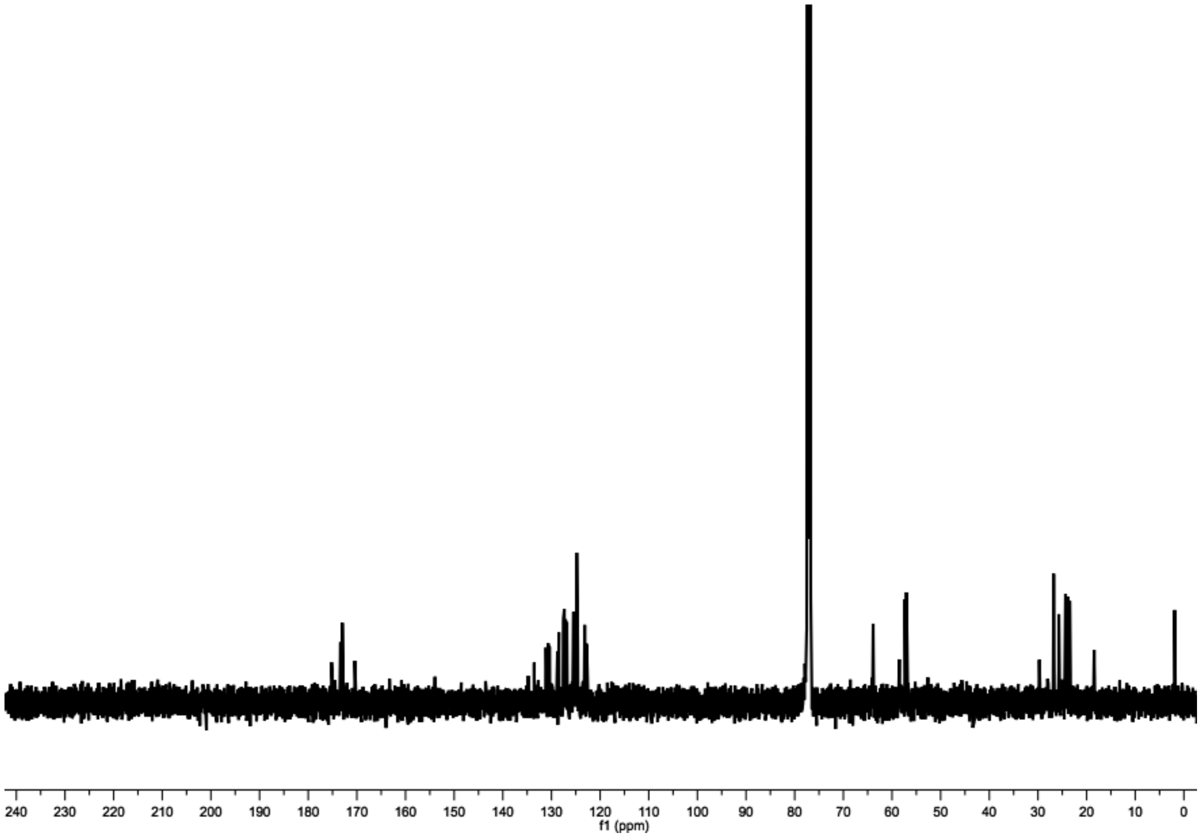

^{1}H NMR spectrum of Cbz(D-Phe)Aib$_4$(*S*,*S*-BisPyrEt)NHAc 44

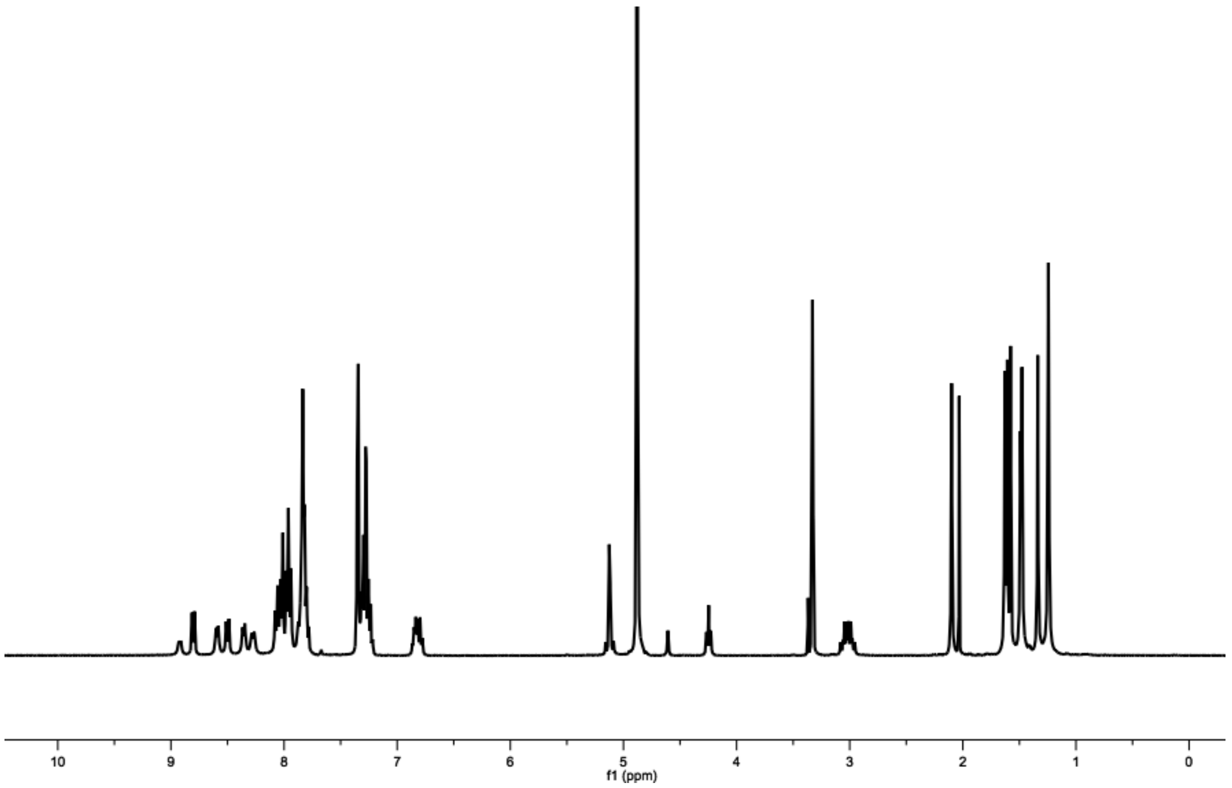

^{13}C NMR spectrum of Cbz(D-Phe)Aib$_4$(*S*,*S*-BisPyrEt)NHAc 44

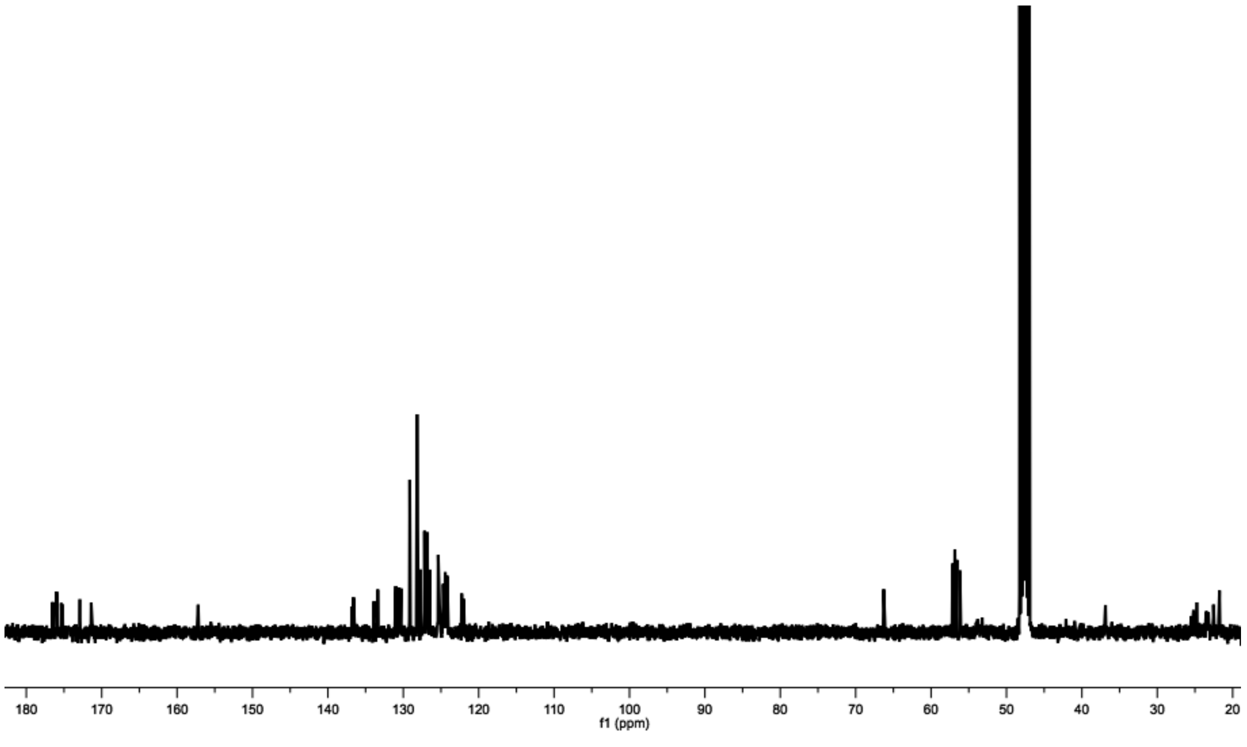

^{1}H NMR spectrum of (1*R*,2*R*)-1,2-(1-pyrene)ethylenediamine dihydrochloride 46

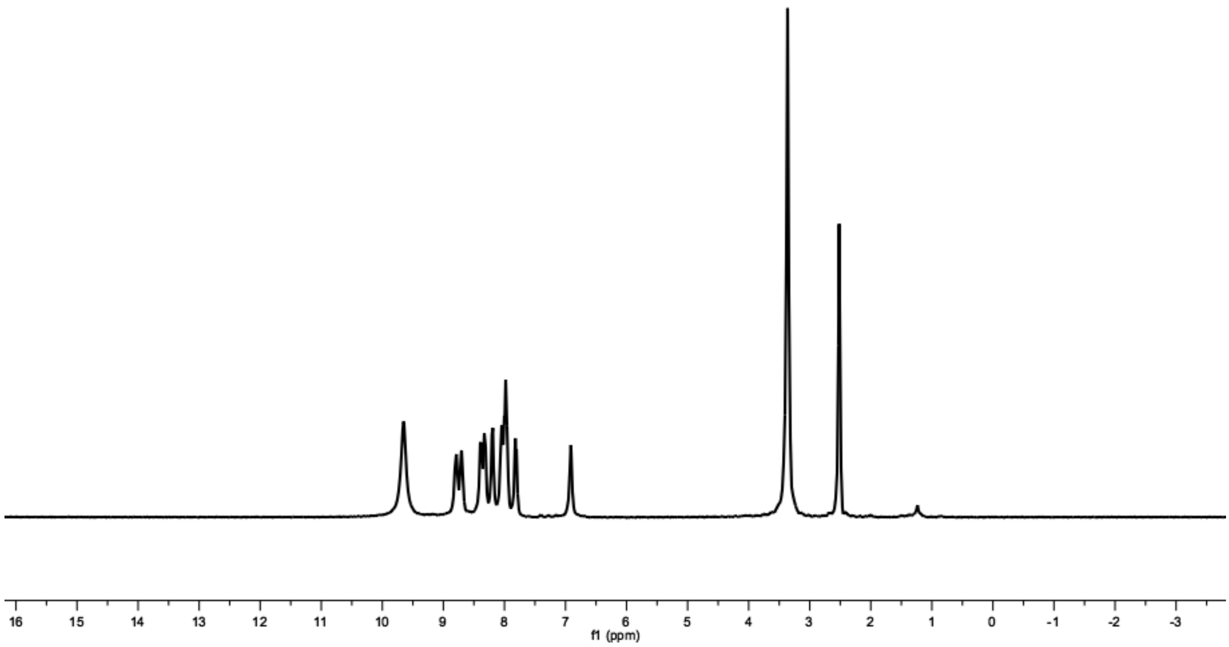

^{13}C NMR spectrum of (1*R*,2*R*)-1,2-(1-pyrene)ethylenediamine dihydrochloride 46

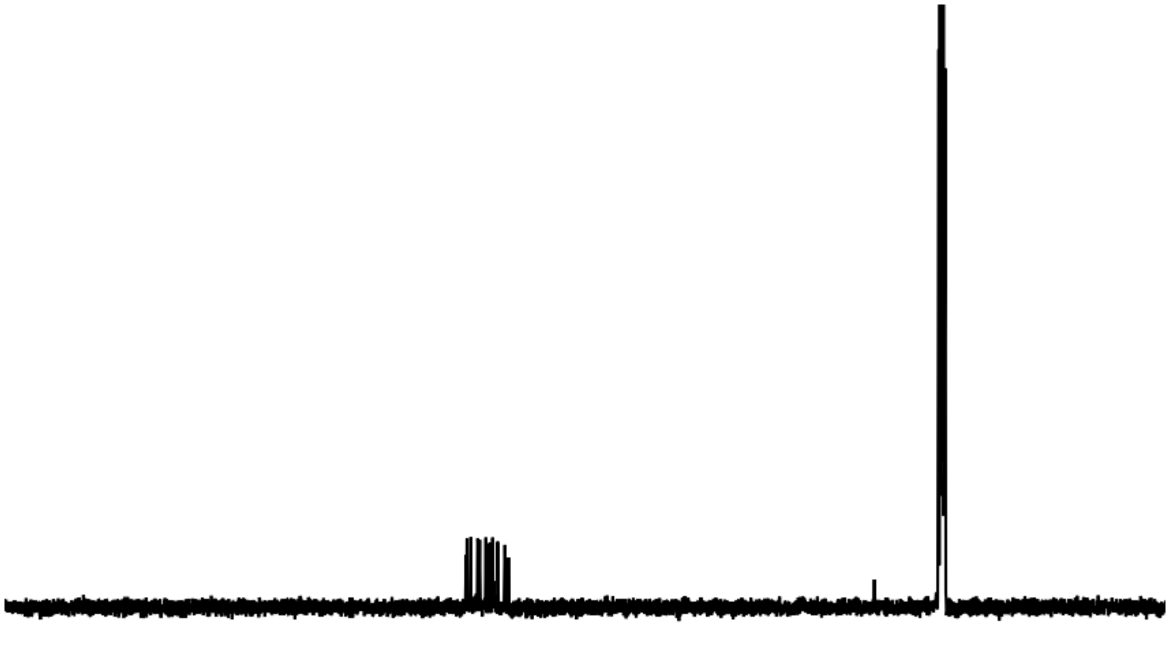

^{1}H NMR spectrum of N_3Aib_4(*R*,*R*-BisPyrEt)NHAc 47

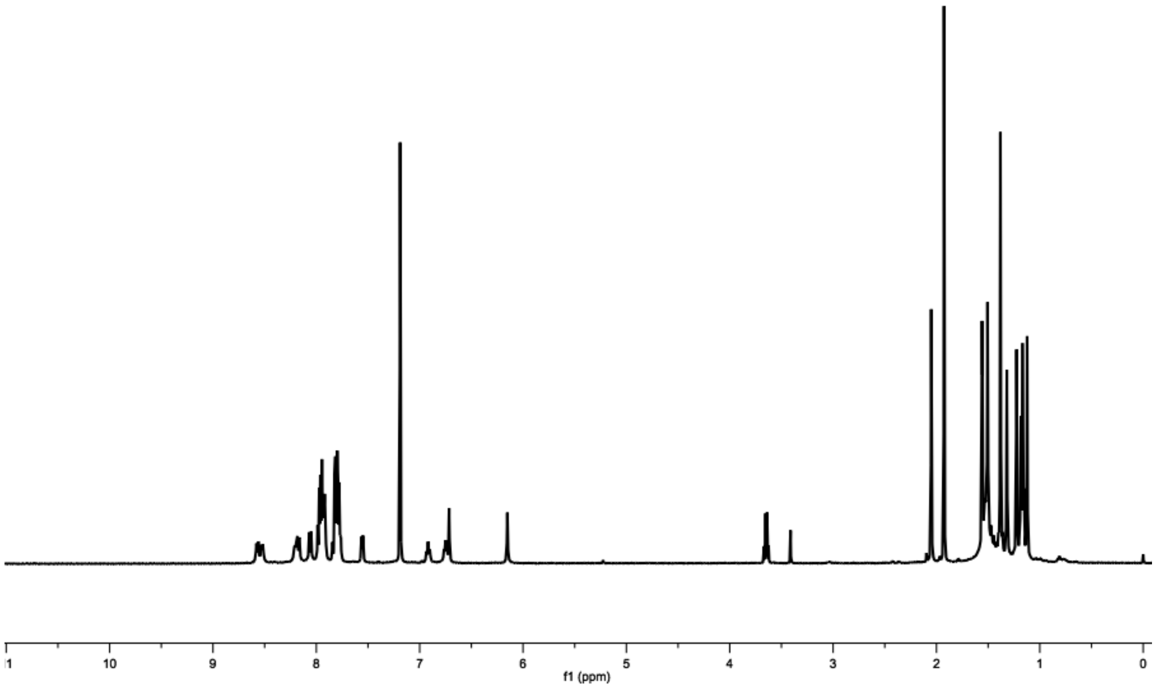

^{13}C NMR spectrum of N_3Aib_4(*R*,*R*-BisPyrEt)NHAc 47

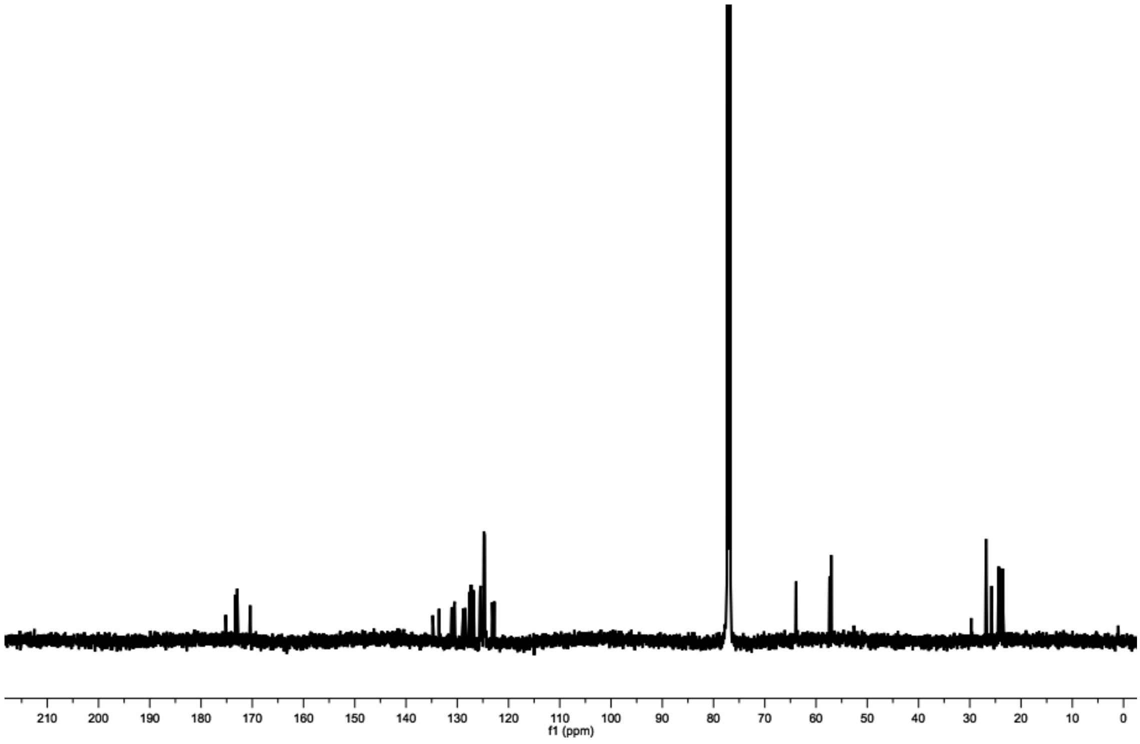

^{1}H NMR spectrum of Cbz(D-Phe)Aib$_4$(*R*,*R*-BisPyrEt)NHAc 48

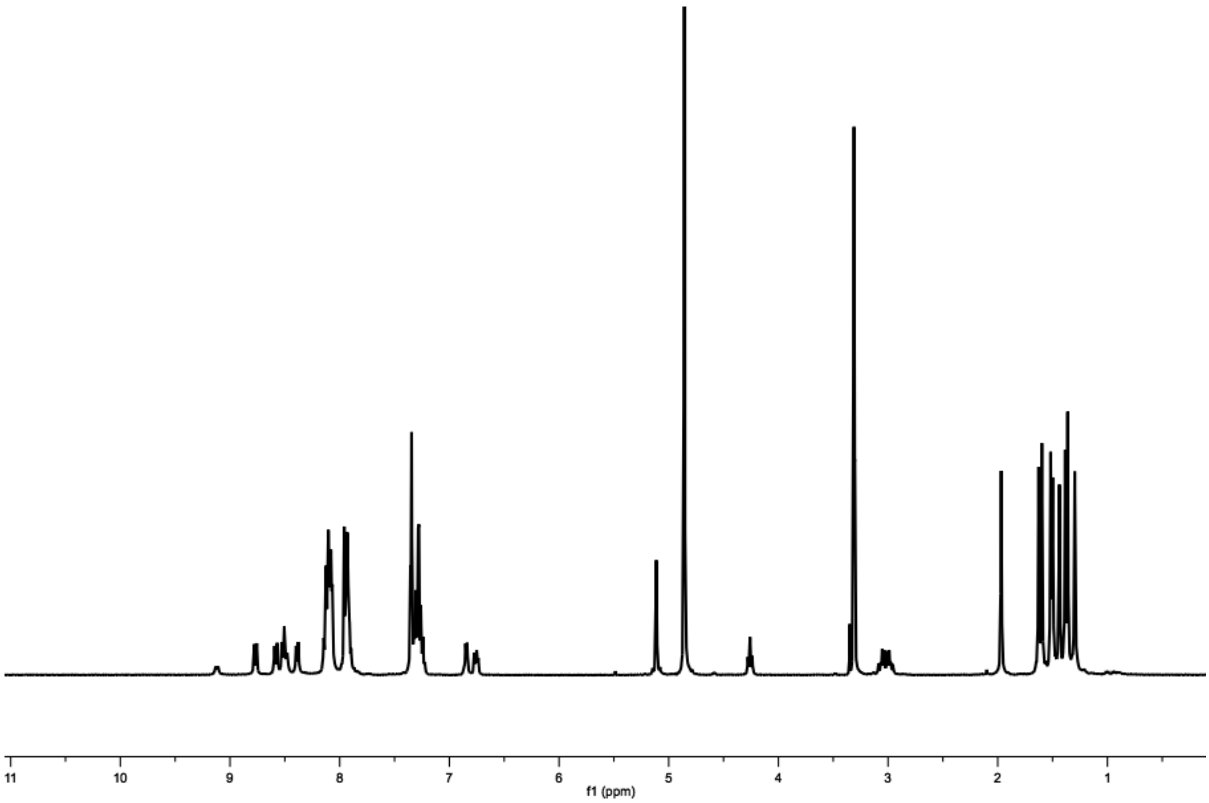

^{13}C NMR spectrum of Cbz(D-Phe)Aib$_4$(*R*,*R*-BisPyrEt)NHAc 48

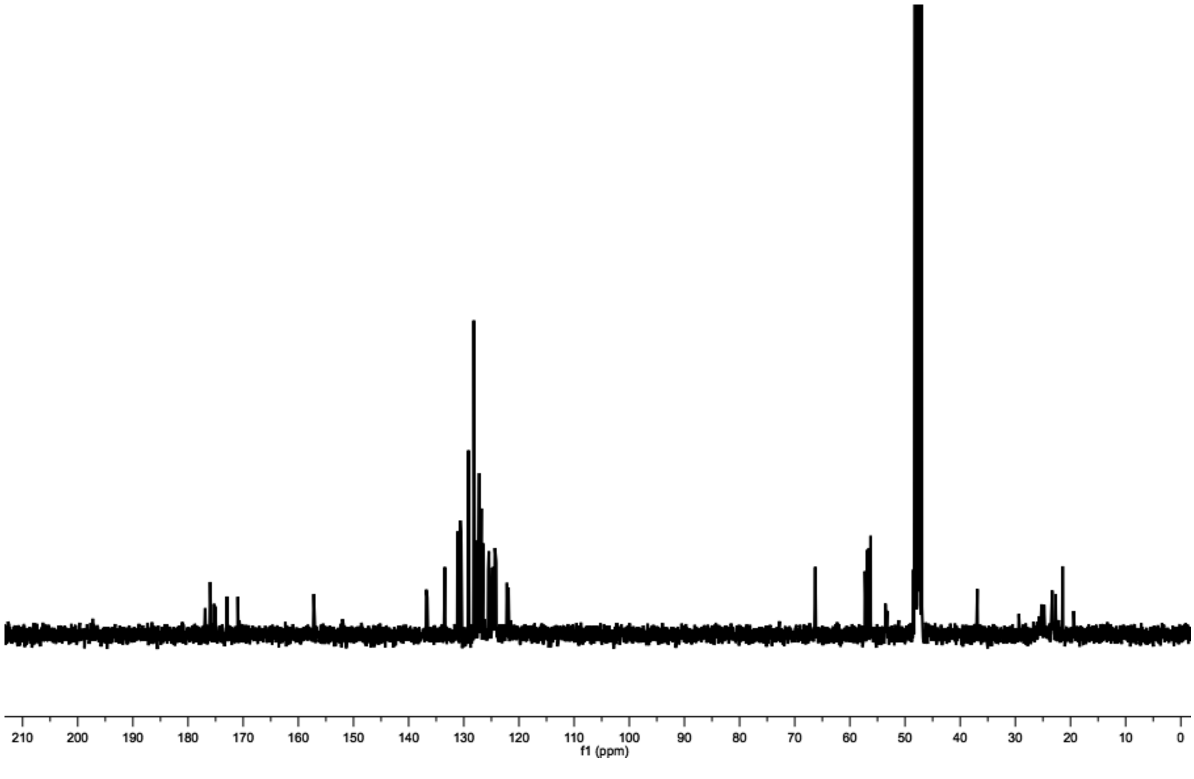

^{1}H NMR spectrum of Cbz(L-αMeVal)Aib$_4$(*R*,*R*-BisPyrEt)NHAc 49

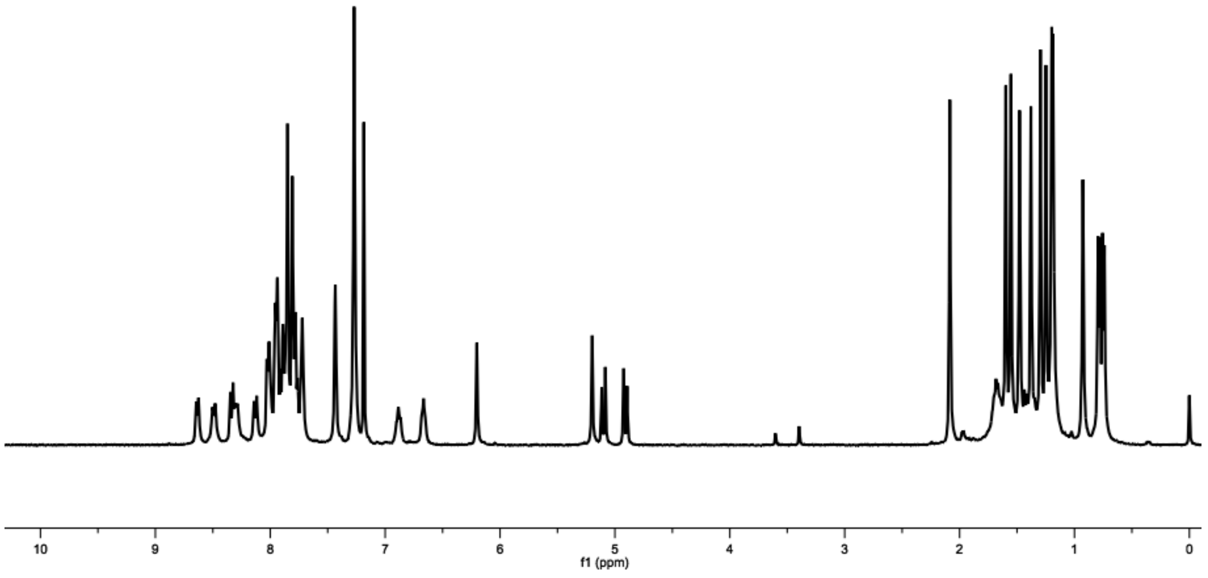

^{13}C NMR spectrum of Cbz(L-αMeVal)Aib$_4$(*R*,*R*-BisPyrEt)NHAc 49

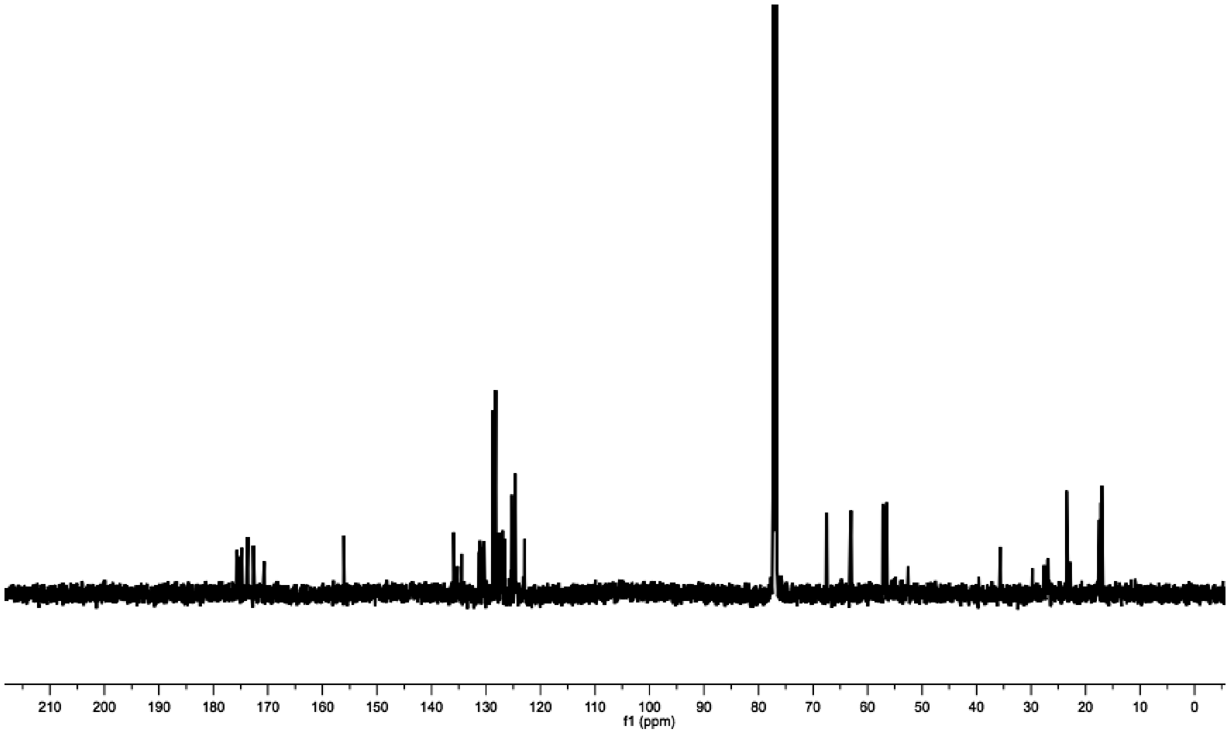

6. X-ray crystal structure data

The X-ray data for **13**, **43, 48 49** and **53** have been deposited with the Cambridge Crystallographic Data Centre.

CCDC 1843678 – 1843682 contain the supplementary crystallographic data for this paper. These data can be obtained free of charge from The Cambridge Crystallographic Data Centre via www.ccdc.cam.ac.uk/data_request/cif.

6.1. Crystal data and refinement for 13

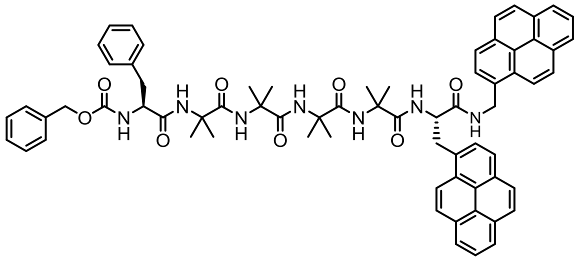

Single crystals suitable for X-ray diffraction analysis were grown by slow evaporation from $CDCl_3$. Data were collected on a were collected on a Bruker X8 prospector diffractometer with an Apex II CCD detector and a Incoatec IμS 1.0 CuK_α Microfocus Source (λ = 1.54184 Å), at a temperature of 100 K. Data were reduced using Bruker SAINT and absorption correction was performed using empirical methods (Bruker SADABS) based upon symmetry-equivalent reflections combined with measurements at different azimuthal angles.[11] The structure was solved and refined against F^2 using Shelx-20XX implemented through Olex2 v1.2.7.[13]

Table S2: Crystal data and structure refinement for **13**

Identification code	s3812ma
Empirical formula	$C_{72}H_{72}Cl_9N_7O_8$
Formula weight	1482.41
Temperature/K	100.15
Crystal system	orthorhombic
Space group	$P2_12_12_1$
a/Å	13.2969(4)

b/Å	22.3943(5)
c/Å	25.1034(6)
α/°	90
β/°	90
γ/°	90
Volume/$Å^3$	7475.2(3)
Z	4
ρ_{calc}g/cm^3	1.317
μ/mm^{-1}	3.549
F(000)	3080.0
Crystal size/mm^3	0.33 × 0.22 × 0.17
Radiation	CuKα (λ = 1.54178)
2Θ range for data collection/°	7.732 to 140.282
Index ranges	-16 ≤ h ≤ 12, -18 ≤ k ≤ 27, -30 ≤ l ≤ 30
Reflections collected	35359
Independent reflections	13829 [R_{int} = 0.0414, R_{sigma} = 0.0542]
Data/restraints/parameters	13829/0/873
Goodness-of-fit on F^2	1.033
Final R indexes [I>=2σ (I)]	R_1 = 0.0620, wR_2 = 0.1603
Final R indexes [all data]	R_1 = 0.0733, wR_2 = 0.1709
Largest diff. peak/hole / e $Å^{-3}$	1.12/-0.82
Flack parameter	0.016(8)

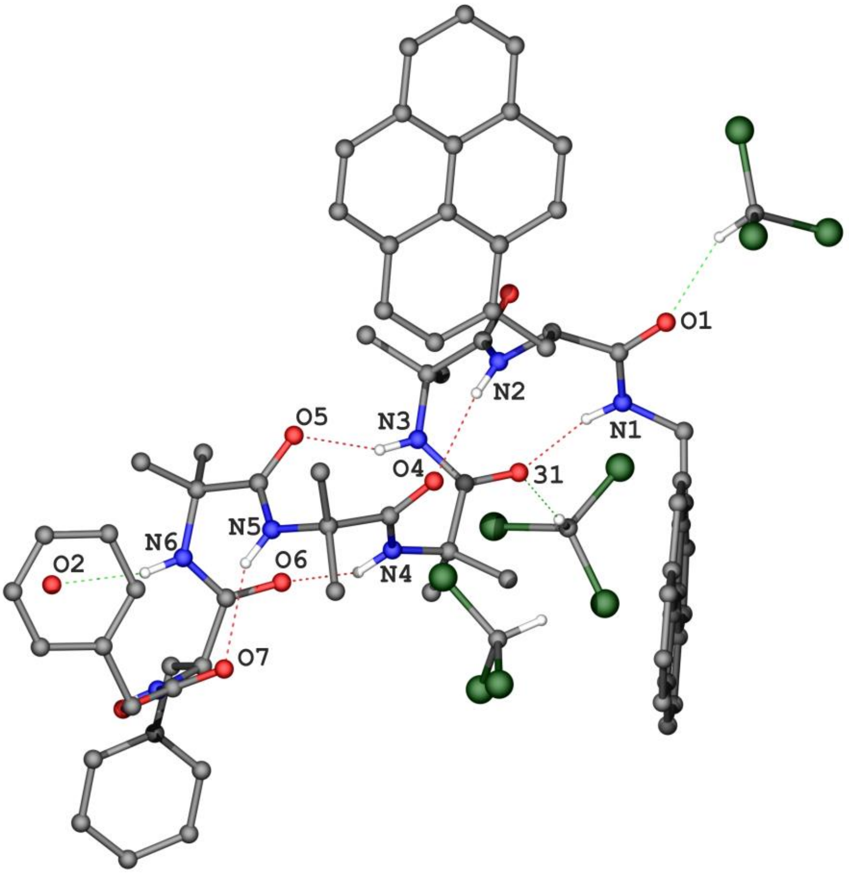

Figure S13: Hydrogen bonds for **13** with distances up to 3 Å [Å]. Methyl and methylene hydrogens and disorder on pyrene and phenyl moieties and non-H-bonding solvent removed for clarity. Grey = C, Blue = N, Red = O, White = H, dark green = Cl, dashed green = intramolecular H-bonding, dashed red = intermolecular H-bonding

Table S3: Hydrogen bonds for **13** with distances up to 3 Å [Å]. D-H distances normalised to neutron diffraction determined distances and calculated using Mercury.[14]

H bond Number	Atom 1	Atom 2	Length / Å	Length-VdW / Å
1	H1	O3	1.922	-0.798
2	H2	O4	2.188	-0.532
3	H3A	O5	2.017	-0.703
4	H4A	O6	2.05	-0.67
5	H5	O7	2.129	-0.591
6 (intermol.)	O2	H6A	1.933	-0.787

6.2. Crystal data and structure refinement for **43**

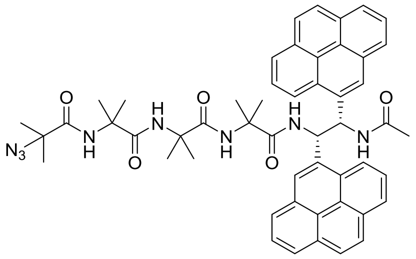

Single crystals suitable for X-ray diffraction analysis were grown by slow evaporation from $CDCl_3$. Data were collected on a dual source Rigaku FR-X rotating anode diffractometer using CuKα radiation (λ = 1.54184 Å) at a temperature of 150K. The data were reduced using CrysAlisPro version 171.39.30c and absorption correction was performed using empirical methods (SCALE3 ABSPACK) based upon symmetry-equivalent reflections combined with measurements at different azimuthal angles.[12] The structure was solved and refined against F^2 using Shelx-2017/1 implemented through Olex2 v1.2.9.[13]

Table S4: Crystal data and structure refinement for **43**

Identification code	s5080l
Empirical formula	$C_{70.57}H_{83.27}N_7O_{9.57}$
Formula weight	1182.59
Temperature/K	150.06(10)
Crystal system	orthorhombic
Space group	$P2_12_12_1$
a/Å	10.5967(4)
b/Å	21.5835(8)
c/Å	28.6986(6)
α/°	90
β/°	90
γ/°	90
Volume/Å^3	6563.8(4)
Z	4
ρ_{calc}g/cm^3	1.197

μ/mm^{-1}	0.641
F(000)	2529.0
Crystal size/mm^3	0.442 × 0.055 × 0.044
Radiation	CuKα (λ = 1.54184)
2Θ range for data collection/°	5.124 to 136.472
Index ranges	-12 ≤ h ≤ 9, -26 ≤ k ≤ 26, -34 ≤ l ≤ 32
Reflections collected	67099
Independent reflections	12007 [R_{int} = 0.0731, R_{sigma} = 0.0534]
Data/restraints/parameters	12007/2988/1128
Goodness-of-fit on F^2	1.061
Final R indexes [I>=2σ (I)]	R_1 = 0.0687, wR_2 = 0.1928
Final R indexes [all data]	R_1 = 0.0806, wR_2 = 0.2035
Largest diff. peak/hole / e Å^{-3}	0.27/-0.19

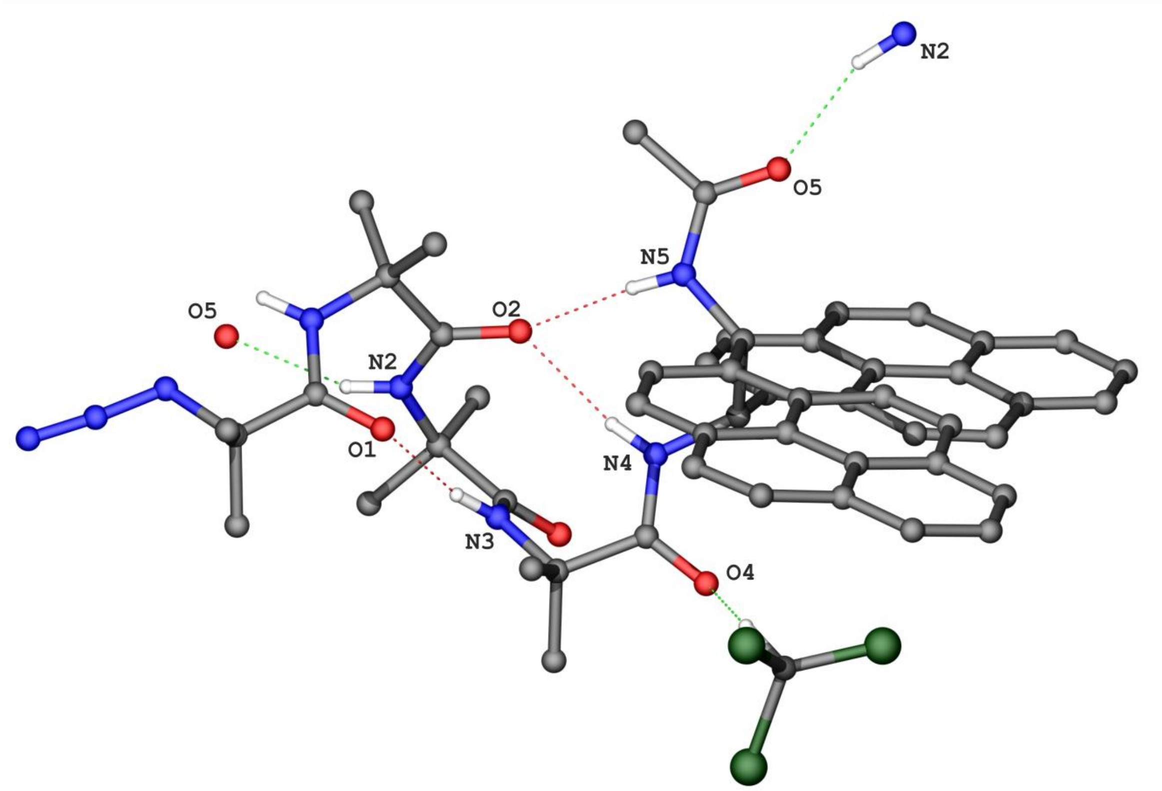

Figure S14: Hydrogen bonds for **43** with distances up to 3 Å [Å]. Methyl and methylene hydrogens and non-H-bonding solvent removed for clarity. Grey = C, Blue = N, Red = O, White = H, dark green = Cl, dashed green = intramolecular H-bonding, dashed red = intermolecular H-bonding

Table S5: Hydrogen bonds for **43** with distances up to 3 Å [Å]. D-H distances normalised to neutron diffraction determined distances and calculated using Mercury.[14]

H bond Number	Atom 1	Atom 2	Length / Å	Length-VdW / Å
1	O59	H45	2.075	-0.645
2	O73	H57	1.903	-0.817
3	O65	H51	2.067	-0.653
4	O53	H38	1.947	-0.773
5	O53	H39	2.173	-0.547
6 (intermol.)	O37	H71	1.906	-0.814
7 (intermol.)	H63	O2B	2.197	-0.523
8 (intermol.)	O37	H2B	2.011	-0.709

6.3. Crystal data and structure refinement for 48

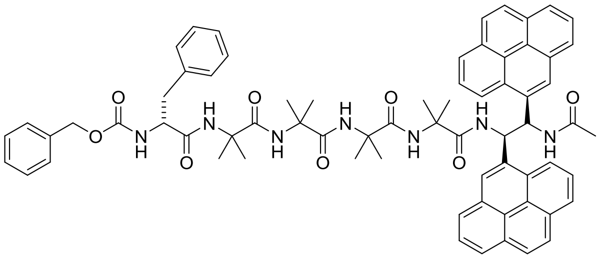

Single crystals suitable for X-ray diffraction analysis were grown by slow evaporation from CD_3OD. Data were collected on a dual source Rigaku FR-X rotating anode diffractometer using CuKα radiation (λ = 1.54184 Å) at a temperature of 150K. The data were reduced using CrysAlisPro version 171.39.45i and absorption correction was performed using empirical methods (SCALE3 ABSPACK) based upon symmetry-equivalent reflections combined with measurements at different azimuthal angles.[12] The structure was solved and refined against F^2 using Shelx-2018/1 implemented through Olex2 v1.2.9.[13]

Table S6: Crystal data and structure refinement for **48**

Identification code	s5190l
Empirical formula	$C_{70.79}H_{77.17}N_7O_{10.4}$
Formula weight	1192.41
Temperature/K	150.02(10)
Crystal system	orthorhombic
Space group	$P2_12_12_1$
a/Å	22.0413(3)
b/Å	23.2593(4)
c/Å	25.4959(5)
α/°	90
β/°	90
γ/°	90
Volume/Å^3	13070.9(4)

Z	8
ρ_{calc}g/cm^3	1.212
μ/mm^{-1}	0.660
F(000)	5073.0
Crystal size/mm^3	0.187 × 0.119 × 0.05
Radiation	CuKα (λ = 1.54184)
2Θ range for data collection/°	5.142 to 142.822
Index ranges	-26 ≤ h ≤ 26, -27 ≤ k ≤ 22, -31 ≤ l ≤ 31
Reflections collected	138717
Independent reflections	25055 [R_{int} = 0.1027, R_{sigma} = 0.0701]
Data/restraints/parameters	25055/2552/2048
Goodness-of-fit on F^2	1.015
Final R indexes [I>=2σ (I)]	R_1 = 0.0608, wR_2 = 0.1617
Final R indexes [all data]	R_1 = 0.0734, wR_2 = 0.1757
Largest diff. peak/hole / e Å^{-3}	0.54/-0.29
Flack parameter	-0.08(11)

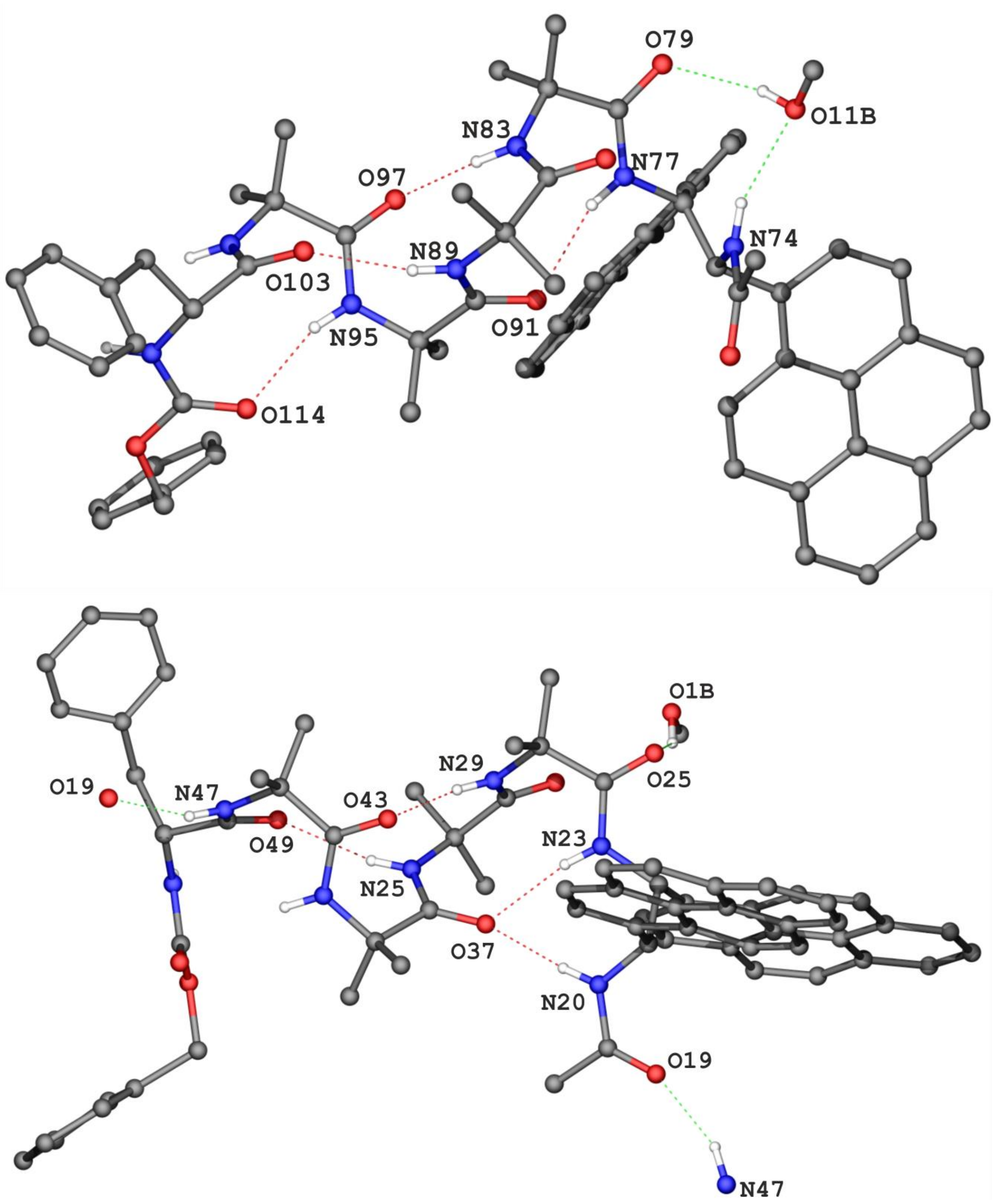

Figure S15: Hydrogen bonds for both species in the asymmetric unit of **48** (displayed separately) with distances up to 3 Å [Å]. Methyl and methylene hydrogens and disorder on pyrene and phenyl moieties and non-H-bonding solvent removed for clarity. Grey = C, Blue = N, Red = O, White = H, dashed green = intramolecular H-bonding, dashed red = intermolecular H-bonding.

Table S7: Hydrogen bonds for **48** with distances up to 3 Å [Å]. D-H distances normalised to neutron diffraction determined distances and calculated using Mercury.[14]

H bond Number	Atom 1	Atom 2	Length / Å	Length-VdW / Å
1	O37	H20	1.924	-0.796
2	O37	H23	2.028	-0.692
3	O49	H35	2.008	-0.712
4	H29	O43	1.943	-0.777
5 (intermol.)	O19	H47	1.963	-0.757
6 (intermol.)	H58	O79	2.322	-0.398
7 (intermol.)	O25	H101	1.898	-0.822
8 (intermol.)	O25	H1B	2.488	-0.232
9 (intermol.)	O43	H3B	2.194	-0.526
10	O103	H89	2.06	-0.66
11	O97	H83	1.959	-0.761
12	H77	O91	2.131	-0.589
13	O114	H95	2.09	-0.63
14 (intermol.)	H112	O1B	1.928	-0.792
15 (intermol.)	O91	H15B	2.21	-0.51
16 (intermol.)	H74	O13B	2.005	-0.715
17 (intermol.)	O3B	H9BA	2.29	-0.43

*This table is incomplete due to the disorder in the structure and the way Mercury deals with disorder.

6.4. Crystal data and structure refinement for 49

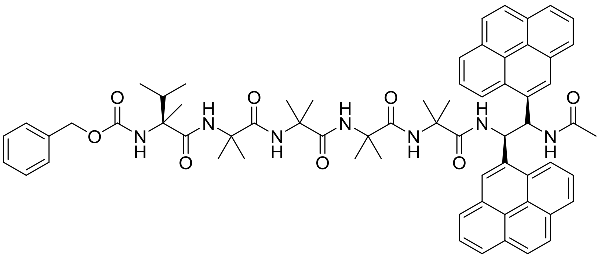

Single crystals suitable for X-ray diffraction analysis were grown by slow diffusion of diethyl ether into methanol. Data were collected on a dual source Rigaku FR-X rotating anode diffractometer using CuKα radiation (λ = 1.54184 Å) at a temperature of 150K. The data were reduced using CrysAlisPro version 171.39.30c and absorption correction was performed using empirical methods (SCALE3 ABSPACK) based upon symmetry-equivalent reflections combined with measurements at different azimuthal angles.[12] The structure was solved and refined against F^2 using Shelx-2017/1 implemented through Olex2 v1.2.9.[13]

Table S8: Crystal data and structure refinement for **49**

Identification code	s5108r
Empirical formula	$C_{53}H_{53}Cl_3N_8O_5$
Formula weight	988.38
Temperature/K	149.99(10)
Crystal system	monoclinic
Space group	$P2_1$
a/Å	9.7198(6)
b/Å	18.6592(11)
c/Å	14.0802(12)
α/°	90
β/°	90.161(7)
γ/°	90
Volume/Å^3	2553.6(3)

Z	2
ρ_{calc}g/cm^3	1.285
μ/mm^{-1}	2.069
F(000)	1036.0
Crystal size/mm^3	0.213 × 0.05 × 0.031
Radiation	CuKα (λ = 1.54184)
2Θ range for data collection/°	6.278 to 148.998
Index ranges	-12 ≤ h ≤ 12, -23 ≤ k ≤ 23, -17 ≤ l ≤ 17
Reflections collected	30504
Independent reflections	10270 [R_{int} = 0.0636, R_{sigma} = 0.0642]
Data/restraints/parameters	10270/76/654
Goodness-of-fit on F^2	1.034
Final R indexes [I>=2σ (I)]	R_1 = 0.0614, wR_2 = 0.1542
Final R indexes [all data]	R_1 = 0.0884, wR_2 = 0.1719
Largest diff. peak/hole / e Å^{-3}	0.31/-0.44

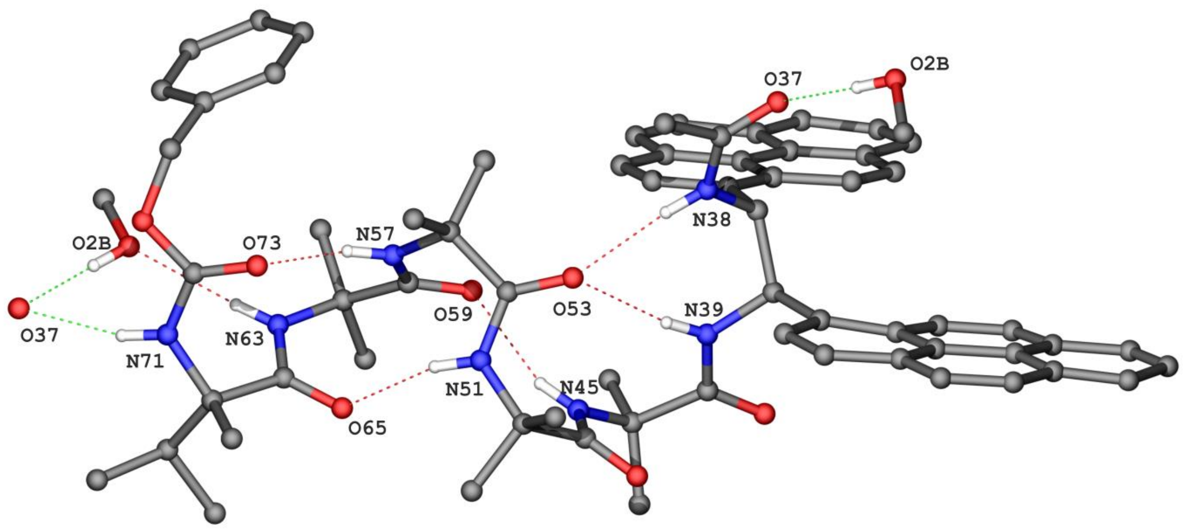

Figure S16: Hydrogen bonds for **52** with distances up to 3 Å [Å]. Methyl and methylene hydrogens removed for clarity. Grey = C, Blue = N, Red = O, White = H, Green = Cl, dashed green = intramolecular H-bonding, dashed red = intermolecular H-bonding

Table S9: Hydrogen bonds for **49** with distances up to 3 Å [Å]. D-H distances normalised to neutron diffraction determined distances and calculated using Mercury.[14]

H bond Number	Atom 1	Atom 2	Length / Å	Length-VdW / Å
1	O1	H3	2.072	-0.648
2	O2	H4	1.98	-0.74
3	O2	H5	2.002	-0.718
4 (intermol.)	H2	O5	2.095	-0.625

6.5. Crystal data and structure refinement for 53

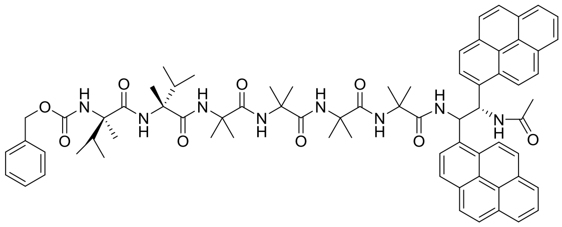

Single crystals suitable for X-ray diffraction analysis were grown by by slow evaporation from $CDCl_3$. Data were collected on a were collected on a Bruker X8 prospector diffractometer with an Apex II CCD detector and a Incoatec IμS 1.0 CuK_α Microfocus Source (λ = 1.54184 Å), at a temperature of 150 K. Data were reduced using CrysAlisPro 171.38.43 and absorption correction was performed using empirical methods (SCALE3 ABSPACK) based upon symmetry-equivalent reflections combined with measurements at different azimuthal angles.[12] The structure was solved and refined against F^2 using Shelx-2016 implemented through Olex2 v1.2.9.[13]

Table S10: Crystal data and structure refinement for **53**

Identification code	s4270
Empirical formula	$C_{72}H_{87.5}N_8O_{11.75}$
Formula weight	1252.99
Temperature/K	150.0
Crystal system	monoclinic
Space group	$P2_1$
a/Å	15.5782(6)
b/Å	18.4753(5)
c/Å	24.4315(7)
α/°	90
β/°	91.976(3)
γ/°	90

Volume/Å^3	7027.5(4)
Z	4
ρ_{calc}g/cm^3	1.184
μ/mm^{-1}	0.654
F(000)	2678.0
Crystal size/mm^3	0.24 × 0.21 × 0.01
Radiation	CuKα (λ = 1.54184)
2Θ range for data collection/°	3.618 to 136.97
Index ranges	-18 ≤ h ≤ 18, -22 ≤ k ≤ 21, -29 ≤ l ≤ 28
Reflections collected	47191
Independent reflections	20163 [R_{int} = 0.0999, R_{sigma} = 0.0865]
Data/restraints/parameters	20163/5703/2256
Goodness-of-fit on F^2	0.981
Final R indexes [I>=2σ (I)]	R_1 = 0.0579, wR_2 = 0.1297
Final R indexes [all data]	R_1 = 0.1089, wR_2 = 0.1572
Largest diff. peak/hole / e Å^{-3}	0.19/-0.18
Flack parameter	-0.2(2)

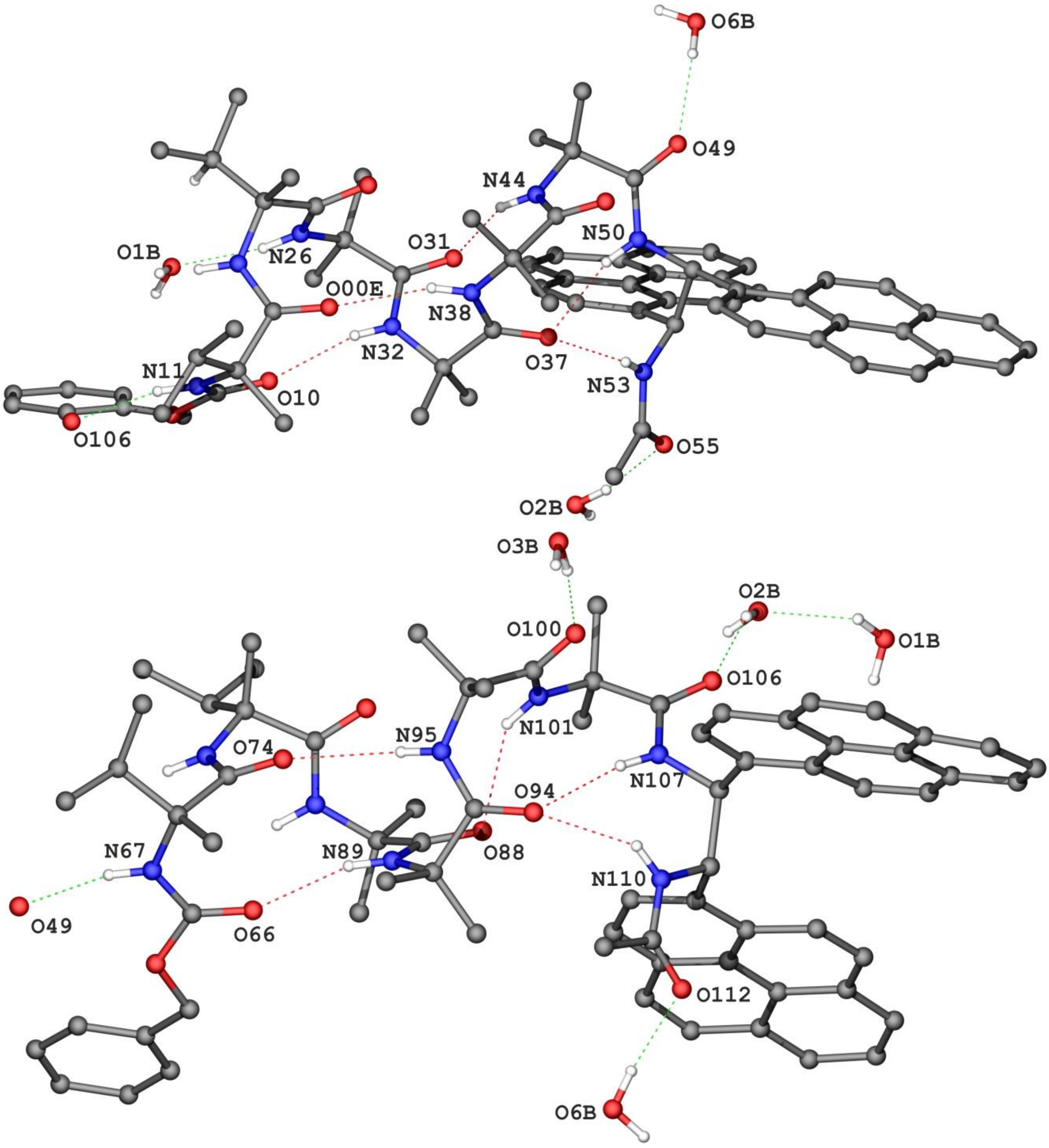

Figure S17: Hydrogen bonds for both species in the asymmetric unit of **48** (displayed separately) with distances up to 3 Å [Å]. Methyl and methylene hydrogens and disorder on pyrene and phenyl moieties and non-H-bonding solvent removed for clarity. Grey = C, Blue = N, Red = O, White = H, dashed green = intramolecular H-bonding, dashed red = intermolecular H-bonding.

Table S11: Hydrogen bonds for **53** with distances up to 3 Å [Å]. D-H distances normalised to neutron diffraction determined distances and calculated using Mercury.[14]

H bond Number	Atom 1	Atom 2	Length / Å	Length-VdW / Å
1	O94	H107	2.165	-0.555
2	O94	H110	2.115	-0.605
3	O82	H95	2.692	-0.028
4	O66	H89	1.995	-0.725
5	O88	H101	2.213	-0.507
6	O74	H95	2.252	-0.468
7 (intermol.)	O106	H11	1.867	-0.853
8 (intermol.)	H67	O49	1.846	-0.874
9 (intermol.)	O106	H2BB	2.021	-0.699
10 (intermol.)	H83	O5B	2.099	-0.621
11 (intermol.)	O112	H6BA	1.997	-0.723
12 (intermol.)	H75	O6B	2.554	-0.166
13 (intermol.)	O100	H3BA	2.095	-0.625
14	O37	H50	2.136	-0.584
15	O37	H53	1.955	-0.765
16	O25	H38	2.681	-0.039
17	O31	H44	2.153	-0.567
18	O00E	H38	2.285	-0.435
19 (intermol.)	O10	H32	1.997	-0.723
20 (intermol.)	H26	O1B	2.005	-0.715
21 (intermol.)	O55	H2BA	1.89	-0.83
22 (intermol.)	H18	O2B	2.692	-0.028
23 (intermol.)	O49	H6BB	1.91	-0.81
24 (intermol.)	H1BA	O2B	1.956	-0.764
25 (intermol.)	H5BA	O6B	1.764	-0.956

*This table is incomplete due to the disorder in the structure and the way Mercury deals with disorder.

7. References

1. H. Mihara, Y. Tanaka, T. Fujimoto and N. Nishino, *J. Chem. Soc. Perkin Trans. 2*, 1995, 1133; (b) S. Egusa, M. Sisido and Y. Imanishi, *Macromolecules*, 1985, **18**, 882. (c) *Private communication*, Prof. M. Sisido, Okayama University; (d) E. Soto, J. C. MacDonald, C. G. F.; Cooper and W. G. McGimpsey, *J. Am. Chem. Soc.*, 2003, **125**, 2838.
2. W. Chen, N. B. Zuckerman, J. W. Lewis, J. P. Konopelski and S. Chen, *J. Phys. Chem. C,* 2009, **113**, 16988.
3. A. Breuning, R. Vicik, and T. Schirmeister, *Tetrahedron: Asymmetry* 2003, *14*, 3301-3312.
4. L. Byrne, J. Solà, T. Boddaert, T. Marcelli, R. W. Adams, G. A. Morris and J. Clayden, *Angew. Chem. Int. Ed.,* 2014, **53**, 151.
5. S. J. Pike, V. Diemer, J. Raftery, S. J. Webb and J. Clayden, *Chem Eur. J.,* 2014, **20**, 15981.
6. F. G. A. Lister, B. A. F. LeBailly, S. J. Webb and J. Clayden, *Nature Chem*., 2017, **9**, 420.
7. H. Kim, Y. Nguyen, C. P.-H. Yen, L. Chagal, A. J. Lough, B. M. Kim and J. Chin, *J. Am. Chem. Soc.,* 2008, **130**, 12184.
8. J. Clayden, A. Castellaños and J. Solà, *Angew. Chem. Int. Ed.,* 2009, **48**, 5962.
9. (a) M. I. Angelova and D. S. Dimitrov, *Faraday Discuss. Chem. Soc.* 1986, **81**, 303-311. (b) G. T. Noble, F. L. Craven, J. Voglmeir, R. Šardzík, S. L. Flitsch and S. J. Webb, *J. Am. Chem. Soc.* 2012, **134**, 13010-13017.
10. M. De Poli, L. Byrne, R. A. Brown, J. Solà, A. Castellanos, T. Boddaert, R. Wechsel, J. D. Beadle and J. Clayden, *J. Org. Chem.* 2014, **79**, 4659.
11. Bruker (2012). *SAINT and SADABS*. Bruker AXS Inc., Madison, Wisconsin, USA.
12. Rigaku Oxford Diffraction, 2017, CrysAlisPro Software system, version 1.171.39.21a, Rigaku Corporation, Oxford, UK
13. (a) G. M. Sheldrick, *Acta Cryst*. 2015, **A71**, 3-8.; (b) G. M. Sheldrick, *Acta Cryst*. 2015, **C71**, 3-8.; (c) O. V. Dolomanov, L. J. Bourhis, R. J. Gildea, J. A. K. Howard and H. Puschmann, *J. Appl. Cryst*. 2009, **42**, 339-341.
14. C. F. Macrae, I. J. Bruno, J. A. Chisholm, P. R. Edgington, P. McCabe, E. Pidcock, L. Rodriguez-Monge, R. Taylor, J. van de Streek and P. A. Wood, *J. Appl. Cryst*., 2008, **41**, 466-470.
